# Maternal hyperglycemia-induced *O*-GlcNAcylation of CaMKIIδ promotes mtDNA release and cardiac remodeling in offspring

**DOI:** 10.1038/s41467-026-75630-4

**Published:** 2026-07-29

**Authors:** Zemeng Xiao, Ling Gao, Yuehong Wang, Chunli Yang, Yuxuan Qian, Lei Chen, Xuxia Li, Na Geng, Taiwei Chen, Ancai Yuan, Liuhua Hu, Phung N. Thai, Hao Li, Ling Gao, Zhice Xu, Xiyuan Lu, Jun Pu

**Affiliations:** 1 Department of Cardiology, Ren Ji Hospital, School of Medicine, State Key Laboratory of Systems Medicine for Cancer, Shanghai Cancer Institute, Shanghai Jiao Tong University, Shanghai, China; 2 Institute of Reproduction and Development, Shanghai Key Laboratory of Reproduction and Development, Obstetrics and Gynecology Hospital, Fudan University, Shanghai, China; 3 Institute for Fetology, First Hospital of Soochow University, Suzhou, China; 4 State Key Laboratory of Systems Medicine for Cancer, Department of Urology, Ren Ji Hospital, Shanghai Cancer Institute, Shanghai Jiao Tong University, School of Medicine, Shanghai, China; 5 Department of Internal Medicine, University of California, Davis, CA USA; 6 Department of Veterans Affairs, Northern California Health Care System, Mather, CA USA; 7 Institute for Regenerative Medicine, State Key Laboratory of Cardiology and Medical Innovation Center, Shanghai East Hospital, School of Medicine, Tongji University, Shanghai, China; 8 The First Affiliated Hospital of Soochow University, Suzhou, China

**Keywords:** Mitochondria, Glycosylation, Cardiology

## Abstract

Maternal diabetes during pregnancy increases the risk of metabolic and cardiac disorders in offspring. Nevertheless, the mechanism by which intrauterine hyperglycemia affects neonatal cardiac remodeling remains uncertain. This study aims to characterize the prenatal environment in the context of gestational diabetes mellitus and to identify the corresponding fetal changes. Using an intrauterine hyperglycemia rodent model, we observe cardiac remodeling and inflammatory responses in offspring hearts. Moreover, the *O*-GlcNAcylation levels are increased in neonatal hearts exposed to gestational diabetes. Further mechanistic investigations, supported by RNA sequencing and mitochondrial functional analyses, reveal that gestational diabetes triggers *O*-GlcNAcylation-dependent activation of CaMKIIδ during the embryonic stage. This activation leads to the release of mitochondrial DNA (mtDNA) from the mitochondrial matrix into the cytosol, which subsequently activates STING signaling and triggers an inflammatory response in neonatal cardiomyocytes. Pharmacological or genetic inhibition of *O*-GlcNAcylation attenuates mtDNA-induced myocardial inflammation and improves cardiac function in neonatal offspring subjected to intrauterine hyperglycemia. Together, these findings identify a previously unrecognized CaMKIIδ/mtDNA/STING axis in which CaMKIIδ *O*-GlcNAcylation leads to mtDNA-dependent activation of cardiac remodeling, suggesting that plasma mtDNA levels could serve as a predictive biomarker in neonatal cardiac inflammatory injury.

## Abstract (web summary)

The study demonstrates that maternal hyperglycemia activates CaMKIIδ via O-GlcNAcylation, leading to mitochondrial DNA release. Targeting this pathway alleviates cardiac remodeling and identifies circulating mtDNA as a potential biomarker of fetal cardiac injury.

## Introduction

Maternal diabetes during pregnancy alters the intrauterine environment, negatively impacting fetal development and predisposing the offspring to diabetes and cardiovascular diseases^1^. Both early- and late-onset diabetes during pregnancy have been associated with serious adverse fetal outcomes, underscoring the importance of these critical developmental periods^2^. Numerous studies show that fetal exposure to hyperglycemia in *utero* increases the risk of cardiovascular complications^3–5^. In contrast to the adult heart, the fetal heart is undergoing continuous development and is more vulnerable to stress, which can have long-lasting effects into adulthood. Nevertheless, the precise mechanisms by which intrauterine hyperglycemia affects cardiac development in offspring remain poorly understood.

Maternal diabetes, both pre-existing and gestational, can significantly affect the development of the cardiovascular system during embryonic and fetal stages^6^. The effects include alterations in metabolite patterns, a pro-inflammatory environment, changes in circulating glucose levels, cardiac remodeling, and fibrosis^7–9^. Mitochondria are pivotal for various metabolic processes, including glucose^10^ and lipid metabolism^11,12^. In recent years, it has been discovered that mitochondrial dysfunction not only impairs adenosine triphosphate (ATP) synthesis and induces cell apoptosis but also causes the release of mitochondrial matrix materials, including N-formyl peptides and mtDNA, which triggers cellular inflammation and exacerbates cell damage^13^. Research has shown that intracellular calcium overload can lead to the release of mtDNA, which can further exacerbate myocardial injury^14–16^. Nevertheless, the mechanisms underlying pathophysiological changes in fetal hearts remain largely unknown.

The internal milieu of the fetus is crucial for proper development; however, intrauterine hyperglycemia alters this environment significantly. Currently, the most promising and druggable metabolic therapies for cardiac injury appear to involve the singular or combined targeting of glycolysis, *O*-linked β-N-acetylglucosamine modification (*O*-GlcNAcylation), which is the attachment of *O*-GlcNAc moieties to proteins, and the metabolism of ketones, fatty acids, and succinate^17,18^. Hyperglycemia has been reported to increase *O*-GlcNAcylation levels^19^, but its role in fetal cardiomyocytes remains unknown. In adult model animals and human patients, failing myocardium is characterized by heightened protein *O*-GlcNAcylation^20^. Under hyperglycemic conditions, *O*-GlcNAcylation is enhanced via the *O*-GlcNAc transferase (OGT) pathway. Specifically, in the heart, increased glucose availability drives excess glucose through the Hexosamine Biosynthetic Pathway (HBP), leading to an increase in uridine diphosphate N-acetylglucosamine (UDP-GlcNAc), which serves as a substrate for OGT. OGT then catalyzes the addition of *N*-acetylglucosamine to the hydroxyl groups of serine and threonine residues of proteins^21^. This persistent post-translational modification is detrimental to cardiac function^22^, as elevated *O*-GlcNAcylation promotes inflammation and fibrosis^23^ by influencing various pathways, including Yes-associated protein 1 (YAP1), nuclear factor kappa-light-chain-enhancer of activated B cells (NF-κB) signaling, calcium/calmodulin-dependent protein kinase II (CaMKII), oxidation stress, and *c*-FOS signaling^24–26^.

Diabetic hyperglycemia and oxidative stress are key factors contributing to harmful cardiac remodeling, where impaired intracellular Ca^2+^ handling and increased activity of CaMKII play significant roles. Besides the classical Ca^2+^/calmodulin-dependent activation that may cause CaMKII autophosphorylation at pT287, additional post-translational modifications (PTMs) such as oxidation and *O*-GlcNAc can also be heightened in diabetes. Oxidation at the Met281/282 pair^27^ and *O*-GlcNAcylation at S280^28^ have been identified in the regulatory hub region of CaMKII, both promoting autonomous and pathologically sustained kinase activity (molecular memory) that leads to cardiac remodeling, heart failure, and arrhythmias^29^. Current research and clinical therapies in diabetes focus on preventing the effects of hyperglycemia and oxidative stress, including those caused by PTMs. Mice with CaMKIIδ over-activation exhibited increased hypertrophy, decreased mitochondrial function, and increased fibrosis^30^. Hence, CaMKIIδ has been shown to serve as a trigger in inflammatory gene expression and subsequent cardiac damage^31^. However, the effect of *O*-GlcNAcylation of CaMKIIδ during gestational diabetes mellitus (GDM) and how CaMKIIδ activates neonatal inflammation have not been investigated.

In this study, we investigated the increase in *O*-GlcNAc of CaMKIIδ and its key effects on the hearts of offspring subjected to intrauterine hyperglycemia. A well-established model of streptozotocin (STZ)-induced hyperglycemia in maternal rats prior to pregnancy was employed to assess cardiac morphology and mitochondrial function in neonatal hearts. We found that pharmacological or genetic inhibition of either *O*-GlcNAcylation or CaMKIIδ improved cardiac function and mitochondrial function in hyperglycemic offspring by decreasing mtDNA release. In *utero* hyperglycemia affected neonatal cardiomyocytes through *O*-GlcNAcylation, resulting in mitochondrial permeability transition pore (mPTP) opening, mtDNA release, and subsequent inflammation in neonatal hearts. Our findings reveal a mechanism for hyperglycemia-induced neonatal cardiac remodeling and propose a potential therapeutic avenue for neonatal hearts exposed to intrauterine hyperglycemia.

## Results

### Maternal diabetes mellitus induced fetal cardiac dysfunction

**Fig. 1:**
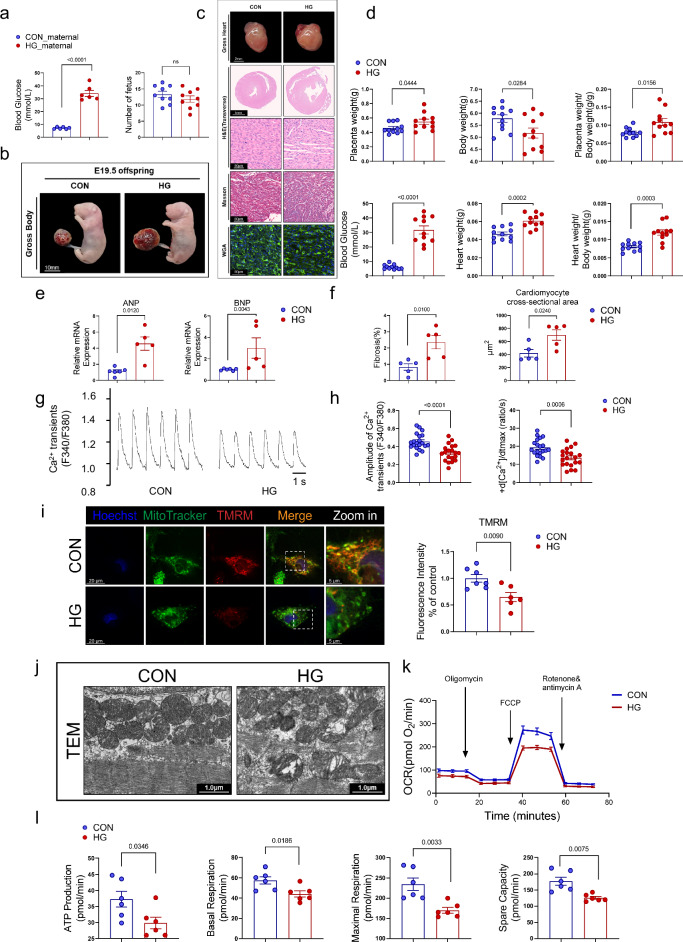
Maternal diabetes mellitus induced cardiac remodeling and mitochondrial dysfunction in offspring. Fetal rats at embryonic day 19.5 (E19.5) were used in Fig. 1 b-f and Fig. 1 j. NRCMs isolated from E19.5 fetal hearts were used in Fig. 1 g–i and k, l. **a** Representative the levels of maternal blood glucose (*n* = 6 per group) and the number of fetuses (*n* = 9 per group). **b** Representative gross morphology of fetuses on E19.5, scale bar = 10 mm. **c** Gross morphology and histological analysis of fetal heart tissues. Gross heart morphology, scale bar = 2.0 mm; HE, Masson and WGA staining, scale bar = 50 μm. **d** The placenta weight, body weight, placenta weight normalized to body weight, blood glucose level, heart weight, and heart weight normalized to body weight were compared between the CON and HG groups, *n* = 11 per group. **e** *ANP* and *BNP* gene expression was quantified by RT-qPCR, *n* = 6 (CON), 5 (HG) biologically independent samples. **f** Quantitative analysis of cardiomyocyte cross-sectional area (WGA staining) and fibrotic area (Masson staining), *n* = 5 per group. **g** Representative traces of Ca^2+^ transients between group CON and HG. **h** The amplitude and rate of rise of Ca^2+^ transients were quantified, *n* = 20 per group. **i** Fluorescence images of cardiomyocytes stained with MitoTracker (green), TMRM (red), and Hoechst (blue), scale bar = 20 μm. Quantification of TMRM fluorescence intensity in cardiomyocytes, *n* = 6 (HG) and 7 (CON) biologically independent samples. **j** Mitochondrial ultrastructure was shown using transmission electron microscopy (TEM), scale bar = 1.0 μm. **k** CON and HG cardiomyocytes were analyzed by Seahorse MitoStress Test for OCR, *n* = 6 per group. **l** Graphs showed ATP production, basal respiration, maximal respiration, and spare capacity between the CON and HG groups, *n* = 6 per group. Data were first assessed for normality and equal variance. For normally distributed data, differences were analyzed using two-tailed unpaired or paired Student’s *t*-test, with Welch’s correction when variances were unequal. For non-normally distributed data, the Mann–Whitney U test was used. Data were expressed as mean ± SEM.

GDM induces fetal growth restriction and abnormal fetal cardiac histomorphology^32,33^. Using a well-established model to induce GDM^4,34^, we observed significantly elevated maternal blood glucose levels in the hyperglycemia (HG) group, although the number of fetuses did not differ statistically compared to the control (CON) group (Fig. 1a). To investigate hyperglycemia-induced cardiac structural alterations, we performed histological staining and morphological analyses. Representative histological images showed myocardial fibrosis and enlarged cardiomyocytes in the hearts of maternal rats from the HG group (Supplementary Fig. 1a). Quantitative analysis confirmed a significant increase in cardiomyocyte size in the HG_maternal group compared to CON_maternal group (Supplementary Fig. 1b). Similarly, the extent of myocardial fibrosis was elevated in HG maternal hearts, as shown in the statistical analysis in Supplementary Fig. 1c. Molecular analysis revealed mitochondrial stress (increased plasma mtDNA copy number; Supplementary Fig. 1d) and activation of the STING-TBK1-IRF3 innate immune pathway (elevated phosphorylation of pathway components; Supplementary Fig. 1e, f), accompanied by upregulated transcription of inflammatory mediators (*Usp18*, *Isg15*, *Ifit1*, *Ifit3*, *Cxcl10*, *Ifi44*, and *Iigp1*; Supplementary Fig. 1g). These results confirm the successful establishment of the maternal diabetes model and demonstrate the impact of gestational maternal hyperglycemia on structural and molecular alterations in maternal hearts.

In offspring, hyperglycemic exposure caused fetal growth restriction with reduced body weight, increased placental weight, and elevated blood glucose levels (Fig. 1d). In addition, we observed an elevation in plasma insulin levels, which was evident at embryonic day 19.5 (E19.5) and persisted through postnatal day 7 (P7) (Supplementary Fig. 1h). Cardiac development was profoundly disrupted, evidenced by heart enlargement (Fig. 1b, c), cardiomyocyte hypertrophy (Fig. 1f), elevated heart-to-body weight ratio (Fig. 1d), and upregulated myocardial injury markers *ANP* and *BNP* (Fig. 1e). Critically, cell contraction impairment manifested as disrupted intracellular Ca²⁺ homeostasis (Fig. 1g), with reduced amplitude and decay rate of Ca²⁺ transients in fetal cardiomyocytes (Fig. 1h), confirming compromised contractile capacity. Collectively, these data establish a pathological cascade whereby maternal diabetes induces mitochondrial stress and inflammation-mediated cardiac damage in mothers, and programming fetal growth restriction, cardiac maldevelopment, and functional deficits in offspring.

### Maternal diabetes induced mitochondrial dysfunction in offspring

To further investigate the potential impact of hyperglycemia on mitochondrial function in neonatal hearts, cardiomyocytes from neonatal rats (NRCMs) in CON and HG groups were subjected to tetramethylrhodamine methyl ester (TMRM) and mitochondria-specific fluorescent dye (MitoTracker) staining. Results showed decreased mitochondrial membrane potential in HG group cardiomyocytes compared to CON group (Fig. 1i). Electron microscopy revealed increased mitochondrial swelling and damage in HG group cardiomyocytes. Specifically, hearts of HG offspring showed abnormal mitochondria with mitochondrial cristae disorganized and vacuoles appearing in some mitochondrial cavities, consistent with mitochondrial dysfunction (Fig. 1j). To understand the effects of in *utero* hyperglycemia on offspring mitochondrial oxygen consumption, mitochondrial function was analyzed by measuring oxygen consumption rate (OCR) using the Seahorse XF Cell Mito Stress Test Kit. Changes in OCR were recorded, and areas under the curve were calculated before and after each treatment with inhibitors of mitochondrial oxidative phosphorylation. The results of Seahorse Mito Stress Test confirmed that NRCMs from the HG group showed a significant reduction in OCR compared with the CON group (Fig. 1k). In particular, ATP rpoduction, basal respiration, maximal respiration, and spare capacity rates were reduced (Fig. 1l). mPTP opening was also observed in HG group NRCMs (Supplementary Fig. 2a, b), accompanied by increased mitochondrial Ca^2+^ levels (Supplementary Fig. 2c, d). Together, our findings support that in *utero* hyperglycemia causes mitochondrial dysfunction and abnormal structure in offspring NRCMs.

### HG enhanced *O*-GlcNAcylation of CaMKIIδ in HG fetal hearts

**Fig. 2:**
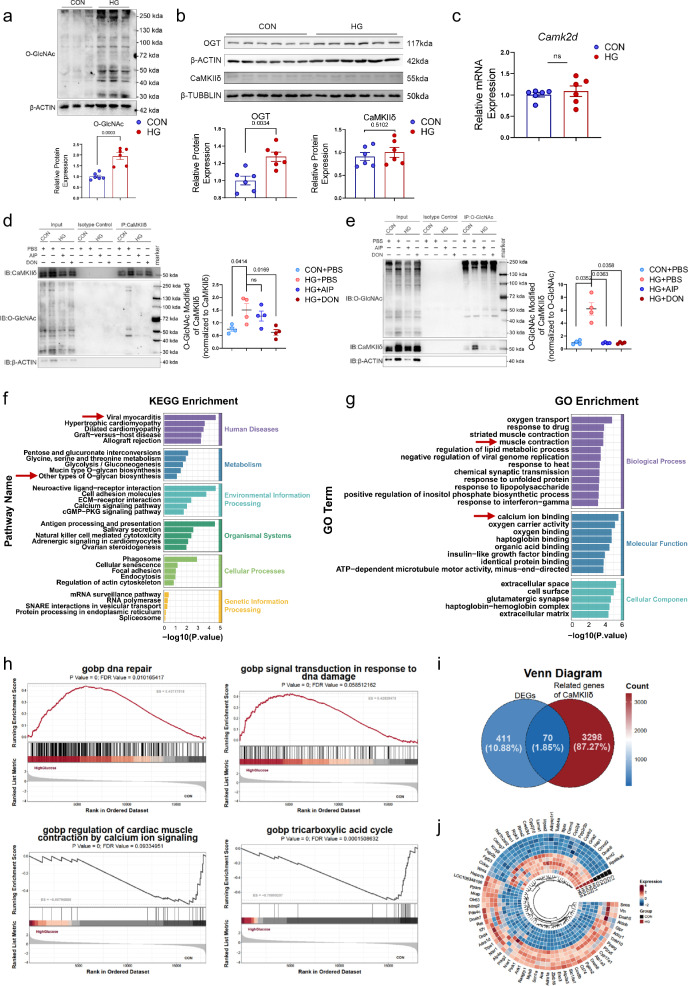
HG enhanced *O*-GlcNAcylation of CaMKIIδ in HG fetal hearts. Fetal rats at E19.5 were used for the experiments shown in Fig. 2 a–c and Fig. 2 f–j. CoIP experiments in Fig. 2 d, e were performed using NRCMs isolated from E19.5 neonatal hearts, DON was applied on NRCMs at 50 μM for 48 h, and AIP at 1 μM for 48 h. **a** Overall protein *O*-GlcNAcylation levels in fetal hearts were detected by western blotting. Quantification of the blotting, *n* = 6 per group. **b** Representative and quantitative western blotting analysis of OGT and CaMKIIδ in the fetal hearts, *n* = 6 per group. **c** *Camk2d* gene expression was quantified by RT-qPCR, *n* = 6 per group. **d** Western blotting of CoIP was performed. IP: CaMKIIδ. Quantification of the blots, *n* = 4 per group. **e** Western blotting of CoIP was performed. IP: O-GlcNAc. Quantification of the blots, *n* = 4 per group. **f**, **g** KEGG and GO enrichment analysis of DEGs (*q* < 0.05 and |Foldchange| > 2) between CON and HG groups in isolated cardiomyocytes of fetuses. Red arrows highlight key enriched pathways. **h** The diagram indicated the enriched pathways of DEGs, using GSEA of biological processes. **i** Venn diagram indicated the overlap of 70 genes between genes associated with CaMKIIδ in KEGG (red) and DEGs between CON and HG groups (blue). **j** Heatmap of the overlap of 70 genes. Data were first assessed for normality and equal variance. For normally distributed data, differences between two groups were analyzed using two-tailed unpaired or paired Student’s *t* test, with Welch’s correction when variances were unequal. Comparisons among three or more groups were performed using one-way ANOVA followed by Tukey’s post hoc test or Welch’s ANOVA when variances were unequal. For non-normally distributed data, the Mann–Whitney U test was used for two-group comparisons. The Kruskal–Wallis test followed by Dunn’s multiple comparisons test was used for comparisons among three or more groups. Data were expressed as mean ± SEM. IP, immunoprecipitation; IB, immunoblotting.

Hyperglycemia induces *O*-GlcNAc modifications and is involved in multiple organ injuries in humans and animals^28^. Furthermore, altered heart *O*-GlcNAcylation is associated with either the etiology or pathology of numerous adult cardiovascular diseases^28^. To gain further insight into the mechanisms underlying intrauterine hyperglycemia-induced myocardial damage in offspring, we investigated the role of *O*-GlcNAc in neonatal hearts. Firstly, we measured overall *O*-GlcNAc levels and OGT expression in CON and HG groups (Fig. 2a, b). Western blotting showed elevated total *O*-GlcNAc levels in HG fetal hearts compared to the CON group (Fig. 2a). Consistently, OGT protein expression was also increased in HG fetal hearts (Fig. 2b). In contrast, both the mRNA (Supplementary Fig. 3a) and protein levels of *O*-GlcNAcase (OGA) were reduced in the HG group (Supplementary Fig. 3b, c), indicating enhanced *O*-GlcNAcylation in hyperglycemic offspring. These findings indicate that protein *O*-GlcNAcylation is elevated in the offspring subjected to maternal hyperglycemia.

*O*-GlcNAc modifies various proteins and affects myocardial function, particularly through the regulation of ATP production and calcium homeostasis, processes in which mitochondria play a central role^35^. CaMKIIδ is a key regulator of Ca^2+^ signaling and mitochondrial function, and its hyperactivation has been shown to contribute to mitochondrial impairment and dysregulated calcium transients^28^. Previous studies have further demonstrated that excessive *O*-GlcNAc modification of CaMKIIδ at Ser280 activates CaMKIIδ and enhances CaMKIIδ-dependent Ca^2+^ release events and cardiac mechanical dysfunction^28,36^.

Based on the role of CaMKIIδ in cardiac Ca²⁺ signaling and mitochondrial function^28^, and given prior evidence that its *O*-GlcNAcylation at Ser280 can drive hyperactivation and cardiac dysfunction^28,36^, we next investigated its role in the context of in *utero* hyperglycemia. We first assessed CaMKIIδ expression and activity. While *Camk2d* mRNA expression and total CaMKIIδ protein levels were unchanged between CON and HG groups (Fig. 2b, c), direct assessment of CaMKII activity using the CaMKII activity sensor (CaMKAR-Adv) revealed enhanced CaMKII activation in HG NRCMs (Supplementary Fig. 2e, f). This activity was corroborated by an increase in phosphorylation of CaMKII at Thr287 in HG fetal hearts (Supplementary Fig. 3d), whereas oxidation of CaMKII at Met281/282 was unaltered (Supplementary Fig. 3e). These results indicated that hyperglycemia affected CaMKIIδ activity rather than its expression or oxidation. We then examined *O*-GlcNAcylation, a known activator of CaMKIIδ. Co-immunoprecipitation (CoIP) experiments demonstrated an increase in CaMKIIδ *O*-GlcNAcylation in the HG group, which was attenuated by the *O*-GlcNAcylation-reducing agent 6-diazo-5-oxo-L-norleucine (DON) (Fig. 2d). Reciprocal CoIP further validated this finding (Fig. 2e). To probe the functional relationship between *O*-GlcNAcylation and CaMKII activation, we applied the CaMKII inhibitor autocamtide-2-related inhibitory peptide (AIP). AIP treatment did not abolish the elevated *O*-GlcNAcylation of CaMKIIδ in the HG group (Fig. 2d), suggesting that *O*-GlcNAcylation acts upstream of, rather than as a consequence of, CaMKIIδ hyperactivation.

To further establish the role of *O*-GlcNAcylation, we induced cardiac-specific OGA overexpression in both CON and HG rats through intramyocardial injection of AdV-Oga-OE. This intervention suppressed the hyperglycemia-induced increase in CaMKIIδ *O*-GlcNAcylation (Supplementary Fig. 4a, b), and concurrently normalized CaMKIIδ activation in HG-treated NRCMs (Supplementary Fig. 4e, f). The efficiency of OGA overexpression was confirmed (Supplementary Fig. 4c, d). Together, these results indicate that in *utero* hyperglycemia enhances *O*-GlcNAcylation of CaMKIIδ, which in turn drives its hyperactivation, and that reducing this modification normalizes CaMKIIδ activity.

Building on these findings, we next investigated the role of CaMKIIδ in the hearts of HG offspring by conducting bulk transcriptome sequencing of hearts from CON and HG groups. Principal component analysis (PCA) and gene expression heatmaps revealed a pervasive alteration in gene expression patterns in hearts of HG fetuses (Supplementary Fig. 5a, b). The volcano plot illustrated a total of 481 differentially expressed genes (DEGs) between the CON and HG groups (Supplementary Fig. 5c), particularly enriched in the Kyoto Encyclopedia of Genes and Genomes (KEGG) pathways of Viral myocarditis pathway and Other types of *O*-glycan biosynthesis pathway (Fig. 2f). Gene ontology (GO) enrichment analysis highlighted significant enrichment in Calcium ion binding pathway and Muscle contraction pathway (Fig. 2g). Gene Set Enrichment Analysis (GSEA) based on KEGG and GO Biological Processes (BP) confirmed myocardial injury, DNA damage, abnormal Ca^2+^ homeostasis, mitochondrial dysfunction, and disturbance in mitochondrial metabolic pathways in HG fetal hearts (Fig. 2h and Supplementary Fig. 5d, e). Furthermore, CON and HG fetal hearts exhibited up to 70 DEGs associated with CaMKIIδ, which were mainly enriched in calcium- and mitochondrial-related pathways (Fig. 2i, j and Supplementary Fig. 5f). In conclusion, the data collectively indicate that intrauterine hyperglycemia-induced cardiac damage is characterized by substantial DNA and mitochondrial damage, the presence of *O*-GlcNAcylation, and CaMKIIδ activation-mediated Ca²⁺ dysregulation.

### Inhibition of *O*-GlcNAcylation of CaMKIIδ rescued HG-induced Ca^2+^-related cardiac contraction dysfunction

**Fig. 3:**
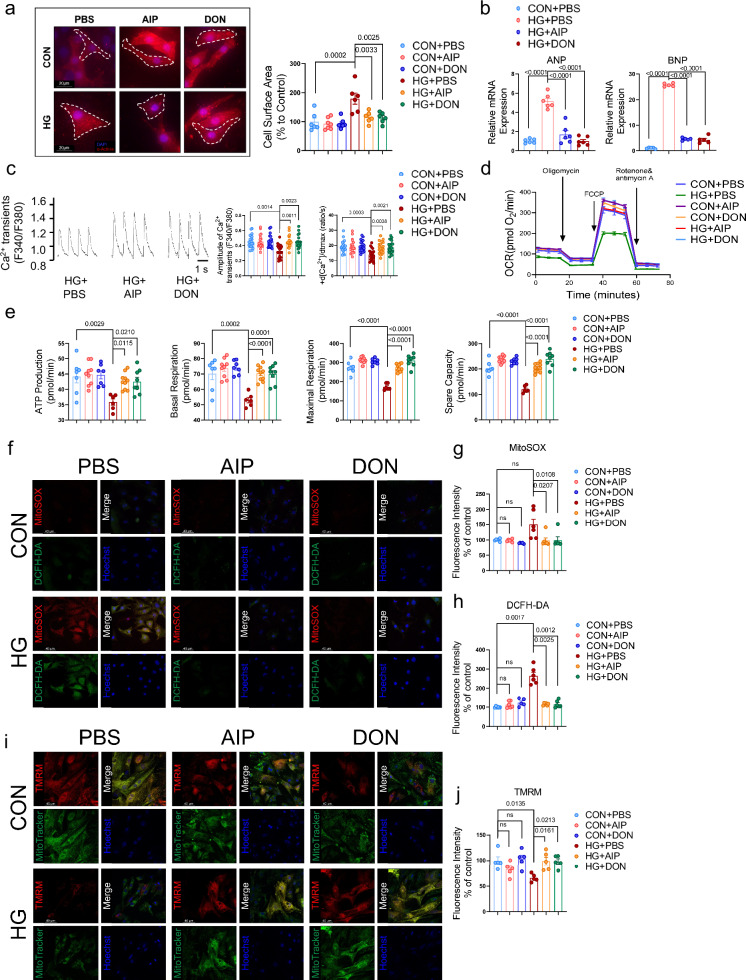
Inhibition of *O*-GlcNAcylation of CaMKIIδ rescued HG induced Ca^2+^-related cardiac contraction dysfunction. Experiments were performed using NRCMs isolated from E19.5 rat hearts. DON was applied on NRCMs at 50 μM for 48 h, and AIP at 1 μM for 48 h. **a** Representative images of cardiomyocytes double-labeled with DAPI (blue) and α-actinin (red). White dashed lines indicated the cell surface area of individual cardiomyocytes. Cell surface area was quantified, control values were considered as 100%. *n* = 6 (CON + PBS, HG + PBS, HG + AIP, HG + DON) and 7 (CON + AIP, CON + DON) biologically independent samples. **b** RT-qPCR was done to assess *ANP* (*n* = 6 per group) and *BNP* (*n* = 5 per group) gene expression. **c** Representative traces of Ca^2+^ transients among various groups. The amplitude and rate of rise of Ca^2+^ transients were quantified, *n* = 20 per group. **d** Seahorse Mito Stress Test for OCR was performed in various groups of cardiomyocytes. **e** Graphs showed ATP production, basal respiration, maximal respiration, and spare capacity in (**d**). *n* = 6 (HG + PBS), 7 (CON + PBS, CON + DON), 8(HG + DON), 9 (CON + AIP), and 10 (HG + AIP) biologically independent samples. **f** Fluorescence images of cardiomyocytes stained with DCFH-DA (green), MitoSOX (red), and Hoechst33342 (blue) for 20 min. Before staining, cardiomyocytes were pretreated with PBS or AIP or DON for 48 h, scale bar = 40 μm. **g** The statistically quantified data on the fluorescence intensity after staining with MitoSOX, *n* = 5 (CON + DON) and 6 (CON + PBS, CON + AIP, HG + PBS, HG + AIP, HG + DON) biologically independent samples. **h** The statistically quantified data on the cellular fluorescence intensity after staining with DCFH-DA, *n* = 5 (CON + DON) and 6 (CON + PBS, CON + AIP, HG + PBS, HG + AIP, HG + DON) biologically independent samples. **i** Fluorescence images of cardiomyocytes stained with TMRM (red), MitoTracker (green), and Hoechst33342 (blue), scale bar = 40 μm. **j** The statistically quantified data on the cellular fluorescence intensity after staining with TMRM, *n* = 5 per group. Data were first assessed for normality and equal variance. For normally distributed data, differences among three or more groups were analyzed using one-way ANOVA followed by Tukey’s post hoc test or Welch’s ANOVA when variances were unequal. For non-normally distributed data, the Kruskal–Wallis test followed by Dunn’s multiple comparisons test was used for comparisons among three or more groups. Data were expressed as mean ± SEM.

Based on the differences in *O*-glycan biosynthesis pathways (Other types of *O*-glycan biosynthesis pathways) and abnormal Ca²⁺ homeostasis (Calcium ion binding) observed in the RNA-seq data of CON and HG groups in Fig. 2f, g, as well as the differential expression of genes regulated by CaMKIIδ in Fig. 2i, we further investigated whether CaMKIIδ was involved in intrauterine hyperglycemia-induced morphology and function changes in fetal cardiomyocytes. Here, we evaluated the effects of CaMKIIδ via applying AIP and *O*-GlcNAcylation inhibitor DON. The effectiveness of DON was validated by reduced *O*-GlcNAcylation (Supplementary Fig. 6g, h), and that of AIP by decreased CaMKII activity (Supplementary Fig. 6i, j). Our results showed the increased cell surface area in the HG group, which were mitigated by AIP and DON treatment (Fig. 3a). Expression levels of *ANP* and *BNP* in HG cardiomyocytes were also reduced by AIP and DON (Fig. 3b). Both amplitude and rate of Ca^2+^ transients, decreased in HG + PBS group, were attenuated by AIP and DON (Fig. 3c). Seahorse MitoStress Test revealed OCR differences, showing increased ATP production, basal respiration, maximal respiration, and spare capacity in HG + AIP and HG + DON groups compared to HG + PBS alone (Fig. 3d, e). As shown by Mitochondrial superoxide indicators (MitoSOX) staining, mitochondrial reactive oxygen species (ROS) levels were decreased in the HG group after treatment with AIP and DON (Fig. 3f, g). 2’,7’-Dichlorodihydrofluorescein diacetate (DCFH-DA) staining demonstrated reduced ROS levels in whole cells from the HG group following AIP or DON treatment (Fig. 3f, h). TMRM staining also indicated that AIP and DON alleviated the mitochondrial membrane potential decrease in the HG group, without affecting the CON group (Fig. 3i, j). Overall, AIP and DON mitigate damage in neonatal cardiomyocytes, particularly in terms of Ca^2+^ transients and mitochondrial metabolism. This suggests that mitochondrial dysfunction and calcium-related abnormalities in myocardial contraction are critical contributors to cardiomyocyte injury in offspring of GDM mothers, which can be alleviated by inhibiting CaMKIIδ *O*-GlcNAcylation.

### *O*-GlcNAcylation mediated mtDNA release and upregulation of inflammatory genes in hyperglycemia

**Fig. 4:**
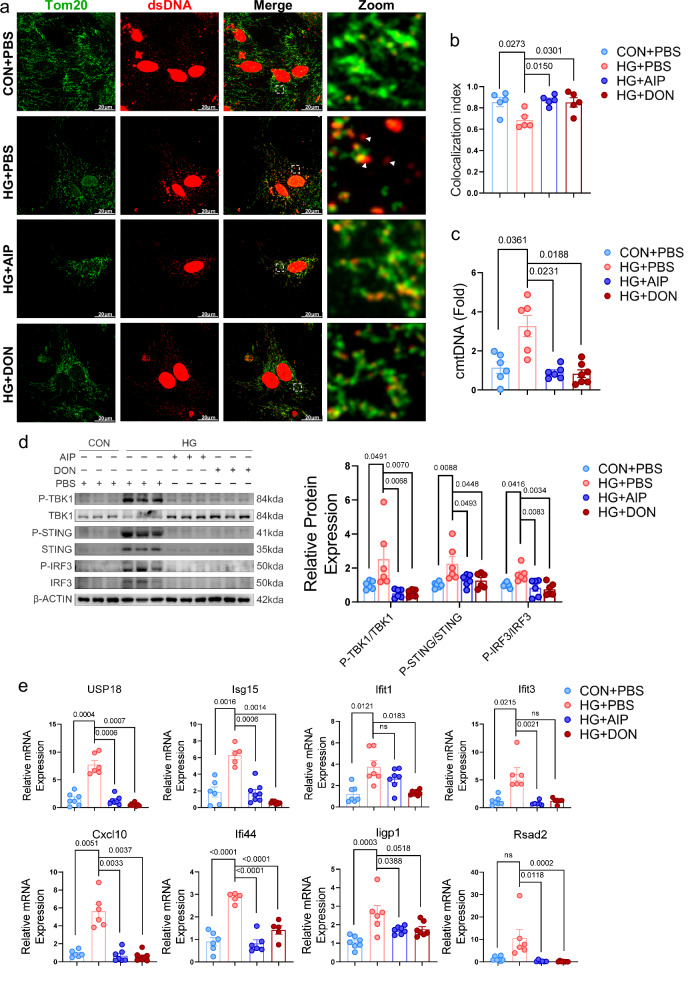
Inhibit CaMKIIδ or *O*-GlcNAcylation alleviated mtDNA release and the subsequent inflammation in NRCMs exposed to hyperglycemia. Experiments were performed using NRCMs isolated from E19.5 rat hearts. DON was applied on NRCMs at 50 μM for 48 h, and AIP at 1 μM for 48 h. **a** Cardiomyocytes were stained with Tom20 (green) and dsDNA (red) in all four groups. White arrows denoted mtDNA released from mitochondria, scale bar = 20 μm. **b** The colocalization index of dsDNA and mitochondria was quantified, *n*  =  5 per group. **c** Cytoplasmic mtDNA release as assessed by qPCR was shown, *n* = 6 (CON + PBS, HG + PBS, HG + AIP) and 7 (HG + DON) biologically independent samples. **d** Representative western blots and their analyses in cardiomyocytes from these groups, *n* = 6 per group. **e** *Usp18* mRNA expression was quantified by RT-qPCR, *n* = 6 (HG + PBS), 7 (CON + PBS, HG + AIP), and 8 (HG + DON). *Isg15* mRNA expression was quantified by RT-qPCR, *n* = 5 (HG + PBS), 6 (CON + PBS), and 8 (HG + AIP, HG + DON). *Ifit1* mRNA expression was quantified by RT-qPCR, *n* = 7 (CON + PBS, HG + PBS, HG + AIP) and 8 (HG + DON). *Ifit3* mRNA expression was quantified by RT-qPCR, *n* = 6 (HG + PBS, HG + AIP, HG + DON) and 7 (CON + PBS). *Cxcl10* mRNA expression was quantified by RT-qPCR, *n* = 6 (CON + PBS, HG + PBS), 7 (HG + AIP), and 8 (HG + DON). *Ifi44* mRNA expression was quantified by RT-qPCR, *n* = 5 (HG + PBS, HG + DON) and 6 (CON + PBS, HG + AIP). *Iigp1* mRNA expression was quantified by RT-qPCR, *n* = 6 (HG + PBS) and 7 (CON + PBS, HG + AIP, HG + DON). *Rsad2* mRNA expression was quantified by RT-qPCR, *n* = 6 (HG + PBS, HG + AIP) and 7 (CON + PBS, HG + DON). For RT-qPCR, *n* represented biologically independent samples. Data were first assessed for normality and equal variance. For normally distributed data, differences among three or more groups were analyzed using one-way ANOVA followed by Tukey’s post hoc test or Welch’s ANOVA when variances were unequal. For non-normally distributed data, the Kruskal–Wallis test followed by Dunn’s multiple comparisons test was used for comparisons among three or more groups. Data were expressed as mean ± SEM.

To further investigate the biological consequences and underlying mechanisms of *O*-GlcNAcylation, we focused on the evidence of altered mitochondrial function and the activation of DNA damage pathways revealed by RNA-seq analysis. Given that mitochondrial injury is known to activate the STING (stimulator of interferon genes) signaling pathway via mtDNA release, as reported by Kim et al., we hypothesized that this pathway might have been implicated in our observations^37^. Immunofluorescence staining of double-stranded DNA (dsDNA) and Tom20 (translocase of outer mitochondrial membrane 20) showed enhanced mtDNA release in NRCMs from the HG + PBS group, which was suppressed by AIP and DON treatment (Fig. 4a, b). Consistently, qPCR analysis showed that cytosolic mtDNA (cmtDNA) levels were elevated in the HG + PBS group and were reduced following AIP and DON treatment (Fig. 4c). The elevated cmtDNA levels observed in the hearts of HG group were a consequence of the release of dsDNA fragments from the mitochondrial matrix to the cytoplasm (Supplementary Fig. 7a). Although total mRNA and protein levels remained unchanged, phosphorylation of STING, TBK1 (TANK-binding kinase 1), and IRF3 (interferon regulatory factor 3) proteins increased in the HG group (Supplementary Fig. 7b, c). This phosphorylation was suppressed by AIP or DON treatment (Fig. 4d). In addition, mRNA expression levels of several inflammation-related genes were consistently elevated in the HG group (Supplementary Fig. 7d), a trend that was similarly mitigated by AIP or DON (Fig. 4e). In summary, AIP and DON suppressed hyperglycemia-induced mtDNA release and the upregulation of inflammatory genes. These findings suggest that *O*-GlcNAcylation and CaMKIIδ act as mediators of the inflammatory response in neonatal cardiomyocytes under hyperglycemic conditions.

### *O*-GlcNAcylation promotes mtDNA release and subsequent inflammatory response in hyperglycemia through CaMKIIδ regulating mPTP opening

**Fig. 5:**
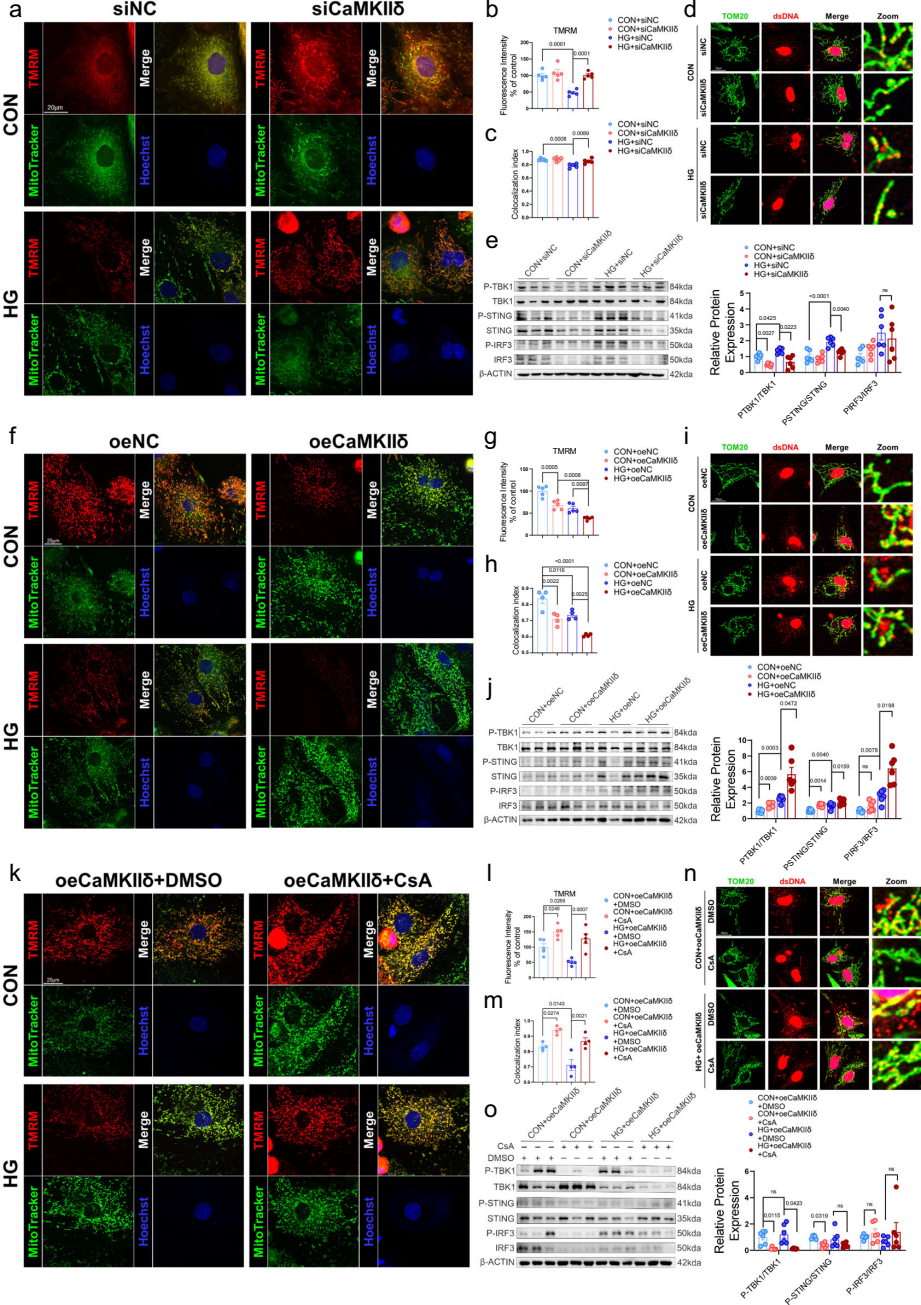
mPTP inhibitor CsA improved mitochondrial membrane potential and reduced mtDNA release. Experiments were performed using NRCMs isolated from E19.5 rat hearts. **a** Fluorescence images of cardiomyocytes stained with TMRM (red), MitoTracker (green), and Hoechst33342 (blue), scale bar = 20 μm. **b** Quantification of the cellular fluorescence intensity after staining with TMRM, *n* = 5 per group. **c** The colocalization index of dsDNA and mitochondria in (**d**) was quantified, *n* = 5 per group. **d** Cardiomyocytes were stained with Tom20 (green) and dsDNA (red), scale bar = 20 μm. **e** Phosphorylated and total protein levels in cardiomyocytes were detected by western blotting. Quantification of the blots, *n* = 6 per group. **f** Fluorescence images of cardiomyocytes stained with TMRM (red), MitoTracker (green), and Hoechst33342 (blue), scale bar = 20 μm. **g** The statistically quantified data on the cellular fluorescence intensity after staining with TMRM, *n* = 5 per group. **h** The colocalization index of dsDNA and mitochondria in (**i**) was quantified, *n* = 4 per group. **i** Cardiomyocytes were stained with Tom20 (green) and dsDNA (red), scale bar = 20 μm. **j** Phosphorylated and total protein levels in cardiomyocytes were detected by western blotting. Quantification of the blots, *n* = 6 per group. **k** Fluorescence images of cardiomyocytes stained with TMRM (red), MitoTracker (green), and Hoechst33342 (blue), scale bar = 20 μm. **l** Quantification of the cellular fluorescence intensity after staining with TMRM, *n* = 5 per group. **m** The colocalization index of dsDNA and mitochondria in (**n**) was quantified, *n* = 4 per group. **n** Cardiomyocytes were stained with Tom20 (green) and dsDNA (red), scale bar = 20 μm. **o** Phosphorylated and total protein levels in cardiomyocytes were detected by western blotting. Quantification of the blots, *n* = 6 per group. Data were first assessed for normality and equal variance. For normally distributed data, differences among three or more groups were analyzed using one-way ANOVA followed by Tukey’s post hoc test or Welch’s ANOVA when variances were unequal. For non-normally distributed data, the Kruskal–Wallis test followed by Dunn’s multiple comparisons test was used for comparisons among three or more groups. Data were expressed as mean ± SEM.

We next examined whether CaMKIIδ acted as a key regulator of mtDNA release and investigated the underlying mechanisms. Fluorescence staining of TMRM and MitoTracker in CON and HG group cardiomyocytes transfected with control siRNA or *Camk2d* siRNA revealed that *Camk2d* siRNA alleviated the decrease in mitochondrial membrane potential observed in the HG group (Fig. 5a, b). dsDNA and Tom20 staining further indicated increased mtDNA release into the cytoplasm of HG group cells transfected with control siRNA, which was reduced by *Camk2d* siRNA treatment (Fig. 5c, d). While total protein levels remained unchanged, phosphorylation of TBK1 and STING proteins decreased following *Camk2d* siRNA transfection in the HG group (Fig. 5e). Moreover, *Camk2d* overexpression exacerbated the reduction of mitochondrial membrane potential caused by hyperglycemia (Fig. 5f, g) and promoted hyperglycemia-induced mtDNA release in cardiomyocytes (Fig. 5h, i). This was accompanied by increased phosphorylation of STING, TBK1, and IRF3 proteins (Fig. 5j). It has been demonstrated that mitochondrial outer membrane permeabilization (MOMP) induced the mtDNA release^38^. The mPTP, a non-specific channel between the inner and outer mitochondrial membranes, can open under pathological conditions^39^. Given established links between CaMKII and mPTP opening^40^, we hypothesized that CaMKIIδ regulates mtDNA release *via* mPTP. Therefore, we investigated the correlation between CaMKIIδ, mPTP, and mtDNA release using Cyclosporin A (CsA), an established mPTP inhibitor^41^. Our data showed that CsA mitigated the decline in TMRM and mtDNA release associated with *Camk2d* overexpression in the HG group of NRCMs (Fig. 5k–n). Additionally, CsA reduced phosphorylation of TBK1 and STING proteins in *Camk2d*-overexpressing NRCMs (Fig. 5o).

**Fig. 6:**
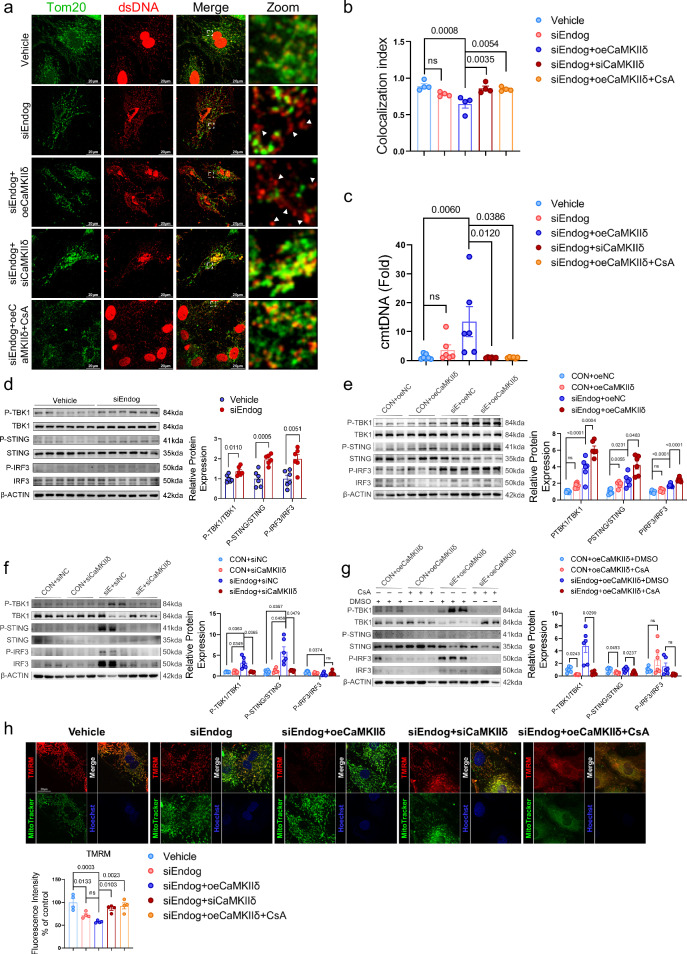
*O*-GlcNAcylation promoted mtDNA release and subsequent inflammatory response in hyperglycemia through CaMKIIδ regulating mPTP opening. Experiments were performed using NRCMs isolated from E19.5 rat hearts. CaMKIIδ siRNA or CaMKIIδ overexpression plasmid was transfected into NRCMs for 48 h. CsA was applied on NRCMs at 10 μM for the final 4 h. **a** Cardiomyocytes were stained with Tom20 (green) and dsDNA (red), scale bar = 20 μm. White arrows denoted mtDNA released from mitochondria. **b** The colocalization index of dsDNA and mitochondria in (**a**) was quantified, *n* = 4 per group. **c** Cytoplasmic mtDNA release was assessed by qPCR, *n* = 6 (siEndog, siEndog+oeCAMK2δ, siEndog+siCAMK2δ, siEndog + oeCAMK2δ + CsA) and 7 (Vehicle) biologically independent samples. **d**–**g** Representative immunoblots and analyses of phosphorylated and total protein levels in cardiomyocytes among multiple groups, *n* = 6 per group. **h** Representative fluorescence images of cardiomyocytes stained with TMRM (red), MitoTracker (green), and Hoechst33342 (blue), scale bar = 20 μm. The statistically quantified data on the fluorescence intensity, *n* = 4 per group. Data were first assessed for normality and equal variance. For normally distributed data, differences between two groups were analyzed using two-tailed unpaired or paired Student’s *t* test, with Welch’s correction when variances were unequal. Comparisons among three or more groups were performed using one-way ANOVA followed by Tukey’s post hoc test or Welch’s ANOVA when variances were unequal. For non-normally distributed data, the Mann–Whitney U test was used for two-group comparisons. The Kruskal–Wallis test followed by Dunn’s multiple comparisons test was used for comparisons among three or more groups. Data were expressed as mean ± SEM.

To obtain direct evidence of CaMKIIδ inducing mtDNA release, we manipulated endonuclease G (*Endog*) in NRCMs under normoglycemic conditions. *Endog* is a mitochondrial intermembrane space-located endonuclease that participates in apoptosis and inflammation by degrading DNA fragments^42^. *Endog*^−/−^ mouse embryonic fibroblasts (MEFs) have higher levels of cmtDNA relative to wild-type MEFs^37^. Hence, the role of CaMKIIδ in regulating mtDNA release was further validated by co-transfecting *Endog* siRNA and *Camk2d* siRNA. Knockdown efficiencies of *Camk2d* (Supplementary Fig. 6a–c) and knockdown efficiencies of *Endog* (Supplementary Fig. 6d–f) were validated by mRNA and protein expression levels. Co-transfecting *Endog* siRNA and *Camk2d* siRNA led to a reduction in mtDNA release compared to transfection with *Endog* siRNA alone (Fig. 6a). Meanwhile, co-transfecting *Endog* siRNA and a *Camk2d*-overexpressing plasmid in NRCMs resulted in an increase in mtDNA release from the mitochondrial matrix (Fig. 6a, b). qPCR results showed that cytosolic mtDNA levels were increased after co-transfection with *Endog* siRNA and a *Camk2d*-overexpressing plasmid (Fig. 6c), consistent with the enlarged images shown in Fig. 6a. *Endog* siRNA transfection in NRCMs led to increased phosphorylation of STING, TBK1, and IRF3 (Fig. 6d), recapitulating the activation of downstream signaling pathways observed in hyperglycemia. NRCMs co-transfected with *Endog* siRNA and *Camk2d* overexpression plasmid showed elevated phosphorylation levels of STING, TBK1, and IRF3 (Fig. 6e). In contrast, NRCMs with *Endog* siRNA and *Camk2d* siRNA exhibited decreased phosphorylation of TBK1 and STING (Fig. 6f). CsA treatment of NRCMs co-transfected with *Endog* siRNA and a *Camk2d*-overexpressing plasmid also reduced TBK1 and STING phosphorylation (Fig. 6g). These findings suggest that CaMKIIδ facilitates mtDNA release through mPTP opening, while CsA inhibits this effect. Fluorescence staining of TMRM and MitoTracker in NRCMs revealed that *Camk2d* overexpression exacerbated the decrease in mitochondrial membrane potential. However, inhibition of  mPTP opening with CsA restored mitochondrial membrane potential (Fig. 6h). In conclusion, CaMKIIδ regulates mtDNA release from the mitochondrial matrix to the cytosol *via* mPTP opening, both in the presence and absence of hyperglycemia, thereby activating the downstream STING signaling pathway. These results suggest that hyperglycemia-induced *O*-GlcNAcylation of CaMKIIδ leads to mtDNA release through mPTP, triggering activation of the STING pathway.

### Cardiac-specific reduction of *O*-GlcNAcylation ameliorated cardiac dysfunction in GDM offspring

To investigate the role of *O*-GlcNAcylation in vivo, AdV-Oga-OE or AdV-Vehicle was intramyocardially injected into neonatal rats from both CON and HG groups at postnatal day 3 (P3). Neonates were euthanized on P7 for analyses. AdV-Oga-OE-treated HG rats exhibited reductions in fibrosis and cardiomyocyte size (Supplementary Fig. 8a). Statistical analysis confirmed significant reductions in myocardial fibrosis and cardiomyocyte size in HG+AdV-Oga-OE rats compared to HG + AdV-Vehicle rats (Supplementary Fig. 8b, c). A reduction in cardiac injury markers *ANP* and *BNP* was observed in HG+AdV-Oga-OE rats at P7 (Supplementary Fig. 8d, e). These results demonstrate that AdV-Oga-OE ameliorated hyperglycemia-induced cardiac injury. Furthermore, plasma mtDNA release was decreased in the HG+AdV-Oga-OE rats (Supplementary Fig. 8f). Following AdV-Oga-OE treatment, phosphorylation levels of STING, TBK1, and IRF3 were also reduced in HG rats (Supplementary Fig. 8g), indicating that inhibition of *O*-GlcNAcylation suppressed STING pathway. Collectively, these findings support that cardiac-specific reduction of *O*-GlcNAcylation attenuates hyperglycemia-induced cardiac remodeling.

To further elucidate the role of CaMKIIδ *O*-GlcNAcylation following in *utero* hyperglycemia, we performed interventions in both CON and HG groups using adenoviral vectors, ADV-shCaMKIIδ and ADV-CaMKIIδ-S280A-OE. The ADV-shCaMKIIδ reduced the endogenous expression of CaMKIIδ, whereas the ADV-CaMKIIδ-S280A-OE abolished the *O*-GlcNAcylation site (S280) while preserving the remaining functions of CaMKIIδ. CoIP results demonstrated that simultaneous in vivo injection of ADV-shCaMKIIδ and ADV-CaMKIIδ-S280A-OE reduced CaMKIIδ *O*-GlcNAcylation in the hearts of HG rats (Supplementary Fig. 9a, b). Transfection with the CaMKAR-Adv demonstrated that ADV-shCaMKIIδ and ADV-CaMKIIδ-S280A-OE attenuated CaMKII activity in the HG group (Supplementary Fig. 9c, d). The efficiency of CaMKIIδ knockdown was confirmed (Supplementary Fig. 9e). These results demonstrate that genetic interventions reduce CaMKIIδ *O*-GlcNAcylation, leading to decreased CaMKIIδ activity under hyperglycemic conditions. More importantly, injection of ADV-shCaMKIIδ and ADV-CaMKIIδ-S280A-OE in HG hearts restored fibrosis levels and cardiomyocyte size (Supplementary Fig. 10a–c). Following genetic intervention, cardiac injury markers *ANP* and *BNP* were also reduced in the HG group (Supplementary Fig. 10d, e). These results demonstrate that specific reduction of CaMKIIδ *O*-GlcNAcylation ameliorates hyperglycemia-induced cardiac injury. Furthermore, plasma mtDNA release and the phosphorylation levels of STING, TBK1, and IRF3 were also reduced in the HG + ADV-shCaMKIIδ and ADV-CaMKIIδ-S280A-OE group (Supplementary Fig. 10f, g). These findings suggest that inactivation of the S280 site, thereby reducing CaMKIIδ *O*-GlcNAcylation, restored cardiac function in offspring subjected to intrauterine hyperglycemia.

### DON attenuated inflammatory damage induced by mtDNA release in neonatal rat hearts exposed to hyperglycemia

**Fig. 7:**
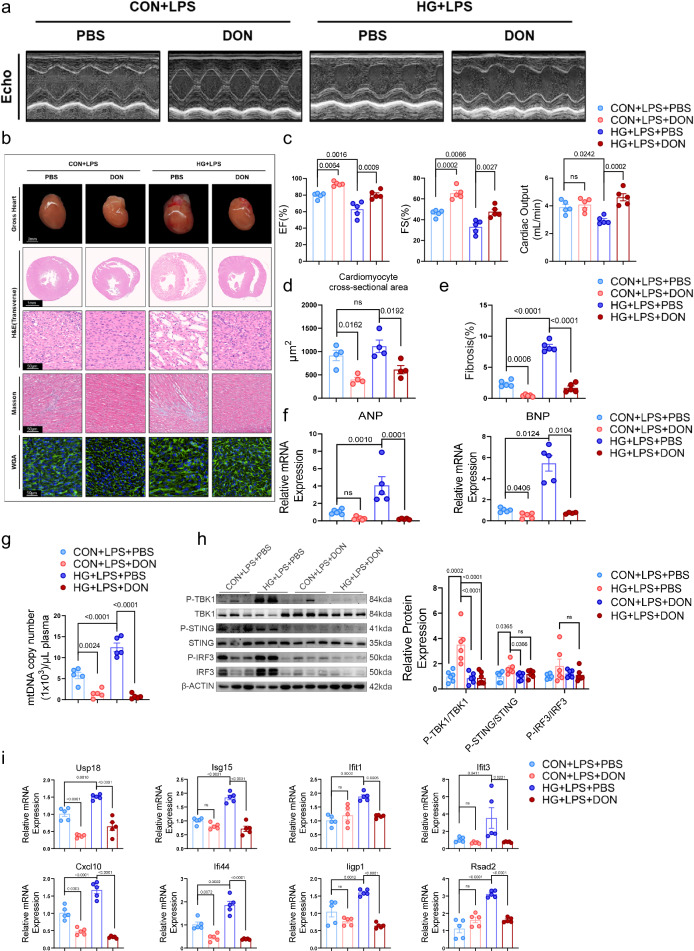
DON attenuated inflammatory damage induced by mtDNA release in hyperglycemic neonatal rat hearts. Neonatal rats received daily intraperitoneal injections of either PBS or DON (1 mg/kg/day) from postnatal day 3 to day 7, followed by a single LPS injection (2 mg/kg, i.p.) on day 7. All experiments in Fig. 7 were performed using heart tissues of P7 neonatal rats, except for the plasma mtDNA copy number experiment. **a**, **b** Echocardiography, gross morphology, and histological analyses were conducted for multiple groups of neonatal hearts. Gross morphology, scale bar = 2.0 mm; HE, Masson, and WGA staining, scale bar = 50 μm. **c** Echocardiographic parameters of EF%, FS%, and Cardiac Output were measured, *n* = 5 per group. **d**, **e** Quantitative analysis was performed on cardiomyocyte cross-sectional area (WGA staining, *n* = 4 per group) and fibrotic area (Masson staining, *n* = 5 per group). **f** *ANP* gene expression was quantified by RT-qPCR, *n* = 5 (HG + LPS + PBS, HG + LPS + DON) and 6 (CON + LPS + PBS, CON + LPS + DON) biologically independent samples. *BNP* gene expression was quantified by RT-qPCR, *n* = 4 (HG + LPS + DON) and 5 (CON + LPS + PBS, CON + LPS + DON, HG + LPS + PBS) biologically independent samples. **g** Plasma mtDNA copy number was assessed, *n* = 5 biologically independent samples. **h** Phosphorylated and total protein levels in hearts from multiple groups were detected by western blotting. Densitometric quantification of the blots, *n* = 6 per group. **i** RNA expression levels of *Usp18, Isg15, Ifit1, Ifit3, Cxcl10, Ifi44, Iigp1* and *Rsad2* were quantified by RT-qPCR, *n* = 5 per group. Data were first assessed for normality and equal variance. For normally distributed data, differences among three or more groups were performed using one-way ANOVA followed by Tukey’s post hoc test or Welch’s ANOVA when variances were unequal. For non-normally distributed data, the Kruskal–Wallis test followed by Dunn’s multiple comparisons test was used for comparisons among three or more groups. Data were expressed as mean ± SEM.

Notably, previous studies have demonstrated that neonates born to mothers with GDM exhibit a higher susceptibility to infections^43–46^. This increased vulnerability is thought to result from impaired immune competence due to metabolic disturbances, including hyperglycemia^47–49^. To model postnatal immune challenges^50,51^, we administered a single intraperitoneal injection of LPS (lipopolysaccharide, 2 mg/kg) to test whether intrauterine hyperglycemia exacerbated cardiac inflammatory responses to secondary stressors. This model enabled us to assess whether offspring subjected to intrauterine hyperglycemia exhibit an exaggerated myocardial response to inflammatory stimuli, thereby revealing the potential interaction between prenatal metabolic insults and postnatal immune stress. Offspring from CON and HG groups received daily intraperitoneal injections of either PBS or DON (1 mg/kg/day) from postnatal day 3 to day 7, followed by a single LPS injection (2 mg/kg, i.p.) on day 7 (Fig. 7). Control cohorts received only LPS or PBS (Supplementary Fig. 11).

Echocardiography showed reduced ejection fraction (EF%), fractional shortening (FS%), and cardiac output (CO) in the HG + PBS group compared with the CON + PBS group. Six hours after the LPS challenge, EF% and FS% were further decreased in the HG + LPS group compared with the CON + LPS group (Supplementary Fig. 11a, c). WGA staining demonstrated enlarged cardiomyocytes in LPS-treated hyperglycemic P7 hearts (Supplementary Fig. 11b, d). Masson staining demonstrated exacerbated fibrosis in LPS-treated hyperglycemic P7 hearts (Supplementary Fig. 11b, e). Hyperglycemia and LPS cotreatment increased *ANP* and *BNP* mRNA levels, with an additive effect observed when both factors were present (Supplementary Fig. 11f). Plasma mtDNA copy number was increased in the HG + LPS group compared to  CON + LPS group, indicating an additive effect of hyperglycemia and LPS on mtDNA release (Supplementary Fig. 11g). Western blotting analysis revealed enhanced phosphorylation of STING, TBK1, and IRF3 proteins in the HG + LPS group (Supplementary Fig. 11h). In line with these findings, mRNA expression of inflammatory genes, including *Usp18*, *Isg15*, *Ifit1*, *Ifit3*, *Cxcl10, Ifi44*, and *Iigp1*, was further upregulated in LPS-treated hyperglycemic offspring compared withCON + LPS offspring (Supplementary Fig. 11i). Collectively, these findings indicate that hyperglycemia sensitizes neonatal hearts to LPS-induced injury, leading to aggravated mitochondrial DNA release and activation of the STING signaling pathway.

**Fig. 8:**
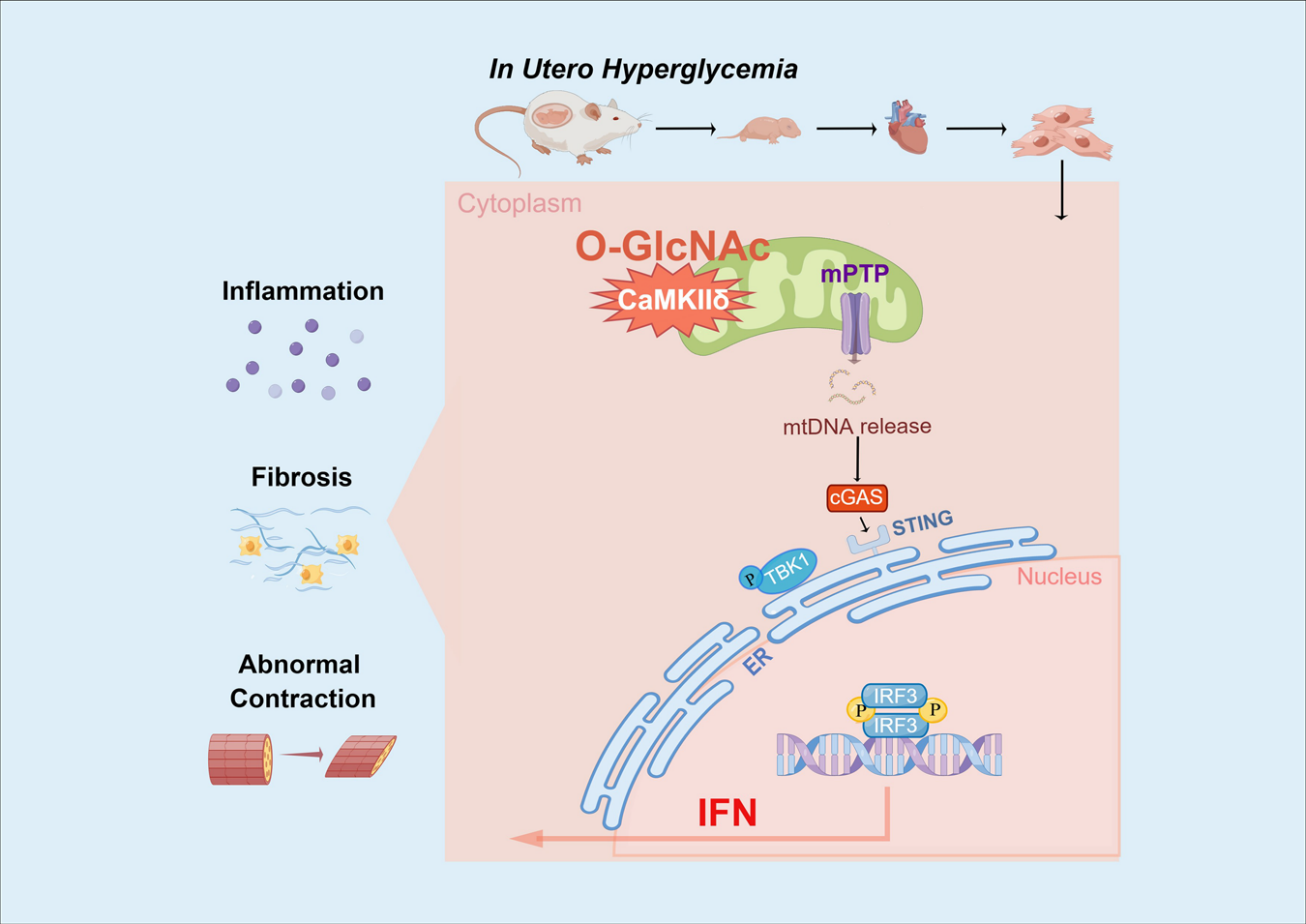
Schematic illustration of the CaMKIIδ/mtDNA/STING axis mediating cardiac remodeling in offspring under maternal hyperglycemia. Maternal hyperglycemia induces activation and post-translational modification of CaMKIIδ, leading to mitochondrial dysfunction and increased release of mtDNA into the cytosol. Cytosolic mtDNA activates the cGAS-STING signaling pathway, which subsequently triggers downstream inflammatory responses. These events collectively contribute to cardiomyocyte dysfunction and cardiac remodeling in offspring.

Crucially, echocardiography showed that DON intervention restored cardiac function of EF% and FS% in both the CON + LPS and HG + LPS groups (Fig. 7a, c). Concurrently, DON administration mitigated offspring cardiac remodeling. WGA staining revealed that DON attenuated cardiomyocyte enlargement in LPS-challenged hyperglycemic offspring (Fig. 7b, d), as well as Masson staining confirmed that DON reduced fibrosis in both CON + LPS and HG + LPS groups (Fig. 7e). Furthermore, DON suppressed pathological upregulation of myocardial injury marker *BNP* (Fig. 7f), regardless of whether LPS was administered under control or hyperglycemic conditions. Mechanistically, DON treatment lowered plasma mtDNA copy numbers elevated by hyperglycemia-LPS co-exposure (Fig. 7g), linking its cardioprotective effects to inhibition of mitochondrial DNA release. This was accompanied by attenuated phosphorylation of TBK1 protein (Fig. 7h) and downregulation of inflammatory gene expression levels (Fig. 7i), confirming disruption of the mtDNA-STING signaling cascade. Collectively, these results establish that targeting *O*-GlcNAcylation with DON preserves cardiac contractility and structure under hyperglycemic-inflammatory stress and suppresses mtDNA-driven innate immune activation via the STING-TBK1-IRF3 axis. Furthermore, it mitigates maladaptive remodeling in neonatal hearts, as evidenced by normalized cardiomyocyte morphology, reduced fibrosis, and diminished biomarker expression levels (Fig. 8). DON demonstrated equivalent therapeutic efficacy to that of AdV-Oga-OE. The therapeutic efficacy of DON thus stems from its dual modulation of mitochondrial integrity (reducing mtDNA release) and inflammatory signaling (inhibiting STING pathway phosphorylation). These findings highlight DON as a promising candidate for ameliorating diabetic cardiomyopathy exacerbated by secondary inflammatory challenges.

## Discussion

In the present work, we identify a previously unrecognized regulatory pathway involving the CaMKIIδ/mtDNA/STING axis in GDM-induced cardiac damage in offspring. The key findings are summarized as follows: We demonstrate that GDM induces abnormal cardiac and mitochondrial function in neonatal offspring. Mechanistically, GDM leads to increased *O*-GlcNAcylation of CaMKIIδ and triggers the release of mtDNA from the mitochondrial matrix to the cytosol. As a result, STING signaling is activated in neonatal cardiomyocytes, leading to the overexpression of interferon-stimulated genes such as *Usp18*, *Isg15, Ifit1, Ifit3, Cxcl10, Ifi44, Iigp1*, and *Rsad2*. *O*-GlcNAcylation of CaMKIIδ enhances its activity, contributing to abnormal inflammation in GDM offspring. Targeting the CaMKIIδ/mtDNA/STING axis attenuates the innate inflammation and cardiac dysfunction in GDM offspring.

Emerging evidence suggests associations between GDM and long-term health issues in offspring, including cardiac dysfunction such as adverse cardiometabolic profile, cardiac remodeling, and fibrosis^52–54^. Consistent with previous studies, our animal model of GDM offspring exhibited aberrant cardiac remodeling, and multiple cardiac dysfunctions including decreased cardiac contractility, reduced mitochondrial membrane potential, and increased inflammation (Fig. 1g–j and Supplementary Fig. 7). Inflammation has been implicated in the pathogenesis of cardiovascular remodeling^55–58^, however, the mechanisms responsible for initiating and integrating inflammatory responses within hearts remain unclear, especially in fetal hearts. Mitochondria are intimately associated with inflammation, acting as regulators beyond their established roles in cell survival and death^59–61^. Mitochondria and inflammasomes intersect at multiple facets and in different diseases^62–66^. In this study, we observed the occurrence of abnormal mtDNA fragment release from the mitochondrial matrix to the cytosol in fetal hearts. These escaped mtDNA in the cytosol provokes inflammation via cGAS-STING pathway activation, marking the instance of an early mitochondrial stress-triggered IFN-I (type I interferon) chronic immune response induced by GDM. Previous studies have demonstrated that mtDNA escaping autophagy can autonomously induce inflammatory responses in cardiomyocytes, leading to myocarditis and dilated cardiomyopathy^67^. Our findings confirm that the inhibition of mtDNA release can reduce the inflammatory response and myocardial remodeling. The findings of this study emphasize the significance of mtDNA release in cardiac inflammatory damage of GDM offspring.

Environmental alterations during critical developmental windows influence PTM modifications, which are essential determinants of the abnormalities induced by GDM^68^. The early embryonic period may represent a sensitive window predisposing individuals to cardiac dysfunction later in life. Intrauterine hyperglycemia acts to alter *O*-GlcNAc abundance and stress response pathways that are crucial for early development^69,70^. In our study, significant increases in OGT and *O*-GlcNAcylation are observed in the hearts of HG offspring, accompanied by fibrosis, cardiac inflammation, and increased cell size. While acute activation of *O*-GlcNAc levels has been demonstrated to protect cells from oxidative stress, increases in *O*-GlcNAcylation associated with hyperglycemia have been shown to have adverse effects on mitochondria. Importantly, a multitude of studies have demonstrated that excessive *O*-GlcNAc can impact mitochondrial function, including the activity of mitochondrial complexes, mitochondrial membrane potential, mitochondrial fragmentation, mitochondrial DNA repair, and mitochondrial quality control^71–75^. Interestingly, mtDNA-driven inflammation points to a crucial nexus between mitochondrial quality control and innate immune signaling pathways, as evidenced in our studies. Nevertheless, the mechanisms by which  *O*-GlcNAcylation responds to excessive glucose and triggers the mtDNA-mediated inflammation remains unclear. Further investigation into this process is warranted, given the crucial role of mitochondrial quality control in normal energy metabolism and disease development.

CaMKIIδ may induce cardiac dysfunction through several mechanisms. Its upregulation and PTM have been shown to be associated with cardiac pathology^76,77^. Our investigation of GDM offspring reveals that maternal hyperglycemia drives *O*-GlcNAc of CaMKIIδ, thereby inducing multiple stress responses in cardiomyocytes, including the oxidative stress response, the DNA damage response, the inflammatory response, and abnormal Ca^2+^ homeostasis. CaMKIIδ, as a key protein kinase, is involved in the regulation of Ca^2+^ homeostasis, whose activation has been linked to various heart diseases, including cardiac hypertrophy, heart failure, and arrhythmias^78^. CaMKII-S280 *O*-GlcNAcylation is required for increased arrhythmia susceptibility in diabetic hyperglycemia^36^. Once activated and glycosylated, CaMKIIδ can not only phosphorylate numerous ion channels and transporters, but also activate mitochondrial proteins^30^. The inflammatory response regulated by CaMKIIδ has been documented by multiple research groups. For example, Yao et al. identified CaMKIIδ9-IKK/IκB-NF-κB signaling as a regulator of human cardiomyocyte inflammation^79^. CaMKIIδ deletion also attenuates ischemia/reperfusion-induced inflammation and upregulation of NF-κB target genes^31^. Later on, subsequent work by Suetomi et al. demonstrates that CaMKIIδ induces an increase in proinflammatory chemokines and NLRP3 (NOD-like receptor thermal protein domain associated protein 3) inflammasome activation^80^. However, its impact on mitochondrial-mediated inflammation, especially on fetal heart development, has not been explored. Our study reveals a previously unrecognized mechanism of in *utero* hyperglycemic neonatal cardiomyocytes injury associated with CaMKIIδ signaling. We show that CaMKIIδ *O*-GlcNAcylation is specifically increased in neonatal hearts under maternal diabetic conditions. We  demonstrate that CaMKIIδ *O*-GlcNAcylation is specifically elevated in neonatal hearts exposed to maternal diabetes, whereas oxidized CaMKIIδ (Met281/282) remains unchanged. Mechanistically, hyperglycemia-induced *O*-GlcNAcylation occurs at the CaMKIIδ-S280 site, and genetic intervention with a CaMKIIδ-S280A mutant reduces this modification. Suppressing CaMKIIδ or its *O*-GlcNAcylation alleviates hyperglycemia-induced cardiomyocytes damage in vivo and in vitro. While CaMKIIδ activation has been linked to cardiac pathology, our S280A mutation experiment provides direct evidence that *O*-GlcNAcylation at this site, rather than oxidation, is the primary modifier in neonatal hyperglycemic injury. The S280A mutant abolishes the activation of CaMKIIδ and rescues mitochondrial membrane potential. This genetic precision excludes compensatory adaptations possible in pharmacological models, solidifying S280 *O*-GlcNAcylation as a therapeutic target. Furthermore, the cGAS-STING signaling is activated by CaMKIIδ-mediated mtDNA release, which promotes the expression of inflammatory cytokines through the IRF3 pathway and ultimately results in GDM neonatal cardiac dysfunction. It has been reported that Bak/Bax-dependent MOMP initiates the release of mtDNA and thus contributes to the cGAS-STING-mediated DNA-sensing pathway^38^. Moreover, although the inner mitochondrial membrane is a tight barrier, a transition in the inner mitochondrial membrane is triggered upon accumulation of Ca^2+^ in the mitochondrial matrix, which leads to release of mitochondrial proteins and DNA^39,81^. CaMKIIδ can promote excessive mitochondrial Ca^2+^ entry and give rise to Ca^2+^ overload^40^. Here, we demonstrate that hyperglycemia activates the *O*-GlcNAcylation level of CaMKIIδ, disrupts mitochondrial Ca^2+^ homeostasis, impairs mitochondrial oxidative respiration, and eventually promotes the opening of mPTP. Although the mPTP’ s molecular nature is poorly-defined^39,82^, Joiner et al. show the same view as ours that CaMKII promotes mPTP opening and myocardial death^40^. Our results further demonstrate that CaMKIIδ-mediated release of mtDNA from open mPTPs leads to subsequent chronic inflammation in neonatal hearts. Thus, our study fully links *O*-GlcNAc CaMKIIδ to mtDNA-induced cardiac inflammation in GDM offspring.

In conclusion, our findings indicate that *O*-GlcNAcylation induces mitochondrial dysfunction and impairs oxidative respiration through the activation of CaMKIIδ, leading to mtDNA release and upregulation of STING signaling and inflammatory genes. *O*-GlcNAcylation contributes to cardiac remodeling in GDM offspring by promoting cardiomyocyte inflammatory injury and fibrosis. These findings reveal mtDNA as a key mediator of cardiac *O*-GlcNAc-induced inflammation, and plasma mtDNA has the potential to serve as a serological marker for predicting the degree of myocardial inflammation in GDM mothers and neonates. Further investigation into the mechanisms of mtDNA release is required to develop more effective cardio-protective strategies. Our identification of the CaMKIIδ/mtDNA/STING axis provides two translatable insights: both pharmacological (DON) and genetic (OGA-OE, S280A) interventions can ameliorate cardiac dysfunction, suggesting that *O*-GlcNAc blockade is viable for neonatal therapy. Moreover, the efficacy of the S280A mutant, despite unchanged Met281/282 oxidation, reveals a distinct pathological role of *O*-GlcNAcylation, independent of oxidative stress cascades.

## Methods

Details of the methods are available in the Supplementary Information.

### Animal model

All procedures used have been approved by the Animal Care and Use Committee of Shanghai SINOGENE Biotechnology (Animal license number: XNG-201-2308-003) and follow the guidelines of the National Institutes of Health Guidelines on Care and Use of Laboratory Animals. Three-month-old female and male wild-type *Sprague-Dawley rats (Rattus norvegicus)* from the Experimental Animal Center of Soochow University were used. After a 1-week acclimation period, normal female rats were mated with male rats at a 1:1 ratio in the same experimental animal cages in the evening. On the following morning, the male and female rats were separated, and the presence of a vaginal plug in the female rats was checked. The day on which a vaginal plug was observed was designated as gestational day 0 (G0). Subsequently, the female rats were randomly assigned to either the CON group or the HG group. Rats in the HG group received intraperitoneal injections of STZ (50 mg/kg body weight, Sigma, USA) dissolved in 0.01 M citric acid buffer (pH 4.5) after overnight fasting. Rats in the CON group were injected with an equivalent volume of citric acid buffer. Both CON and HG groups received daily injections for three consecutive days^4,83^. Blood glucose levels were measured using a blood glucose meter (One Touch) 7 days after the injection to confirm that STZ-treated rats exhibited diabetes (non-fasting blood glucose > 16 mM). Rats were kept in a controlled environment at 22 °C with a 12/12 h light-dark cycle. Food and water were freely available^4,34^.

Sex was not considered a biological variable in this study because neonatal cardiac tissues were used, and sex hormone signaling is not fully established at this developmental stage.

### In vivo genetic and pharmacologic modulation

Genetic treatment: Neonatal rats were anesthetized on an ice bed for 2–3 min. An intercostal incision was performed on the left sternal chest to expose the heart by blunt dissection following skin incision. AdV (adenoviral vector) was evenly injected into three sites of the myocardium. After the operation, air bubbles were squeezed out of the thoracic cavity. Then, the thoracic wall and skin incision were sutured with 8–0 non-absorbable silk suture. The neonatal pups were then placed on a heater at 37 °C for several minutes until they recovered. Rats were placed back with their mothers when they woke up^84,85^.

Pharmacologic treatment: To evaluate the phenotypic response of offspring to postnatal infection, some offspring from the CON and HG groups received a single intraperitoneal injection of LPS^86–88^ (2 mg/kg; Sigma, USA) or an equal volume of PBS on postnatal day 7. Both male and female *Sprague-Dawley* rat pups were included. The injection site was disinfected with 75% ethanol before and after injection to minimize the risk of infection. Pups were maintained on a 37 °C heating pad during the procedure and recovery period for at least 30 min. All animals survived the procedure. Cardiac function was assessed by echocardiography 6 h after injection, followed by tissue collection^87–89^. To assess the protective effects of pharmacologic intervention, some offspring from both the CON and HG groups received daily intraperitoneal injections of DON (1 mg/kg/day; MCE, China) or PBS from postnatal day 3 to day 7^28,90–92^.

### Echocardiography

Cardiac function was determined by echocardiography at the designated time points using a digital ultrasound system (Vevo 2100 Imaging System, FUJIFILM-VISUALSONICS, JAPAN). Echocardiographic M-mode images were obtained from a parasternal short-axis view at the level of the papillary muscles. Contractile function parameters were automatically calculated after we traced the left ventricle's anterior and posterior walls using an electronic caliper in Visual Sonics Vevo imaging software. Three representative contraction cycles were selected for analysis in each rat. All echocardiography measurements were performed in a blinded manner^84^.

### Isolation and treatment of neonatal rat cardiomyocytes

The heart was excised with scissors, and the atrium, large blood vessels, and surrounding connective tissue were carefully removed. The ventricles were isolated, placed in pre-chilled Hank’s Balanced Salt solution (HBSS), rinsed several times to remove residual blood, and then cut into small pieces of approximately 1 mm^3^. The tissues were transferred to a 50 mL centrifuge tube containing 10 mL of 0.125% trypsin and incubated overnight at 4 °C. On the following day, the tissue fragments were transferred to DMEM medium containing 10% FBS and incubated in a 37 °C water bath with gentle shaking for 8–10 min. The supernatant was discarded, and the remaining tissue was subjected to enzymatic digestion with 0.08% collagenase II (Worthington, USA) for 3–5 cycles. An equal volume of DMEM containing 10% FBS was added to terminate the digestion reaction. All digestion fractions were collected into a 50 mL centrifuge tube and centrifuged at 1000 rpm for 10 min, and then the supernatant was discarded. Medium containing 70% DMEM, 19% M199, 10% FBS, 1% PS, and 0.1 mM bromodeoxyuridine to inhibit the growth of fibroblasts was added. Cells were seeded into cell culture dishes at an appropriate density and cultured in a 37 °C, 5% CO_2_ incubator. The medium was changed every 48 h for subsequent experiments.

Following cell attachment, the inhibitors were added directly to the culture medium and maintained. NRCMs were treated with the CaMKIIδ inhibitor AIP (1 μM; HY-P0214, MedChemExpress, China) or the *O*-GlcNAcylation inhibitor DON (50 μM; S8620, Selleck, USA) for 48 h prior to harvest^28,93,94^. CsA (10 μM; HY-B0579, MedChemExpress, China) was added to the culture medium for the final 4 h prior to sample collection^95^. DON was applied on NRCMs at 50 μM for 48 h, and AIP at 1 μM for 48 h. CsA was applied on NRCMs at 10 μM for the final 4 h.

For siRNA or plasmid transfection, transfection procedures were performed according to the manufacturer’s instructions. For siRNA transfection, RiboFECT™ CP Transfection Kit (R10035.5; RiboBio, China) was used. For plasmid transfection, Lipofectamine™ 3000 Reagent (L3000015; Thermo Fisher Scientific, USA) was used. CaMKIIδ siRNA or CaMKIIδ overexpression plasmid was transfected into NRCMs for 48 h.

### Quantification of mtDNA release

NRCMs (2 × 10^6^ cells) were resuspended in 170 μl of digitonin buffer containing 150 mM NaCl, 50 mM HEPES, pH 7.4, and 25 μg/ml digitonin. The homogenates were incubated on a rotator for 15 min at room temperature, followed by centrifugation at 16,000 × *g* for 25 min at 4 °C. The supernatant (cmtDNA) was used for qPCR. The pellet (total mtDNA) was resuspended in 340 μl of lysis buffer containing 5 mM EDTA and proteinase K and incubated at 55 °C overnight. The digested pellet was diluted with water and heated at 95 °C for 10 min to inactivate proteinase K, and the sample was used for qPCR with mtDNA-specific primers. The cmtDNA in the supernatant was normalized to the total mtDNA in the pellet^37^.

### Quantification of mtDNA Levels in cmt-DNA (Circulating cell-free mitochondrial DNA) from plasma

Plasma was collected in EDTA-coated blood collection tubes. The plasma samples were stored at −80 °C. The cmt-DNA was isolated using a Blood Genomic DNA Extraction Kit (DP304; TIANGEN BIOTECH) and used for qPCR. For the standard of mtDNA copy number, the D-LOOP cDNA clone was used. Concentrations were converted to copy number using the formula, mol/gram × molecules/mol = molecules/gram. The levels of mtDNA in all plasma analyses were expressed in copies per microliter of plasma.

### Fluorescent reporter for CaMKII activity

CaMKAR is a highly sensitive and recently developed FRET-based biosensor for monitoring CaMKII activity in living cells^96^. CaMKAR plasmid was a gift from Mark Anderson & Elizabeth Luczak (Addgene plasmid #205315; http://n2t.net/addgene:205315; RRID:Addgene_205315). We packaged the CaMKAR plasmid (Addgene #205315) into an adenoviral vector (CaMKAR-Adv) to enhance infection efficiency in NRCMs. To monitor CaMKII activity, NRCMs were infected with the CaMKAR-Adv for 24 h. Fluorescence signals were then observed and imaged under a Leica TCS SP8 Confocal Laser Scanning Platform (Leica SP8, Leica, USA).

### RNA sequencing and data analysis

Total RNA was isolated from rat heart samples using TRIzol reagent. The RNA integrity was assessed by Agilent 2100 with RIN number >7.0. A cDNA library constructed by technology from the pooled RNA from the 10 samples was sequenced on the Illumina HiSeqTM 2500 platform (OE Biotech, China). DEGs analysis was performed using the DESeq R package. *q* < 0.05 and foldchange >2 or foldchange <0.5 was set as the threshold for significantly differential expression. KEGG was conducted using the Bioconductor package clusterProfiler 4.0^97^ and visualized using the Bioconductor package ggplot2 (v3.3.3; Wickham, 2016). At the same time, GO, functional enrichment (biological processes, cellular component, and molecular functions), and pathway enrichment studies were also conducted. GSEA was carried out using clusterProfiler 4.0 for the functional annotation of the DEGs and visualized using the Bioconductor package enrichplot. |NES| > 1, *p* < 0.05, FDR < 0.25 was considered as a standard metric for quantifying the top listed pathways.

### Statistical analysis

All experiments included at least three biological replicates with comparable results. All statistical analyses were performed using GraphPad Prism 8 software. All error bars in the figure represent the mean ± SEM. For statistical comparisons, we initially evaluated data for normality and equality of variances. If the data were normally distributed, statistical differences between two groups were assessed using a two-tailed unpaired or paired Student *t* test. If equal variances were not assumed, Welch’s *t* test was applied. If the data were not normally distributed, nonparametric analyses were performed using the Mann-Whitney U test for two-group comparisons. For comparisons of three or more groups, we used one-way ANOVA (*p* < 0.05) followed by Tukey’s test for post-hoc analysis when the data were normally distributed. If equal variances were not assumed, Welch’s ANOVA was applied. If the data were not normally distributed, the Kruskal–Wallis test followed by Dunn’s multiple comparisons test was used. A *p* value < 0.05 was considered statistically significant.

### Reporting summary

Further information on research design is available in the Nature Portfolio Reporting Summary linked to this article.

## Supplementary information

Supplementary Information

Reporting Summary

Transparent Peer Review File

## Source data

Source Data file

## Data Availability

The bulk RNA-seq data have been deposited in the GEO database under accession code GSE298866 and are publicly available. The data generated in this study are provided in the Supplementary Information and Source Data file. Source data are provided with this paper.
