# Supplementary material for: Maternal hyperglycemia-induced O-GlcNAcylation of CaMKIIδ promotes mtDNA release and cardiac remodeling in offspring
Source: Nat Commun. 2026 Jul 29;17:7769. doi: 10.1038/s41467-026-75630-4 (PMC13439061; doi:10.1038/s41467-026-75630-4)
Supplement: Supplementary file 1 [file 41467_2026_75630_MOESM1_ESM.pdf]

SUPPLEMENTARY INFORMATION

Maternal Hyperglycemia-Induced *O*-GlcNAcylation of CaMKIIδ Promotes mtDNA Release and Cardiac Remodeling in Offspring

Zemeng Xiao[1, 9], Ling Gao[2, 9], Yuehong Wang[1, 9], Chunli Yang[3, 9], Yuxuan Qian[1,4], Lei Chen[1], Xuxia Li[1], Na Geng[1], Taiwei Chen[1], Ancai Yuan[1], Liuhua Hu[1], Phung N Thai[5,6], Hao Li[7], Ling Gao[7, 10], Zhice Xu[8, 10], Xiyuan Lu[1, 10], Jun Pu[1, 10]

[1]Department of Cardiology, Ren Ji Hospital, School of Medicine, State Key Laboratory of Systems Medicine for Cancer, Shanghai Cancer Institute, Shanghai Jiao Tong University, Shanghai, China.

[2]Institute of Reproduction and Development, Shanghai Key Laboratory of Reproduction and Development, Obstetrics and Gynecology Hospital, Fudan University, Shanghai, China.

[3]Institute for Fetology, First Hospital of Soochow University, Suzhou, China.

[4]State Key Laboratory of Systems Medicine for Cancer, Department of Urology, Ren Ji Hospital, Shanghai Cancer Institute, Shanghai Jiao Tong University School of Medicine, Shanghai, China.

[5]Department of Internal Medicine, University of California, Davis, CA, USA.

[6]Department of Veterans Affairs, Northern California Health Care System, Mather, CA, USA.

[7]Institute for Regenerative Medicine, State Key Laboratory of Cardiology and Medical Innovation Center, Shanghai East Hospital, School of Medicine, Tongji University, Shanghai, China.

[8]The First Affiliated Hospital of Soochow University, Suzhou, China.

[9]These authors contributed equally: Zemeng Xiao, Ling Gao, Yuehong Wang and Chunli Yang.

[10]These authors jointly supervised this work:

Jun Pu, M.D., Ph.D., FACC, FESC, Department of Cardiology, Ren Ji Hospital, School of Medicine, State Key Laboratory of Systems Medicine for Cancer, Shanghai Cancer Institute, Shanghai Jiao Tong University, Shanghai, China. E-mail: pujun310@hotmail.com

Xiyuan Lu, Ph.D., Department of Cardiology, Ren Ji Hospital, School of Medicine, State Key Laboratory of Systems Medicine for Cancer, Shanghai Cancer Institute, Shanghai Jiao Tong University, Shanghai, China. E-mail: luxiyuan@sjtu.edu.cn

Zhice Xu, Ph.D., The First Affiliated Hospital of Soochow University, Suzhou, China. E-mail: xuzhice@suda.edu.cn

Ling Gao, Ph.D., Institute for Regenerative Medicine, State Key Laboratory of Cardiology and Medical Innovation Center, Shanghai East Hospital, School of Medicine, Tongji University, Shanghai, China. E-mail: gaoling@tongji.edu.cn

EXPANDED METHODS

Morphological Analyses

In order to determine whether the mother's persistent hyperglycemia altered the neonatal heart morphology, we assessed the neonatal rat hearts. They were washed 3 times with pre-chilled PBS. Connective tissue was cut off. Hearts were then fixed in 4% paraformaldehyde (PFA, Beyotime, China). Tissues were dehydrated, embedded, and sectioned for hematoxylin and eosin staining. Images were taken by Nanozoomer S360 (Hamamatsu, Japan) slide scanner and visualized using the NDP.view.2.9.29 software. The average cross-sectional area of cardiomyocytes was analyzed, and at least 3 samples were analyzed in each group.

Immunofluorescence

Isolated cells were seeded in a glass-bottom dish, fixed with 4% PFA for 20 min at room temperature, permeabilized with 0.5% Triton X-100 (Beyotime, China) for 20 min, and then blocked with PBS containing 10% goat serum 30 min. We added different primary antibodies in PBS containing 5% goat serum and incubated overnight at 4°C. The following day, we incubated the cells with Alexa fluorescent secondary antibody (Beyotime, China) for 45 min, DAPI stained, and mounted. The processed samples were imaged with a Leica TCS SP8 Confocal Laser Scanning Platform (Leica SP8, Leica, USA) and analyzed with Leica Application Suite (LAS) X version 3.7.1.21655 imaging software. The following primary antibodies were used: anti-α-actinin antibody (Sigma, A7732), Rabbit polyclonal antibody against TOM20 (Proteintech, 11802-1-AP), Mouse monoclonal antibody against dsDNA (Abcam, ab27156). Experiments were performed using independently prepared

neonatal rat cardiomyocytes (NRCMs) cultures as biological replicates. The colocalization index of dsDNA and mitochondria (TOM20) was quantified based on Manders' coefficient index.

Co-immunoprecipitation (CoIP)

CoIP was performed using the Pierce™ Crosslink Magnetic IP/CoIP Kit (88805; Thermo Scientific, USA) following the manufacturer's protocol. Briefly, protein A/G magnetic beads were prewashed with Coupling Buffer and incubated with 5-10 μg of primary antibody for 15 minutes at room temperature. After three washes with Coupling Buffer, the antibody was crosslinked to the beads using disuccinimidyl suberate (DSS) for 30 minutes. The crosslinked beads were then washed three times with Elution Buffer and twice with IP Lysis/Wash Buffer. Cell lysates were prepared in IP Lysis/Wash Buffer and incubated with the prepared antibody-crosslinked beads overnight at 4 °C. After incubation, the beads were washed twice with IP Lysis/Wash Buffer and once with purified water. Bound protein complexes were eluted using the provided Elution Buffer and subjected to SDS-PAGE and western blotting analysis.

Western Blotting

Heart tissues were lysed in RIPA buffer (containing protease inhibitors) and homogenized with a Tissue Homogenizer (SCIENTZ, China) at 60 Hz. The lysates were then incubated on ice for 30 min, followed by centrifugation at 12,000 × g for 15 min at 4°C. Protein concentrations in the supernatant were determined using a BCA kit (Beyotime, China). Equal amounts of proteins were separated by 10% or 12.5% SDS-polyacrylamide gel electrophoresis, transferred to a polyvinylidene fluoride membrane (Immobilon-P;

Millipore), and blocked with 5% bovine serum albumin (w/v) for 1 hour. Membranes were incubated with primary antibodies overnight at 4°C, followed by incubation with secondary antibodies (Beyotime, China) for 1 h at room temperature. Protein bands were visualized using ECL and recorded with a ChemiDoc Imaging System (Bio-Rad, USA). Image J was used to quantify protein bands.

The following primary antibodies were used: *O*-linked N-acetylglucosamine (*O*-GlcNAc) Monoclonal Antibody (RL2) (Thermo Fisher Scientific, MA1-072), CaMKII delta Polyclonal Antibody (Thermo Fisher Scientific, PA5-22168), anti-*O*-GlcNAc monoclonal antibody CTD110.6 (Cell Signaling Technology, America, 9875S), rabbit polyclonal antibody against OGT (Beyotime, AF7651), rabbit polyclonal antibody against CaMKIIδ (Proteintech, 20667-1-AP), mouse monoclonal antibody against Beta Tubulin (Proteintech, 66240-1-Ig), rabbit monoclonal antibody against beta-Actin (ABclonal, AC038), mouse monoclonal antibody against beta-Actin (Proteintech, 66009-1-Ig), rabbit monoclonal antibody against TBK1 (Cell Signaling Technology, 3504S), rabbit monoclonal antibody against Phospho-TBK1 (Cell Signaling Technology, 5483S), rabbit monoclonal antibody against STING (Cell Signaling Technology, 13647S), rabbit monoclonal antibody against Phospho-STING (Cell Signaling Technology, 72971S), mouse monoclonal antibody against IRF-3 (Santa Cruz Biotechnology, sc-33641), rabbit monoclonal antibody against Phospho-IRF-3 (Cell Signaling Technology, 29047S), OGA/*O*-GlcNAcase Polyclonal Antibody (Thermo Fisher Scientific, 61426), Phospho-CaMKII beta/gamma/delta (Thr287) Polyclonal Antibody (Thermo Fisher Scientific, PA5-37833), Anti-oxidized-CaM Kinase II (Met281/282) Antibody (Sigma, 07-1387), ENDOG Polyclonal antibody (Proteintech,

22148-1-AP).

RNA isolation and RT-PCR

Total RNA was isolated using TRIzol reagent (Invitrogen, USA) according to the manufacturer's instructions. RNA was reverse transcribed into cDNA using HiScript III RT SuperMix (R323-01; Vazyme, China). Quantitative PCR was performed on the LightCycler® 480 System (Roche, Germany) using ChamQ Universal SYBR qPCR Master Mix (Q711-03; Vazyme, China). Relative mRNA expression levels were calculated using the $2^{-\Delta\Delta Ct}$ method and normalized to Actin. The primers used in this study were as follows:

ANP forward: 5'- GAGGAGAAGATGCCGGTAG-3'

ANP reverse: 5'-CTAGAGAGGGAGCTAAGTG-3 '

BNP forward: 5'-TGATTCTGCTCCTGCTTTTC-3 '

BNP reverse: 5'-GTGGATTGTTCTGGAGACTG-3'

Actin forward: 5'CCTAAGGCCAACCGTGAAAAG-3'

Actin reverse: 5'GCTCGAAGTCTAGGGCAACATAG-3'

Usp18 forward: 5'TGCTGTGATTGCCCCATGTT-3'

Usp18 reverse: 5'GGCAGTTTCCCTCCAACGAT-3'

TBK1 forward: 5'ACGAGATTGGGCGGAATGAA-3'

TBK1 reverse: 5'TCTAGCGGCACCAAAGTCTG-3'

Isg15 forward: 5'GAAGGCCCATGGAGGACAAA-3'

Isg15 reverse: 5'CATTGGCTCTGGATAGGGGC-3'

Ifit1 forward: 5'TCACCTTCCTCTGGCTACCT-3'

Ifit1 reverse: 5'GGCCTGATGTGCCAATTCCT-3'

Ifit3 forward: 5' GCCTCAAAGCCCTGAAGGAT-3'

Ifit3 reverse: 5' AATCGTGTTGCCTCCCACAT-3'

Cxcl10 forward: 5' AAGCGGTGAGCCAAAGAAGGTCAA-3'

Cxcl10 reverse: 5' AGCCGCACACTGGGTAAAGGGA-3'

Ifi44 forward: 5' CTGTGAAGGTCAAGGTATCAGA-3'

Ifi44 reverse: 5' ACTCCAGTTTCTGCACCACC-3'

Iigp1 forward: 5' GCGACAATCTTTGAAGCAGAAGA-3'

Iigp1 reverse: 5' GGAAGGTCAGTGAAGGGATGATG-3'

Rsad2 forward: 5' CGTTGGAGTTTGGGGGTCTA-3'

Rsad2 reverse: 5' TGGGAGGAGTCAACCTCTGAT-3'

IRF3 forward: 5' GGAGTTAGGCCGGTTCAGTC-3'

IRF3 reverse: 5' TAGTTGAGACTCGCAGCCAC-3'

D-loop forward: 5' GAAATCAACAACCCGCCCAC -3'

D-loop reverse: 5' GGGGAACGTATGGACGATGA -3'

Camk2d forward: 5' TGGCATAGTTCACAGGGACC -3'

Camk2d reverse: 5' TGCCAGCAAAACCAAACCAC -3'

Oga forward: 5' GCAGTAGCTGCTTAGGGACT -3'

Oga reverse: 5' GCCAAGAGACTAAGCGGAGG -3'

Endog forward: 5' ATGCCTGGAACAACCTTGAGAA -3'

Endog reverse: 5' CATAGGAACGCAGCTCGATTTG-3'

Ca^{2+} Imaging and Measurement of Ca^{2+} transients

Isolated cardiomyocytes were seeded in glass-bottom dishes. Once the cells had reached an appropriate density and started beating spontaneously, they were loaded with the Ca^{2+} fluorescent indicator Fura-2 AM (5 μM) for 10 min at room temperature. The cells were then washed three times with PBS at 7 min intervals. The cells were continuously perfused with Tyrode's solution using a perfusion system, and the solution temperature was maintained at 37 °C with a microscope thermostat. Cell Ca^{2+} transients were imaged and recorded using a Ca^{2+} recording system (IonOptix, MA, USA). The amplitudes of Ca^{2+} transients (F340/F380) were analyzed using IonWizard imaging software (version 7.5).

Transmission Electron Microscopy

Fresh myocardial tissue was cut into small cubes (approximately 1 mm^3) and fixed with glutaraldehyde, then dehydrated with a gradient acetone solution. The cubes were embedded and cut into ultra-thin sections and stained with uranyl acetate and lead staining solution at room temperature. Subsequently, the samples were dried using filter paper and observed using a transmission electron microscope (TEM).

Mitochondrial Histology

Isolated cardiomyocytes were seeded in glass-bottom dishes and stained with 1 μM MitoTracker Green FM (Invitrogen, USA) for 20 min to observe mitochondrial localization. Cells were imaged with a Leica TCS SP8 Confocal Laser Scanning Platform (Leica SP8, Leica, USA) and analyzed with Leica Application Suite (LAS) X version 3.7.1.21655 imaging software.

Assessment of mPTP Opening

The opening of the mPTP was assessed using the mPTP Assay Kit (Beyotime, C2009S)

according to the manufacturer's instructions. NRCMs were stained with Beyotime's mPTP Assay Kit. Fluorescence signals were observed and imaged under a Leica TCS SP8 Confocal Laser Scanning Platform (Leica SP8, Leica, USA). Calcein AM is a cell-permeable dye that is hydrolyzed by intracellular esterases to produce calcein, a green-fluorescent compound. $CoCl_2$ quenches cytosolic calcein fluorescence by binding to calcein molecules in the cytoplasm. Ionomycin triggers mPTP opening, resulting in mitochondrial calcein fluorescence loss, and is commonly used as a positive control.

Mitochondrial Ca^{2+} Measurement

Mitochondrial Ca^{2+} levels were assessed using the Mitochondrial Calcium Assay Kit with Rhod-2 AM (Beyotime, S1062S) according to the manufacturer's instructions.

Mitochondrial Membrane Potential and ROS Production

Mitochondrial membrane potential ($\Delta\Psi m$) was measured by Tetramethylrhodamine methyl ester (TMRM; Thermo Fisher Scientific, USA). The cells were incubated in a medium containing 100 nM TMRM for 20 min. Cells were imaged with a Leica TCS SP8 Confocal Laser Scanning Platform (Leica SP8, Leica, USA), and the intensity of mitochondrial fluorescence was measured with ImageJ Software (version 2.0.0). ROS was measured by MitoSOX (Thermo Fisher Scientific, USA) or DCFH-DA (Beyotime, China). Cells were incubated in a medium containing 5 μM MitoSOX or DCFH-DA for 20 min, and the fluorescence intensity was measured by the same method as above.

Seahorse XF Cell Mito Stress Test

The Agilent Seahorse XF Cell Mito Stress Test measures key parameters of mitochondrial function by directly assessing the oxygen consumption rate (OCR) of cells on the Seahorse

XFe and XF Extracellular Flux Analyzers. The assay utilizes the built-in injection ports on XF sensor cartridges to add modulators of respiration into cells during the assay to reveal the key parameters of mitochondrial function. The modulators included in this assay kit are Oligomycin, Carbonyl cyanide-4 (trifluoromethoxy) phenylhydrazone (FCCP), Rotenone (Rot), and Antimycin (AA). Specifically, we began by plating cells at a previously optimized density and then hydrated a sensor cartridge in Seahorse XF Calibrant at 37°C in a non-CO_2 incubator overnight. Following the manufacturer's instructions, we prepared compound stock solutions and working solutions. We added 1.5 μM Oligomycin, 0.5 μM FCCP, and 0.5 μM Rot/AA into the designated ports on the sensor cartridge. After running the Assay, we conducted data analysis.

Graphical illustrations and schematic preparation

Schematic figures, including Figure 8, were generated using Figdraw.

SUPPLEMENTARY FIGURES

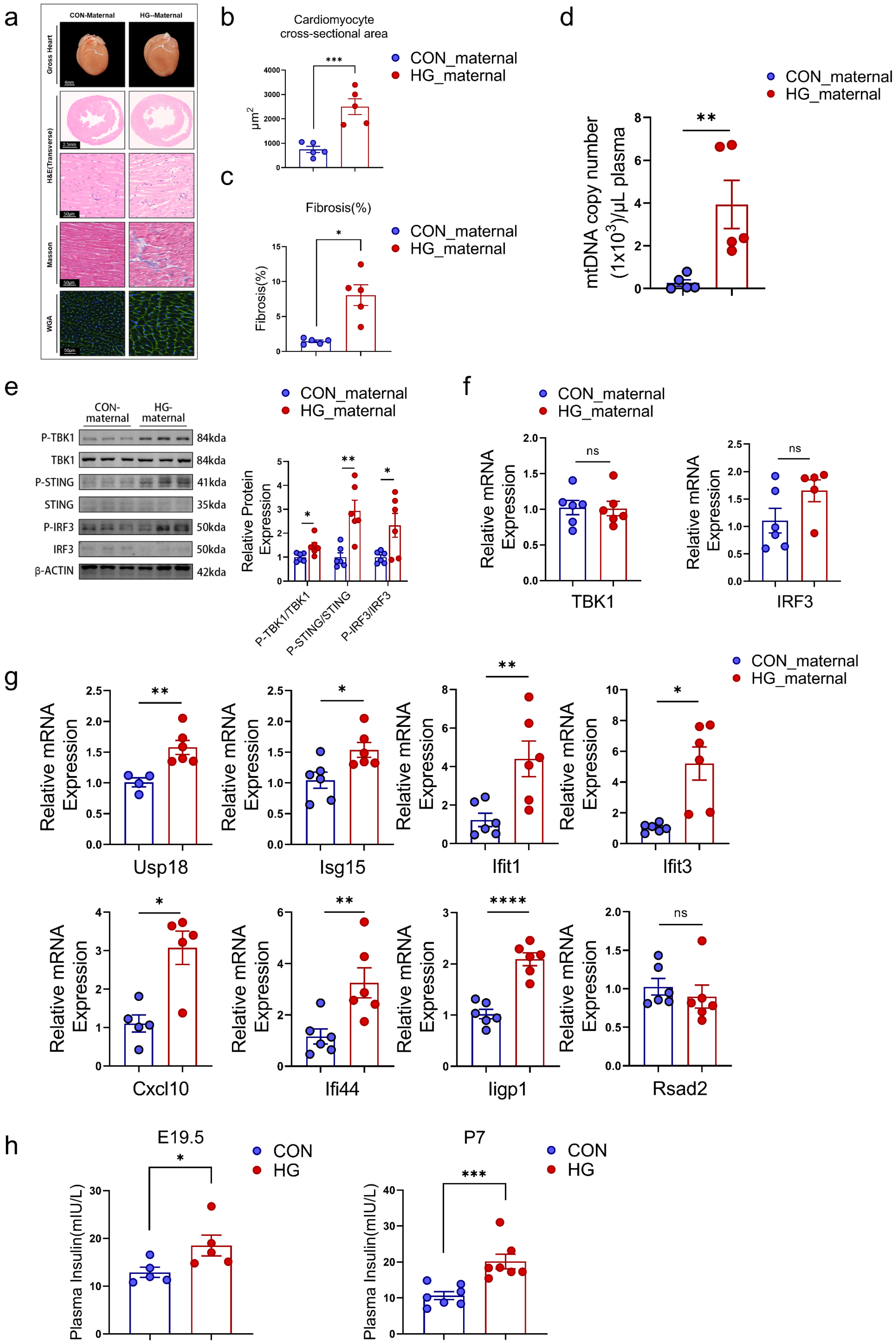

Supplementary Fig. 1. **Plasma mtDNA release level and inflammatory genes were up-regulated in hyperglycemic maternal rats.** All experiments were performed using maternal hearts at Embryonic day 19.5 (E19.5, late gestation stage), except for the plasma mtDNA copy number experiment. Experiments in Supplementary Fig. 1h were performed using E19.5 and P7 rat plasma. (a) Gross morphology and histological analysis of maternal heart tissues. Gross morphology, scale bar = 4.0 mm; HE, Masson and WGA staining, scale bar = 50 μm. (b-c) Quantitative analysis of cardiomyocyte cross-sectional area (WGA staining) and fibrotic area (Masson staining), n = 5 biological samples per group. (d) Quantification plasma mtDNA release levels in maternal CON and HG groups using qPCR, n = 5 per group. (e) Western blotting of phosphorylated and total protein levels in maternal CON and HG groups, n = 6 per group. (f) mRNA expression levels of *Tbk1* were performed by RT-qPCR, n = 6. mRNA expression levels of *Irf3* were performed by RT-qPCR, n = 5 (HG_maternal) and 6 (CON_maternal). (g) mRNA expression levels of *Usp18* were quantified by RT-qPCR, n = 4 (CON_maternal) and 6 (HG_maternal). mRNA expression levels of *Cxcl10* were quantified by RT-qPCR, n = 5. mRNA expression levels of *Isg15, Ifit1, Ifit3, Ifi44, Iigp1 and Rsad2* were quantified by RT-qPCR, n = 6. For RT-qPCR, n represented biologically independent samples. (h) Plasma insulin levels of E19.5 rats, n= 5 per group. Plasma insulin levels of P7 rats, n= 7 per group. Data were first assessed for normality and equal variance. For normally distributed data, differences were analyzed using two-tailed unpaired or paired Student's *t* test, with Welch's correction when variances were unequal. For non-normally distributed data, the Mann-Whitney U test was used. Data were expressed as mean±SEM, **p*<0.05, ***p*<0.01, ****p*<0.001, *****p*<0.0001.

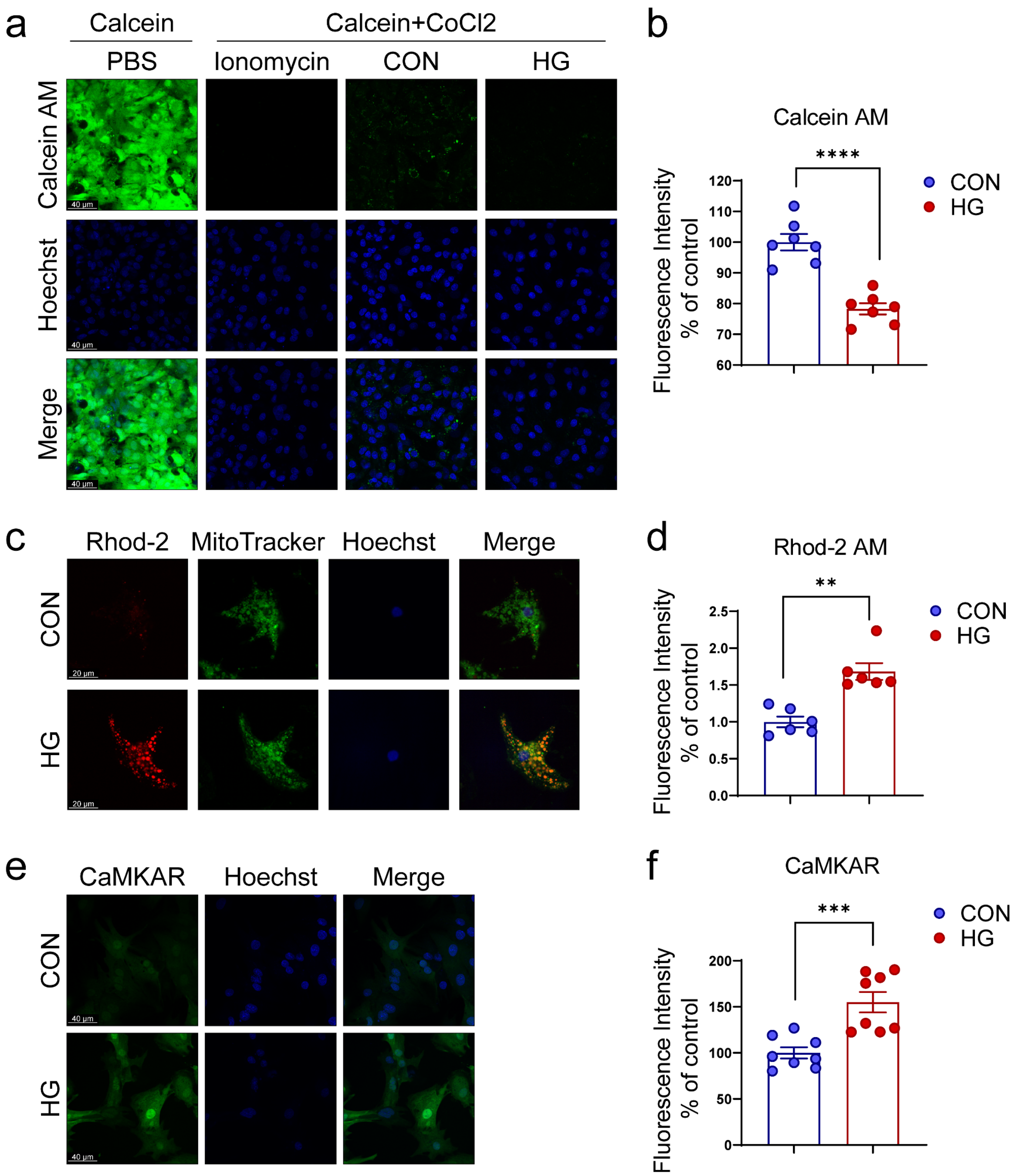

Supplementary Fig. 2. **Assessment of mPTP Opening, Mitochondrial Calcium, and CaMKIIδ Activity in NRCMs.** Fluorescence experiments were conducted using NRCMs isolated from E19.5 neonatal hearts. (a) Representative fluorescence images of NRCMs stained with Calcein AM (green) and Hoechst 33342 (blue) to evaluate mPTP opening, scale bar = 40 μm. (b) Quantification of Calcein AM (green) fluorescence intensity, n = 7 per

group. (c) Representative fluorescence images of NRCMs stained with Rhod-2 AM (red), MitoTracker (green), and Hoechst 33342 (blue) to assess mitochondrial Ca^{2+} levels, scale bar = 20 μm. (d) Quantification of Rhod-2 AM (red) fluorescence intensity, n = 6 per group. (e) Fluorescence images of cardiomyocytes infected with CaMKAR for 24 hours and stained with Hoechst 33342 for 20 min, scale bar = 40 μm. (f) The statistically quantified data on the cellular fluorescence intensity after infected with CaMKAR, n = 8 per group. Data were first assessed for normality and equal variance. For normally distributed data, differences were analyzed using two-tailed unpaired or paired Student's *t* test, with Welch's correction when variances were unequal. For non-normally distributed data, the Mann-Whitney U test was used. Data were expressed as mean±SEM, *$p<0.05$, **$p<0.01$, ***$p<0.001$, ****$p<0.0001$ vs. CON.

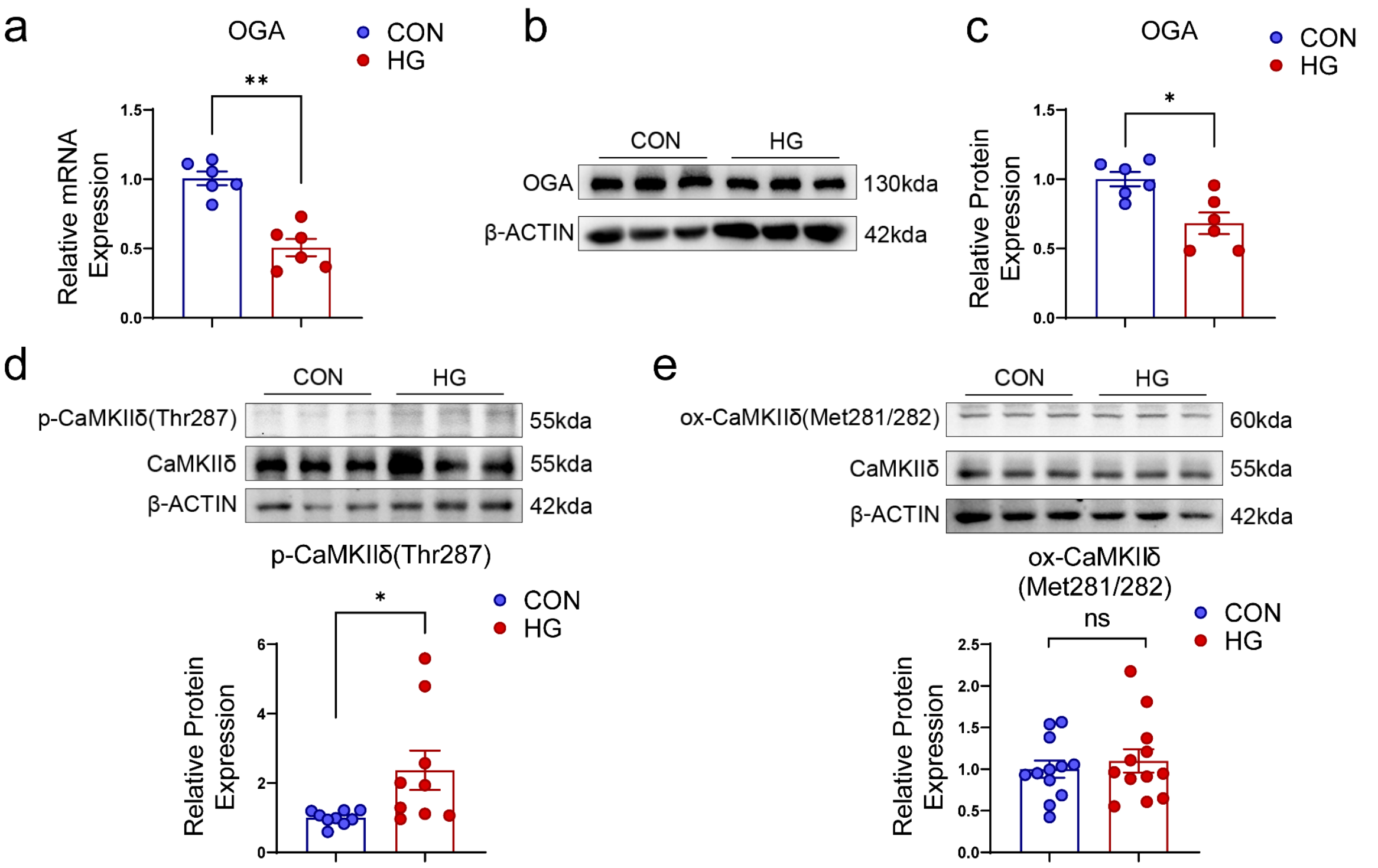

Supplementary Fig. 3. **Analysis of OGA expression, post-translational modifications of CaMKIIδ in hyperglycemia offspring.** Experiments were performed using E19.5 rat hearts in Supplementary Fig. 3a-e. (a) mRNA expression level of OGA in the CON and HG groups, n= 6 per group. (b) Representative immunoblots showing OGA protein expression level in the CON and HG groups. (c) Quantification of OGA protein expression level, n = 6 per group. (d) Representative immunoblots showing CaMKIIδ autophosphorylation in the CON and HG groups. Quantification of CaMKIIδ autophosphorylation levels, n = 9 per group. (e) Representative immunoblots showing CaMKIIδ oxidation in the CON and HG groups. Quantification of CaMKIIδ oxidation levels, n = 12 per group. Data were first assessed for normality and equal variance. For normally distributed data, differences were analyzed using two-tailed unpaired or paired Student's *t* test, with Welch's correction when variances were unequal. For non-normally distributed data, the Mann-Whitney U test was used. Data were expressed as mean±SEM, *p<0.05, **p<0.01, ***p<0.001, ****p<0.0001.

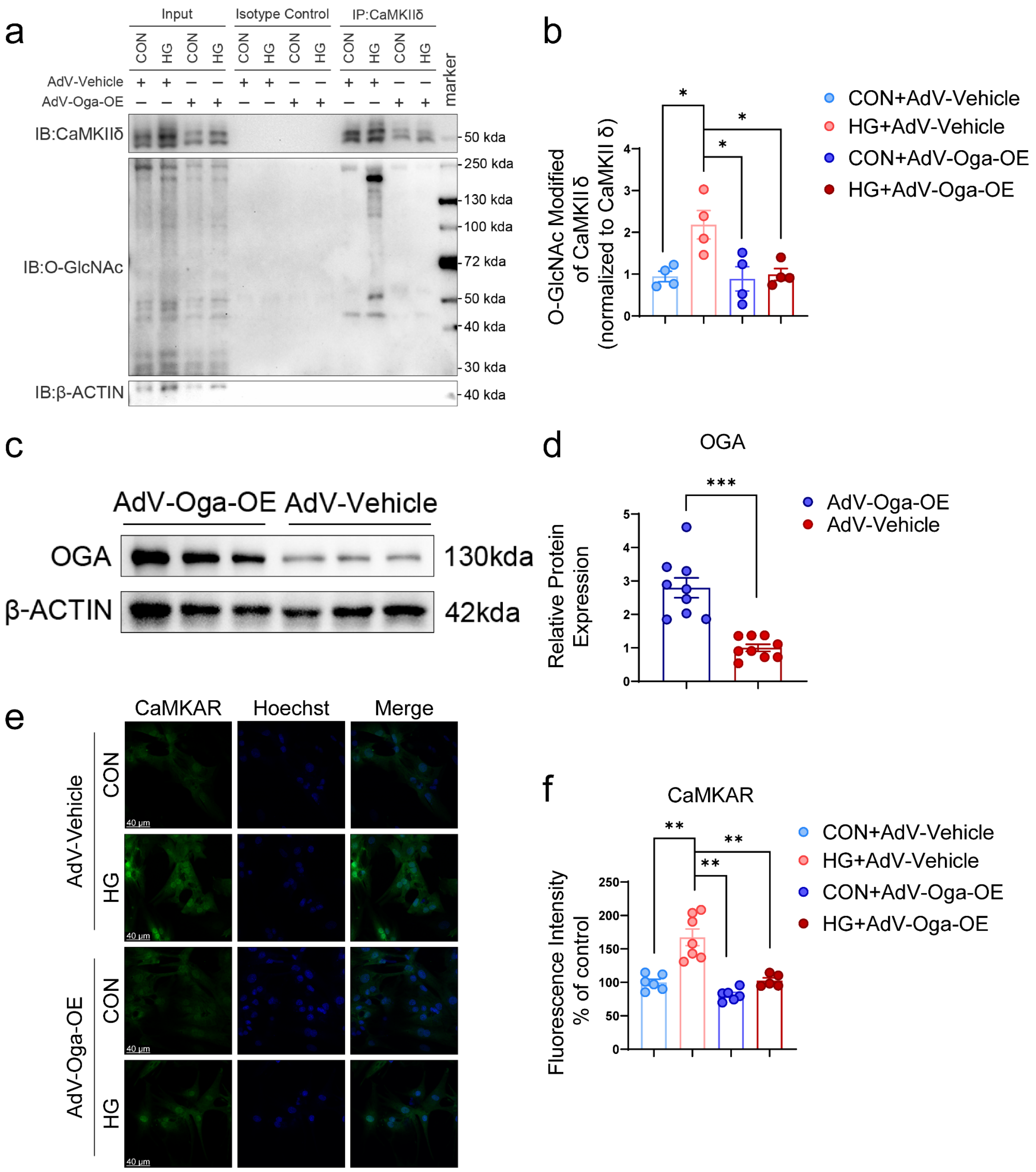

Supplementary Fig. 4. **Overexpression of OGA reduced cardiac CaMKIIδ *O*-GlcNAcylation and inhibited CaMKIIδ activation.** Neonatal rats received injections on postnatal day 3 and were euthanized on postnatal day 7. CoIP and western blotting experiments were performed using heart tissues from P7 neonatal rats. CaMKAR fluorescence imaging experiments were conducted using NRCMs isolated from P7 neonatal hearts. (a) Representative western blotting analysis of CoIP were measured for CON+AdV-

Vehicle, HG+AdV-Vehicle, CON+ AdV-Oga-OE, HG+AdV-Oga-OE groups, IP: CaMKIIδ. (b) The statistically quantified data on the western blotting analysis of *O*-GlcNAc modified CaMKIIδ, n = 4 per group. (c) Representative immunoblots of OGA protein expression in the following groups: AdV-Oga-OE, AdV-Vehicle. (d) Quantification of OGA protein level shown in panel C, n = 9 per group. (e) Fluorescence images of cardiomyocytes infected with CaMKAR for 24 hours and stained with Hoechst 33342 for 20 min, scale bar = 40 μm. (f) The statistically quantified data on the cellular fluorescence intensity after infected with CaMKAR, n = 5 (HG+AdV-Oga-OE), 6 (CON+AdV-Vehicle, CON+AdV-Oga-OE), and 7 (HG+AdV-Vehicle) biologically independent samples. Data were first assessed for normality and equal variance. For normally distributed data, differences between two groups were analyzed using two-tailed unpaired or paired Student's *t* test, with Welch's correction when variances were unequal. Comparisons among three or more groups were performed using one-way ANOVA followed by Tukey's post hoc test, or Welch's ANOVA when variances were unequal. For non-normally distributed data, the Mann-Whitney U test was used for two-group comparisons. The Kruskal-Wallis test followed by Dunn's multiple comparisons test was used for comparisons among three or more groups. Data were expressed as mean±SEM, *p<0.05, **p<0.01, ***p<0.001, ****p<0.0001. Abbreviations: IP, immunoprecipitation; IB, immunoblotting.

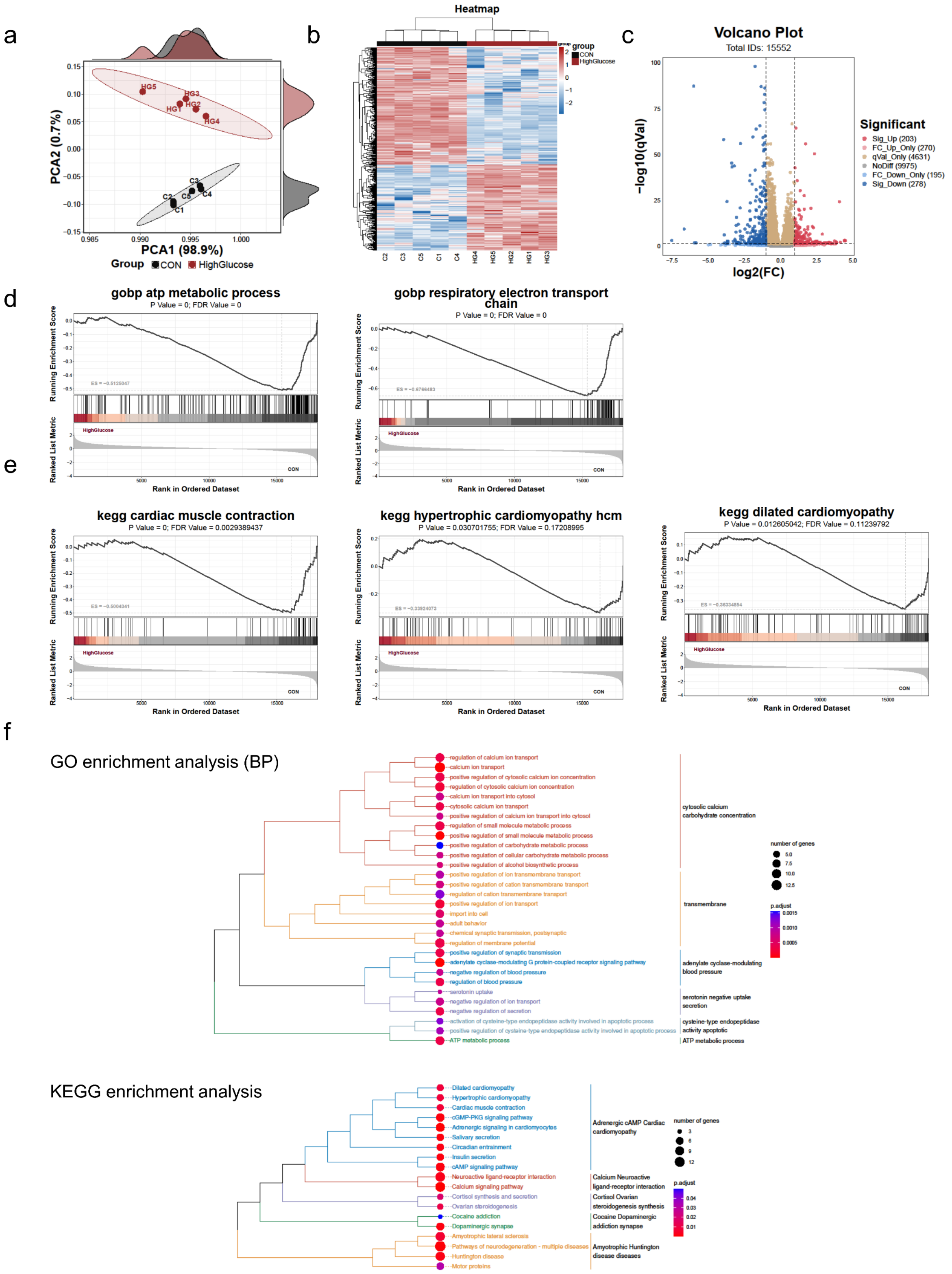

Supplementary Fig. 5. **Supplementary material for transcriptome sequencing of CON and HG groups.** Neonatal hearts at E19.5 were used for the experiments. (a) A PCA plot of transcriptome sequencing and analysis for CON and HG groups. (b) Heatmap of DEGs in

CON and HG groups. (c) Volcano plot of DEGs with color-coded. Genes with $q < 0.05$ and FC > 2 is marked in red; genes with $q < 0.05$ and FC < -2 is marked in blue; genes with $q >$ 0.05 and -2 < FC < 2 is marked in yellow. (d) Enriched pathways of DEGs using GSEA of GO enrichment analysis (BP). (e) The diagram indicated the enriched pathways of DEGs, using GSEA of KEGG. (f) Dendrograms of 70 DEGs associated with CaMKIIδ were performed, using GO enrichment analysis (BP) or KEGG enrichment analysis.

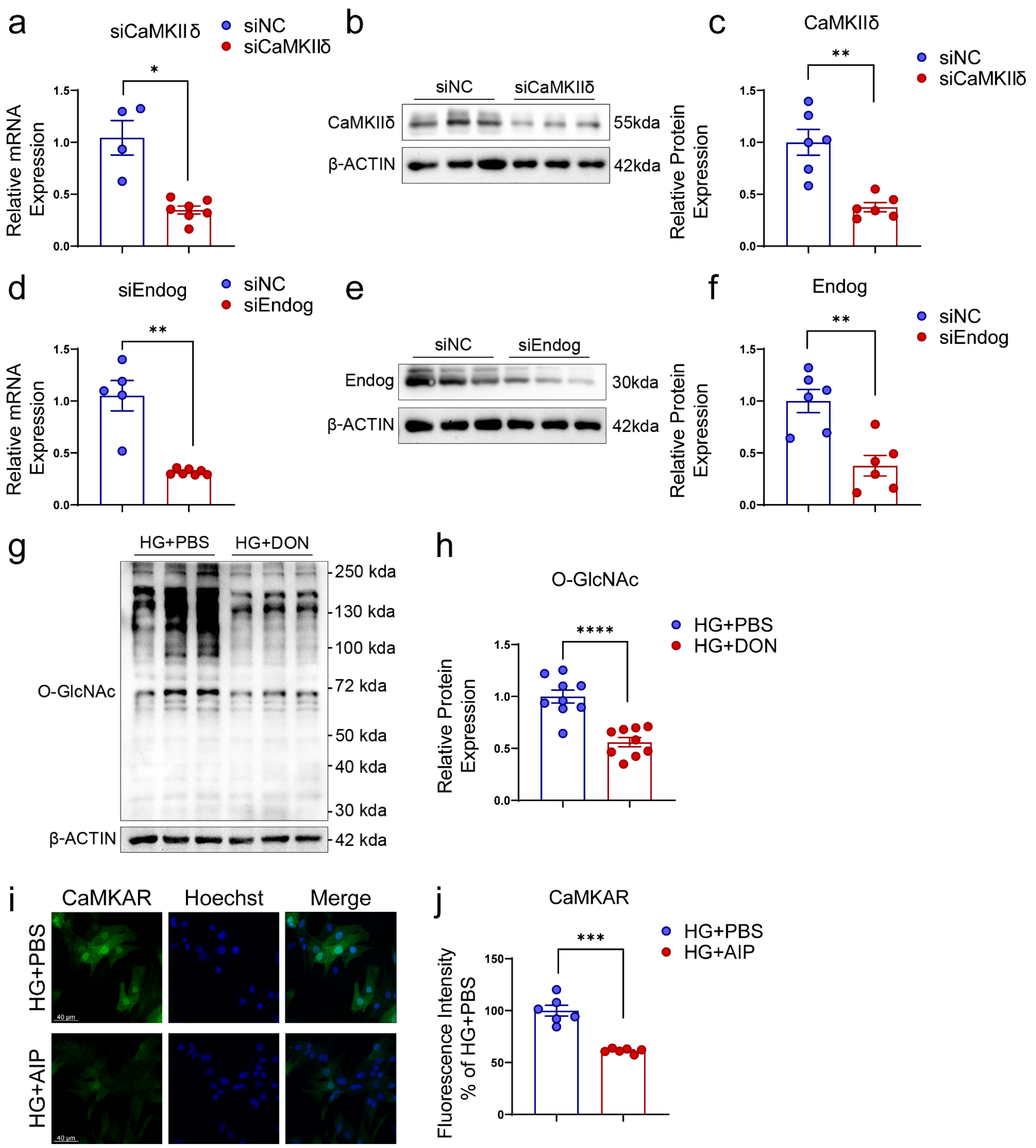

Supplementary Fig. 6. **Evaluation of siRNA Knockdown Efficiency and Inhibitor Efficacy in NRCMs.** Experiments were performed using NRCMs isolated from E19.5 rat hearts. CaMKIIδ siRNA and Endog siRNA were transfected into NRCMs for 48 hours. DON was applied on NRCMs at 50 μM for 48 hours, and AIP at 1 μM for 48 hours. (a) mRNA expression of CaMKIIδ after CaMKIIδ siRNA transfection, n = 4 (siNC), and 7 (siCaMKIIδ) biologically independent samples. (b) Representative immunoblots showing

CaMKIIδ protein levels after CaMKIIδ siRNA transfection. (c) Quantification of CaMKIIδ protein expression in panel B, n = 6 per group. (d) mRNA expression of Endog after Endog siRNA transfection, n = 5 (siNC), and 7 (siEndog) biologically independent samples. (e) Representative immunoblots showing Endog protein levels after Endog siRNA transfection. (f) Quantification of Endog protein expression in panel (e), n = 6 per group. (g) Representative immunoblots of *O*-GlcNAcylation after DON treatment. (h) Quantification of *O*-GlcNAcylation levels in panel (g), n = 9 per group. (i) Fluorescence images of NRCMs infected with CaMKAR for 24 hours and stained with Hoechst 33342 for 20 min. Scale bar = 40 μm. (j) Quantification of fluorescence intensity in panel (i), n = 6 per group. Data were first assessed for normality and equal variance. For normally distributed data, differences were analyzed using two-tailed unpaired or paired Student's *t* test, with Welch's correction when variances were unequal. For non-normally distributed data, the Mann-Whitney U test was used. Data were expressed as mean±SEM, *$p<0.05$, **$p<0.01$, ***$p<0.001$, ****$p<0.0001$.

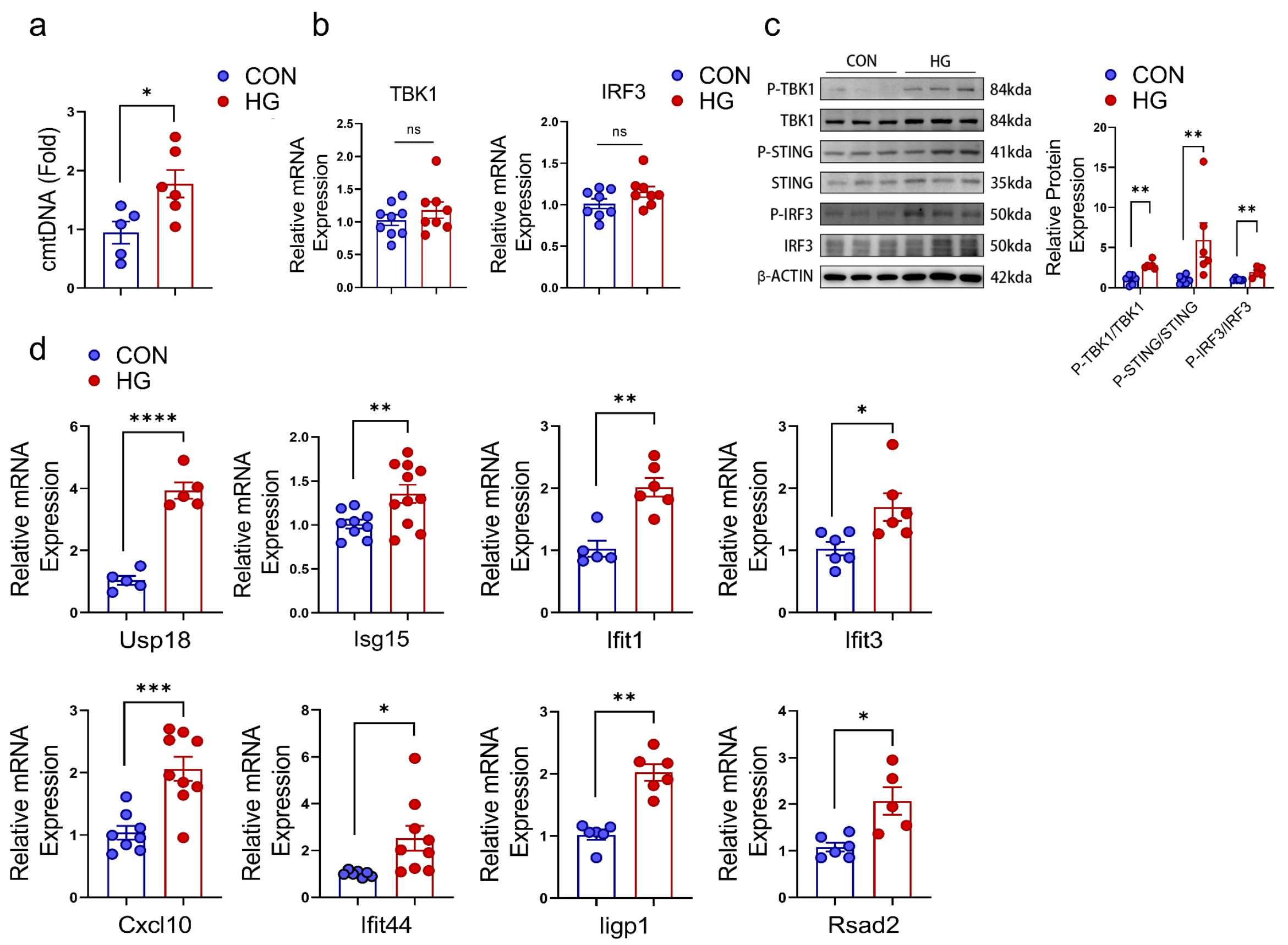

Supplementary Fig. 7. **Increased mtDNA release in the hearts of hyperglycemic neonatal rats led to upregulation of inflammatory genes.** Neonatal hearts at E19.5 were used for the experiments. (a) Quantification cmtDNA levels in neonatal hearts using qPCR, n = 5 (CON), and 6 (HG) biologically independent samples. (b) mRNA expression levels of *Tbk1* were performed by RT-qPCR, n = 8 (HG), and 9 (CON). mRNA expression levels of *Irf3* were performed by RT-qPCR, n = 8 per group. (c) Western blotting of phosphorylated and total protein levels in CON and HG groups. For P-TBK1 and P-STING, n = 6 per group. For P-IRF3, n = 6 in the CON group and n = 5 in the HG group. (d) mRNA expression levels of *Usp18* were performed by RT-qPCR, n = 5. mRNA expression levels of *Isg15* were performed by RT-qPCR, n = 9 (CON) and 11 (HG). mRNA expression levels of *Ifit1* were performed by RT-qPCR, n = 5 (CON) and 6 (HG). mRNA expression levels of *Ifit3* were

performed by RT-qPCR, n = 6. mRNA expression levels of *Cxcl10* were performed by RT-qPCR, n = 8 (CON) and 9 (HG). mRNA expression levels of *Ifi44* were performed by RT-qPCR, n = 7 (CON) and 9 (HG). mRNA expression levels of *Iigp1* were performed by RT-qPCR, n = 6. mRNA expression levels of *Rsad2* were performed by RT-qPCR, n = 5 (HG) and 6 (CON). For RT-qPCR, n represented biologically independent samples. Data were first assessed for normality and equal variance. For normally distributed data, differences were analyzed using two-tailed unpaired or paired Student's *t* test, with Welch's correction when variances were unequal. For non-normally distributed data, the Mann-Whitney U test was used. Data were expressed as mean±SEM, *p<0.05, **p<0.01, ***p<0.001, ****p<0.0001.

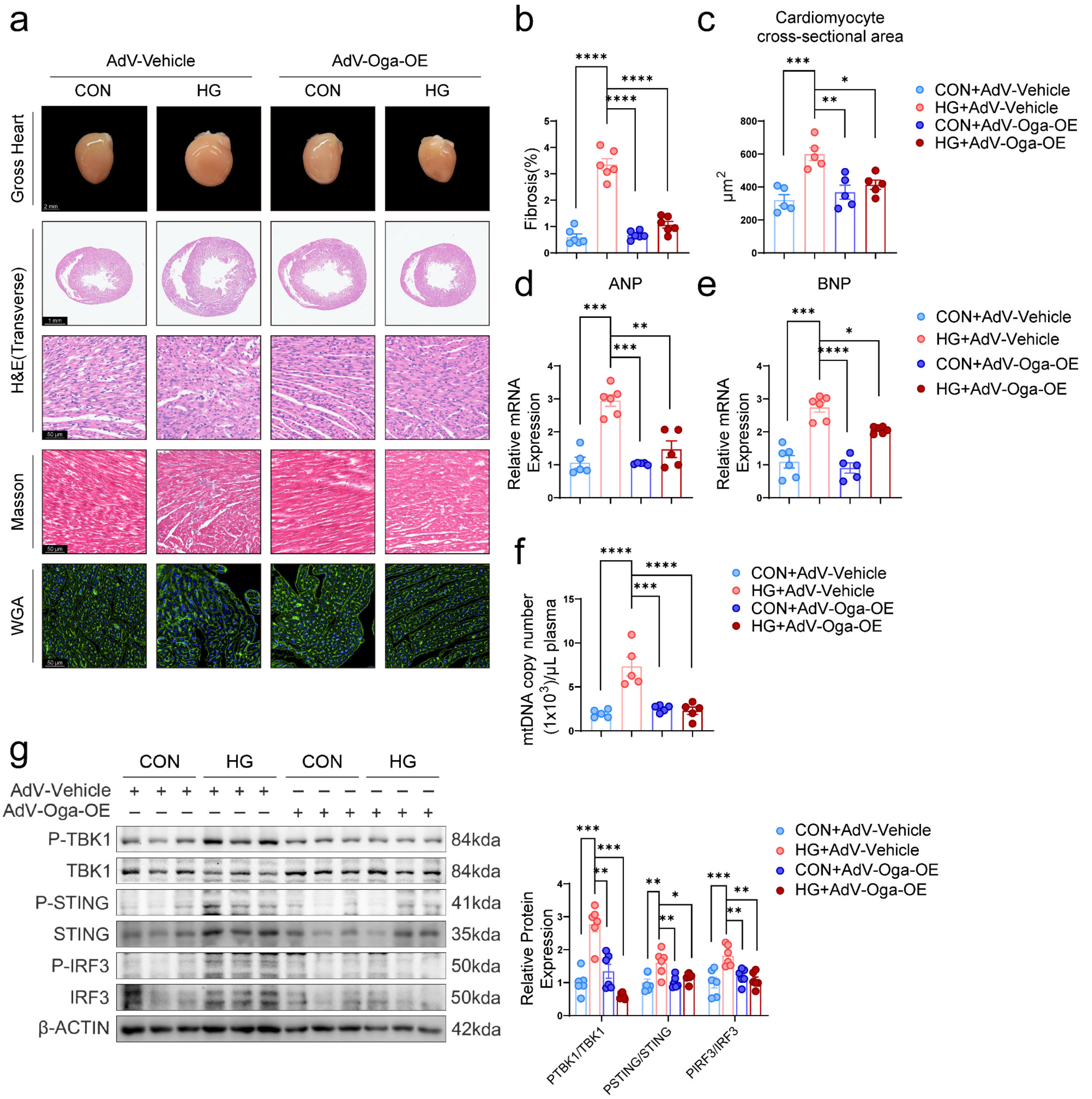

Supplementary Fig. 8. **Overexpression of OGA recovered cardiac remodeling in offspring subjected to intrauterine hyperglycemia.** Neonatal rats received injections on postnatal day 3 and were euthanized on postnatal day 7. (a) Echocardiography, gross morphology and histological analyses were conducted for multiple groups of neonatal hearts. Gross morphology, scale bar = 2.0 mm; HE, Masson and WGA staining, scale bar = 50 μm. (b) Quantitative analysis was performed on fibrotic area (Masson staining), n = 6 per group. (c) Quantitative analysis was performed on cardiomyocyte cross-sectional area (WGA

staining), n = 5 per group. (d) *ANP* gene expression was quantified by RT-qPCR, n = 5 (CON+AdV-Vehicle, CON+AdV-Oga-OE, HG+AdV-Oga-OE) and 6 (HG+AdV-Vehicle) biologically independent samples. (e) *BNP* gene expression was quantified by RT-qPCR, n = 5 (CON+AdV-Oga-OE) and 6 (CON+AdV-Vehicle, HG+AdV-Vehicle, HG+AdV-Oga-OE) biologically independent samples. (f) Plasma mtDNA copy number was assessed, n = 5 per group. (g) Phosphorylated and total protein levels in hearts from multiple groups were detected by western blotting. Densitometric quantification of the blots, n = 6 per group. Data were first assessed for normality and equal variance. For normally distributed data, differences among three or more groups were analyzed using one-way ANOVA followed by Tukey's post hoc test, or Welch's ANOVA when variances were unequal. For non-normally distributed data, the Kruskal-Wallis test followed by Dunn's multiple comparisons test was used for comparisons among three or more groups. Data were expressed as mean±SEM, *$p<0.05$, **$p<0.01$, ***$p<0.001$, ****$p<0.0001$.

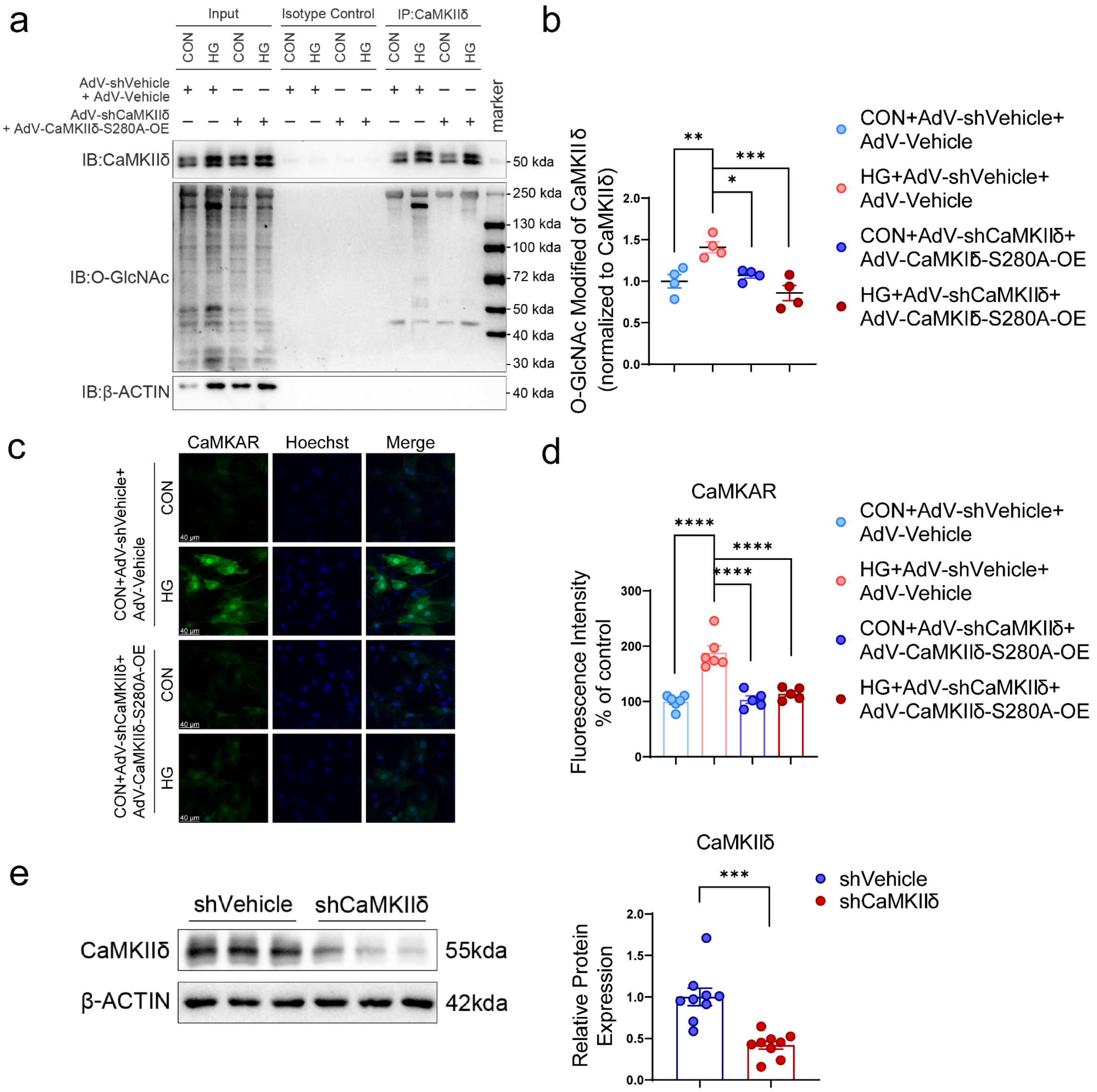

Supplementary Fig. 9**. S280A mutation of CaMKIIδ abolished cardiac CaMKIIδ *O*-GlcNAcylation and reduced CaMKIIδ activation.** Neonatal rats received injections on postnatal day 3 and were euthanized on postnatal day 7. CoIP experiments were performed using heart tissues from P7 neonatal rats. CaMKAR fluorescence imaging experiments were conducted using NRCMs isolated from P7 neonatal hearts. (a) Western blotting analysis of CoIP were measured for the CON+AdV-shVehicle+AdV-Vehicle, HG+AdV-shVehicle+AdV-Vehicle, CON+AdV-shCaMKIIδ+AdV-CaMKIIδ-S280A-OE, HG+AdV-

shCaMKIIδ+AdV-CaMKIIδ-S280A-OE groups, IP: CaMKIIδ, n = 4 per group. (b) The statistically quantified data on the western blotting analysis of *O*-GlcNAc modified CaMKIIδ. (c) Fluorescence images of cardiomyocytes infected with CaMKAR for 24 hours and stained with Hoechst 33342 for 20 min, scale bar = 40 μm. (d) The statistically quantified data on the cellular fluorescence intensity after infected with CaMKAR, n = 5 (CON+AdV-shCaMKIIδ+AdV-CaMKIIδ-S280A-OE, HG+AdV-shCaMKIIδ+AdV-CaMKIIδ-S280A-OE) and 6 (CON+AdV-shVehicle+AdV-Vehicle, HG+AdV-shVehicle+AdV-Vehicle) biologically independent samples. (e) Statistical quantification of CaMKIIδ protein levels following injection of AdV-shCaMKIIδ, n = 9 per group. Data were first assessed for normality and equal variance. For normally distributed data, differences between two groups were analyzed using two-tailed unpaired or paired Student's *t* test, with Welch's correction when variances were unequal. Comparisons among three or more groups were performed using one-way ANOVA followed by Tukey's post hoc test, or Welch's ANOVA when variances were unequal. For non-normally distributed data, the Mann-Whitney U test was used for two-group comparisons. The Kruskal-Wallis test followed by Dunn's multiple comparisons test was used for comparisons among three or more groups. Data were expressed as mean±SEM, *$p<0.05$, **$p<0.01$, ***$p<0.001$, ****$p<0.0001$. Abbreviations: IP, immunoprecipitation; IB, immunoblotting.

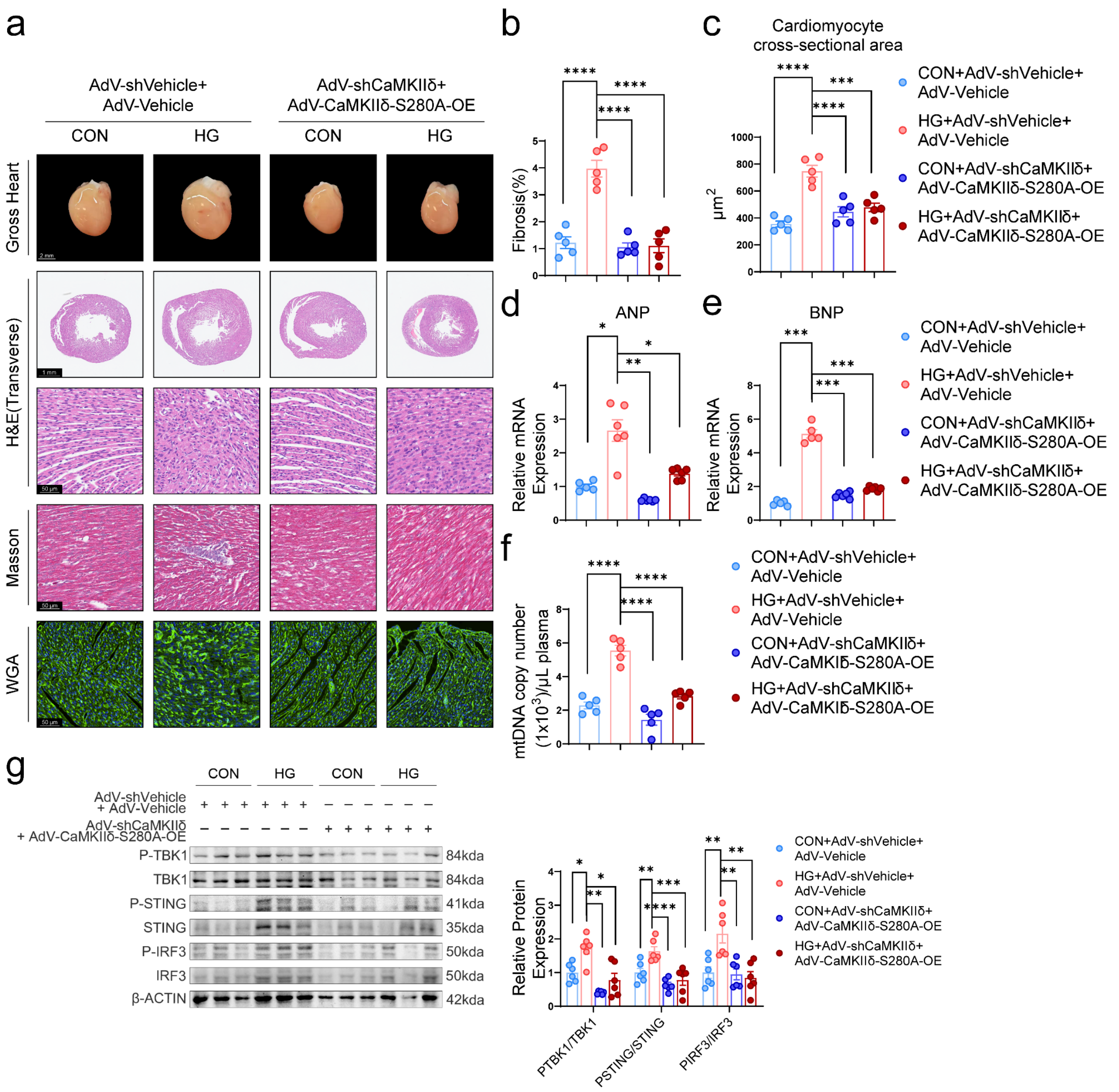

Supplementary Fig. 10. **S280A mutation of CaMKIIδ abolishes cardiac CaMKIIδ *O*-GlcNAcylation in offspring subjected to intrauterine hyperglycemia.** Neonatal rats received injections on postnatal day 3 and were euthanized on postnatal day 7. All experiments in Supplementary Fig. 10 were performed using heart tissues from P7 neonatal rats, except for the plasma mtDNA copy number experiment. (a) Echocardiography, gross morphology and histological analyses were conducted for multiple groups of neonatal hearts. Gross morphology, scale bar = 2.0 mm; HE, Masson and WGA staining, scale bar = 50 μm. (b) Quantitative analysis was performed on fibrotic area (Masson staining), n = 5 per group.

(c) Quantitative analysis was performed on cardiomyocyte cross-sectional area (WGA staining), n = 5 per group. (d) *ANP* gene expression was quantified by RT-qPCR, n = 5 (CON+AdV-shVehicle+AdV-Vehicle) and 6 (HG+AdV-shVehicle+AdV-Vehicle, CON+AdV-shCaMKIIδ+AdV-CaMKIIδ-S280A-OE, HG+AdV-shCaMKIIδ+AdV-CaMKIIδ-S280A-OE) biologically independent samples. (e) *BNP* gene expression was quantified by RT-qPCR, n = 5 (CON+AdV-shVehicle+AdV-Vehicle, HG+AdV-shVehicle+AdV-Vehicle) and 6 (CON+AdV-shCaMKIIδ+AdV-CaMKIIδ-S280A-OE, HG+AdV-shCaMKIIδ+AdV-CaMKIIδ-S280A-OE) biologically independent samples. (f) Plasma mtDNA copy number was assessed, n = 5 per group. (g) Phosphorylated and total protein levels in hearts from multiple groups were detected by western blotting. Densitometric quantification of the blots, n = 6 per group. Data were first assessed for normality and equal variance. For normally distributed data, differences among three or more groups were analyzed using one-way ANOVA followed by Tukey's post hoc test, or Welch's ANOVA when variances were unequal. For non-normally distributed data, the Kruskal-Wallis test followed by Dunn's multiple comparisons test was used for comparisons among three or more groups. Data were expressed as mean±SEM, *$p<0.05$, **$p<0.01$, ***$p<0.001$, ****$p<0.0001$.

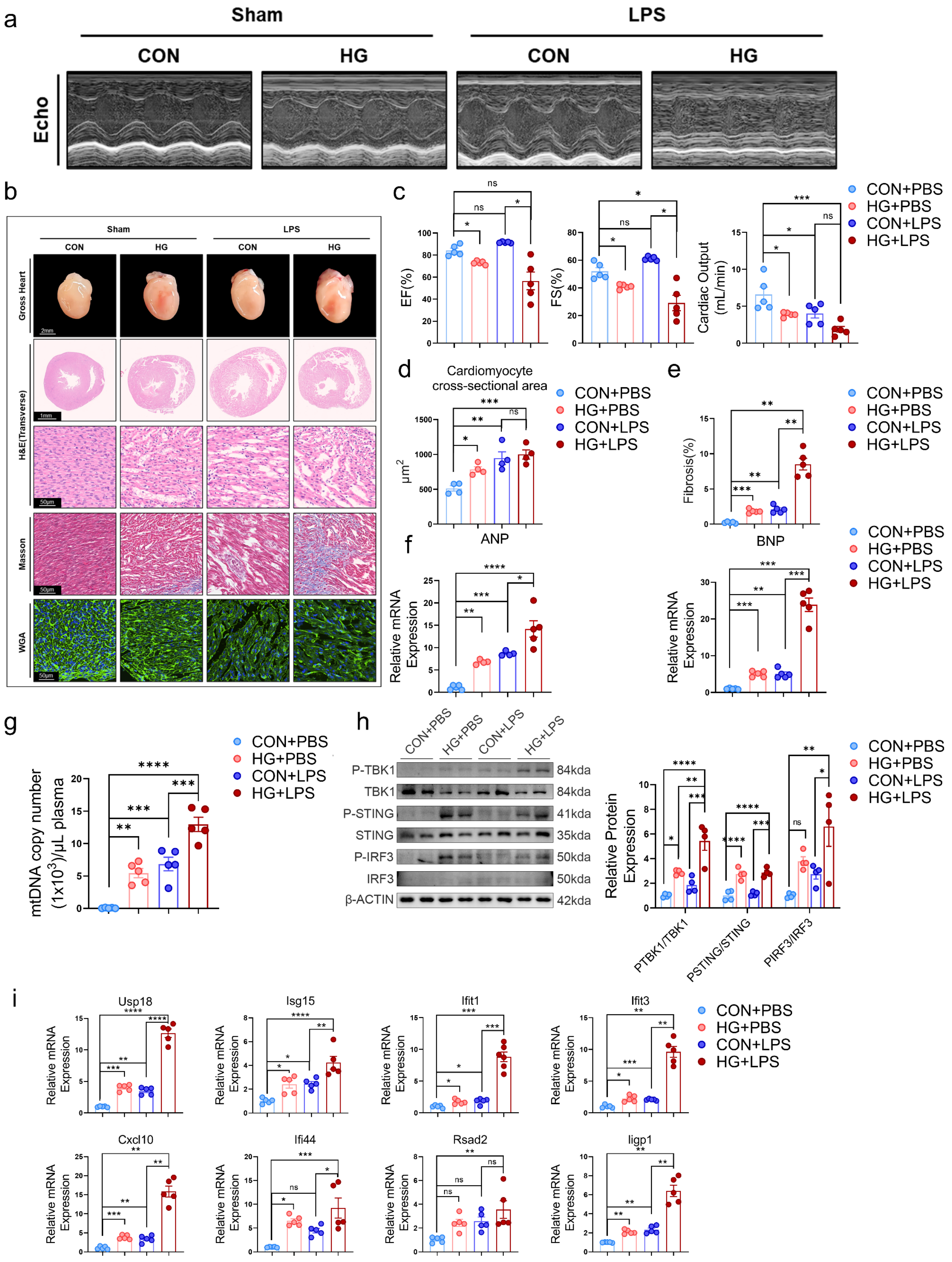

Supplementary Fig. 11. **LPS increased the release of mtDNA and induced inflammatory damage in the hearts of hyperglycemic neonatal rats.** Neonatal rats received a single LPS injection (2 mg/kg, i.p.) on postnatal day 7 (P7). All experiments in Supplementary Fig. 11

were performed using heart tissues of P7 neonatal rats, except for the plasma mtDNA copy number experiment. (a-b) Echocardiography, gross morphology and histological analyses were conducted for multiple groups of neonatal hearts. Gross morphology, scale bar = 2.0 mm; HE, Masson and WGA staining, scale bar = 50 μm. (c) Echocardiographic parameters of EF%, FS% and Cardiac Output were measured, n = 5 per group. (d-e) Quantitative analyses were performed on cardiomyocyte cross-sectional area (WGA staining, n = 4 per group) and fibrotic area (Masson staining, n = 5 per group). (f) mRNA expression levels of *ANP* were quantified by RT-qPCR, n = 4 (HG+PBS, CON+LPS) and 5 (CON+PBS, HG+LPS) biologically independent samples. mRNA expression levels of *BNP* were quantified by RT-qPCR, n = 5 per group. (g) Plasma mtDNA copy number was assessed, n = 5 per group. (h) Phosphorylated and total protein levels in hearts from multiple groups were detected by western blotting. Densitometric quantification of the blots, n = 4 per group. (i) mRNA expression levels of *Usp18*, *Isg15, Ifit3, Cxcl10, Ifi44, Iigp1* and *Rsad2* were quantified by RT-qPCR, n = 5. mRNA expression levels of *Ifit1* were quantified by RT-qPCR, n = 5 (CON+PBS, HG+PBS, CON+LPS) and 6 (HG+LPS). For RT-qPCR, n represented biologically independent samples. Data were first assessed for normality and equal variance. For normally distributed data, differences among three or more groups were analyzed using one-way ANOVA followed by Tukey's post hoc test, or Welch's ANOVA when variances were unequal. For non-normally distributed data, the Kruskal-Wallis test followed by Dunn's multiple comparisons test was used for comparisons among three or more groups. Data were expressed as mean±SEM, *$p<0.05$, **$p<0.01$, ***$p<0.001$, ****$p<0.0001$.

SUPPLEMENTARY TABLES

Supplementary Table 1. **70 overlap genes in Fig. 2j.**

Number	Gene_ID
1	Ace
2	Adcy1
3	Adcyap1r1
4	Adra1a
5	Adra1d
6	Aldob
7	Ank1
8	Arnt2
9	Atp1a3
10	Atp2a3
11	Atp4a
12	Cacng7
13	Cckar
14	Cd74
15	Chrm5
16	Cox8b
17	Creb3l4
18	Ctnnd2
19	Cyp17a1
20	Cyp2j10
21	Cyp2j4
22	Cysltr2
23	Dnah5
24	Dnah6
25	Dnah7
26	Dnah8
27	Drd4
28	Eno3
29	Fcgr2b
30	Fgf23
31	Gipr
32	Gria2
33	Hap1
34	hist1h2ail2
35	Hpse2
36	Hspa1b
37	Il7r
38	Itgax

39	Kcnj9
40	Lama1
41	LOC108348108
42	Mrap
43	Myh6
44	Nos1
45	Ntng2
46	Ntsr1
47	Olr63
48	P2rx5
49	Pde4c
50	Pgam2
51	Pink1
52	Ppara
53	Pparg
54	Ppp2r2b
55	Prkg2
56	Rasgrp1
57	Ret
58	Rims2
59	Ripk3
60	Robo1
61	Rps6ka6
62	Scn1a
63	Slc18a1
64	Snca
65	Tnfsf10
66	Trpa1
67	Tubb4a
68	Vtn
69	Wnt4
70	Zbtb16
