# Supplementary material for: Maternal hyperglycemia-induced O-GlcNAcylation of CaMKIIδ promotes mtDNA release and cardiac remodeling in offspring
Source: Nat Commun. 2026 Jul 29;17:7769. doi: 10.1038/s41467-026-75630-4 (PMC13439061; doi:10.1038/s41467-026-75630-4)
Supplement: Supplementary file 2 [file 41467_2026_75630_MOESM2_ESM.pdf]

nature portfolio

Corresponding author(s): Jun Pu, Xiyuan Lu, Zhice Xu, Ling Gao

Last updated by author(s): Jun 25, 2026

Reporting Summary

Nature Portfolio wishes to improve the reproducibility of the work that we publish. This form provides structure for consistency and transparency in reporting. For further information on Nature Portfolio policies, see our Editorial Policies and the Editorial Policy Checklist.

Statistics

For all statistical analyses, confirm that the following items are present in the figure legend, table legend, main text, or Methods section.

n/a	Confirmed	
☐	☒	The exact sample size (*n*) for each experimental group/condition, given as a discrete number and unit of measurement
☐	☒	A statement on whether measurements were taken from distinct samples or whether the same sample was measured repeatedly
☐	☒	The statistical test(s) used AND whether they are one- or two-sided<br>*Only common tests should be described solely by name; describe more complex techniques in the Methods section.*
☒	☐	A description of all covariates tested
☐	☒	A description of any assumptions or corrections, such as tests of normality and adjustment for multiple comparisons
☐	☒	A full description of the statistical parameters including central tendency (e.g. means) or other basic estimates (e.g. regression coefficient) AND variation (e.g. standard deviation) or associated estimates of uncertainty (e.g. confidence intervals)
☐	☒	For null hypothesis testing, the test statistic (e.g. *F*, *t*, *r*) with confidence intervals, effect sizes, degrees of freedom and *P* value noted<br>*Give P values as exact values whenever suitable.*
☒	☐	For Bayesian analysis, information on the choice of priors and Markov chain Monte Carlo settings
☒	☐	For hierarchical and complex designs, identification of the appropriate level for tests and full reporting of outcomes
☒	☐	Estimates of effect sizes (e.g. Cohen's *d*, Pearson's *r*), indicating how they were calculated

Our web collection on statistics for biologists contains articles on many of the points above.

Software and code

Policy information about availability of computer code

Data collection	No software is used in data collection.
Data analysis	GraphPad Prism 8; clusterProfiler 4.0; ggplot2 (v3.3.3; Wickham, 2016);

For manuscripts utilizing custom algorithms or software that are central to the research but not yet described in published literature, software must be made available to editors and reviewers. We strongly encourage code deposition in a community repository (e.g. GitHub). See the Nature Portfolio guidelines for submitting code & software for further information.

Data

Policy information about availability of data

All manuscripts must include a data availability statement. This statement should provide the following information, where applicable:

- Accession codes, unique identifiers, or web links for publicly available datasets
- A description of any restrictions on data availability
- For clinical datasets or third party data, please ensure that the statement adheres to our policy

The bulk RNA-seq datasets have been deposited in the GEO database under the GSE298866. All data are included in the Supplementary Information or available from the authors, as are unique reagents used in this Article. The raw numbers for charts and graphs are available in the Source Data file whenever possible. Source data are provided with this paper.

Research involving human participants, their data, or biological material

Policy information about studies with human participants or human data. See also policy information about sex, gender (identity/presentation), and sexual orientation and race, ethnicity and racism.

Reporting on sex and gender	No human information has been collected.
Reporting on race, ethnicity, or other socially relevant groupings	No human information has been collected.
Population characteristics	No human information has been collected.
Recruitment	No human information has been collected.
Ethics oversight	No human information has been collected.

Note that full information on the approval of the study protocol must also be provided in the manuscript.

Field-specific reporting

Please select the one below that is the best fit for your research. If you are not sure, read the appropriate sections before making your selection.

☒ Life sciences ☐ Behavioural & social sciences ☐ Ecological, evolutionary & environmental sciences

For a reference copy of the document with all sections, see nature.com/documents/nr-reporting-summary-flat.pdf

Life sciences study design

All studies must disclose on these points even when the disclosure is negative.

Sample size	No formal statistical power calculation was performed to predetermine sample size. For each experiment, sample sizes were determined with based on similar studies previously published in the field, where such sample sizes are widely accepted. The chosen sample sizes were sufficient to produce consistent results across repeated experiments.
Data exclusions	No data were excluded from the analyses. All collected data points were included in the final analysis.
Replication	All Co-IP experiments were independently repeated at least three times using distinct biological replicates, and consistent results were obtained across replicates, confirming the robustness of the data. For other experiments, such as Western blotting, PCR, and histological staining, each data point represents a biological replicate derived from an individual animal or an independent cell culture dish. Findings were ensured by biological replication, multi-method validation, and randomized blinded animal experiments.
Randomization	Female rats were randomly assigned to either the control (CON) group or the hyperglycemia (HG) group to ensure unbiased allocation.<br><br>Investigators were blinded to group allocation during both data collection and analysis. Animal labels were coded by an independent researcher not involved in data analysis. In instances where blinding was not feasible, data analysis was performed by a separate investigator who remained unaware of group assignments.
Blinding	Investigators were blinded to group allocation during both data collection and analysis. Animal identifiers were coded by an researcher who was not involved in data processing. If blinding was not feasible for certain procedures (e.g., due to visible phenotypic differences between groups), data analysis was performed by a separate investigator who was unaware of the experimental conditions.

Reporting for specific materials, systems and methods

We require information from authors about some types of materials, experimental systems and methods used in many studies. Here, indicate whether each material, system or method listed is relevant to your study. If you are not sure if a list item applies to your research, read the appropriate section before selecting a response.

Materials & experimental systems

n/a	Involved in the study
☐	☒ Antibodies
☒	☐ Eukaryotic cell lines
☒	☐ Palaeontology and archaeology
☐	☒ Animals and other organisms
☒	☐ Clinical data
☒	☐ Dual use research of concern
☒	☐ Plants

Methods

n/a	Involved in the study
☒	☐ ChIP-seq
☒	☐ Flow cytometry
☒	☐ MRI-based neuroimaging

Antibodies

Antibodies used

O-linked N-acetylglucosamine (O-GlcNAc) Monoclonal Antibody (RL2) (Thermo Fisher Scientific, MA1-072)
CaMKII delta Polyclonal Antibody(Thermo Fisher Scientific, PA5-22168)
anti-O-GlcNAc monoclonal antibody CTD110.6 (Cell Signaling Technology, America,9875S)
rabbit polyclonal antibody against OGT (Beyotime, AF7651)
rabbit polyclonal antibody against CaMKIIδ (Proteintech, 20667-1-AP)
mouse monoclonal antibody against Beta Tubulin (Proteintech, 66240-1-Ig)
rabbit monoclonal antibody against beta-Actin (ABclonal, AC038)
rabbit monoclonal antibody against TBK1 (Cell Signaling Technology, 3504S)
rabbit monoclonal antibody against Phospho-TBK1 (Cell Signaling Technology, 5483S)
mouse monoclonal antibody against IRF-3 (Santa Cruz Biotechnology, sc-33641)
rabbit monoclonal antibody against Phospho-IRF-3 (Cell Signaling Technology, 29047S)
Phospho-CaMKII beta/gamma/delta (Thr287) Polyclonal Antibody (Thermo Fisher Scientific, PA5-37833)
Anti-oxidized-CaM Kinase II (Met281/282) Antibody (Sigma, 07-1387)
ENDOG Polyclonal antibody (Proteintech, 22148-1-AP)
anti-α-actin antibody (Sigma, A7732)
Rabbit polyclonal antibody against TOM20 (Proteintech, 11802-1-AP)
Mouse monoclonal antibody against dsDNA (Abcam, ab27156)
mouse monoclonal antibody against beta-actin (Proteintech, 66009-1-Ig)
For Western blotting, primary antibodies were used at dilutions ranging from 1:500 to 1:1000.
For immunofluorescence staining, primary antibodies were used at dilutions ranging from 1:50 to 1:100.

Validation

O-linked N-acetylglucosamine (O-GlcNAc) Monoclonal Antibody (RL2) (Thermo Fisher Scientific, MA1-072) has been successfully used in Western blot, immunofluorescence and immunoprecipitation procedures on Amphibian, Bovine, Chemical, Fish, Fruit fly, Hamster, Human, Invertebrate, Mouse, Rat.
CaMKII delta Polyclonal Antibody(Thermo Fisher Scientific, PA5-22168) has been successfully used in Western blot, immunofluorescence and immunoprecipitation on Human, Mouse, Rabbit, Rat.
anti-O-GlcNAc monoclonal antibody CTD110.6 (Cell Signaling Technology, America,9875S) has been successfully used in Western blot on All Species Expected.
Rabbit polyclonal antibody against OGT (Beyotime, AF7651) has been successfully used in Western blot and immunofluorescence on Human, Mouse and Rat.
Rabbit polyclonal antibody against CaMKIIδ (Proteintech, 20667-1-AP) has been successfully used in Western blot, IHC, immunoprecipitation and Flow on Human, Mouse, and Rat.
Mouse monoclonal antibody against Beta Tubulin (Proteintech, 66240-1-Ig) has been successfully used in WB, IHC, IF/ICC, IF-P, IF-Fro, IP, ELISA on human, mouse, rat, pig, zebrafish, nematode.
Rabbit monoclonal antibody against beta-Actin (ABclonal, AC038) has been successfully used in WB, IHC-P, ELISA on Human, Mouse, Rat, Chicken, Zebrafish, Pig.
Rabbit monoclonal antibody against TBK1 (Cell Signaling Technology, 3504S) has been successfully used in WB and IP on Human, Mouse, Rat, and Monkey.
Rabbit monoclonal antibody against Phospho-TBK1 (Cell Signaling Technology, 5483S) has been successfully used in WB, IP, IF, and Flow Cytometry on Human, Mouse, and Rat.
Mouse monoclonal antibody against IRF-3 (Santa Cruz Biotechnology, sc-33641) has been successfully used in WB, IP, IF and IHC(P) on mouse, rat and human.
Rabbit monoclonal antibody against Phospho-IRF-3 (Cell Signaling Technology, 29047S) has been successfully used in WB, IP, IF, and Flow Cytometry on Human, Mouse, and Rat.
Phospho-CaMKII beta/gamma/delta (Thr287) Polyclonal Antibody (Thermo Fisher Scientific, PA5-37833) has been successfully used in WB, ELISA, and Flow Cytometry on Human, Mouse, and Rat.
Anti-oxidized-CaM Kinase II (Met281/282) Antibody (Sigma, 07-1387) has been successfully used in WB and ICC on Human, Mouse, and Rat.
ENDOG Polyclonal antibody (Proteintech, 22148-1-AP) has been successfully used in WB, IHC, IF, IP, and ELISA on human, mouse, rat.
Anti-α-actin antibody (Sigma, A7732) has been successfully used in WB, IHC, IF on cat, rat, fish, rabbit, sheep, bovine, goat, canine, pig, mouse, human, hamster, snake, frog, lizard, chicken.
Rabbit polyclonal antibody against TOM20 (Proteintech, 11802-1-AP) has been successfully used in WB, IHC, IF/ICC, IP, ELISA on human, mouse, rat, chicken.
Mouse monoclonal antibody against dsDNA (Abcam, ab27156) has been successfully used in IHC-P and IF on human, mouse, rat.
mouse monoclonal antibody against beta-actin (Proteintech, 66009-1-Ig) has been successfully used in WB, IHC, IF/ICC, IF-P, FC (Intra), IP, ELISA on human, mouse, rat, pig, rabbit, canine, monkey, chicken, zebrafish, hamster.

Animals and other research organisms

Policy information about studies involving animals; ARRIVE guidelines recommended for reporting animal research, and Sex and Gender in Research

Laboratory animals	Three-month-old maternal and E19.5-P7 Sprague-Dawley (Rattus norvegicus) rats
Wild animals	This study did not involve wild animals.
Reporting on sex	Sex-based information has not been collected. Sex was not considered as a biological variable in this study because neonatal cardiac tissues were used and sex hormone signaling is not fully established at this developmental stage.
Field-collected samples	After a one-week acclimation period, normal female rats were mated with male rats at a 1:1 ratio in the same experimental animal cages in the evening. On the following morning, the male and female rats were separated, and the presence of a vaginal plug in the female rats was checked. The day on which a vaginal plug was observed was designated as gestational day 0 (G0). Subsequently, the female rats were randomly assigned to either the CON group or the HG group. Rats in the HG group received intraperitoneal injections of STZ (50 mg/kg body weight, Sigma, USA) dissolved in 0.01 M citric acid buffer (pH 4.5) after overnight fasting. Rats in the CON group were injected with an equivalent volume of citric acid buffer. Both CON and HG groups received daily injections for three consecutive days. Blood glucose levels were measured using a blood glucose meter (One Touch) 7 days after the injection to confirm that STZ-treated rats exhibited diabetes (non-fasting blood glucose > 16 mM). Rats were kept in a controlled environment at 22°C with a 12/12 hour light-dark cycle. Food and water were freely available.
Ethics oversight	All procedures used have been approved by the Animal Care and Use Committee of Shanghai SINOGENE Biotechnology (Animal license number: XNG-201-2308-003) and follow the guidelines of the National Institutes of Health Guidelines on Care and Use of Laboratory Animals.

Note that full information on the approval of the study protocol must also be provided in the manuscript.

Plants

Seed stocks	*Report on the source of all seed stocks or other plant material used. If applicable, state the seed stock centre and catalogue number. If plant specimens were collected from the field, describe the collection location, date and sampling procedures.*
Novel plant genotypes	*Describe the methods by which all novel plant genotypes were produced. This includes those generated by transgenic approaches, gene editing, chemical/radiation-based mutagenesis and hybridization. For transgenic lines, describe the transformation method, the number of independent lines analyzed and the generation upon which experiments were performed. For gene-edited lines, describe the editor used, the endogenous sequence targeted for editing, the targeting guide RNA sequence (if applicable) and how the editor was applied.*
Authentication	*Describe any authentication procedures for each seed stock used or novel genotype generated. Describe any experiments used to assess the effect of a mutation and, where applicable, how potential secondary effects (e.g. second site T-DNA insertions, mosiacism, off-target gene editing) were examined.*
