# Supplementary material for: Maternal hyperglycemia-induced O-GlcNAcylation of CaMKIIδ promotes mtDNA release and cardiac remodeling in offspring
Source: Nat Commun. 2026 Jul 29;17:7769. doi: 10.1038/s41467-026-75630-4 (PMC13439061; doi:10.1038/s41467-026-75630-4)
Supplement: Supplementary file 3 [file 41467_2026_75630_MOESM3_ESM.pdf]

nature portfolio

Peer Review File

Maternal Hyperglycemia-Induced O-GlcNAcylation of CaMKIIδ Promotes mtDNA Release and Cardiac Remodeling in Offspring

Corresponding Author: Professor Xiyuan Lu

This file contains all reviewer reports in order by version, followed by all author rebuttals in order by version.

Version 0:

Reviewer comments:

Reviewer #1

(Remarks to the Author)
In this paper by Xiao et al, the authors use a rat model to study the impact of intrauterine hyperglycemia on cardiac remodeling and damage in the offspring. They show that hyperglycemia induces mitochondrial morphological and functional alterations in fetal cardiomyocytes and promotes cardiac dysfunctions in the offspring. Mechanistically they linked these alterations to an increased in O-GlcNAcylation that contributes to the activation a CaMKIIδ/mtDNA/STING axis. They indicate that targeting of O-GlcNAcylation could alleviate some of consequences of in utero hyperglycemia. They suggest that detection of plasma mtDNA could serve as a biomarker of neonatal cardiac injury linked to gestational hyperglycemia. The manuscript presents a thorough and logical investigation of the effect of intrauterine hyperglycemia on offspring heart. However, critical scientific and presentation concerns should be fully addressed for the manuscript to be suitable for publication.
Specific Concerns:
1/ The direct demonstration of CamKIId O-GlcNAcylation associated with in utero hyperglycemia is weak. Minimal immuno-precipitation and minimal impact of DON/AIP inhibition are shown in Figure 2D and 2E. As it stands, these results are not conclusive. This is a major claim from the authors (emphasized in the manuscript title and discussion) that is not directly supported by experimental data.
2/ Conditions of use of the DON/AIP inhibitors should be described (timing, concentration…). It is not clear how the authors have set up and validated these conditions to achieve effects. Minimal validation of their effects is needed. Similarly, efficacy of the used siRNA is not evaluated.
3/ Figure 6: Minimal description of the experimental settings, results and conclusions should be provided for each figure: e.g.: one single sentence “Co-transfecting Endog siRNA and Camk2d siRNA led to reduced mtDNA release and lower phosphorylation levels of inflammatory proteins” is supposed to describe a complex 6-panel figure (Fig. 6A-F). A minimal step-by-step description will help the demonstration. As it stands, the figures 6A-F are difficult to interpret for the reader.
4/ While the text is generally well-written, several presentation issues dampen the clarity of the demonstration.
*Figures should be rearranged to appear in the order of their citation in the text (e.g. Fig.1C called before Fig1B, FigS3E-G called before FigS1 and S2, intertwined figure 5 and 6 etc….).
*All Figures should be called in the text. (e.g. Fig S3A-D, Fig 7A-C, FigS4F-H do not appear to be “called” in the text).
* The “Methods” section is incomplete. it should describe the use of the chemical inhibitors and siRNA which are key to the authors’ claims. Other methods are also missing (e.g.: Echocardiography…).

Others points
1/ The authors should clarify the cell type used in the different experiments as there are some confusions between the use of primary fetal heart (e19.5?), primary neonate heart (P?) and cultured neonatal rat cardiomyocytes (NRCM)
2/ line 354: “Additionally, placental weight was significantly higher in the HG group (Fig1D). HG fetuses exhibited lower body weight compared to CON fetuses accompanied by an increased ratio of placenta weight to body weight”. According to the figure, these statements are not correct.
3/ Line 369: “Results showed significantly decreased mitochondrial membrane potential in HG group cardiomyocytes compared to CON group”. Quantification should be provided to assess significance.
4/ The use of DON and AIP as a O-GlcNAcylation and CAMKIIδinhibitors should be defined at first use (currently line 408)
5/ The choice of the LPS treatment used in figure 7 and S4 should be explained. In vivo DON treatment should be described

(duration…).
6/ Figure 5K: oeCAMK2D should read oeCAMKIIδ
7/ Echocardiography results shown in figure 7A and S4A are too small to be readable in the figure.
8/ Line 555: sentence to be revised “Environmental alterations during critical developmental periods impact growth PTM modification, an essential determinant of the onset caused by GDM”.

Reviewer #2

(Remarks to the Author)
Comments to the Authors:
This manuscript by Xiao and colleagues investigates the mechanism by which intrauterine hyperglycemia related to maternal diabetes mellitus (specifically preexisting maternal diabetes mellitus) leads to pathologic fetal cardiac remodeling. O-GlcNAcylation is a nutrient and stress post-translational modification (PTM) that involves the addition of O-Linked β-N-acetylglucosamine (O-GlcNAc) to intracellular proteins that plays a critical role in physiology and pathology, especially in conditions of hyperglycemia such as diabetes mellitus. CaMKII, a protein kinase which is well established as an intracellular signaling node for cardiac disease, was previously shown to undergo O-GlcNAcylation and causally linked to cardiac disease in the context of diabetes mellitus.
This study employed a streptozocin (STZ) induced rat model of diabetes mellitus/intrauterine hyperglycemia. Data presented in this manuscript are interpreted to show fetal myocardium in STZ treated dams have elevated O-GlcNAc and enhanced O-GlcNAcylation of CaMKII. This is associated with disordered intracellular Ca2+ handling, elevated ROS, mitochondrial dysfunction and increased mtDNA release leading to activation of the cGAS-STING pathway and inflammation. Inhibition of O-GlcNAcylation (using DON) or CaMKII (using AIP) effectively attenuated this pathologic response. The strength of this study is the identification of a novel link between CaMKIIδ/mtDNA/cGAS-STING signaling in hyperglycemia dependent mtDNA activation of fetal cardiac remodeling in maternal diabetes mellitus. Also, the study raises the potential of plasma mtDNA as a biomarker for fetal/neonatal cardiac inflammatory injury. However, the conclusions about O-GlcNAcylation of CaMKIIδ in this study is overstated and not convincingly supported by the data presented. The use of DON alone is insufficient to prove the role of O-GlcNAcylation in driving the hypothesized pathway. Further, none of the established CaMKIIδ PTMs, including O-GlcNAcylation (S280), autophosphorylation (T287) and oxidation (MM281/282), were directly tested. Instead, all data rely on CaMKII inhibition (with AIP or siRNA) or over-expression. Knockin mice are available to the field for each of these post-translational modifications on the appropriate CaMKIId background. Although the authors refer to CaMKII activation through the manuscript, there was no direct measurement of CaMKII activation/activity performed. Multiple tools for measuring CaMKII in living cells are available to the community, the most recent and best performing being CaMKAR (https://www.addgene.org/205315/)

Major issues:
1. The banding pattern in the O-GlcNAc blot in Fig 2A is not typical for O-GlcNAc immunoblots. Specifically, the main visible band at 50kDa likely represents non-specific banding or IgG. There are hardly any visible bands above 50kDa. What bands were used for the quantification in the summary data? These blots should be repeated and consider using a GlcNAc competition blot to assess for non-specific banding.

2. What samples were used for the CoIP experiments for O-GlcNAC-CaMKKII in Fig 2D – fetal hearts or NRCMs? In reference to #1 above, the banding pattern for the O-GlcNAc IB for the input samples (Fig 2D) is quite different from the blot in Fig 2A.
In addition, the CaMKIIδ bands do not appear consistent across the samples in Fig 2D and E. Typical CaMKII band is expected at 50kDa. Fig 2E does not show convincing evidence that CaMKIId is O-GlcNAc modified. Given the primary conclusion of this study that O-GlcNAc modification of CaMKII is the key mechanism for the in-utero effects of hyperglycemia in this model, more convincing data is necessary to support this conclusion (see comments above).

3. There is no demonstration that increased O-GlcNAc increases CaMKII activity in this model/study (see above).

4. O-GlcNAcylation is a dynamic process regulated exclusively by two enzymes – O-GlcNAc transferase (OGT) and O-GlcNAcase (OGA). Protein expression of OGT was measured (Fig 2B) and elevated in the HG group. It will be insightful to also measure OGA expression in these groups/samples.

5. Treatment with AIP or DON will inhibit CaMKII or O-GlcNAc respectively but the effects observed do not necessarily prove inhibition of CaMKIIδ O-GlcNAcylation specifically as suggested in line 450. Mice with myocardial-targeted OGA transgenic over-expression are published and available to the field and would be a dispositive reagent for these studies.

6. How were AIP and DON administered, this is not clear in the text of the manuscript although Fig 3 legend would suggest cardiomyocytes were treated with these compounds. Can the authors clarify – were these in the pregnant maternal rats or fetuses vs in in-vitro experiments with cardiomyocytes. What doses and routes were administered and how long was the duration of treatment? AIP should be defined at first use, Autocamtide-2-Related Inhibitory Peptide (AIP)

7. Is it helpful to measure the insulin levels especially in the fetuses? Are the effects seen due to hyperglycemia vs hyperinsulinemia or both?

8. In this study, fetal body weight was down in the fetuses of hyperglycemic maternal rats (Fig 1D). How do the authors

explain this finding in the context of what is known about hyperinsulinism in gestational diabetes resulting in macrosomia/big babies?

9. Lines 602 – 605, some of the claims here in the discussion about mitochondrial Ca2+ homeostasis, mitochondrial swelling and opening of mPTP are overstated, as they were not directly tested in this study as suggested.

Minor issues:
1. Was STZ injected for 3 days consecutively? It is not clearly stated in the manuscript.

2. How long were the female mice diabetic prior to mating with normal male rats?

3. For the data in Fig 2J, may be helpful for the overlap 70 genes be legibly displayed for instance in a supplemental table in addition to the figure in 2J.

4. Line 432 – 'Given that O-Glycan biosynthesis pathways and abnormal Ca2+ homeostasis are the top of 'core pathways' – what is the basis of this statement?

5. Several abbreviations should be defined at first mention in the manuscript. Examples include - AIP - Autocamtide-2-Related Inhibitory Peptide
- GDM – gestational diabetes mellitus

Reviewer #3

(Remarks to the Author)
The authors investigate the mechanisms through which maternal diabetes during pregnancy causes cardiac remodeling and inflammation in the offspring's hearts. This study proposes an intriguing mechanism involving CaMKIIδ O-GlcNAcylation, which leads to mtDNA leakage and subsequent activation of the cGAS-STING pathway, driving cardiac remodeling. Although the human relevance is unclear, the conceptual framework is solid, and the authors present insightful data to support their hypothesis.
However, the manuscript should be organized more effectively to enhance its flow and overall readability, and some figures and data appear unconvincing. Below are specific comments:

General Comments:
Unconvincing representative images raise concerns about reproducibility.
More proper alignment between representative data and quantifications is needed.
Ensuring logical flow in figure presentation and improving the quality of supporting data will greatly enhance the manuscript's clarity and impact.

Reorganization:
1. The authors start with methods, which deviates from the typical order of presentation in papers published in Nature Communications.
2. The order of the figures (e.g., Fig. 6A-F appearing before Fig. 5K-O) makes it challenging to follow the story.

Data and Figures:
1. Fig. 2D and E: These images do not clearly demonstrate O-GlcNAcylation in CaMKIIδ.
2. Fig. 2H and J: The labels are too small, making the data difficult to interpret.
3. AIP and DON explanations: The explanation for these compounds appears after their initial presentation in the data, disrupting the logical flow.
4. Fig. 3F: No significant difference is observed in mitosox between HG and HG+AIP conditions.
5. Fig. 3I: Are the imaging conditions consistent? In some figures, the signal appears saturated, and the mitotracker staining is unclear in HG+AIP.
6. Fig. 3H: The representative images do not align with the quantification data; HG and HG+AIP appear similar.
7. Fig. 5C: One of the figure borders is missing.
8. Fig. 5E and J: The β-actin levels differ significantly, suggesting variations in protein loading. Baseline variations make the interpretation of the blots unclear.
9. Fig. 5E, J, and O: The changes in p-IRF3/IRF3 are not significant due to substantial IRF3 upregulation. The authors should consider an alternative method of quantification
10. Fig. 6A: The representative images of siEndog do not demonstrate the quantification as depicted in Fig. 6B and C. Knockdown efficiency for siEndog is missing.
11. Fig. 6E: The β-actin signal is very weak, raising concerns about protein loading amount.
12. Fig. 6H: The cells in the "Vehicle" condition should be centered for clarity.
13. Fig. 7: The rationale for adding LPS is unclear. Does it enhance mitochondrial injury and mtDNA leakage without TLR4 CaMKII signaling? Providing a clearer explanation would benefit readers.

Version 1:

Reviewer comments:

Reviewer #1

(Remarks to the Author)
The authors have responded effectively to my scientific comments. The scope of this work is impressive. Overall, this is a compelling and impactful study, and I have no further scientific concerns.
However, a few presentation-related issues remain:
1/ The paragraph spanning lines 185–216 is convoluted and noticeably weaker than the rest of the manuscript. It should be reworked to improve the logical flow. In particular, the text should be logically articulated, progressing from the rationale for focusing on CaMKIIδ, the absence of changes in CaMKIIδ expression but evidence of enhanced CaMKIIδ activation (including sensor readouts and residue-specific phosphorylation), the increased O-GlcNAcylation and the functional modulation via OGA overexpression.
Additionally, AIP should be introduced and discussed in this paragraph, as AIP treatment is presented in Figures 2D and 2E.

2/ The authors should make every effort to order all the supplementary figures according to their first citation in the main text. The seemingly random referencing of supplementary figures is distracting and inconvenient for the reader (for example, supplementary figure 11 appears before supplementary figure 2)

3/ The authors should carefully review the size of all figure panels to ensure that text, labels, and data are easily readable.

Reviewer #2

(Remarks to the Author)
multiple revisions and new experimental data fortify the conclusions

Open Access This Peer Review File is licensed under a Creative Commons Attribution 4.0 International License, which permits use, sharing, adaptation, distribution and reproduction in any medium or format, as long as you give appropriate credit to the original author(s) and the source, provide a link to the Creative Commons license, and indicate if changes were made.
In cases where reviewers are anonymous, credit should be given to 'Anonymous Referee' and the source.
The images or other third party material in this Peer Review File are included in the article's Creative Commons license, unless indicated otherwise in a credit line to the material. If material is not included in the article's Creative Commons license and your intended use is not permitted by statutory regulation or exceeds the permitted use, you will need to obtain permission directly from the copyright holder.
To view a copy of this license, visit https://creativecommons.org/licenses/by/4.0/

Detailed Responses to Reviewer #1:

General Comments:

In this paper by Xiao et al, the authors use a rat model to study the impact of intrauterine hyperglycemia on cardiac remodeling and damage in the offspring. They show that hyperglycemia induces mitochondrial morphological and functional alterations in fetal cardiomyocytes and promotes cardiac dysfunctions in the offspring. Mechanistically they linked these alterations to an increased in *O*-GlcNAcylation that contributes to the activation a CaMKIIδ/mtDNA/STING axis. They indicate that targeting of *O*-GlcNAcylation could alleviate some of consequences of in *utero* hyperglycemia. They suggest that detection of plasma mtDNA could serve as a biomarker of neonatal cardiac injury linked to gestational hyperglycemia. The manuscript presents a thorough and logical investigation of the effect of intrauterine hyperglycemia on offspring heart. However, critical scientific and presentation concerns should be fully addressed for the manuscript to be suitable for publication.

<u>Response</u>:

Thanks for your positive comments that "<u>The manuscript presents a thorough and logical investigation of the effect of intrauterine hyperglycemia on offspring heart</u>". We have followed your suggestions and made every possible effort to address the concerns. Detailed responses to all further comments are provided below.

Specific Concerns:

(Q1) The direct demonstration of CamKIId *O*-GlcNAcylation associated with in *utero* hyperglycemia is weak. Minimal immuno-precipitation and minimal impact of DON/AIP inhibition are shown in Figure 2D and 2E. As it stands, these results are not conclusive. This is a major claim from the authors (emphasized in the manuscript title and discussion) that is not directly supported by experimental data.

<u>Response</u>:

We sincerely appreciate your insightful critique. To further confirm the role of CaMKIIδ *O*-GlcNAcylation in intrauterine hyperglycemia-induced cardiac dysfunction, we performed a series of additional experiments:

1) According to your suggestions, we revised Figure 2D and 2E with more representative images and conducted statistical analysis of the data to confirm the occurrence of CaMKIIδ *O*-GlcNAcylation in fetal cardiomyocytes subjected to in *utero* hyperglycemia, as well as the inhibitory effects of AIP and DON on CaMKIIδ *O*-GlcNAcylation. The results demonstrated a significant increase in CaMKII *O*-GlcNAcylation in NRCMs from the HG+PBS group, which was effectively attenuated by DON treatment (Figure 2D and 2E in the revised Manuscript, which is inserted below for the Reviewer's convenience).

D

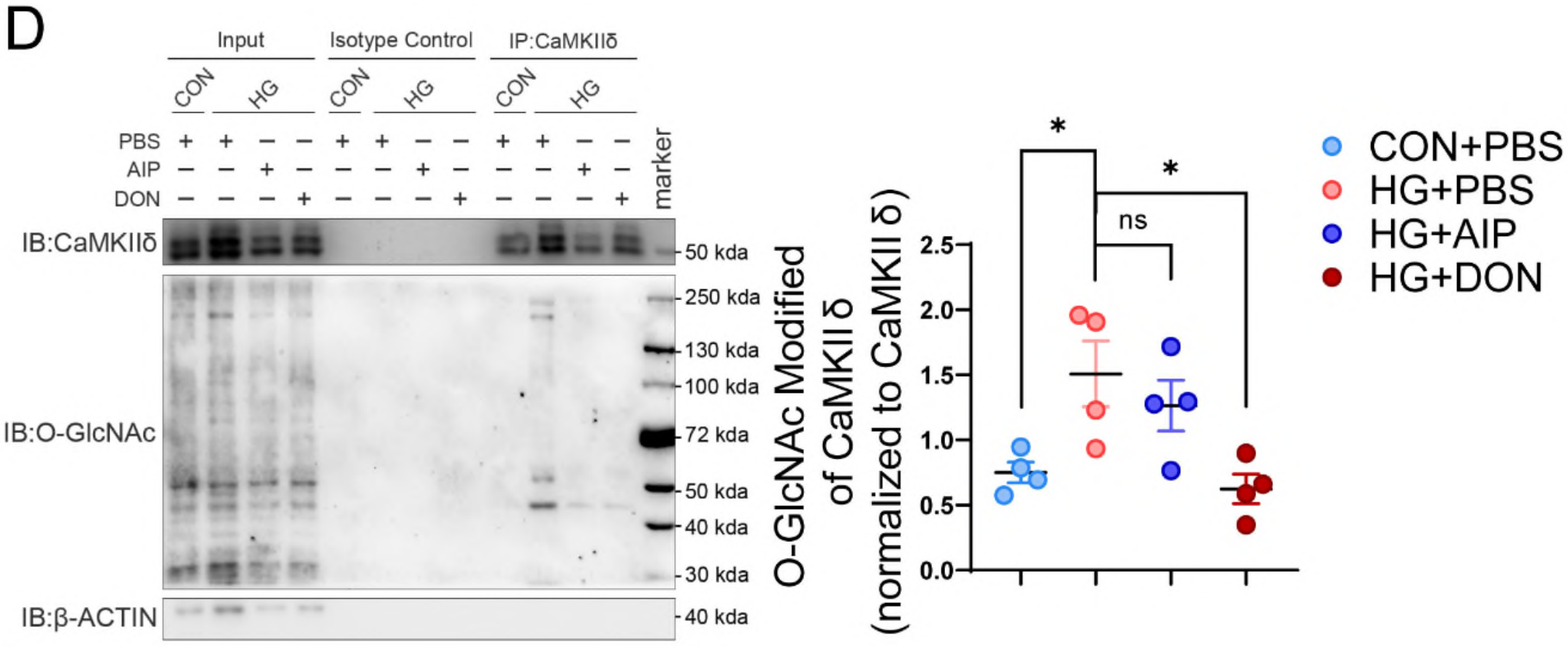

E

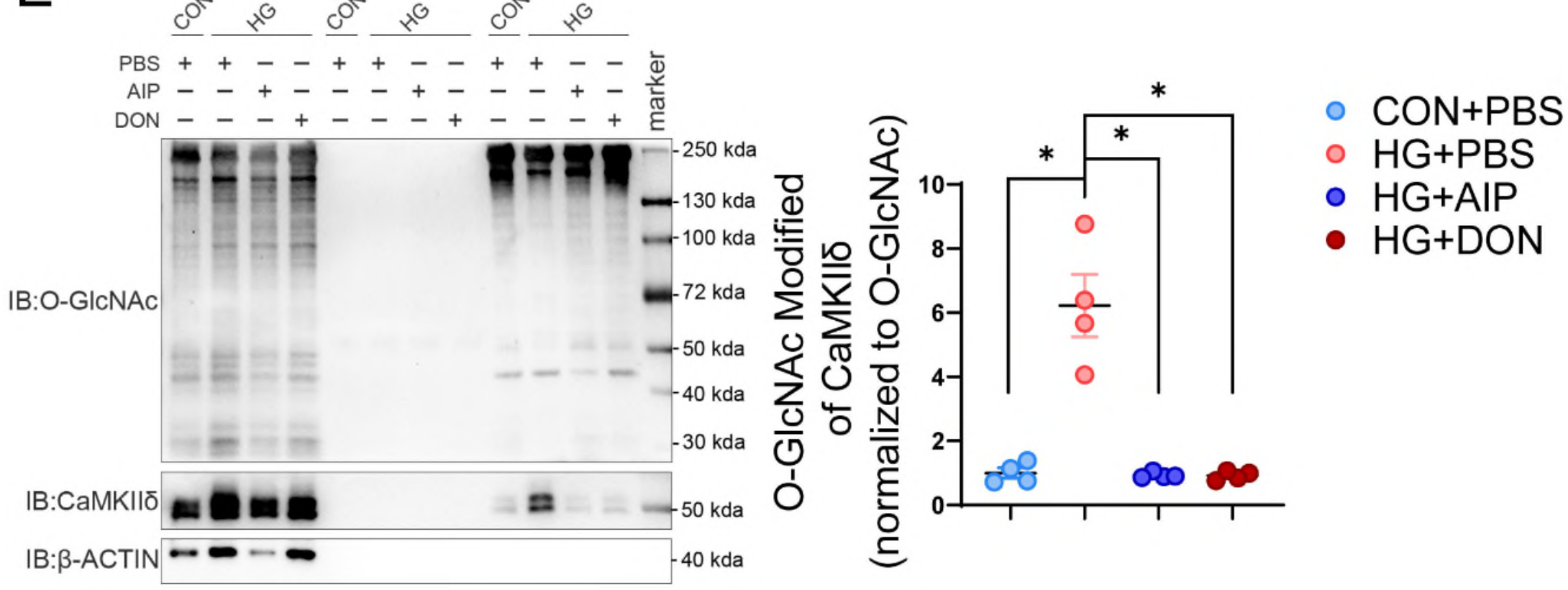

Fig 2D and 2E: CoIP western blotting and analysis of CaMKIIδ *O*-GlcNAcylation. CoIP experiments were performed using NRCMs isolated from E19.5 neonatal hearts, DON was applied on NRCMs at 50 μM for 48 hours, and AIP at 1 μM for 48 hours. (D) Western blotting of CoIP were performed for the CON+PBS, HG+PBS, HG+AIP, HG+DON groups (left). IP: CaMKIIδ. Statistical quantification of the blots (right), n = 4 per group. (E) Western blotting of CoIP were performed for the CON+PBS, HG+PBS, HG+AIP, HG+DON groups (left). IP: *O*-GlcNAc. Statistical quantification of the blots (right), n = 4 per group. For statistical comparisons, data were initially evaluated for normality and equality of variances. One-way analysis of variance (ANOVA) was used to determine statistical differences among multiple groups. If equal variances were not assumed, Welch's ANOVA was applied. Data were expressed as mean±SEM, *p<0.05, **p<0.01, ***p<0.001, ****p<0.0001. Abbreviations: IP, immunoprecipitation; IB, immunoblotting.

2) To validate the role of CaMKIIδ *O*-GlcNAcylation *in vivo*, we engineered two cardiac-specific adenoviral vectors to dissect the functional impact of CaMKIIδ *O*-GlcNAcylation:

i) pADV-cTNT-Camk2d (p.S280A)-HA (abbreviated as AdV-CaMKIIδ-S280A-OE): To preserve kinase activity while blocking hyperglycemia-induced post-translational modification, we overexpressed a CaMKIIδ mutant with abolished *O*-GlcNAcylation at Ser280 (S280A mutation).

ii) pADV-cTNT-mCherry-3xFLAG-shCamk2d (abbreviated as AdV-shCaMKIIδ): To isolate the effects of *O*-GlcNAcylation from baseline CaMKIIδ signaling, we knocked down endogenous CaMKIIδ (58% reduction in protein levels, p = 0.0004).

Simultaneous intramyocardial injection of these two adenoviral vectors into neonatal rat hearts resulted in a specific reduction of CaMKIIδ *O*-GlcNAcylation in *utero* hyperglycemia.

Next, to verify changes in CaMKIIδ *O*-GlcNAcylation following *in vivo* gene interventions, we isolated NRCMs from hearts injected with adenoviral vectors and performed CoIP experiments. According to your suggestions, we performed CoIP experiments using a crosslink IP kit (Thermo Scientific, USA, Cat#88848), which covalently immobilizes the antibody to magnetic beads. This method ensured that the eluted samples used for subsequent western blotting were free of antibody-derived IgG contamination. As shown in Supplementary Fig. 9, overexpression of the CaMKIIδ S280A mutant effectively reduced cardiac CaMKIIδ *O*-GlcNAcylation in offspring subjected to intrauterine hyperglycemia. Moreover, the S280A mutant reduced CaMKIIδ activation in response to intrauterine hyperglycemia (inserted below for the Reviewer's convenience).

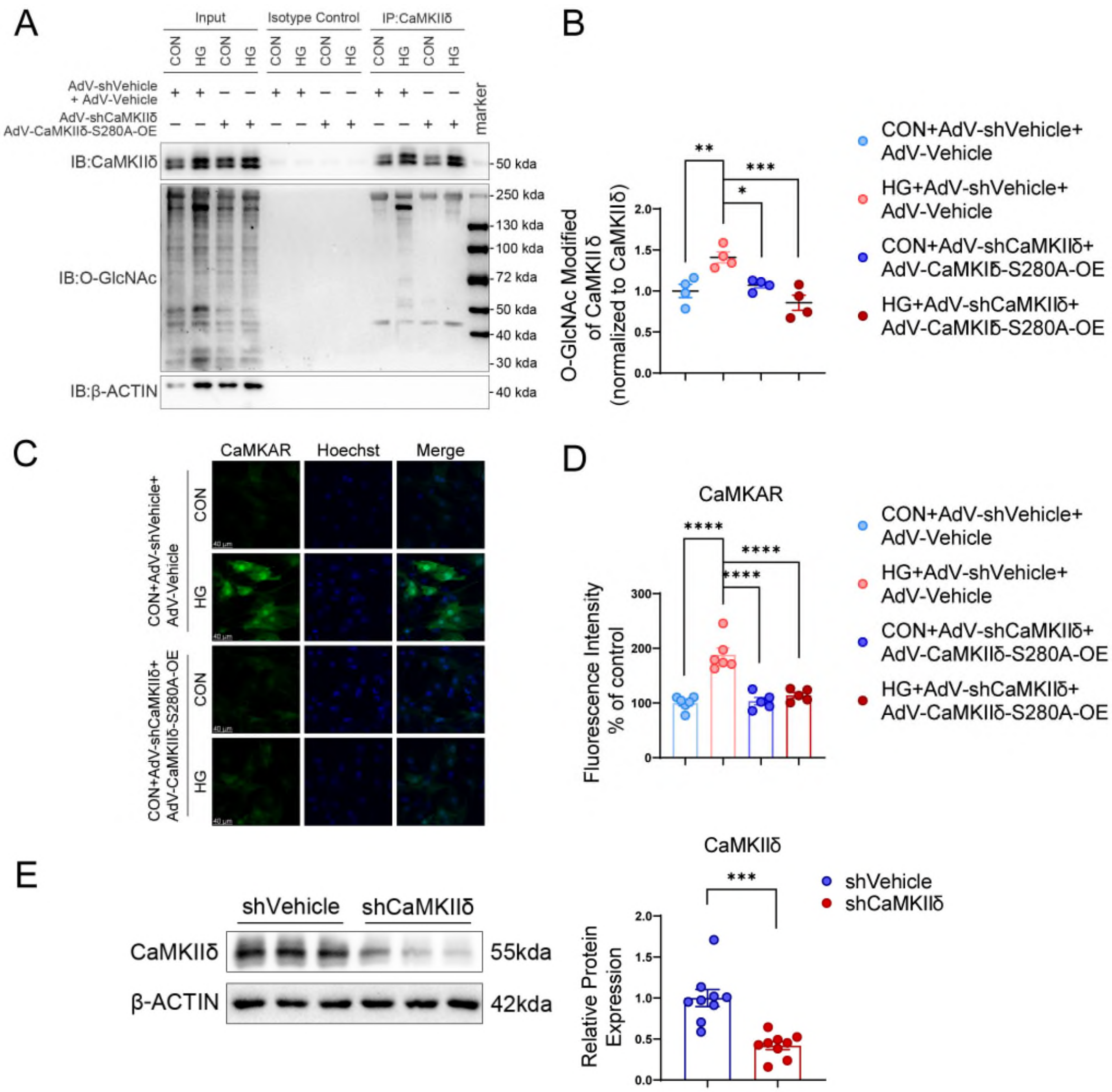

Supplementary Fig. 9: S280A mutation of CaMKIIδ abolished cardiac CaMKIIδ *O*-GlcNAcylation and reduced CaMKIIδ activation. Neonatal rats received injections on postnatal day 3 and were euthanized on postnatal day 7. CoIP experiments were performed using heart tissues from P7 neonatal rats. CaMKAR fluorescence imaging experiments were conducted using NRCMs isolated from P7 neonatal hearts. (A) Western blotting analysis of CoIP were measured for the CON+AdV-shVehicle+AdV-Vehicle, HG+AdV-shVehicle+AdV-Vehicle, CON+AdV-shCaMKIIδ+AdV-CaMKIIδ-S280A-OE, HG+AdV-shCaMKIIδ+AdV-CaMKIIδ-S280A-OE groups, IP: CaMKIIδ, n = 4 per group. (B) The statistically quantified data on the western blotting analysis of *O*-GlcNAc modified CaMKIIδ. (C) Fluorescence images of cardiomyocytes infected with CaMKAR for 24 hours and stained with Hoechst33342 for 20 min, scale bar = 40 μm. (D) The statistically quantified data on the cellular

fluorescence intensity after infected with CaMKAR, n = 5-6 per group. (E) Statistical quantification of CaMKIIδ protein levels following injection of AdV-shCaMKIIδ, n = 9 per group. For statistical comparisons, data were initially evaluated for normality and equality of variances. Student's *t* test and one-way ANOVA were used to determine the levels of statistical differences between two groups and among multiple groups, respectively. If equal variances were not assumed, Welch's *t* test or Welch's ANOVA were applied. Data were expressed as mean±SEM, *p<0.05, **p<0.01, ***p<0.001, ****p<0.0001. Abbreviations: IP, immunoprecipitation; IB, immunoblotting.

3) To investigate *in vivo* changes in cardiac function following AdV-CaMKIIδ-S280A overexpression, we conducted a series of histological analyses and western blotting experiments. As shown in Supplementary Fig. 10, overexpression of the CaMKIIδ S280A mutant effectively attenuated myocardial fibrosis in offspring subjected to intrauterine hyperglycemia. Moreover, the S280A mutant reduced cardiomyocyte cross-sectional area in the HG group. Additionally, the expression levels of ANP and BNP were decreased in the HG+AdV-shCaMKIIδ+AdV-CaMKIIδ-S280A-OE group compared to the HG+AdV-shVehicle+AdV-Vehicle group. Notably, plasma mtDNA levels were also reduced in the HG+AdV-shCaMKIIδ+AdV-CaMKIIδ-S280A-OE group (inserted below for the Reviewer's convenience).

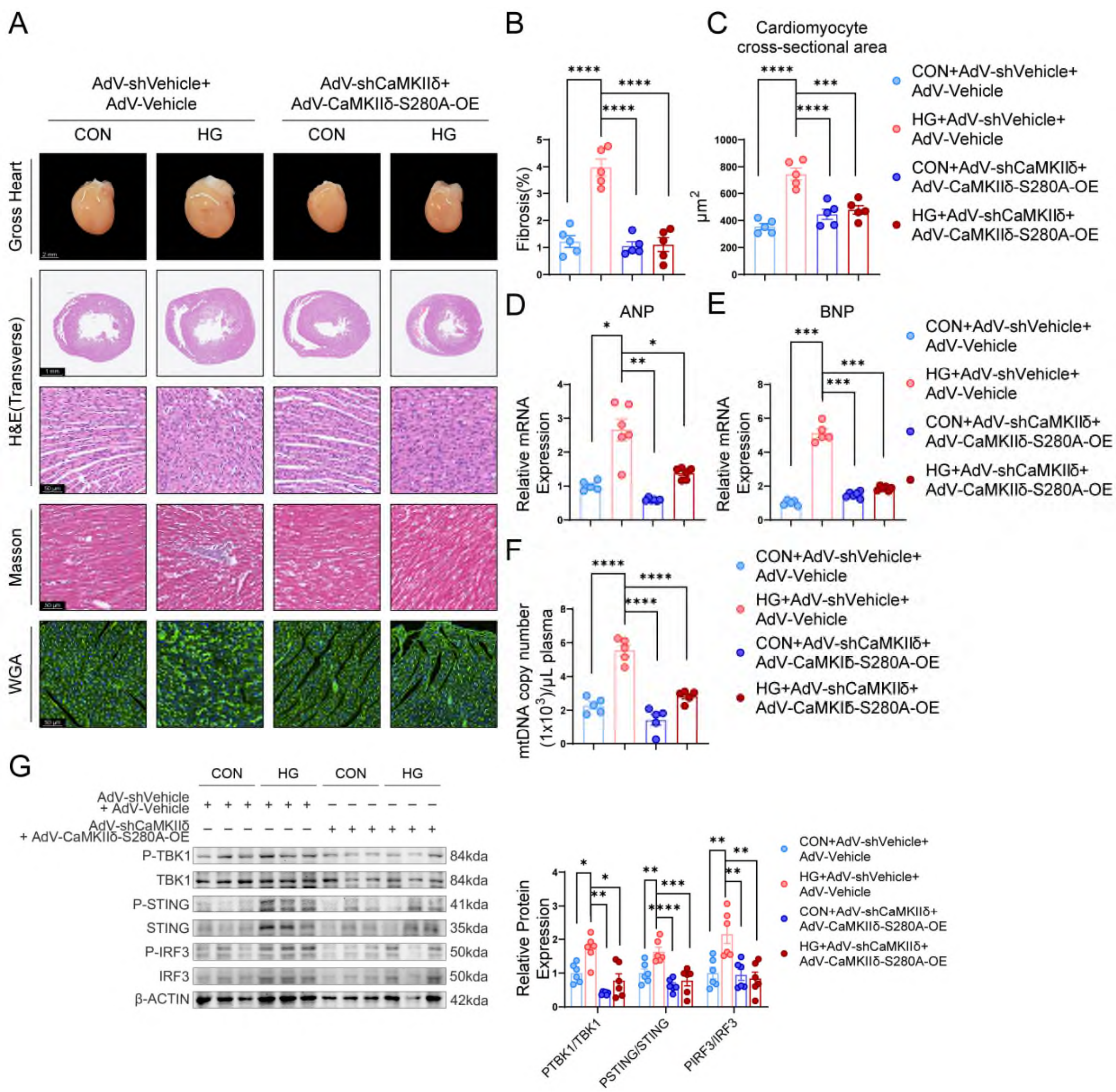

Supplementary Fig. 10: S280A mutation of CaMKIIδ abolishes cardiac CaMKIIδ *O*-GlcNAcylation in offspring subjected to intrauterine hyperglycemia. Neonatal rats received

injections on postnatal day 3 and were euthanized on postnatal day 7. All experiments in Supplementary Fig. 10 were performed using heart tissues from P7 neonatal rats, except for the plasma mtDNA copy number experiment. (A) Echocardiography, gross morphology and histological analyses were conducted for multiple groups of fetal hearts. Gross morphology, scale bar = 2.0 mm; HE, Masson and WGA staining, scale bar = 50 μm. (B) Quantitative analysis was performed on fibrotic area (Masson staining), n = 5 per group. (C) Quantitative analysis was performed on cardiomyocyte cross-sectional area (WGA staining), n = 5 per group. (D) ANP gene expression was quantified by RT-qPCR, n = 5-6 per group. (E) BNP gene expression was quantified by RT-qPCR, n = 5-6 per group. (F) Plasma mtDNA copy number was assessed, n = 5 per group. (G) Phosphorylated and total protein levels in hearts from multiple groups were detected by western blotting (left). Densitometric quantification of the blots (right), n = 6 per group. For statistical comparisons, data were initially evaluated for normality and equality of variances. One-way ANOVA was used to determine statistical differences among multiple groups. If equal variances were not assumed, Welch's ANOVA was applied. Data were expressed as mean±SEM, *p<0.05, **p<0.01, ***p<0.001, ****p<0.0001.

We hope these additional experiments address your concerns and strengthen the support for this critical claim in our manuscript.

(Q2) Conditions of use of the DON/AIP inhibitors should be described (timing, concentration…). It is not clear how the authors have set up and validated these conditions to achieve effects. Minimal validation of their effects is needed. Similarly, efficacy of the used siRNA is not evaluated.

Response:

Thanks for your constructive comments. Following your suggestion, we provided the detailed information on the timing and concentration of the DON/AIP inhibitors. Specifically, DON was applied at 50 μM for 48 hours, and AIP at 1 μM for 48 hours. These experimental conditions were based on previous literature and were optimized to achieving effective inhibition[1-4]. We have now included additional data validating the efficacy of these treatments (Supplementary Fig. 5 in the revised Manuscript, which is inserted below for the Reviewer's convenience).

Furthermore, we have added the following statement in the **Materials and Methods** section of the revised Manuscript: "Following cell attachment, the inhibitors were added directly to the culture medium and maintained. NRCMs were treated with the CaMKIIδ inhibitor AIP (1 μM; HY-P0214, MedChemExpress, China) or the *O*-GlcNAcylation inhibitor DON (50 μM; S8620, Selleck, USA) for 48 hours prior to harvest[2,5,6]. CsA (10 μM; HY-B0579, MedChemExpress, China) was added to the culture medium for the final 4 hours prior to sample collection[7]."

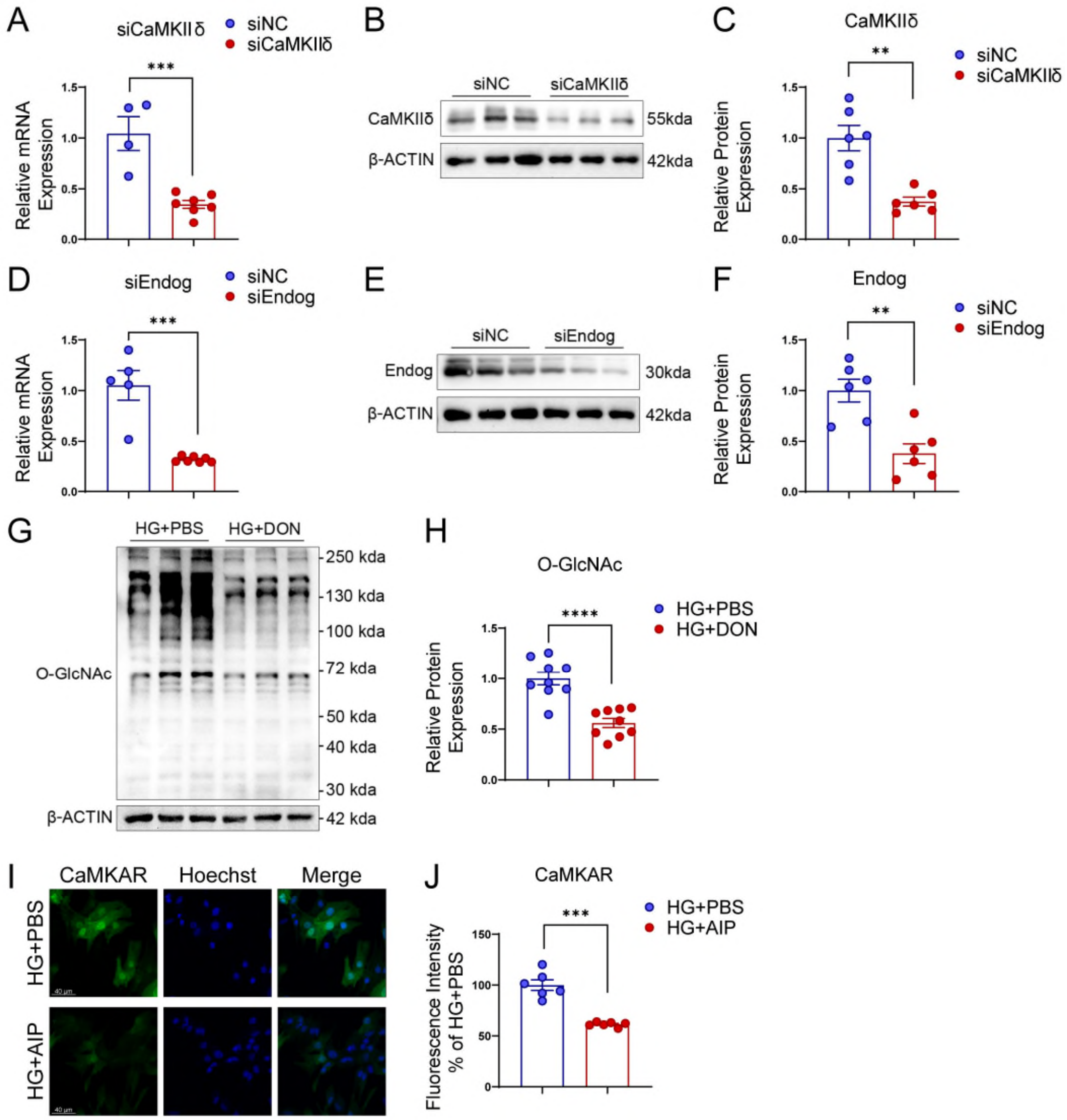

Supplementary Fig. 5: Evaluation of siRNA knockdown efficiency and inhibitor efficacy in NRCMs. Experiments were performed using NRCMs isolated from E19.5 rat hearts. CaMKIIδ siRNA and Endog siRNA were transfected into NRCMs for 48 hours. DON was applied on NRCMs at 50 μM for 48 hours, and AIP at 1 μM for 48 hours. (A) mRNA expression of CaMKIIδ after CaMKIIδ siRNA transfection, n = 4-7 per group. (B) Representative immunoblots showing CaMKIIδ protein levels after CaMKIIδ siRNA transfection. (C) Quantification of CaMKIIδ protein expression in panel B, n = 6 per group. (D) mRNA expression of Endog after Endog siRNA transfection, n = 5-7 per group. (E) Representative immunoblots showing Endog protein levels after *Endog* siRNA transfection. (F) Quantification of Endog protein expression in panel E, n = 6 per group. (G) Representative immunoblots of *O*-GlcNAcylation after DON treatment. (H) Quantification of *O*-GlcNAcylation levels in panel G, n = 9 per group. (I) Fluorescence images of NRCMs infected with CaMKAR for 24 hours and stained with Hoechst33342 for 20 minutes. Scale bar = 40 μm. (J) Quantification of fluorescence intensity in panel I, n = 6 per group. For statistical comparisons, we initially evaluated data for normality and equality of variances. Statistical significance was assessed using two-tailed unpaired Student's *t* test. If equal variances were not assumed, Welch's *t* test was applied. Data were expressed as mean±SEM, *p<0.05, **p<0.01, ***p<0.001, ****p<0.0001 vs. CON.

References:

1. Herzog, R. *et al.* Dynamic O-linked N-acetylglucosamine modification of proteins

affects stress responses and survival of mesothelial cells exposed to peritoneal dialysis fluids. *J Am Soc Nephrol* **25**, 2778-2788 (2014).

2. Erickson, J. R. *et al.* Diabetic hyperglycaemia activates CaMKII and arrhythmias by O-linked glycosylation. *Nature* **502**, 372-376 (2013).
3. Gonano, L. A. *et al.* Calcium-calmodulin kinase II mediates digitalis-induced arrhythmias. *Circ Arrhythm Electrophysiol* **4**, 947-957 (2011).
4. Trum, M. *et al.* Empagliflozin inhibits increased Na influx in atrial cardiomyocytes of patients with HFpEF. *Cardiovasc Res* **120**, 999-1010 (2024).
5. Sharma, N. S. *et al.* Targeting tumor-intrinsic hexosamine biosynthesis sensitizes pancreatic cancer to anti-PD1 therapy. *J Clin Invest* **130**, 451-465 (2020).
6. Daniels, L. J. *et al.* Inhibition of calcium/calmodulin-dependent kinase II restores contraction and relaxation in isolated cardiac muscle from type 2 diabetic rats. *Cardiovasc Diabetol* **17**, 89 (2018).
7. Chao, T. *et al.* Autophagy restricts mitochondrial DNA damage-induced release of ENDOG (endonuclease G) to regulate genome stability. *Autophagy* **17**, 3444-3460 (2021).

(Q3) Figure 6: Minimal description of the experimental settings, results and conclusions should be provided for each figure: e.g.: one single sentence "Co-transfecting Endog siRNA and Camk2d siRNA led to reduced mtDNA release and lower phosphorylation levels of inflammatory proteins" is supposed to describe a complex 6-panel figure (Fig. 6A-F). A minimal step-by-step description will help the demonstration. As it stands, the figures 6A-F are difficult to interpret for the reader.

Response:

This has been done. In response to your suggestions, we have revised the description for Figure 6 to provide a clearer and more detailed description of the experimental settings and results. We have included a step-by-step description to ensure that each panel (Fig. 6A-F) is properly interpreted in the revised Manuscript: "Meanwhile, co-transfecting *Endog* siRNA and a *Camk2d*-overexpressing plasmid in NRCMs resulted in a marked increase in mtDNA release from mitochondrial matrix (Fig. 6A-B). qPCR results showed that cytosolic mtDNA levels were increased after *Endog* siRNA transfection (Fig. 6C), consistent with the enlarged images shown in Fig. 6A. *Endog* siRNA transfection in NRCMs led to increased phosphorylation of STING, TBK1, and IRF3 (Fig. 6D), effectively recapitulating the activation of downstream signaling pathways observed in hyperglycemia. NRCMs co-transfected with *Endog* siRNA and Camk2d overexpression plasmid showed elevated phosphorylation levels of STING, TBK1, and IRF3 (Fig. 6E). In contrast, NRCMs with *Endog* siRNA and *Camk2d* siRNA exhibited decreased phosphorylation of STING, TBK1, and IRF3 (Fig. 6F)." (lines 322-331 of the revised manuscript)

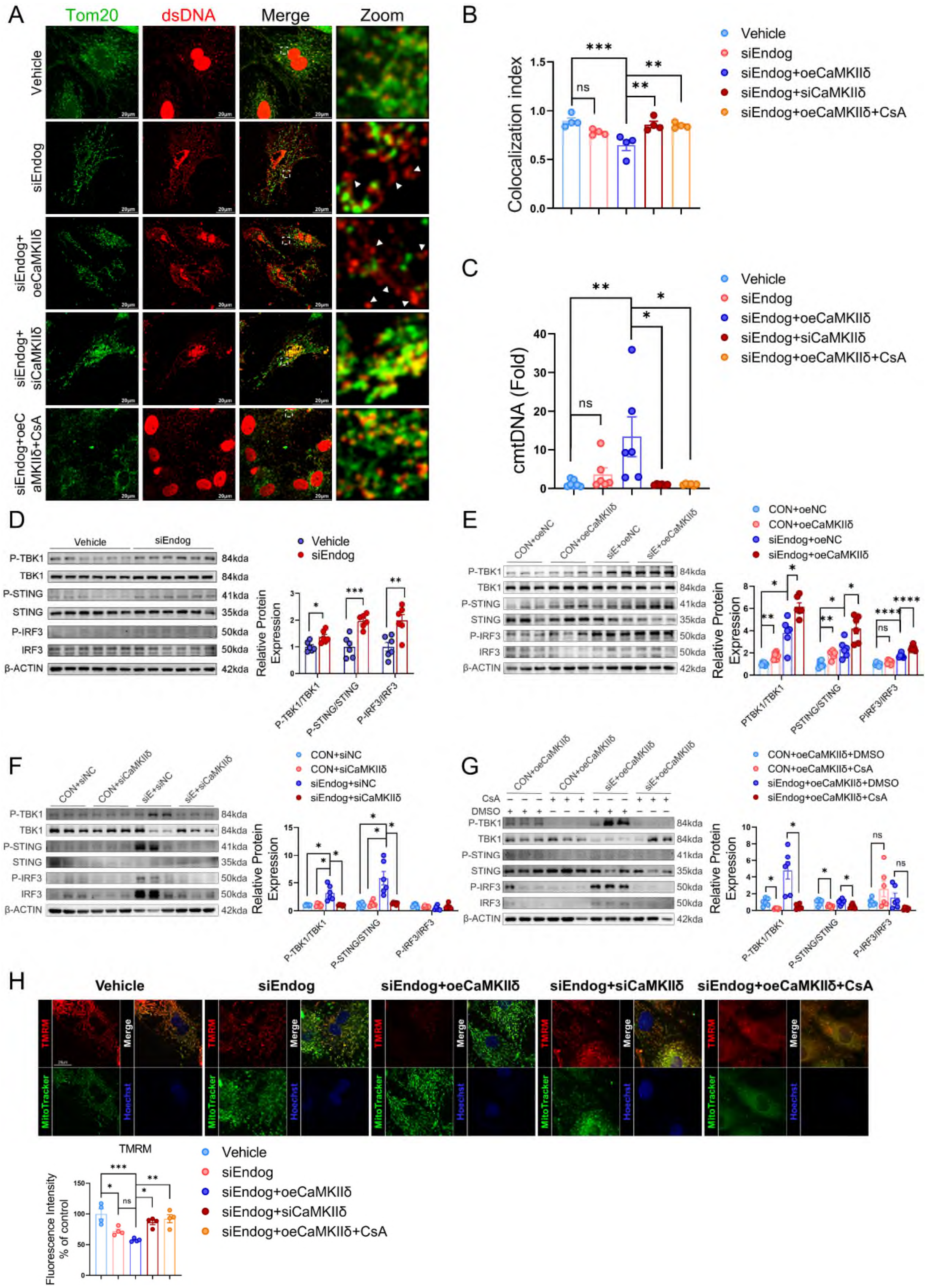

Figure 6. *O*-GlcNAcylation promoted mtDNA release and subsequent inflammatory response in hyperglycemia through CaMKIIδ regulating mPTP opening. Experiments were performed using NRCMs isolated from E19.5 rat hearts. CaMKIIδ siRNA or CaMKIIδ overexpression plasmid was transfected into NRCMs for 48 hours. CsA was applied on NRCMs at 10 μM for the final 4 hours. (A) Cardiomyocytes were stained with Tom20 (green) and dsDNA (red) in all four groups, scale bar = 20 μm. White arrows denoted mtDNA released from mitochondria. (B) The colocalization index of dsDNA and mitochondria in panel (A) was quantified based on Manders' coefficient index, n = 4 per group. (C)

Cytoplasmic mtDNA release was assessed by qPCR, n = 6-7 per group. (D-G) Representative immunoblots and analyses of phosphorylated and total protein levels in cardiomyocytes among multiple groups, n = 6 per group. (H) Representative fluorescence images of cardiomyocytes stained with TMRM, MitoTracker and Hoechst33342 for 20 mins (left), scale bar = 20 μm. The statistically quantified data on the fluorescence intensity (right), n = 4 per group. For statistical comparisons, we initially evaluated data for normality and equality of variances. Student's *t* test and one-way ANOVA were used to determine the levels of statistical differences between two groups and among multiple groups, respectively. If equal variances were not assumed, Welch's *t* test or Welch's ANOVA were applied. Data were expressed as mean±SEM, *p<0.05, **p<0.01, ***p<0.001, ****p<0.0001.

(Q4) While the text is generally well-written, several presentation issues dampen the clarity of the demonstration.

***Figures should be rearranged to appear in the order of their citation in the text (e.g. Fig.1C called before Fig1B, FigS3E-G called before FigS1 and S2, intertwined figure 5 and 6 etc….).**

***All Figures should be called in the text. (e.g. Fig S3A-D, Fig 7A-C, FigS4F-H do not appear to be "called" in the text).**

*** The "Methods" section is incomplete. it should describe the use of the chemical inhibitors and siRNA which are key to the authors' claims. Other methods are also missing (e.g.: Echocardiography…).**

<u>Response</u>:

Thank you for your positive comments that "<u>the text is generally well-written</u>". Following your suggestion, we have made the following detailed revisions on the presentation issues:

1) We have reorganized the **Results** section and rearranged the figures to ensure that the order of the text and figures follows a top-down sequence;

2) We have provided detailed descriptions for each figure in the revised Manuscript. For example (inserted below for the Reviewer's convenience):

"<u>The elevated cmtDNA levels observed in the hearts of HG group were a consequence of the release of dsDNA fragments from the mitochondrial matrix to the cytoplasm (Supplementary Fig. 3A). Although total mRNA and protein levels remained unchanged, phosphorylation of STING, TBK1 (TANK-binding kinase 1), and IRF3 (interferon regulatory factor 3) proteins increased in the HG group (Supplementary Fig. 3B-C). This phosphorylation was effectively suppressed by AIP or DON treatment (Fig. 4D). In addition, mRNA expression levels of several inflammation-related genes were consistently elevated in the HG group (Supplementary Fig. 3D).</u>" (Supplementary Fig. 3A-D, lines 276-283 of the revised manuscript)

"<u>Crucially, echocardiography showed that DON intervention effectively restored cardiac function of EF%, FS%, and CO in both the CON+LPS and HG+LPS groups (Fig. 7A and C). Concurrently, DON administration mitigated offspring cardiac remodeling. WGA staining revealed attenuated cardiomyocyte enlargement in LPS-challenged hyperglycemic offspring (Fig. 7B and D).</u>" (Fig. 7A-D, lines 412-416 of the revised manuscript)

"<u>Plasma mtDNA copy number was increased in the HG+LPS group compared to either the HG+PBS or CON+LPS groups, indicating an additive effect of hyperglycemia and LPS on mtDNA release (Supplementary Fig. 4G). Western blotting analysis revealed markedly enhanced phosphorylation of STING, TBK1, and IRF3 proteins in the HG+LPS group</u>

(Supplementary Fig. 4H). In line with these findings, mRNA expression of inflammatory genes, including *Usp18*, *Isg15*, *Ifit1*, *Ifit3*, *Cxcl10*, *Ifi44*, *Iigp1*, and *Rsad2*, was further upregulated in hyperglycemic offspring treated with LPS compared to either condition alone (Supplementary Fig. 4I)." (Supplementary Fig. 4F-I, lines 402-409 of the revised manuscript)

3) We have now included a more detail description of the use of the chemical inhibitors and siRNA, and included other methods including echocardiography. We have added the following statement in the **Materials and Methods** section of the revised Manuscript: "Following cell attachment, the inhibitors were added directly to the culture medium and maintained. NRCMs were treated with the CaMKIIδ inhibitor AIP (1 μM; HY-P0214, MedChemExpress, China) or the *O*-GlcNAcylation inhibitor DON (50 μM; S8620, Selleck, USA) for 48 hours prior to harvest[1-3]. CsA (10 μM; HY-B0579, MedChemExpress, China) was added to the culture medium for the final 4 hours prior to sample collection[4].

For siRNA or plasmid transfection, transfection procedures were performed according to the manufacturer's instructions. For siRNA transfection, RiboFECT™ CP Transfection Kit (R10035.5; RiboBio, China) was used. For plasmid transfection, Lipofectamine™ 3000 Reagent (L3000015; Thermo Fisher Scientific, USA) was used." (lines 612-620 of the revised manuscript). Details of the methods are now available in the **Supplemental Information**.

References:

1. Erickson, J. R. *et al.* Diabetic hyperglycaemia activates CaMKII and arrhythmias by O-linked glycosylation. *Nature* **502**, 372-376 (2013).
2. Sharma, N. S. *et al.* Targeting tumor-intrinsic hexosamine biosynthesis sensitizes pancreatic cancer to anti-PD1 therapy. *J Clin Invest* **130**, 451-465 (2020).
3. Daniels, L. J. *et al.* Inhibition of calcium/calmodulin-dependent kinase II restores contraction and relaxation in isolated cardiac muscle from type 2 diabetic rats. *Cardiovasc Diabetol* **17**, 89 (2018).
4. Chao, T. *et al.* Autophagy restricts mitochondrial DNA damage-induced release of ENDOG (endonuclease G) to regulate genome stability. *Autophagy* **17**, 3444-3460 (2021).

Others points

(Q1) The authors should clarify the cell type used in the different experiments as there are some confusions between the use of primary fetal heart (e19.5?), primary neonate heart (P?) and cultured neonatal rat cardiomyocytes (NRCM)

Response:

We apologize for the confusion. In the revised manuscript, we have carefully clarified the specific cell sources and stages in each figure legend and in the **Results** section. For example, "Fetal rats at embryonic day 19.5 (E19.5) were used for the experiments shown in Fig. 1B-F and Fig. 1J. NRCMs isolated from E19.5 fetal hearts were used in Fig. 1G-I and K-L." (legends of Fig. 1, lines 958-960 of the revised manuscript)

(Q2) line 354: "Additionally, placental weight was significantly higher in the HG group (Fig1D). HG fetuses exhibited lower body weight compared to CON fetuses

accompanied by an increased ratio of placenta weight to body weight". According to the figure, these statements are not correct.

Response:

We apologize for the confusion. Following your suggestion, we have modified this statement, also inserted below: "In offspring, hyperglycemic exposure caused fetal growth restriction with reduced body weight, increased placental weight, and elevated blood glucose levels (Fig. 1D)." (lines 137-138 of the revised manuscript)

(Q3) Line 369: "Results showed significantly decreased mitochondrial membrane potential in HG group cardiomyocytes compared to CON group". Quantification should be provided to assess significance.

Response:

We appreciate your constructive suggestions. In response, we have added the quantitative data of mitochondrial membrane potential (now presented as Fig.1I, lines 956-980 of the revised manuscript; inserted below for the Reviewer's convenience)

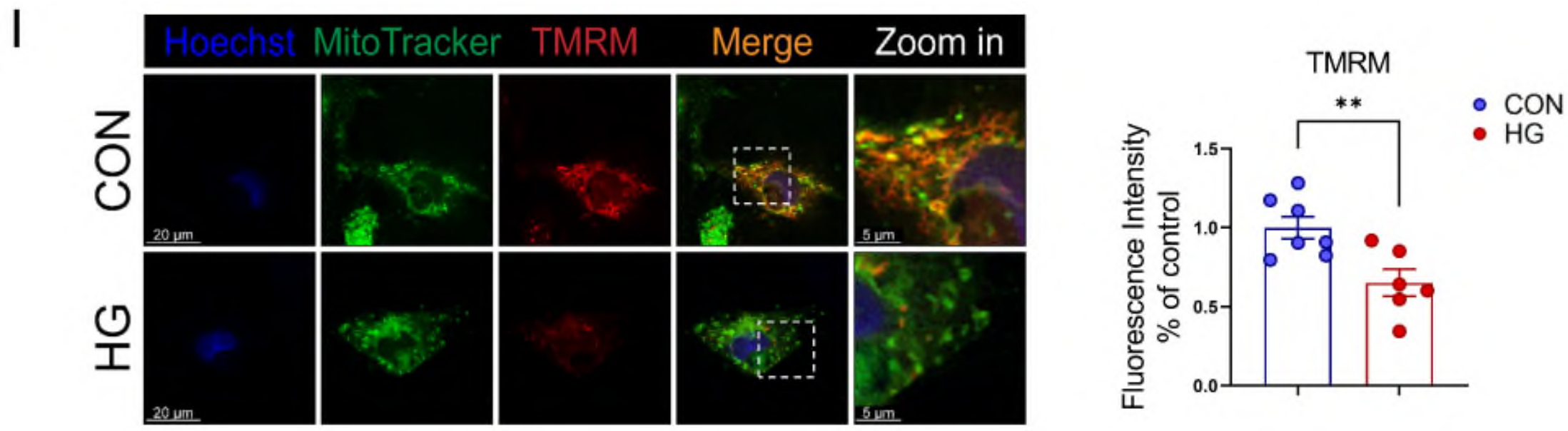

Fig. 1I: Assessment of mitochondrial membrane potential in NRCMs. Experiments were performed using NRCMs isolated from E19.5 rat hearts. (I) Fluorescence images of cardiomyocytes stained with MitoTracker (green), TMRM (red), and Hoechst (blue), scale bar = 20 μm (left). Quantification of TMRM fluorescence intensity in cardiomyocytes, n = 6-7 per group (right). For statistical comparisons, we initially evaluated data for normality and equality of variances. Statistical significance was assessed using two-tailed unpaired Student's *t* test. If equal variances were not assumed, Welch's *t* test was applied. Data were expressed as mean±SEM, *p<0.05, **p<0.01, ***p<0.001, ****p<0.0001 vs. CON.

(Q4) The use of DON and AIP as a *O*-GlcNAcylation and CAMKIIδ inhibitors should be defined at first use (currently line 408)

Response:

This has been done. Following your suggestion, we have now clearly defined both abbreviations upon their initial introduction:

1) "Then, coimmunoprecipitation (CoIP) experiments confirmed that elevated *O*-GlcNAcylation levels of CaMKIIδ in HG group compared to CON group, which was reduced by 6-Diazo-5-oxo-L-norleucine (DON, an inhibitor of *O*-GlcNAcylation) treatment (Fig. 2D)." (lines 201-204 of the revised manuscript)

2) "Here, we evaluated the effects of CaMKIIδ *via* applying autocamtide-2-related inhibitory peptide (AIP, a CaMKIIδ inhibitor) and *O*-GlcNAcylation inhibitor DON." (lines 242-244 of the revised manuscript)

(Q5) The choice of the LPS treatment used in figure 7 and S4 should be explained. *In vivo* DON treatment should be described (duration…).

Response:

We appreciate your constructive suggestions. The rationale for using LPS in Figure 7 and Supplementary Fig. 4 was to model the physiological postnatal exposure to microbial components and infection-related stress that all neonates typically face after birth[1,2]. Notably, studies have demonstrated that neonates born to mothers with GDM exhibit a higher susceptibility to infections[3-6]. This increased vulnerability is thought to result from impaired immune competence due to metabolic disturbances, including hyperglycemia[7-9]. Therefore, in order to simulate the clinical context in which both normal and GDM-exposed neonates encounter postnatal microbial challenges, a single intraperitoneal injection of LPS[10-12] was administered. This model enabled us to assess whether offspring subjected to intrauterine hyperglycemia exhibit an exaggerated myocardial response to inflammatory stimuli, thereby revealing the potential interaction between prenatal metabolic insults and postnatal immune stress.

Additionally, we have supplemented the description of the *in vivo* DON treatment protocol, including detailed information on the dosage (DON: 1 mg/kg; LPS: 2 mg/kg), frequency, and duration of administration, in the revised **Methods** section: "Pharmacologic treatment: To evaluate the phenotypic response of offspring to postnatal infection, some offspring from the CON and HG groups received a single intraperitoneal injection of LPS (2 mg/kg; Sigma, USA) or PBS on postnatal day 7, according to previously published methods[10,13,14]. Cardiac function was assessed by echocardiography 6 hours after injection, followed by tissue collection[10,12,14]. To assess the protective effects of pharmacologic intervention, some offspring from both the CON and HG groups received daily intraperitoneal injections of DON (1 mg/kg/day; MCE, China) or PBS from postnatal day 3 to day 7[15-18]." (lines 580-587 of the revised manuscript)

References:

1. Torow, N., Marsland, B. J., Hornef, M. W. & Gollwitzer, E. S. Neonatal mucosal immunology. *Mucosal Immunol* **10**, 5-17 (2017).
2. Tamburini, S., Shen, N., Wu, H. C. & Clemente, J. C. The microbiome in early life: implications for health outcomes. *Nat Med* **22**, 713-722 (2016).
3. Liu, J. *et al.* Gestational diabetes exacerbates intrauterine microbial exposure induced intestinal microbiota change in offspring contributing to increased immune response. *Nutr Diabetes* **14**, 87 (2024).
4. Liu, C. *et al.* Multiple perinatal characteristics affect the association between maternal diabetes status and early neonatal gut microbiota. *mSphere* **10**, e0091424 (2025).
5. Al-Shahrani, A. M. Predictors of neonatal intensive care unit admission and adverse outcomes related to gestational diabetes. *Cureus* **15**, e38579 (2023).
6. Wang, B. B. & Xue, M. Early neonatal complications in pregnant women with gestational diabetes mellitus and the effects of glycemic control on neonatal infection. *World J Diabetes* **14**, 1393-1402 (2023).
7. Shao, W. *et al.* Understanding the link between different types of maternal diabetes and the onset of autism spectrum disorders. *Diabetes Metab* **50**, 101543 (2024).
8. Jimenez-Escutia, R. *et al.* High glucose promotes inflammation and weakens placental defenses against E. coli and S. agalactiae infection: Protective role of insulin and

metformin. *Int J Mol Sci* **24** (2023).

9. Xiong, Y. *et al.* Aberrant NK cell profile in gestational diabetes mellitus with fetal growth restriction. *Front Immunol* **15**, 1346231 (2024).
10. Planavila, A. *et al.* Fibroblast growth factor 21 protects the heart from oxidative stress. *Cardiovasc Res* **106**, 19-31 (2015).
11. Sun, Y. *et al.* Beclin-1-dependent autophagy protects the heart during sepsis. *Circulation* **138**, 2247-2262 (2018).
12. Wang, Y. *et al.* beta(1)-adrenoceptor stimulation promotes LPS-induced cardiomyocyte apoptosis through activating PKA and enhancing CaMKII and IkappaBalpha phosphorylation. *Crit Care* **19**, 76 (2015).
13. Kaizaki, A. *et al.* Celecoxib reduces brain dopaminergic neuronaldysfunction, and improves sensorimotor behavioral performance in neonatal rats exposed to systemic lipopolysaccharide. *J Neuroinflammation* **10**, 45 (2013).
14. Cuskelly, A. *et al.* Neonatal immune challenge influences the microbiota and behaviour in a sexually dimorphic manner. *Brain Behav Immun* **103**, 232-242 (2022).
15. Xiang, J. *et al.* Gluconeogenic enzyme PCK1 deficiency promotes CHK2 O-GlcNAcylation and hepatocellular carcinoma growth upon glucose deprivation. *J Clin Invest* **131** (2021).
16. Erickson, J. R. *et al.* Diabetic hyperglycaemia activates CaMKII and arrhythmias by O-linked glycosylation. *Nature* **502**, 372-376 (2013).
17. Chen, G. *et al.* Glutamine antagonist synergizes with electrodynamic therapy to induce tumor regression and systemic antitumor immunity. *ACS Nano* **16**, 951-962 (2022).
18. Rais, R. *et al.* Discovery of DRP-104, a tumor-targeted metabolic inhibitor prodrug. *Sci Adv* **8**, eabq5925 (2022).

(Q6) Figure 5K: oeCAMK2D should read oeCAMKIIδ

<u>Response</u>:

Following your suggestion, we have corrected “oeCAMK2D” to “oeCaMKIIδ” in Figure 5K, and carefully checked the manuscript on this issue (inserted below for the Reviewer’s convenience).

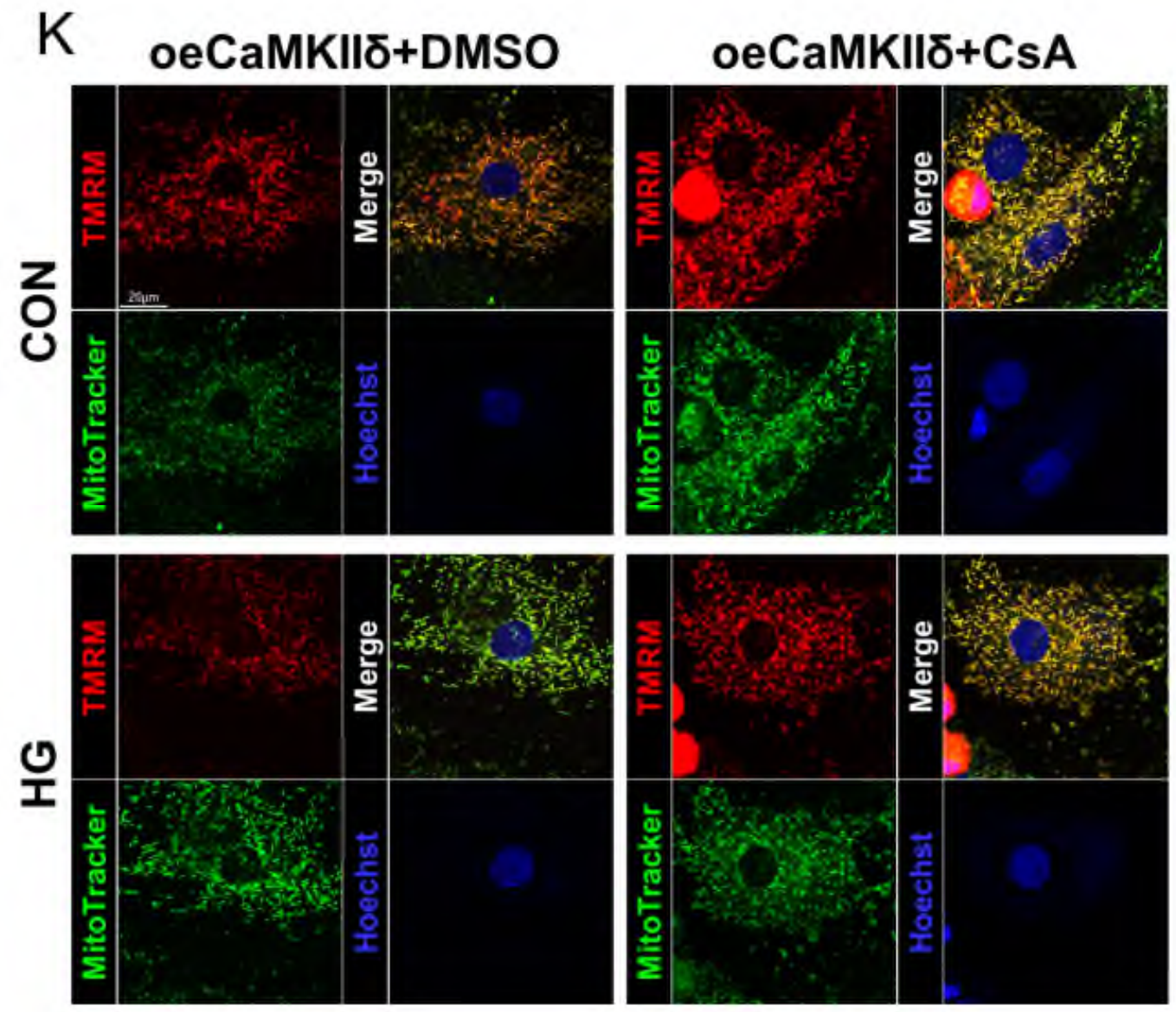

Figure 5K: Fluorescence images of cardiomyocytes stained with TMRM, MitoTracker and Hoechst33342 for 20 mins, scale bar = 20 μm.

(Q7) Echocardiography results shown in figure 7A and S4A are too small to be readable in the figure.

<u>Response</u>:

Following your suggestion, we have separated the echocardiography images in Fig. 7A and Supplementary Fig. S4A and enlarged them to improve readability. (inserted below for the Reviewer's convenience)

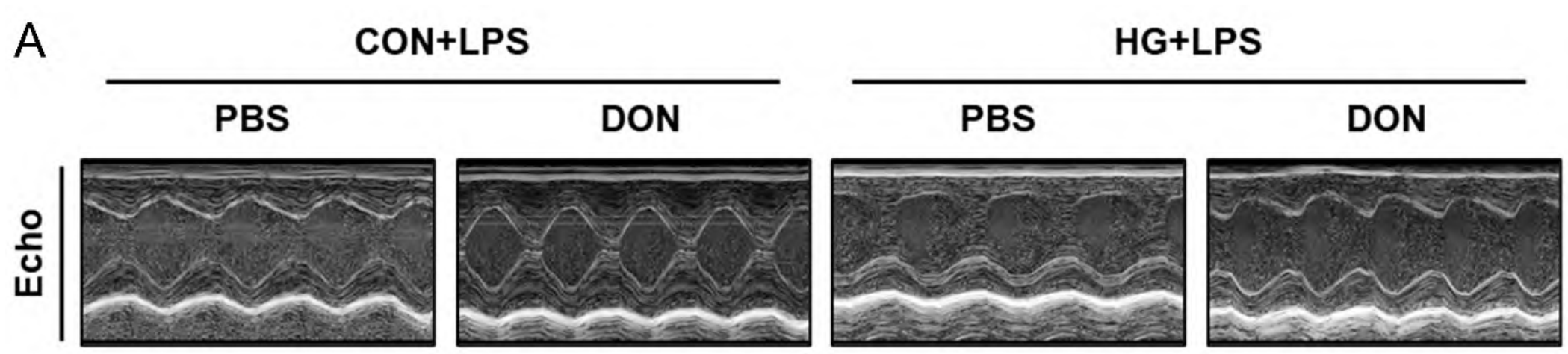

Figure 7A: Echocardiography analyses were conducted for multiple groups of fetal hearts. Neonatal rats received daily intraperitoneal injections of either PBS or DON (1 mg/kg/day) from postnatal day 3 to day 7, followed by a single LPS injection (2 mg/kg, i.p.) on day 7.

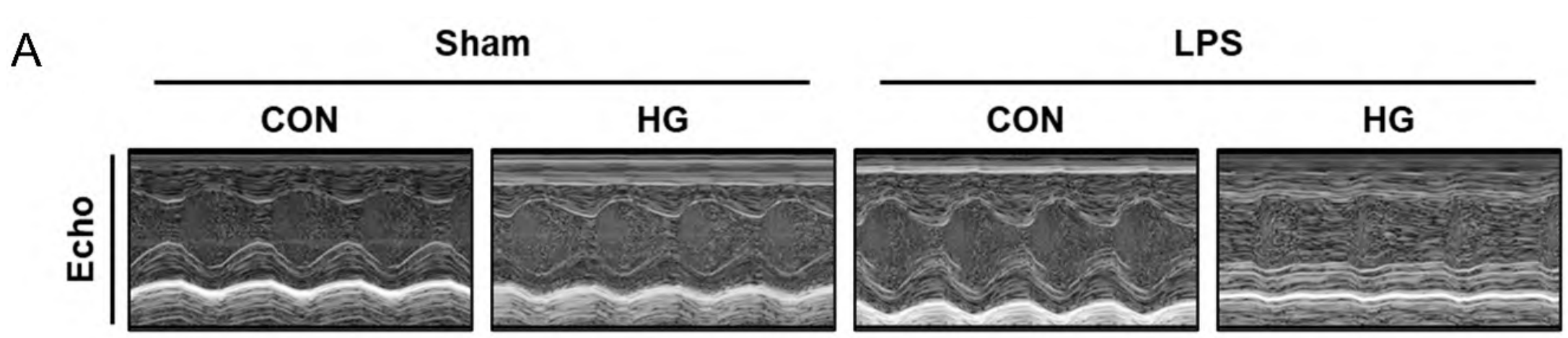

Supplementary Fig. 4: Echocardiography analyses were conducted for multiple groups of fetal hearts. Neonatal rats received a single LPS injection (2 mg/kg, i.p.) on postnatal day 7 (P7).

(Q8) Line 555: sentence to be revised "Environmental alterations during critical developmental periods impact growth PTM modification, an essential determinant of the onset caused by GDM".

Response:

Following your suggestion, the sentence has been revised to: "Environmental alterations during critical developmental windows influence PTM modifications, which are essential determinants of the abnormalities induced by GDM[1]." (lines 467-468 in the revised Manuscript)

References:

1. Eriksson, J. G. Developmental origins of health and disease - from a small body size at birth to epigenetics. *Annals of Medicine* **48**, 456 - 467 (2016).

Detailed Responses to Reviewer #2:

General Comments:

This manuscript by Xiao and colleagues investigates the mechanism by which intrauterine hyperglycemia related to maternal diabetes mellitus (specifically preexisting maternal diabetes mellitus) leads to pathologic fetal cardiac remodeling. *O*-GlcNAcylation is a nutrient and stress post-translational modification (PTM) that involves the addition of O-Linked β-N-acetylglucosamine (*O*-GlcNAc) to intracellular proteins that plays a critical role in physiology and pathology, especially in conditions of hyperglycemia such as diabetes mellitus. CaMKII, a protein kinase which is well established as an intracellular signaling node for cardiac disease, was previously shown to undergo *O*-GlcNAcylation and causally linked to cardiac disease in the context of diabetes mellitus.

This study employed a streptozocin (STZ) induced rat model of diabetes mellitus/intrauterine hyperglycemia. Data presented in this manuscript are interpreted to show fetal myocardium in STZ treated dams have elevated *O*-GlcNAc and enhanced *O*-GlcNAcylation of CaMKII. This is associated with disordered intracellular Ca2+ handling, elevated ROS, mitochondrial dysfunction and increased mtDNA release leading to activation of the cGAS-STING pathway and inflammation. Inhibition of *O*-GlcNAcylation (using DON) or CaMKII (using AIP) effectively attenuated this pathologic response. The strength of this study is the identification of a novel link between CaMKIIδ/mtDNA/cGAS-STING signaling in hyperglycemia dependent mtDNA activation of fetal cardiac remodeling in maternal diabetes mellitus. Also, the study raises the potential of plasma mtDNA as a biomarker for fetal/neonatal cardiac inflammatory injury.

Response:

Thank you for the positive comments that "The strength of this study is the identification of a novel link between CaMKIIδ/mtDNA/cGAS-STING signaling in hyperglycemia dependent mtDNA activation of fetal cardiac remodeling in maternal diabetes mellitus. Also, the study raises the potential of plasma mtDNA as a biomarker for fetal/neonatal cardiac inflammatory injury." We have followed your suggestions and made every possible effort to address the concerns. Detailed responses to all further comments are provided below.

General Comments:

However, the conclusions about *O*-GlcNAcylation of CaMKIIδ in this study is overstated and not convincingly supported by the data presented. The use of DON alone is insufficient to prove the role of *O*-GlcNAcylation in driving the hypothesized pathway. Further, none of the established CaMKIIδ PTMs, including *O*-GlcNAcylation (S280), autophosphorylation (T287) and oxidation (MM281/282), were directly tested. Instead, all data rely on CaMKII inhibition (with AIP or siRNA) or over-expression. Knockin mice are available to the field for each of these post-translational modifications on the appropriate CaMKIId background.

Response:

Thanks for your constructive comments. According to your suggestions, to further confirm the role of CaMKIIδ *O*-GlcNAcylation in intrauterine hyperglycemia, we performed a series of additional experiments as follows:

1) To rigorously evaluate CaMKIIδ PTMs in the context of intrauterine hyperglycemia, we performed western blotting experiments on cardiac tissues from offspring in both CON and HG groups. Using site-specific antibodies, we quantified: i) autophosphorylation at T287, ii) oxidation at Met281/282. Our data identified that autophosphorylation (T287) of CaMKIIδ was upregulated in the HG group (Supplementary Fig. 6D and F), while oxidation (Met281/282) showed no significant differences between groups (Supplementary Fig. 6D-E).

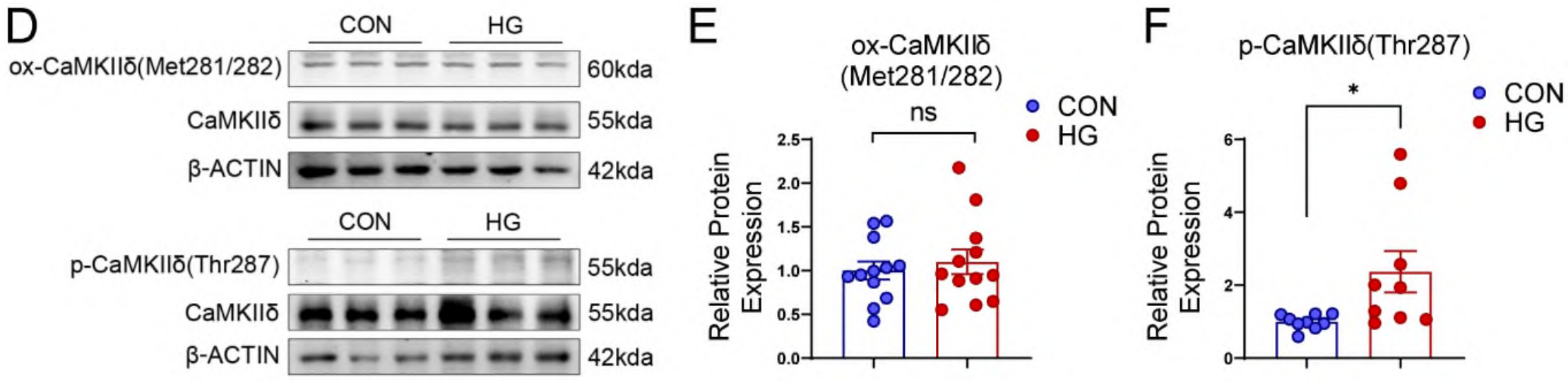

Supplementary Fig. 6D-F: Post-translational modifications of CaMKIIδ in hyperglycemia offspring. Experiments were performed using E19.5 rat hearts. (D) Representative immunoblots showing CaMKIIδ oxidation (upper) and autophosphorylation (lower) in the CON and HG groups. (E) Quantification of CaMKIIδ oxidation levels, n = 12 per group. (F) Quantification of CaMKIIδ autophosphorylation levels, n = 9 per group. For statistical comparisons, we initially evaluated data for normality and equality of variances. Statistical significance was assessed using two-tailed unpaired Student's *t* test. If equal variances were not assumed, Welch's *t* test was applied. Data were expressed as mean±SEM, **p*<0.05, ***p*<0.01, ****p*<0.001, *****p*<0.0001 vs. CON.

2) Following your suggestions, we revised Figures 2D and 2E with more representative images and conducted further statistical analysis of the data to confirm the occurrence of CaMKIIδ *O*-GlcNAcylation in fetal cardiomyocytes exposed to in utero hyperglycemia, as well as the inhibitory effects of AIP and DON on CaMKIIδ *O*-GlcNAcylation. The results demonstrated a significant increase in CaMKII *O*-GlcNAcylation in NRCMs from the HG+PBS group, which was effectively attenuated by DON treatment (Figure 2D and 2E in the revised Manuscript, inserted below for the Reviewer's convenience).

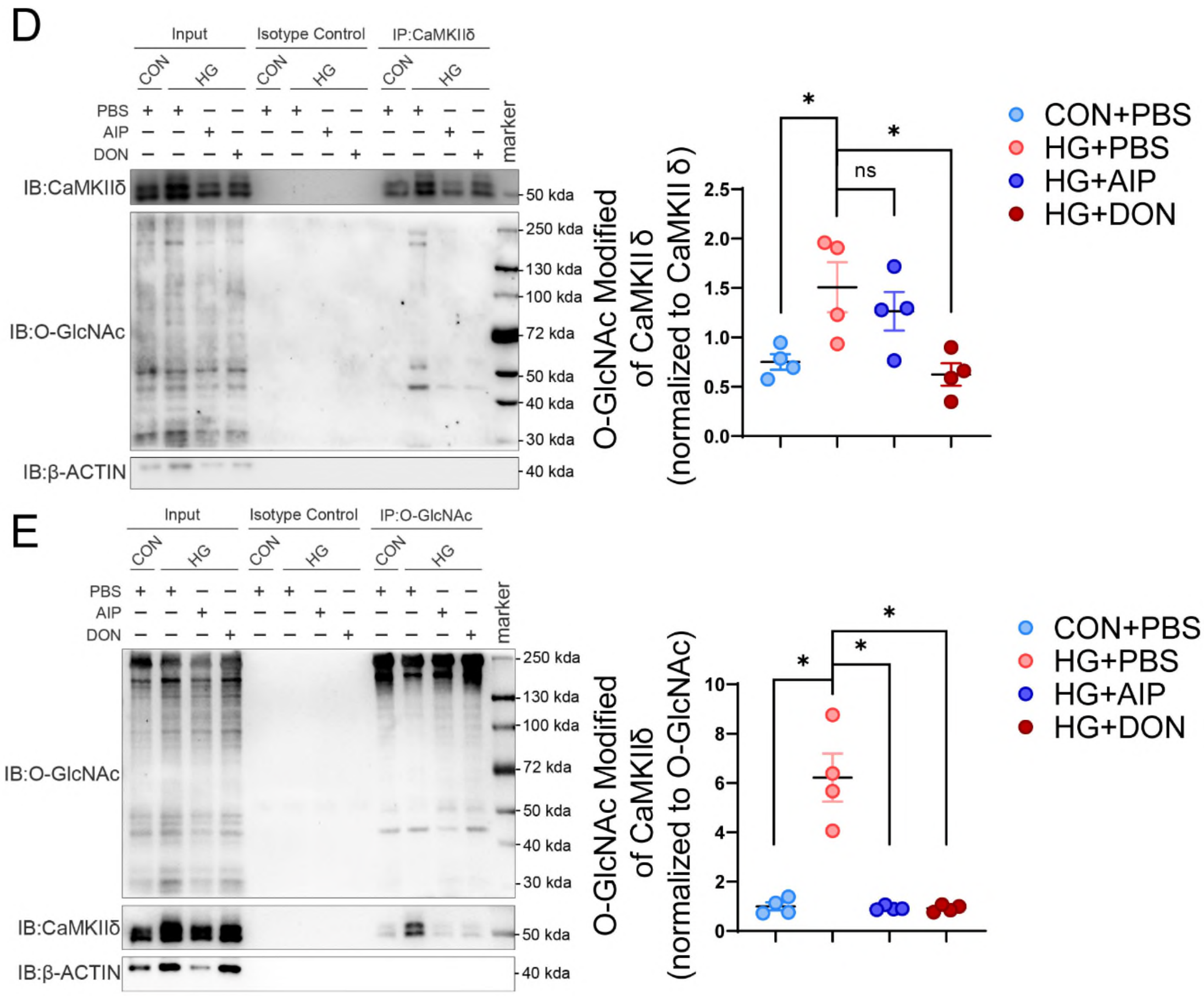

Figure 2D and 2E: CoIP western blotting and analysis of CaMKIIδ *O*-GlcNAcylation. CoIP experiments were performed using NRCMs isolated from E19.5 neonatal hearts, DON was applied on NRCMs at 50 μM for 48 hours, and AIP at 1 μM for 48 hours. (D) Western blotting of CoIP were performed for the CON+PBS, HG+PBS, HG+AIP, HG+DON groups (left). IP: CaMKIIδ. Statistical quantification of the blots (right), n = 4 per group. (E) Western blotting of CoIP were performed for the CON+PBS, HG+PBS, HG+AIP, HG+DON groups (left). IP: *O*-GlcNAc. Statistical quantification of the blots (right), n = 4 per group. For statistical comparisons, data were initially evaluated for normality and equality of variances. One-way analysis of variance (ANOVA) was used to determine statistical differences among multiple groups. If equal variances were not assumed, Welch's ANOVA was applied. Data were expressed as mean±SEM, *$p<0.05$, **$p<0.01$, ***$p<0.001$, ****$p<0.0001$. Abbreviations: IP, immunoprecipitation; IB, immunoblotting.

3) Based on the results of above experiments, we excluded the possibility of CaMKII oxidation at Met281/282, and found that the HG group exhibited increased *O*-GlcNAcylation of CaMKIIδ accompanied by enhanced autophosphorylation at T287 (Thanks for your suggestion, which helped us make this finding). We fully agree with your point that "The use of DON alone is insufficient to prove the role of *O*-GlcNAcylation in driving the hypothesized pathway". Next, to validate the role of CaMKIIδ *O*-GlcNAcylation(S280) *in vivo*, we engineered two cardiac-specific adenoviral vectors to dissect the functional impact of CaMKIIδ *O*-GlcNAcylation(S280):

i) pADV-cTNT-Camk2d (p.S280A)-HA (abbreviated as AdV-CaMKIIδ-S280A-OE): To preserve kinase activity while blocking hyperglycemia-induced post-translational modification, we overexpressed a CaMKIIδ mutant with abolished *O*-GlcNAcylation at Ser280 (S280A mutation).

ii) pADV-cTNT-mCherry-3xFLAG-shCamk2d (abbreviated as AdV-shCaMKIIδ): To

isolate the effects of *O*-GlcNAcylation from baseline CaMKIIδ signaling, we knocked down endogenous CaMKIIδ (58% reduction in protein levels, $p = 0.0004$, Supplementary Fig. S9E in the revised Manuscript, which is inserted below for the Reviewer's convenience)

Simultaneous intramyocardial injection of these two adenoviral vectors into neonatal rat hearts resulted in a specific reduction of CaMKIIδ *O*-GlcNAcylation in *utero* hyperglycemia.

To verify changes in CaMKIIδ *O*-GlcNAcylation(S280) following *in vivo* gene interventions, we isolated NRCMs from hearts injected with adenoviral vectors and performed CoIP experiments. Additional CoIP results further support CaMKIIδ *O*-GlcNAcylation(S280) in *utero* hyperglycemia. As shown in Supplementary Fig. 9, overexpression of the CaMKIIδ S280A mutant effectively reduced cardiac CaMKIIδ *O*-GlcNAcylation in offspring subjected to intrauterine hyperglycemia. Moreover, the S280A mutant reduced CaMKIIδ activation in response to intrauterine hyperglycemia (inserted below for the Reviewer's convenience).

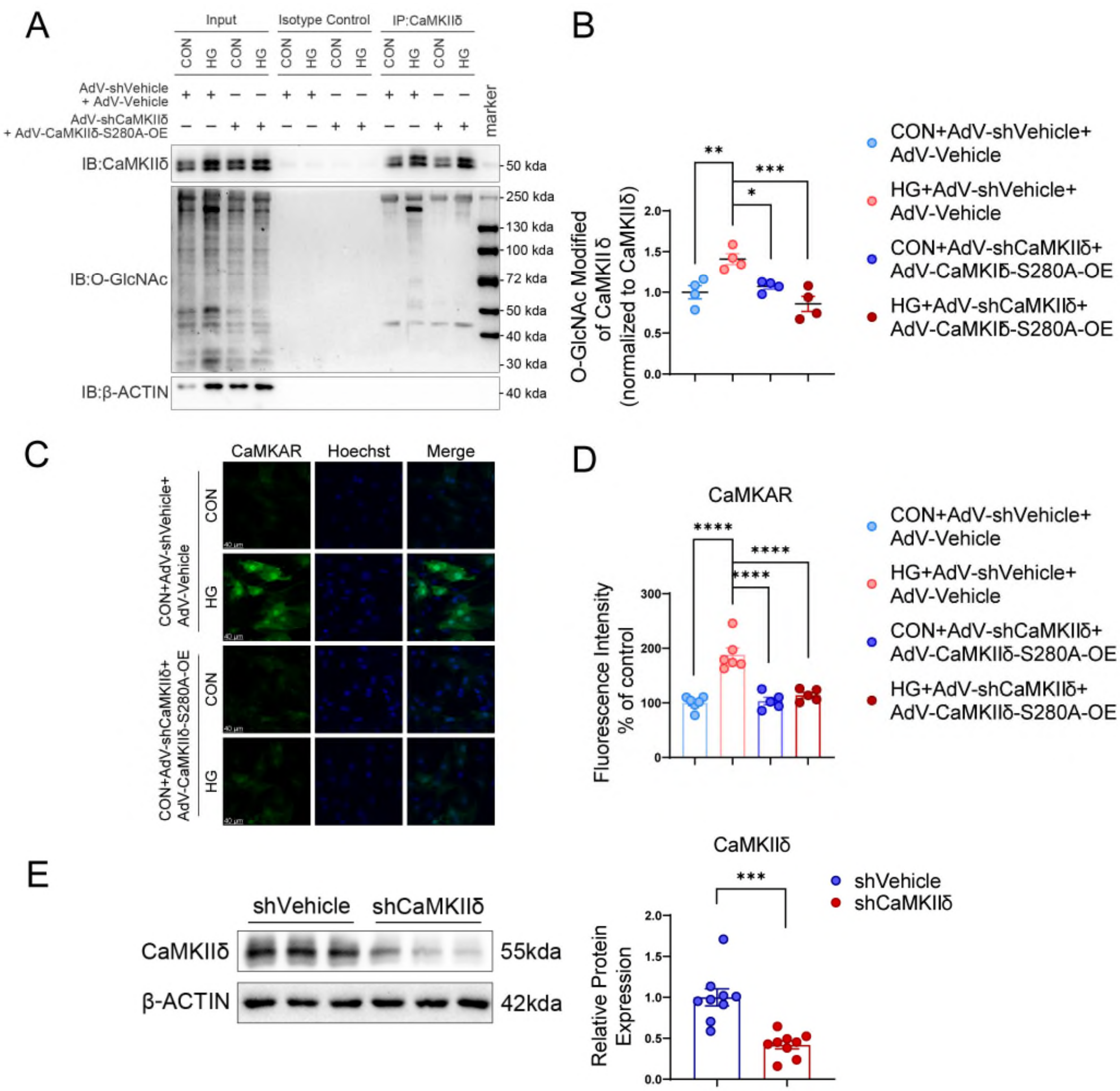

Supplementary Fig. 9: S280A mutation of CaMKIIδ abolished cardiac CaMKIIδ *O*-GlcNAcylation and reduced CaMKIIδ activation. Neonatal rats received injections on postnatal day 3 and were euthanized on postnatal day 7. CoIP experiments were performed using heart tissues from P7 neonatal rats. CaMKAR fluorescence imaging experiments were conducted using NRCMs isolated from P7

neonatal hearts. (A) Western blotting analysis of CoIP were measured for the CON+AdV-shVehicle+AdV-Vehicle, HG+AdV-shVehicle+AdV-Vehicle, CON+AdV-shCaMKIIδ+AdV-CaMKIIδ-S280A-OE, HG+AdV-shCaMKIIδ+AdV-CaMKIIδ-S280A-OE groups, IP: CaMKIIδ, n = 4 per group. (B) The statistically quantified data on the western blotting analysis of *O*-GlcNAc modified CaMKIIδ. (C) Fluorescence images of cardiomyocytes infected with CaMKAR for 24 hours and stained with Hoechst 33342 for 20 min, scale bar = 40 μm. (D) The statistically quantified data on the cellular fluorescence intensity after infected with CaMKAR, n = 5-6 per group. (E) Statistical quantification of CaMKIIδ protein levels following injection of AdV-shCaMKIIδ, n = 9 per group. For statistical comparisons, data were initially evaluated for normality and equality of variances. Student's *t* test and one-way ANOVA were used to determine the levels of statistical differences between two groups and among multiple groups, respectively. If equal variances were not assumed, Welch's *t* test or Welch's ANOVA were applied. Data were expressed as mean±SEM, *p<0.05, **p<0.01, ***p<0.001, ****p<0.0001. Abbreviations: IP, immunoprecipitation; IB, immunoblotting.

4) To investigate the *in vivo* changes in cardiac function following AdV-CaMKIIδ-S280A overexpression, we conducted a series of histological analyses and western blotting experiments. As shown in Supplementary Fig. 10, overexpression of the CaMKIIδ S280A mutant effectively attenuated myocardial fibrosis in offspring subjected to intrauterine hyperglycemia. Moreover, the S280A mutant reduced cardiomyocyte cross-sectional area in the HG group. Additionally, the expression levels of ANP and BNP were decreased in the HG+AdV-shCaMKIIδ+AdV-CaMKIIδ-S280A-OE group compared to the HG+AdV-shVehicle+AdV-Vehicle group. Notably, plasma mtDNA levels were also reduced in the HG+AdV-shCaMKIIδ+AdV-CaMKIIδ-S280A-OE group (inserted below for the Reviewer's convenience). We hope that these additional experiments have strengthened our manuscript and addressed your concerns.

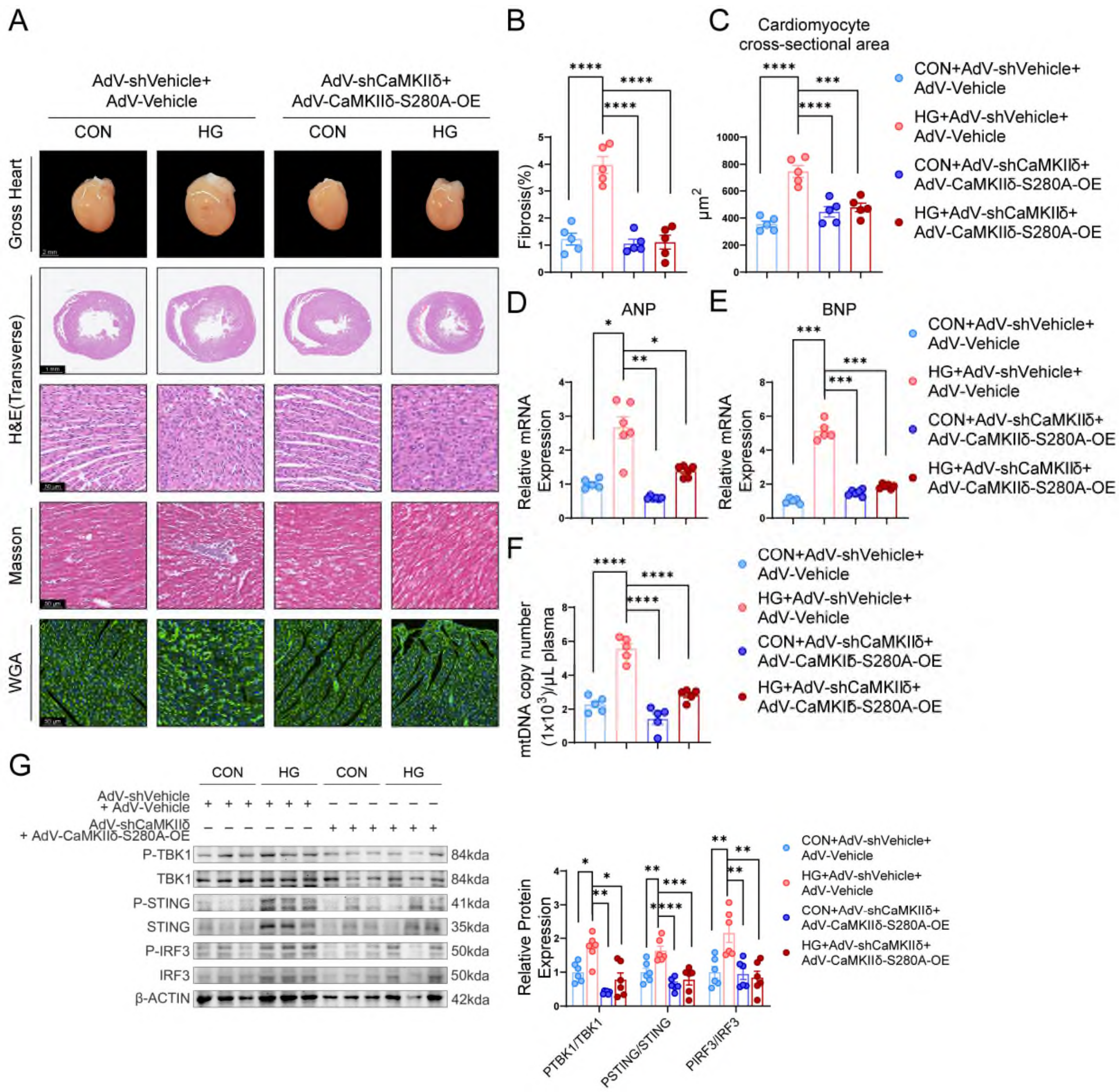

Supplementary Fig. 10: S280A mutation of CaMKIIδ abolishes cardiac CaMKIIδ *O*-GlcNAcylation in offspring subjected to intrauterine hyperglycemia. Neonatal rats received injections on postnatal day 3 and were euthanized on postnatal day 7. All experiments in Supplementary Fig. 10 were performed using heart tissues from P7 neonatal rats, except for the plasma mtDNA copy number experiment. (A) Echocardiography, gross morphology and histological analyses were conducted for multiple groups of fetal hearts. Gross morphology, scale bar = 2.0 mm; HE, Masson and WGA staining, scale bar = 50 μm. (B) Quantitative analysis was performed on fibrotic area (Masson staining), n = 5 per group. (C) Quantitative analysis was performed on cardiomyocyte cross-sectional area (WGA staining), n = 5 per group. (D) ANP gene expression was quantified by RT-qPCR, n = 5-6 per group. (E) BNP gene expression was quantified by RT-qPCR, n = 5-6 per group. (F) Plasma mtDNA copy number was assessed, n = 5 per group. (G) Phosphorylated and total protein levels in hearts from multiple groups were detected by western blotting (left). Densitometric quantification of the blots (right), n = 6 per group. For statistical comparisons, data were initially evaluated for normality and equality of variances. One-way ANOVA was used to determine statistical differences among multiple groups. If equal variances were not assumed, Welch's ANOVA was applied. Data were expressed as mean±SEM, *$p<0.05$, **$p<0.01$, ***$p<0.001$, ****$p<0.0001$.

5) We thank you for your suggestion regarding the *in vivo* over-expression experiments. The detailed responses are provided in the response to Major issues (Q5) below.

References:

1. Holemans, K., Caluwaerts, S., Poston, L. & Van Assche, F. A. Diet-induced obesity in

the rat: a model for gestational diabetes mellitus. *Am J Obstet Gynecol* **190**, 858-865 (2004).

2. Velmurugan, S. *et al*. Cardiac remodelling, recognition memory deficits and accelerated ageing in a rat model of gestational diabetes. *Diabetologia* **68**, 1574-1584 (2025).
3. Martin-Carro, B. *et al.* Experimental models to study diabetes mellitus and its complications: Limitations and new opportunities. *Int J Mol Sci* **24** (2023).
4. Gheibi, S., Kashfi, K. & Ghasemi, A. A practical guide for induction of type-2 diabetes in rat: Incorporating a high-fat diet and streptozotocin. *Biomed Pharmacother* **95**, 605-613 (2017).
5. Luo, Y., Tian, Y. & Zhao, C. Taurine attenuates liver autophagy and injury of offspring in gestational diabetic mellitus rats. *Life Sci* **257**, 117889 (2020).
6. Hosni, A. A., Abdel-Moneim, A. A., Abdel-Reheim, E. S., Mohamed, S. M. & Helmy, H. Cinnamaldehyde potentially attenuates gestational hyperglycemia in rats through modulation of PPARgamma, proinflammatory cytokines and oxidative stress. *Biomed Pharmacother* **88**, 52-60 (2017).
7. Chen, Z. *et al*. Epigenetic down-regulation of Sirt 1 via DNA methylation and oxidative stress signaling contributes to the gestational diabetes mellitus-induced fetal programming of heart ischemia-sensitive phenotype in late life. *Int J Biol Sci* **15**, 1240-1251 (2019).
8. Wang, S. *et al.* Protective effect of soy isolate protein against streptozotocin induced gestational diabetes mellitus via TLR4/MyD88/NF-kappaB signaling pathway. *Biomed Pharmacother* **168**, 115688 (2023).
9. de Matos Manoel, B. *et al.* Can maternal exposure to tamoxifen compromise sperm and behavioural parameters of male rat offspring? *Reprod Toxicol* **108**, 1-9 (2021).
10. Lee, J. *et al.* Production of large, defined genome modifications in rats by targeting rat embryonic stem cells. *Stem Cell Reports* **18**, 394-409 (2023).
11. Szpirer, C. Rat models of human diseases and related phenotypes: a systematic inventory of the causative genes. *J Biomed Sci* **27**, 84 (2020).

General Comments:

Although the authors refer to CaMKII activation through the manuscript, there was no direct measurement of CaMKII activation/activity performed. Multiple tools for measuring CaMKII in living cells are available to the community, the most recent and best performing being CaMKAR (https://www.addgene.org/205315/)

Response:

We appreciate your constructive suggestions. In response, we conducted additional experiments using CaMKAR, a highly sensitive and recently developed FRET-based biosensor for monitoring CaMKII activity in living cells[1]. We packaged the CaMKAR plasmid (Addgene #205315) into an adenoviral vector to enhance infection efficiency in NRCMs. Detailed construction strategy has been provided in the **Materials and Methods**.

To directly assess CaMKIIδ activation under hyperglycemic conditions, NRCMs from the HG group were infected with CaMKAR-Adv. As shown in Supplementary Fig. 11E-F, CaMKAR-Adv fluorescence intensity was higher in the HG group compared to the CON

group, indicating an increased CaMKIIδ activity under hyperglycemic condition. (inserted below for the Reviewer's convenience)

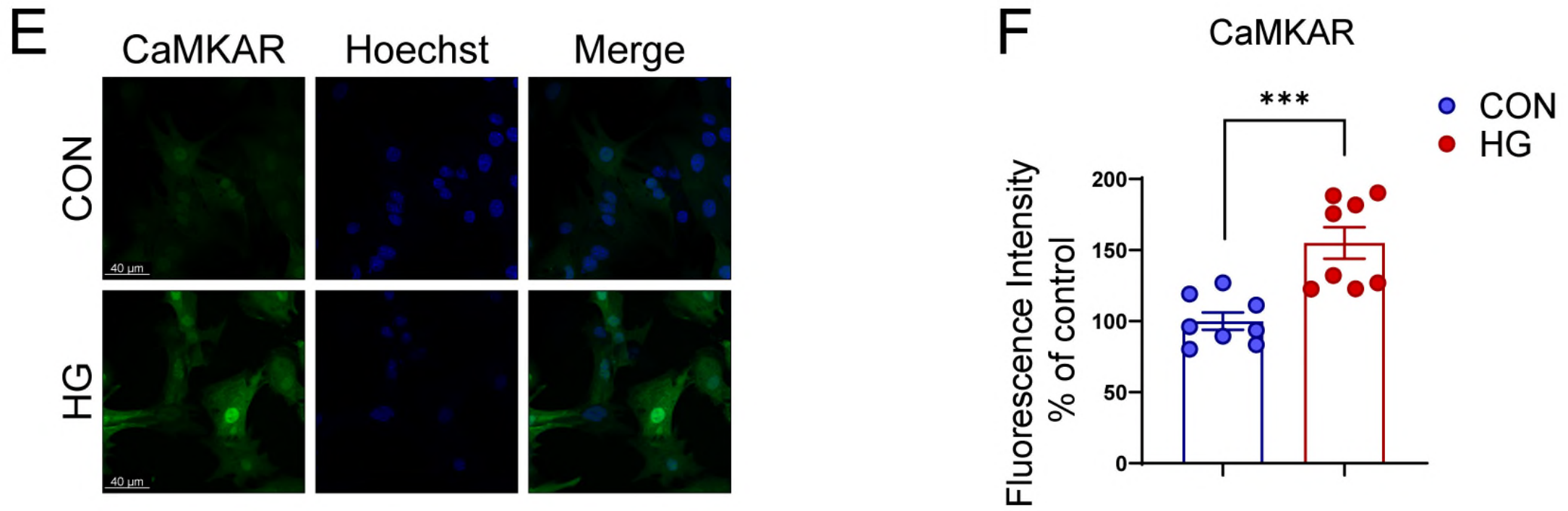

Supplementary Fig. 11E-F: CaMKAR-based evaluation of CaMKIIδ activity in NRCMs. CaMKAR-Adv fluorescence experiments were conducted using NRCMs isolated from E19.5 neonatal hearts. (E) Fluorescence images of cardiomyocytes infected with CaMKAR for 24 hours and stained with Hoechst 33342 for 20 min, scale bar = 40 μm. (F) The statistically quantified data on the cellular fluorescence intensity after infected with CaMKAR, n = 8 per group. For statistical comparisons, we initially evaluated data for normality and equality of variances. Statistical significance was assessed using two-tailed unpaired Student's *t* test. If equal variances were not assumed, Welch's *t* test was applied. Data were expressed as mean±SEM, *p<0.05, **p<0.01, ***p<0.001, ****p<0.0001 vs. CON.

References:

1. Reyes Gaido, O. E. *et al.* An improved reporter identifies ruxolitinib as a potent and cardioprotective CaMKII inhibitor. *Sci Transl Med* **15**, eabq7839 (2023).

Major issues:

(Q1) The banding pattern in the *O*-GlcNAc blot in Fig 2A is not typical for *O*-GlcNAc immunoblots. Specifically, the main visible band at 50kDa likely represents non-specific banding or IgG. There are hardly any visible bands above 50kDa. What bands were used for the quantification in the summary data? These blots should be repeated and consider using a GlcNAc competition blot to assess for non-specific banding.

Response:

Thanks for your constructive comments. Following your suggestion, we repeated the immunoblotting experiments and revised all *O*-GlcNAc-related western blotting using a more stable and widely cited *O*-GlcNAc antibody (Thermo Fisher Scientific; catalog number: MA1-072; with 94 published figures and 172 references supporting its use). The new antibody efficiently detected all bands within the 30-250 kDa range. Representative full-membrane immunoblots and corresponding quantitative analyses are shown below. For quantification of the full-membrane *O*-GlcNAc western blotting results, all visible bands within the 30-250 kDa range were included. Full-membrane images and quantification data are provided in the source data files.

The results demonstrated that *O*-GlcNAcylation levels were significantly elevated in the HG group compared to the CON group (Figure 2A in the revised Manuscript, which is inserted below for the Reviewer's convenience). We hope these additional experiments address your concerns and help further strengthen the study.

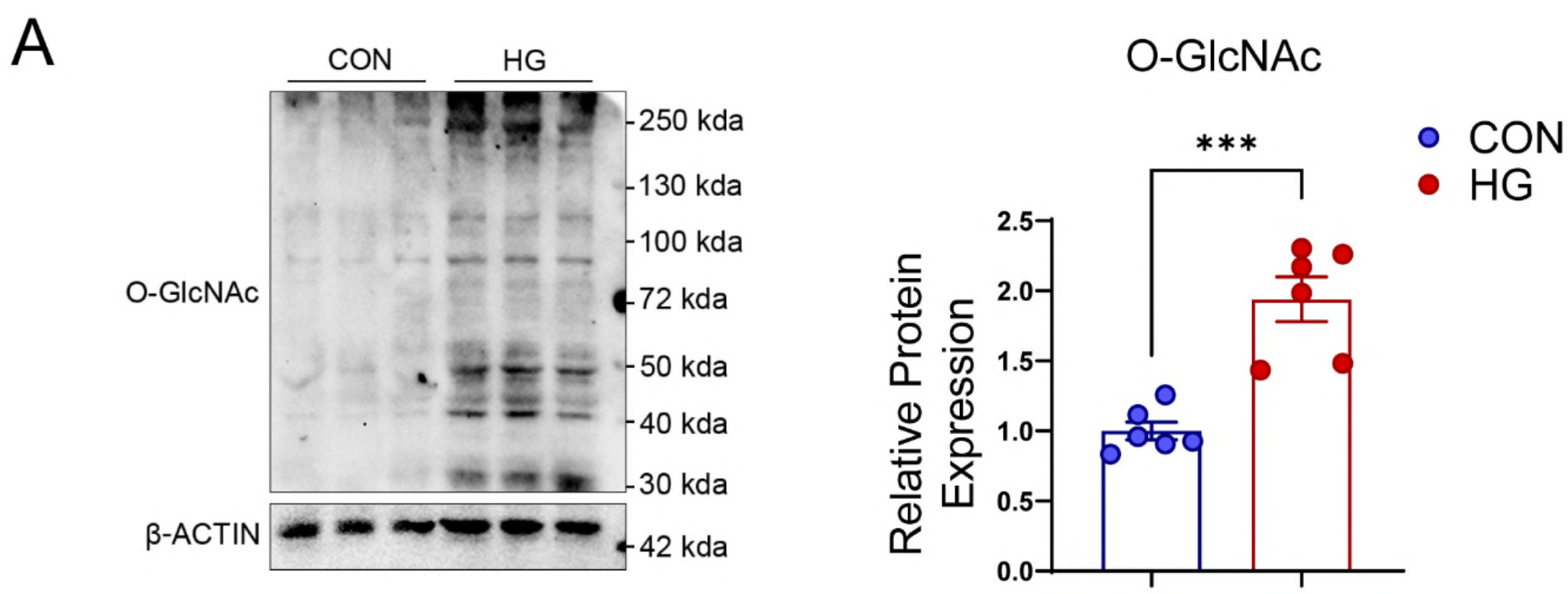

Fig. 2A: Quantification of protein *O*-GlcNAc level of NRCMs. Experiments were performed using E19.5 rat hearts. Overall protein *O*-GlcNAcylation levels in fetal hearts were detected by western blotting (left). Densitometric quantification of the blotting (right), n = 6 per group. For statistical comparisons, we initially evaluated data for normality and equality of variances. Statistical significance was assessed using two-tailed unpaired Student's *t* test. If equal variances were not assumed, Welch's *t* test was applied. Data were expressed as mean±SEM, *p<0.05, **p<0.01, ***p<0.001, ****p<0.0001 vs. CON.

(Q2) What samples were used for the CoIP experiments for *O*-GlcNAC-CaMKKII in Fig 2D – fetal hearts or NRCMs? In reference to #1 above, the banding pattern for the *O*-GlcNAc IB for the input samples (Fig 2D) is quite different from the blot in Fig 2A.

In addition, the CaMKIIδ bands do not appear consistent across the samples in Fig 2D and E. Typical CaMKII band is expected at 50kDa. Fig 2E does not show convincing evidence that CaMKIId is *O*-GlcNAc modified. Given the primary conclusion of this study that *O*-GlcNAc modification of CaMKII is the key mechanism for the in-*utero* effects of hyperglycemia in this model, more convincing data is necessary to support this conclusion (see comments above).

<u>Response</u>:

We apologize for the confusion. To address this, we performed a series of additional experiments as follows:

1) In the CoIP experiment in Figure 2D and 2E, we used lysates from NRCMs.

2) We repeated the *O*-GlcNAc experiments and revised all *O*-GlcNAc-related western blotting using a more stable and widely cited *O*-GlcNAc antibody (Thermo Fisher Scientific; catalog number: MA1-072). Moreover, we performed additional CoIP experiments using a crosslink IP kit (Thermo Scientific, USA, Cat#88848), which covalently immobilized the antibody to magnetic beads. This method ensured that the eluted samples used for subsequent western blotting were free of antibody-derived IgG contamination. Representative CoIP blots with statistical analyses are now shown in Figure 2D and 2E.

The results confirmed the occurrence of CaMKIIδ *O*-GlcNAcylation in fetal cardiomyocytes exposed to in *utero* hyperglycemia, as well as the inhibitory effects of AIP and DON on CaMKIIδ *O*-GlcNAcylation. As shown in Fig. 2D, *O*-GlcNAcylation of CaMKIIδ was increased in the HG+PBS group compared to the CON+PBS group. DON treatment reduced CaMKIIδ *O*-GlcNAcylation in HG-treated NRCMs, as confirmed by quantification. Similar trends were observed in the reverse CoIP using anti-*O*-GlcNAc as the pull-down antibody (Fig. 2E), further validating the interaction between CaMKIIδ and *O*-GlcNAc under hyperglycemic conditions and the inhibitory effects of DON. (Figure 2D-

E in the revised Manuscript, which is inserted below for the Reviewer's convenience)

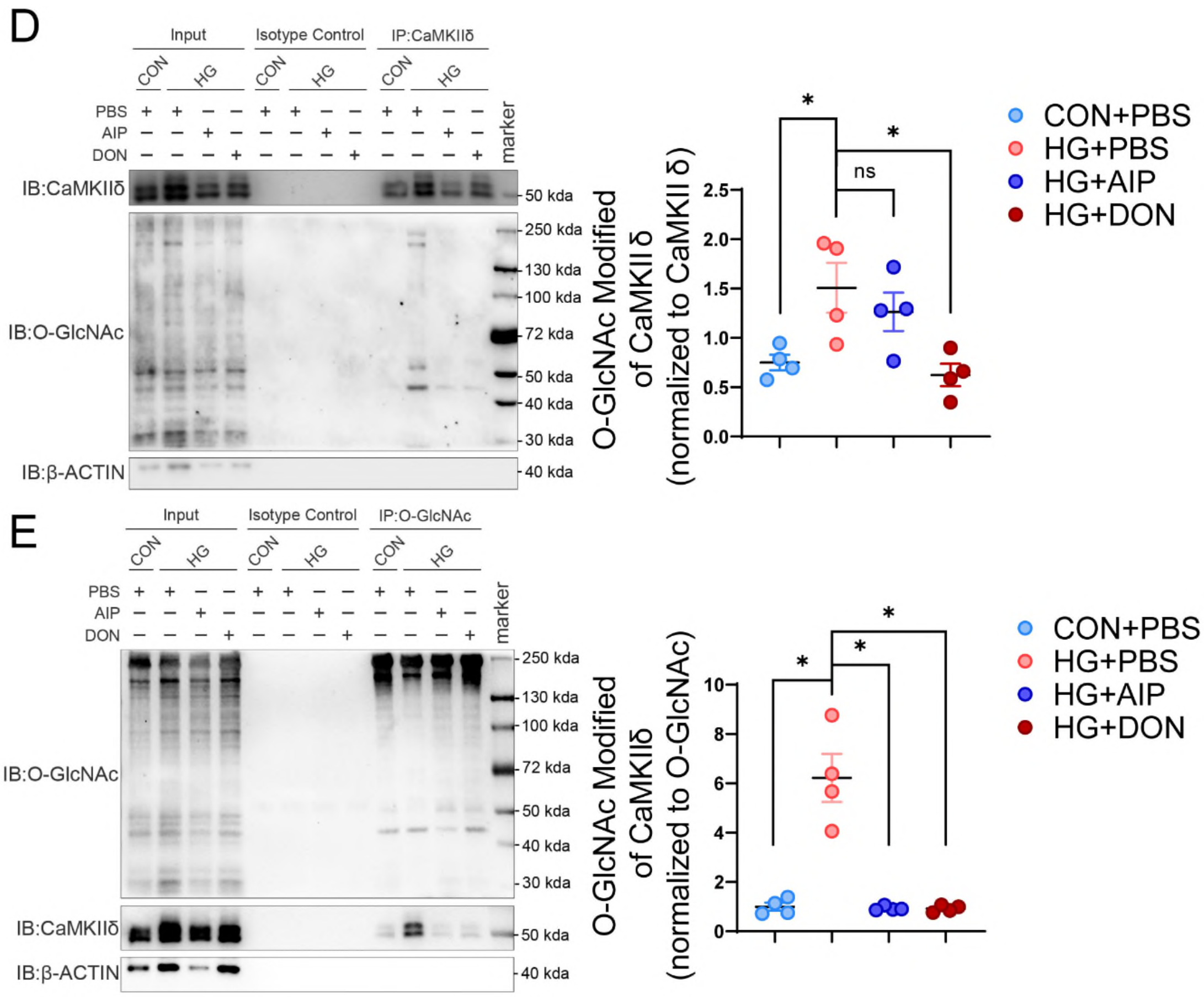

Figure 2D and 2E: CoIP western blotting and analysis of CaMKIIδ *O*-GlcNAcylation. CoIP experiments were performed using NRCMs isolated from E19.5 neonatal hearts, DON was applied on NRCMs at 50 μM for 48 hours, and AIP at 1 μM for 48 hours. (D) Western blotting of CoIP were performed for the CON+PBS, HG+PBS, HG+AIP, HG+DON groups (left). IP: CaMKIIδ. Statistical quantification of the blots (right), n = 4 per group. (E) Western blotting of CoIP were performed for the CON+PBS, HG+PBS, HG+AIP, HG+DON groups (left). IP: *O*-GlcNAc. Statistical quantification of the blots (right), n = 4 per group. For statistical comparisons, data were initially evaluated for normality and equality of variances. One-way analysis of variance (ANOVA) was used to determine statistical differences among multiple groups. If equal variances were not assumed, Welch's ANOVA was applied. Data were expressed as mean±SEM, *p<0.05, **p<0.01, ***p<0.001, ****p<0.0001. Abbreviations: IP, immunoprecipitation; IB, immunoblotting.

3) To validate the role of CaMKIIδ *O*-GlcNAcylation *in vivo*, we engineered two cardiac-specific adenoviral vectors to dissect the functional impact of CaMKIIδ *O*-GlcNAcylation:

i) pADV-cTNT-Camk2d (p.S280A)-HA (abbreviated as AdV-CaMKIIδ-S280A-OE): To preserve kinase activity while blocking hyperglycemia-induced post-translational modification, we overexpressed a CaMKIIδ mutant with abolished *O*-GlcNAcylation at Ser280 (S280A mutation).

ii) pADV-cTNT-mCherry-3xFLAG-shCamk2d (abbreviated as AdV-shCaMKIIδ): To isolate the effects of *O*-GlcNAcylation from baseline CaMKIIδ signaling, we knocked down the endogenous CaMKIIδ (58% reduction in protein levels, $p = 0.0004$).

Simultaneous intramyocardial injection of these two adenoviral vectors into neonatal rat hearts resulted in a specific reduction of CaMKIIδ *O*-GlcNAcylation in *utero*

hyperglycemia.

Next, to verify changes in CaMKIIδ *O*-GlcNAcylation following *in vivo* gene interventions, we isolated NRCMs from hearts injected with adenoviral vectors and performed CoIP experiments. As shown in Supplementary Fig. 9, overexpression of the CaMKIIδ S280A mutant effectively reduced cardiac CaMKIIδ *O*-GlcNAcylation in offspring exposed to intrauterine hyperglycemia. Moreover, the S280A mutant reduced CaMKIIδ activation in response to intrauterine hyperglycemia (inserted below for the Reviewer's convenience).

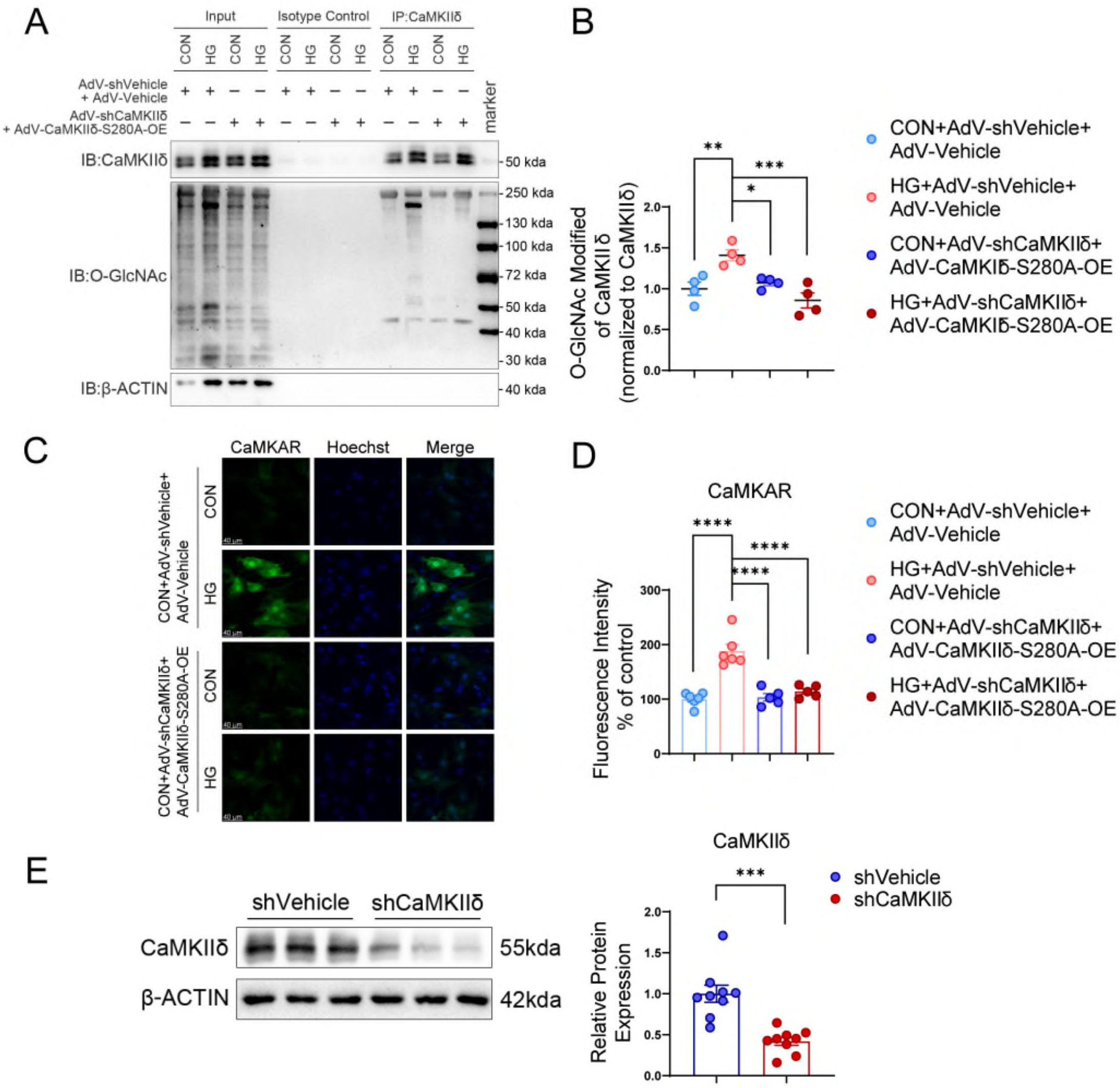

Supplementary Fig. 9: S280A mutation of CaMKIIδ abolished cardiac CaMKIIδ *O*-GlcNAcylation and reduced CaMKIIδ activation. Neonatal rats received injections on postnatal day 3 and were euthanized on postnatal day 7. CoIP experiments were performed using heart tissues from P7 neonatal rats. CaMKAR fluorescence imaging experiments were conducted using NRCMs isolated from P7 neonatal hearts. (A) Western blotting analysis of CoIP were measured for the CON+AdV-shVehicle+AdV-Vehicle, HG+AdV-shVehicle+AdV-Vehicle, CON+AdV-shCaMKIIδ+AdV-CaMKIIδ-S280A-OE, HG+AdV-shCaMKIIδ+AdV-CaMKIIδ-S280A-OE groups, IP: CaMKIIδ, n = 4 per group. (B) The statistically quantified data on the western blotting analysis of *O*-GlcNAc modified CaMKIIδ. (C) Fluorescence images of cardiomyocytes infected with CaMKAR for 24 hours and stained with Hoechst 33342 for 20 min, scale bar = 40 μm. (D) The statistically quantified data on the cellular fluorescence intensity after infected with CaMKAR, n = 5-6 per group. (E) Statistical quantification of CaMKIIδ protein levels following injection of AdV-shCaMKIIδ, n = 9 per group. For statistical

comparisons, data were initially evaluated for normality and equality of variances. Student's *t* test and one-way ANOVA were used to determine the levels of statistical differences between two groups and among multiple groups, respectively. If equal variances were not assumed, Welch's *t* test or Welch's ANOVA were applied. Data were expressed as mean±SEM, *p<0.05, **p<0.01, ***p<0.001, ****p<0.0001. Abbreviations: IP, immunoprecipitation; IB, immunoblotting.

(Q3) There is no demonstration that increased *O*-GlcNAc increases CaMKII activity in this model/study (see above).

Response:

We appreciate your constructive suggestions. In response, we conducted additional experiments using CaMKAR, a highly sensitive and recently developed FRET-based biosensor for monitoring CaMKII activity in living cells[1]. We packaged the CaMKAR plasmid (Addgene #205315) into an adenoviral vector to enhance infection efficiency in NRCMs. Detailed construction strategy has been provided in the **Materials and Methods**.

To directly assess CaMKIIδ activation under hyperglycemic conditions, NRCMs from the HG group were infected with CaMKAR-Adv. As shown in Supplementary Fig. 11E-F, CaMKAR-Adv fluorescence intensity was higher in the HG group compared to the CON group, indicating an increased CaMKIIδ activity under hyperglycemic condition. (inserted below for the Reviewer's convenience)

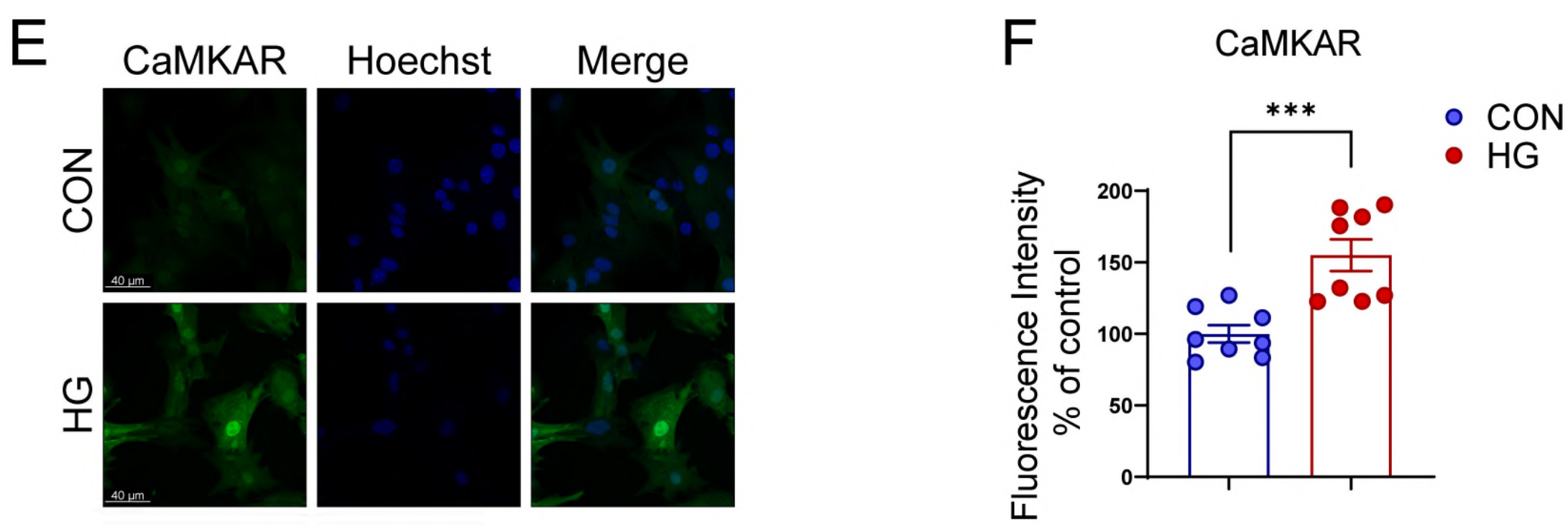

Supplementary Fig. 11E-F: CaMKAR-based evaluation of CaMKIIδ activity in NRCMs from the HG group. CaMKAR-Adv fluorescence experiments were conducted using NRCMs isolated from E19.5 neonatal hearts. (E) Fluorescence images of cardiomyocytes infected with CaMKAR for 24 hours and stained with Hoechst 33342 for 20 min, scale bar = 40 μm. (F) The statistically quantified data on the cellular fluorescence intensity after infected with CaMKAR, n = 8 per group. For statistical comparisons, we initially evaluated data for normality and equality of variances. Statistical significance was assessed using two-tailed unpaired Student's *t* test. If equal variances were not assumed, Welch's *t* test was applied. Data were expressed as mean±SEM, *p<0.05, **p<0.01, ***p<0.001, ****p<0.0001 vs. CON.

References:

1. Reyes Gaido, O. E. *et al.* An improved reporter identifies ruxolitinib as a potent and cardioprotective CaMKII inhibitor. *Sci Transl Med* **15**, eabq7839 (2023).

(Q4) *O*-GlcNAcylation is a dynamic process regulated exclusively by two enzymes – *O*-GlcNAc transferase (OGT) and *O*-GlcNAcase (OGA). Protein expression of OGT was measured (Fig 2B) and elevated in the HG group. It will be insightful to also measure OGA expression in these groups/samples.

Response:

Thank you for your constructive suggestion. In response, we performed additional experiments to assess the mRNA and protein expression of OGA in the hearts of the CON and HG groups. Supplementary Fig. 6A showed significantly decreased OGA mRNA and protein levels in the HG group versus CON, confirmed by western blotting analysis. (Supplementary Fig. 6A-C in the revised Manuscript, which is inserted below for the Reviewer's convenience).

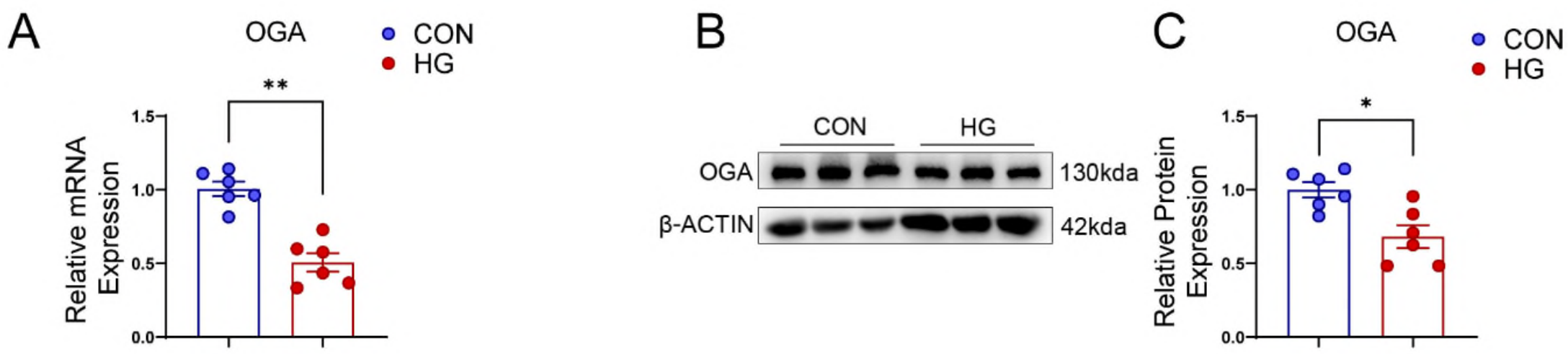

Supplementary Fig. 6A-C: The mRNA and protein expression level of OGA in NRCMs. Experiments were performed using E19.5 rat hearts. (A) mRNA expression level of OGA in the CON and HG groups, n= 6 per group. (B) Representative immunoblots showing OGA protein expression level in the CON and HG groups. (C) Quantification of OGA protein expression level, n = 6 per group. For statistical comparisons, we initially evaluated data for normality and equality of variances. Statistical significance was assessed using two-tailed unpaired Student's *t* test. If equal variances were not assumed, Welch's *t* test was applied. Data were expressed as mean±SEM, *$p<0.05$, **$p<0.01$, ***$p<0.001$, ****$p<0.0001$ vs. CON.

(Q5) Treatment with AIP or DON will inhibit CaMKII or *O*-GlcNAc respectively but the effects observed do not necessarily prove inhibition of CaMKIIδ *O*-GlcNAcylation specifically as suggested in line 450. Mice with myocardial-targeted OGA transgenic over-expression are published and available to the field and would be a dispositive reagent for these studies.

Response:

Thanks for your constructive comments. We fully agree with your point that "Treatment with AIP or DON will inhibit CaMKII or *O*-GlcNAc respectively but the effects observed do not necessarily prove inhibition of CaMKIIδ *O*-GlcNAcylation specifically as suggested". OGA overexpression in hearts conferred enhanced resistance to pathological stress versus wild-type controls by attenuating myocardial *O*-GlcNAcylation[1-3]. Following your suggestion, we engineered a cardiac-specific adenoviral vector to dissect the functional impact of *O*-GlcNAc: pADV-cTNT-HA-Oga (abbreviated as AdV-Oga-OE): To reduce global *O*-GlcNAcylation, *O*-GlcNAcase was overexpressed *via* intramyocardial injection of the adenoviral vector into neonatal rat hearts, leading to decreased global *O*-GlcNAcylation.

1) To verify changes in CaMKIIδ *O*-GlcNAcylation following *in vivo* gene interventions, we isolated NRCMs from hearts injected with adenoviral vectors and performed CoIP experiments. OGA overexpression reduced cardiac CaMKIIδ *O*-GlcNAcylation in CoIP experiments and inhibited CaMKIIδ activation following CaMKAR-Adv infection (Supplementary Fig. 7 in the revised Manuscript, which is inserted below for the Reviewer's convenience).

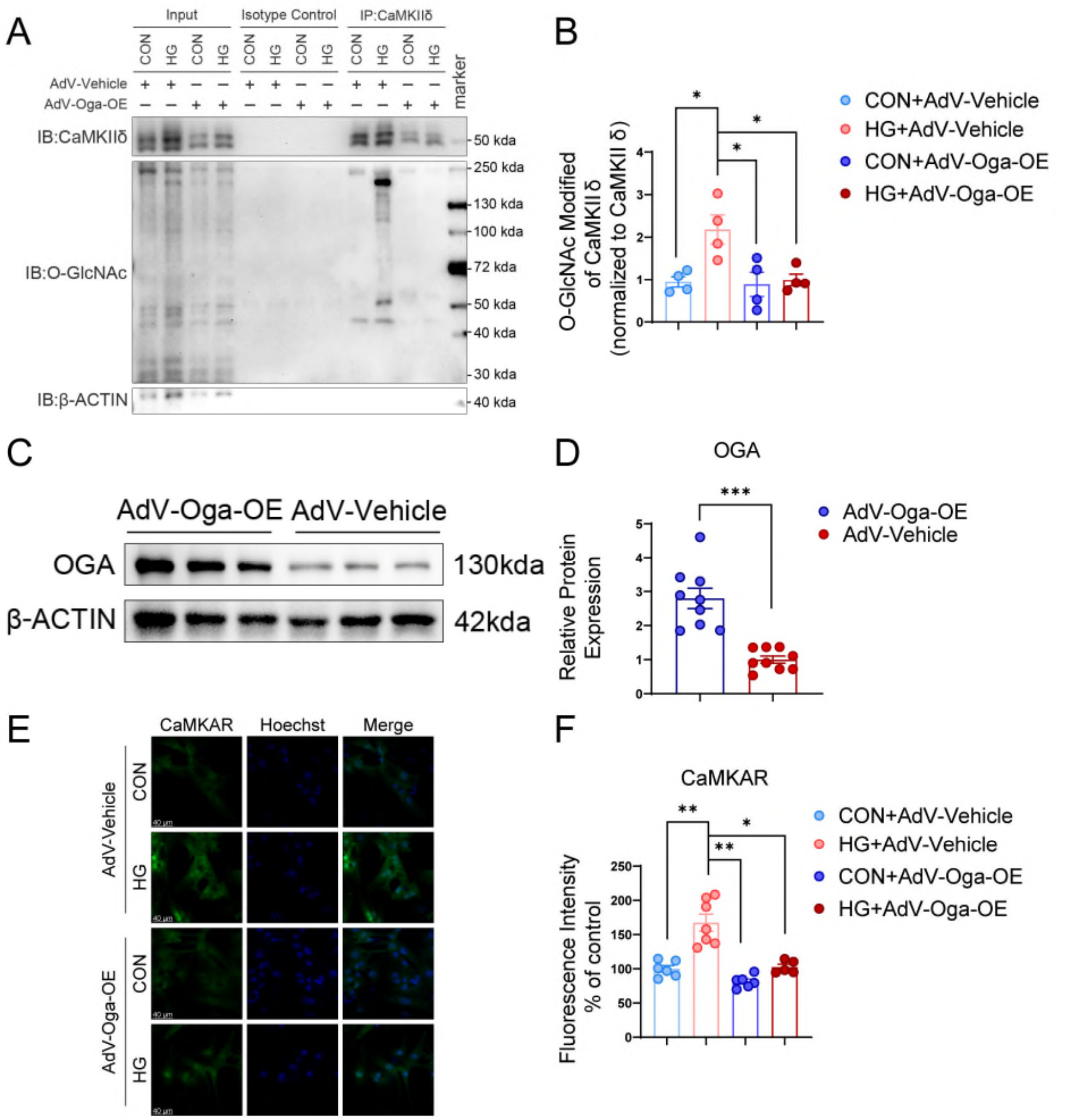

Supplementary Fig. 7: Overexpression of OGA reduced cardiac CaMKIIδ *O*-GlcNAcylation and inhibited CaMKIIδ activation. Neonatal rats received injections on postnatal day 3 and were euthanized on postnatal day 7. CoIP and western blotting experiments were performed using heart tissues from P7 neonatal rats. CaMKAR fluorescence imaging experiments were conducted using NRCMs isolated from P7 neonatal hearts. (A) Western blotting analysis of CoIP were measured for the CON+AdV-Vehicle, HG+AdV-Vehicle, CON+ AdV-Oga-OE, HG+AdV-Oga-OE groups, IP: CaMKIIδ, n = 4 per group. (B) The statistically quantified data on the western blotting analysis of *O*-GlcNAc modified CaMKIIδ. (C) Representative immunoblots of OGA protein expression in the following groups: CON + AdV-Vehicle, HG + AdV-Vehicle, CON + AdV-OGA-OE, and HG + AdV-OGA-OE. (D) Quantification of OGA protein level shown in panel C, n = 9 per group. (E) Fluorescence images of cardiomyocytes infected with CaMKAR for 24 hours and stained with Hoechst 33342 for 20 min, scale bar = 40 μm. (F) The statistically quantified data on the cellular fluorescence intensity after infected with CaMKAR, n = 5-7 per group. For statistical comparisons, data were initially evaluated for normality and equality of variances. Student's *t* test and one-way ANOVA were used to determine the levels of statistical differences between two groups and among multiple groups, respectively. If equal variances were not assumed, Welch's *t* test or Welch's ANOVA were applied. Data were expressed as mean±SEM, *p<0.05, **p<0.01, ***p<0.001, ****p<0.0001. Abbreviations: IP, immunoprecipitation; IB, immunoblotting.

2) Next, to investigate *in vivo* changes in cardiac function following AdV-Oga-OE

overexpression, we conducted a series of histological analyses and western blotting experiments. As shown in Supplementary Fig. 8, overexpression of the AdV-Oga-OE effectively attenuated myocardial fibrosis in offspring from HG group. Moreover, the AdV-Oga-OE reduced cardiomyocyte cross-sectional area in the HG group. Additionally, the expression levels of ANP and BNP were decreased in the HG+AdV-Oga-OE group compared to the HG+AdV-Vehicle group. Notably, plasma mtDNA levels were also reduced in the HG+AdV-Oga-OE group (inserted below for the Reviewer's convenience).

3) We thank you for your suggestion regarding *in vivo* over-expression experiments using knockin mice. In the present study, we performed *in vivo* cardiac-specific over-expression experiments in rat models (*via* intramyocardial injection of adenoviral vectors into neonatal rat hearts) because: 1) compared with mice, rats are more suitable for GDM research: the larger fetal body size of rats allows more reliable monitoring of fetal parameters[4-7](for instance, fetal heart weight, body length, and placental weight); 2) fetal echocardiography can be assessed more accurately in neonatal rats[5,8-11].

Rodent models with conditional myocardial-targeted transgenic over-expression are suitable for studying the effects of cardiac-specific genetic activation in various cardiac diseases. However, since our experiments studied GDM-related fetal cardiac disorders and were conducted in neonatal rats from E19.5 to P7, cardiac-specific knockin rodent models (in the maternal or fetal heart) are technically difficult to perform. For example: 1) The Cre-ERT2/LoxP system is widely used for generating conditional knockin rodent models. However, it relies on tamoxifen administration, which is known to interfere with offspring development[12]. If tamoxifen is administered during pregnancy, both vehicle and experimental groups may experience embryonic lethality and developmental malformations[13,14]. In addition, tamoxifen administration during pregnancy may induce maternal cardiac-specific gene expression changes, which could potentially affect the experimental outcomes. 2) If tamoxifen is administered after birth, gene editing typically requires continuous injections for 5-7 days[15-17], leaving too short a window for gene expression before neonatal euthanasia. 3) Alternative approaches using Cre/LoxP without ERT2 avoid tamoxifen-related effects but lack temporal control over gene induction.

Thus, in response to your concerns, we employed cardiomyocyte-specific adenoviral vectors (AdV-Oga-OE) to target OGA in neonatal rats. The details are provided as Supplementary Fig. 7 and Supplementary Fig. 8 in the revised manuscript. We hope that these clarifications and additional data address your concerns and help further strengthen the study.

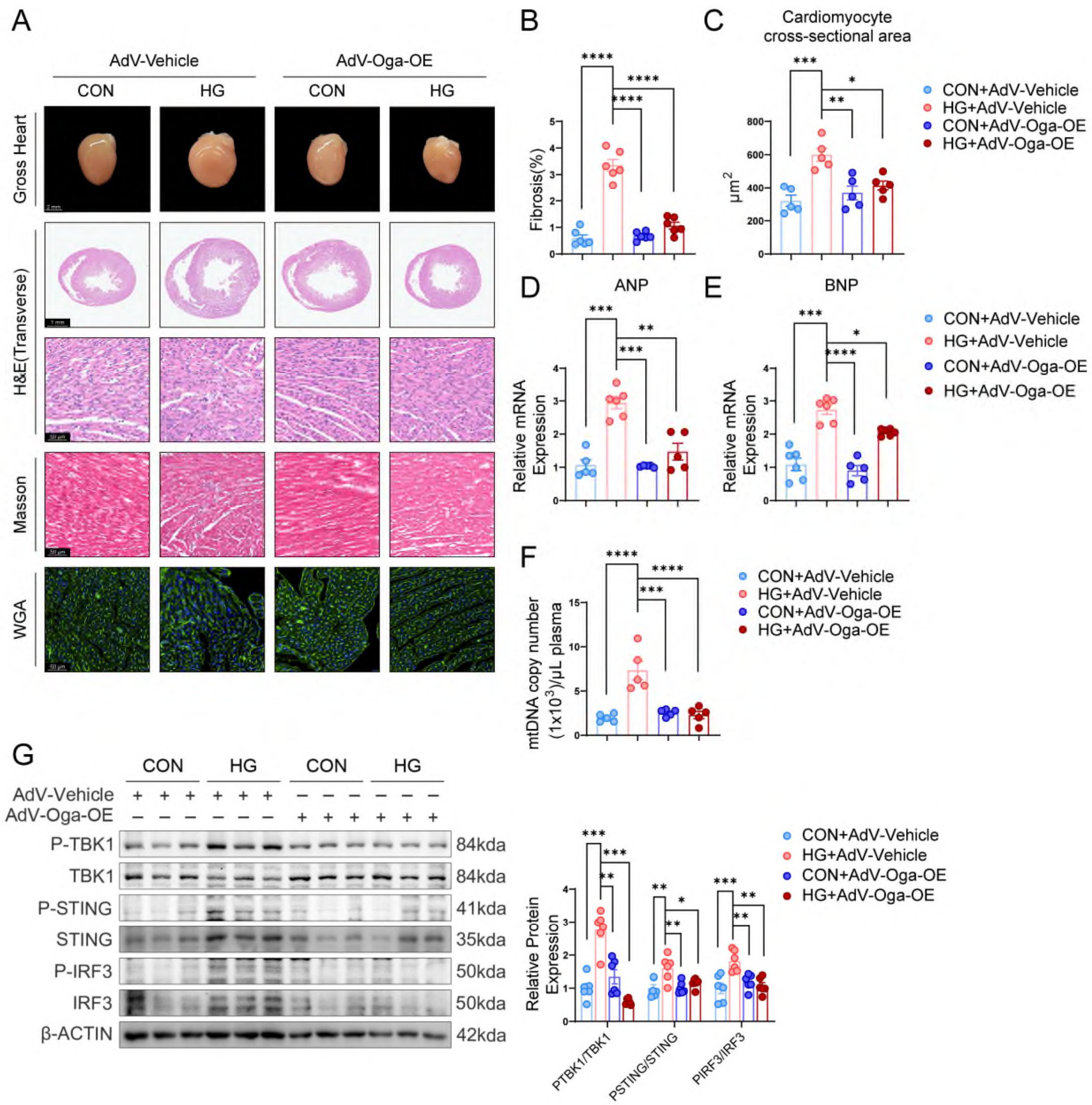

Supplementary Fig. 8: Overexpression of OGA recovered cardiac remodeling in offspring subjected to intrauterine hyperglycemia. Neonatal rats received injections on postnatal day 3 and were euthanized on postnatal day 7. (A) Echocardiography, gross morphology and histological analyses were conducted for multiple groups of fetal hearts. Gross morphology, scale bar = 2.0 mm; HE, Masson and WGA staining, scale bar = 50 μm. (B) Quantitative analysis was performed on fibrotic area (Masson staining), n = 6 per group. (C) Quantitative analysis was performed on cardiomyocyte cross-sectional area (WGA staining), n = 5 per group. (D)ANP gene expression was quantified by RT-qPCR, n = 5-6 per group. (E) BNP gene expression was quantified by RT-qPCR, n = 5-6 per group. (F) Plasma mtDNA copy number was assessed, n = 5 per group. (G) Phosphorylated and total protein levels in hearts from multiple groups were detected by western blotting (left). Densitometric quantification of the blots (right), n = 6 per group. For statistical comparisons, data were initially evaluated for normality and equality of variances. One-way ANOVA was used to determine statistical differences among multiple groups. If equal variances were not assumed, Welch's ANOVA was applied. Data were expressed as mean±SEM, *p<0.05, **p<0.01, ***p<0.001, ****p<0.0001.

References:

1. Umapathi, P. *et al.* Excessive O-GlcNAcylation causes heart failure and sudden death. *Circulation* **143**, 1687-1703 (2021).

2. Prakoso, D. *et al.* Fine-tuning the cardiac O-GlcNAcylation regulatory enzymes

governs the functional and structural phenotype of the diabetic heart. *Cardiovasc Res* **118**, 212-225 (2022).

3. Packer, M. Foetal recapitulation of nutrient surplus signalling by O-GlcNAcylation and the failing heart. *Eur J Heart Fail* **25**, 1199-1212 (2023).
4. Holemans, K., Caluwaerts, S., Poston, L. & Van Assche, F. A. Diet-induced obesity in the rat: a model for gestational diabetes mellitus. *Am J Obstet Gynecol* **190**, 858-865 (2004).
5. Velmurugan, S. *et al.* Cardiac remodelling, recognition memory deficits and accelerated ageing in a rat model of gestational diabetes. *Diabetologia* **68**, 1574-1584 (2025).
6. Martin-Carro, B. *et al.* Experimental models to study diabetes mellitus and its complications: Limitations and new opportunities. *Int J Mol Sci* **24** (2023).
7. Gheibi, S., Kashfi, K. & Ghasemi, A. A practical guide for induction of type-2 diabetes in rat: Incorporating a high-fat diet and streptozotocin. *Biomed Pharmacother* **95**, 605-613 (2017).
8. Luo, Y., Tian, Y. & Zhao, C. Taurine attenuates liver autophagy and injury of offspring in gestational diabetic mellitus rats. *Life Sci* **257**, 117889 (2020).
9. Hosni, A. A., Abdel-Moneim, A. A., Abdel-Reheim, E. S., Mohamed, S. M. & Helmy, H. Cinnamaldehyde potentially attenuates gestational hyperglycemia in rats through modulation of PPARgamma, proinflammatory cytokines and oxidative stress. *Biomed Pharmacother* **88**, 52-60 (2017).
10. Chen, Z. *et al.* Epigenetic down-regulation of Sirt 1 via DNA methylation and oxidative stress signaling contributes to the gestational diabetes mellitus-induced fetal programming of heart ischemia-sensitive phenotype in late life. *Int J Biol Sci* **15**, 1240-1251 (2019).
11. Wang, S. *et al.* Protective effect of soy isolate protein against streptozotocin induced gestational diabetes mellitus via TLR4/MyD88/NF-kappaB signaling pathway. *Biomed Pharmacother* **168**, 115688 (2023).
12. de Matos Manoel, B. *et al.* Can maternal exposure to tamoxifen compromise sperm and behavioural parameters of male rat offspring? *Reprod Toxicol* **108**, 1-9 (2021).
13. Ilchuk, L. A. *et al.* Limitations of tamoxifen application for in vivo genome editing using Cre/ER(T2) system. *Int J Mol Sci* **23** (2022).
14. Ved, N., Curran, A., Ashcroft, F. M. & Sparrow, D. B. Tamoxifen administration in pregnant mice can be deleterious to both mother and embryo. *Lab Anim* **53**, 630-633 (2019).
15. Kang, M. *et al.* Latrophilin-2 deletion in cardiomyocyte disrupts cell junction, leading to D-CMP. *Circ Res* **135**, 1098-1115 (2024).
16. Yang, R. *et al.* Cardiomyocyte betaII spectrin plays a critical role in maintaining cardiac function by regulating mitochondrial respiratory function. *Cardiovasc Res* **120**, 1312-1326 (2024).
17. Feil, S., Valtcheva, N. & Feil, R. Inducible Cre mice. *Methods Mol Biol* **530**, 343-363 (2009).

(Q6) How were AIP and DON administered, this is not clear in the text of the manuscript although Fig 3 legend would suggest cardiomyocytes were treated with these compounds. Can the authors clarify – were these in the pregnant maternal rats or fetuses vs in *in-vitro* experiments with cardiomyocytes. What doses and routes were administered and how long was the duration of treatment? AIP should be defined at first use, Autocamtide-2-Related Inhibitory Peptide (AIP)

Response:

Sorry for the confusion. For Fig.3, AIP and DON were used on NRCMs. Specifically, primary cardiomyocytes were isolated from E19.5 offspring hearts of both the CON and HG groups. After cell attachment, DON was applied on NRCMs at 50 μM for 48 hours, and AIP at 1 μM for 48 hours. These experimental conditions were based on previous literature and were optimized to achieving effective inhibition[1-4].

Furthermore, we have added the following statement in the **Materials and Methods** section of the revised Manuscript: "Following cell attachment, the inhibitors were added directly to the culture medium and maintained. NRCMs were treated with the CaMKIIδ inhibitor AIP (1 μM; HY-P0214, MedChemExpress, China) or the *O*-GlcNAcylation inhibitor DON (50 μM; S8620, Selleck, USA) for 48 hours prior to harvest[2,5,6]. CsA (10 μM; HY-B0579, MedChemExpress, China) was added to the culture medium for the final 4 hours prior to sample collection[7]."

References:

1. Herzog, R. *et al.* Dynamic O-linked N-acetylglucosamine modification of proteins affects stress responses and survival of mesothelial cells exposed to peritoneal dialysis fluids. *J Am Soc Nephrol* **25**, 2778-2788 (2014).
2. Erickson, J. R. *et al.* Diabetic hyperglycaemia activates CaMKII and arrhythmias by O-linked glycosylation. *Nature* **502**, 372-376 (2013).
3. Gonano, L. A. *et al.* Calcium-calmodulin kinase II mediates digitalis-induced arrhythmias. *Circ Arrhythm Electrophysiol* **4**, 947-957 (2011).
4. Trum, M. *et al.* Empagliflozin inhibits increased Na influx in atrial cardiomyocytes of patients with HFpEF. *Cardiovasc Res* **120**, 999-1010 (2024).
5. Sharma, N. S. *et al.* Targeting tumor-intrinsic hexosamine biosynthesis sensitizes pancreatic cancer to anti-PD1 therapy. *J Clin Invest* **130**, 451-465 (2020).
6. Daniels, L. J. *et al.* Inhibition of calcium/calmodulin-dependent kinase II restores contraction and relaxation in isolated cardiac muscle from type 2 diabetic rats. *Cardiovasc Diabetol* **17**, 89 (2018).
7. Chao, T. *et al.* Autophagy restricts mitochondrial DNA damage-induced release of ENDOG (endonuclease G) to regulate genome stability. *Autophagy* **17**, 3444-3460 (2021).

(Q7) Is it helpful to measure the insulin levels especially in the fetuses? Are the effects seen due to hyperglycemia vs hyperinsulinemia or both?

Response:

We appreciate your thoughtful comment. Following your suggestion, we performed insulin measurements in the offspring. Specifically, plasma insulin levels were assessed in

fetal rats at embryonic day 19.5 and in postnatal day 7 rats. Plasma insulin levels were significantly elevated in both E19.5 and P7 offspring from the HG group compared to the CON group. (Supplementary Fig. 6G in the revised Manuscript, which is inserted below for the Reviewer's convenience).

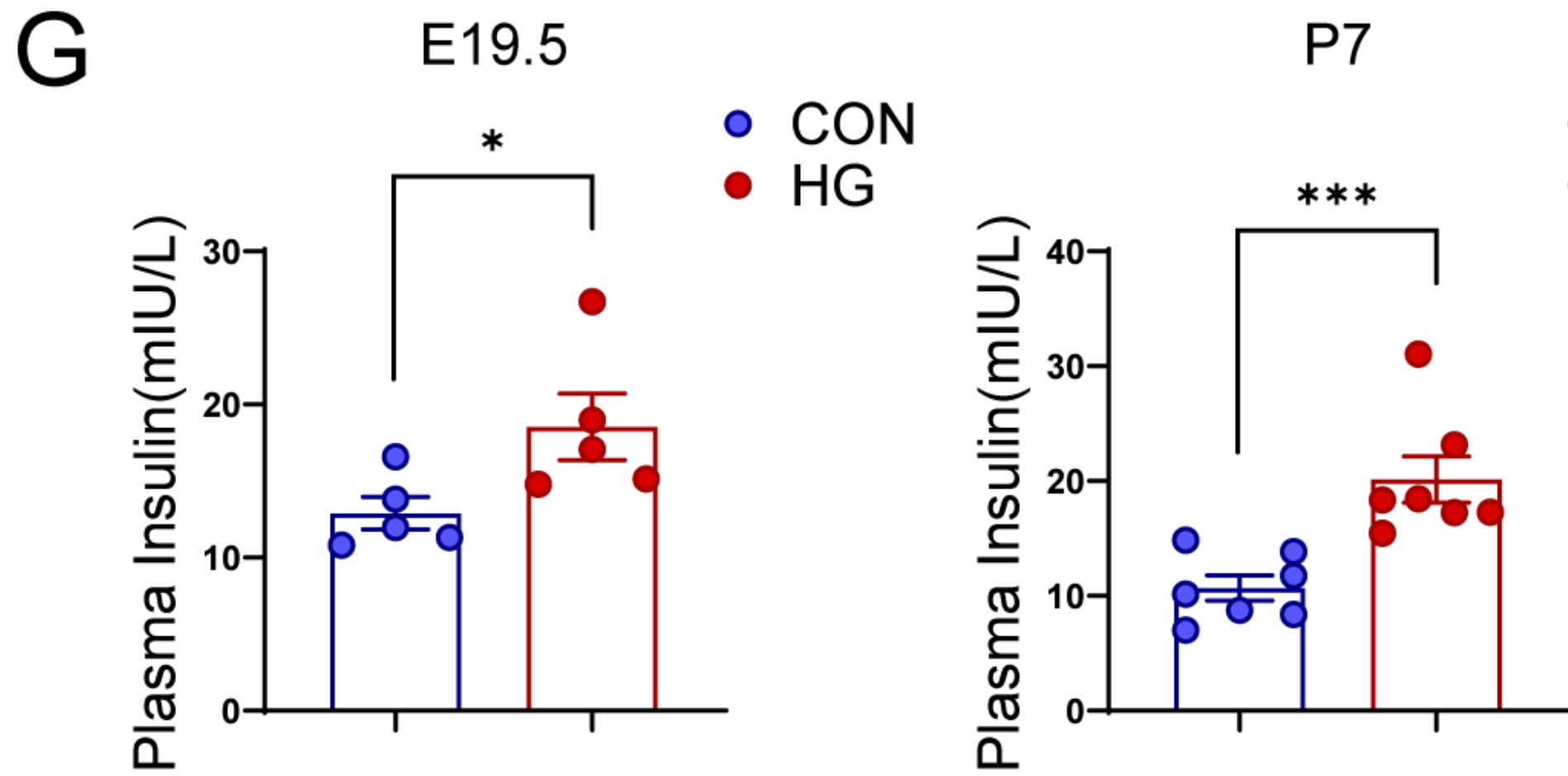

Supplementary Fig. 6G: Plasma insulin levels of E19.5 and P7 offspring. Experiments were performed using E19.5 and P7 rat plasma. (G) Plasma insulin levels of E19.5 rats, n = 5 per group (left). Plasma insulin levels of P7 rats, n= 7 per group (right). For statistical comparisons, we initially evaluated data for normality and equality of variances. Statistical significance was assessed using two-tailed unpaired Student's *t* test. If equal variances were not assumed, Welch's *t* test was applied. Data were expressed as mean±SEM, $*p<0.05$, $**p<0.01$, $***p<0.001$, $****p<0.0001$ vs. CON.

Many clinical and experimental studies have demonstrated that gestational diabetes often leads to secondary fetal hyperinsulinemia due to chronic intrauterine hyperglycemia[1]. This phenomenon occurs as a consequence of the early transition to β-cell glucose competency, as evidenced by an expanded islet β-cell mass and an increase in fetal insulin levels measured in the amniotic fluid[2,3]. Meanwhile, the development of fetal insulin resistance is generally not sufficient to protect neonates of diabetic mothers from excessive metabolic dysfunctions. Also, fetal hyperinsulinemia has been demonstrated to enhance glucose transfer from mother to fetus by increasing the maternal-fetal glucose concentration gradient[4]. Eventually, the offspring of mothers with type 1 diabetes with the highest insulin/glucose ratio measured at birth were observed with signs of persistent insulin hypersecretion, on average, 1.4 years after birth[5], indicating the persistence of fetal insulin hypersecretion into childhood. Therefore, based on previous evidence and our data, gestational diabetes commonly induces secondary fetal hyperinsulinemia as a result of chronic intrauterine hyperglycemia. Moreover, fetal hyperinsulinemia further promotes glucose transfer from mother to fetus by increasing the maternal-fetal glucose gradient. Consequently, hyperinsulinemia and hyperglycemia likely act synergistically.

References:

1. Hivert, M. F. *et al.* Pathophysiology from preconception, during pregnancy, and beyond. *Lancet* **404**, 158-174 (2024).

2. Carpenter, M. W. *et al.* Amniotic fluid insulin at 14-20 weeks' gestation: association with later maternal glucose intolerance and birth macrosomia. *Diabetes Care* **24**, 1259-1263 (2001).

3. Reiher, H. *et al.* Age-dependent insulin secretion of the endocrine pancreas in vitro from fetuses of diabetic and nondiabetic patients. *Diabetes Care* **6**, 446-451 (1983).

4. Bianco, M. E. & Josefson, J. L. Hyperglycemia during pregnancy and long-term offspring outcomes. *Curr Diab Rep* **19**, 143 (2019).
5. Harder, T., Kohlhoff, R., Dorner, G., Rohde, W. & Plagemann, A. Perinatal 'programming' of insulin resistance in childhood: critical impact of neonatal insulin and low birth weight in a risk population. *Diabet Med* **18**, 634-639 (2001).

(Q8) In this study, fetal body weight was down in the fetuses of hyperglycemic maternal rats (Fig 1D). How do the authors explain this finding in the context of what is known about hyperinsulinism in gestational diabetes resulting in macrosomia/big babies?

Response:

Thank you for your comment. Indeed, fetal macrosomia is a common complication associated with maternal diabetes[1,2]. However, consistent with several previous studies using maternal hyperglycemia models[3-5], we observed a slightly reduction in fetal body weight at E19.5 in the HG group compared to controls (Fig. 1D). This difference may be due to the severity and timing of maternal hyperglycemia in models. Specifically, the STZ-induced hyperglycemia prior to pregnancy likely resulted in early and sustained maternal metabolic disturbances, including impaired placental development and nutrient transfer capacity. These factors have been previously reported to restrict intrauterine growth[6].

References:

1. Ye, W. *et al.* Gestational diabetes mellitus and adverse pregnancy outcomes: systematic review and meta-analysis. *BMJ* **377**, e067946 (2022).
2. Venkatesh, K. K. *et al.* Risk of adverse pregnancy outcomes among pregnant individuals with gestational diabetes by race and ethnicity in the United States, 2014-2020. *JAMA* **327**, 1356-1367 (2022).
3. Zhang, Q. *et al.* GLUT1 exacerbates trophoblast ferroptosis by modulating AMPK/ACC mediated lipid metabolism and promotes gestational diabetes mellitus associated fetal growth restriction. *Mol Med* **30**, 257 (2024).
4. Steele, E. J. Observations on offspring of mice made diabetic with streptozocin. *Diabetes* **37**, 1035-1043 (1988).
5. Dong, M. Z. *et al.* Diabetic uterine environment leads to disorders in metabolism of offspring. *Front Cell Dev Biol* **9**, 706879 (2021).
6. Ishihara, G., Hiramatsu, Y., Masuyama, H. & Kudo, T. Streptozotocin-induced diabetic pregnant rats exhibit signs and symptoms mimicking preeclampsia. *Metabolism* **49**, 853-857 (2000).

(Q9) Lines 602 – 605, some of the claims here in the discussion about mitochondrial Ca^{2+} homeostasis, mitochondrial swelling and opening of mPTP are overstated, as they were not directly tested in this study as suggested.

Response:

Thank you for your suggestion. In response, we have conducted additional experiments to assess mPTP opening in both the CON and HG groups (Supplementary Fig. 11A-B). In addition, mitochondrial Ca^{2+} levels were evaluated using the Rhod-2 AM fluorescent probe (Supplementary Fig. 11C-D). The results showed that Calcein AM fluorescence intensity

was reduced in the HG group compared to the CON group, indicating enhanced mPTP opening (Supplementary Fig. 11A-B). Concurrently, Rhod-2 AM fluorescence intensity was markedly increased in the HG group (Supplementary Fig. 11C-D), reflecting mitochondrial Ca^{2+} overload. These findings suggested that fetal exposure to hyperglycemia disrupts mitochondrial homeostasis, characterized by increased permeability transition and abnormal calcium accumulation.

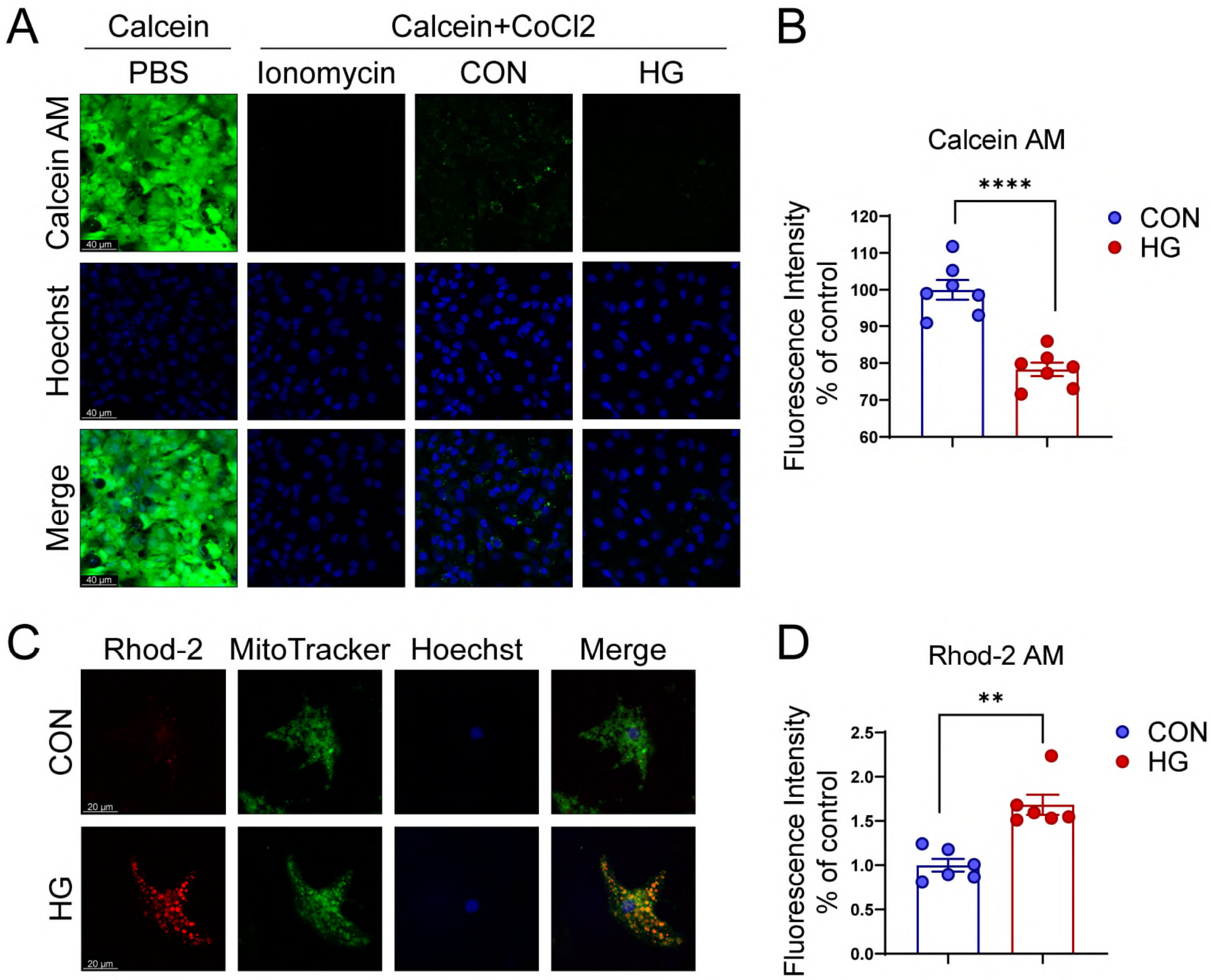

Supplementary Fig. 11A-D: Evaluation of mPTP opening and mitochondrial Ca^{2+} levels in NRCMs. Fluorescence experiments were conducted using NRCMs isolated from E19.5 neonatal hearts. (A) Representative fluorescence images of NRCMs stained with Calcein AM (green) and Hoechst 33342 (blue) to evaluate mPTP opening., scale bar = 40 μm. (B) Quantification of Calcein AM (green) fluorescence intensity, n = 7 per group. (C) Representative fluorescence images of NRCMs stained with Rhod-2 AM (red), MitoTracker (green), and Hoechst 33342 (blue) to assess mitochondrial Ca^{2+} levels, scale bar = 20 μm. (D) Quantification of Rhod-2 AM (red) fluorescence intensity, n = 6 per group. For statistical comparisons, we initially evaluated data for normality and equality of variances. Statistical significance was assessed using two-tailed unpaired Student's *t* test. If equal variances were not assumed, Welch's *t* test was applied. Data were expressed as mean±SEM, **p*<0.05, ***p*<0.01, ****p*<0.001, *****p*<0.0001 vs. CON.

We have added the following related discussion in the revised Manuscript: "Here, we demonstrate that hyperglycemia activates the *O*-GlcNAcylation level of CaMKIIδ, disrupts mitochondrial Ca^{2+} homeostasis, impairs mitochondrial oxidative respiration, and eventually promotes the open of mPTP." (lines 528-530 of the revised manuscript)

Minor issues:

(Q1) Was STZ injected for 3 days consecutively? It is not clearly stated in the

manuscript.

Response:

We apologize for the confusion. Both CON and HG groups received daily injections for three consecutive days. We have added the following statement in the **Materials and Methods** section of the revised Manuscript: "Rats in the HG group received intraperitoneal injections of STZ (50 mg/kg body weight, Sigma, USA) dissolved in 0.01 M citric acid buffer (pH 4.5) after overnight fasting. Rats in the CON group were injected with an equivalent volume of citric acid buffer. Both CON and HG groups received daily injections for three consecutive days[1,2]." (lines 564-567 of the revised manuscript)

References:

1. Kumar, S. D., Dheen, S. T. & Tay, S. S. Maternal diabetes induces congenital heart defects in mice by altering the expression of genes involved in cardiovascular development. *Cardiovasc Diabetol* **6**, 34 (2007).
2. Mdaki, K. S. *et al.* Maternal high-fat diet impairs cardiac function in offspring of diabetic pregnancy through metabolic stress and mitochondrial dysfunction. *Am J Physiol Heart Circ Physiol* **310**, H681-692 (2016).

(Q2) How long were the female mice diabetic prior to mating with normal male rats?

Response:

Sorry for the confusion. The female rats were injected with STZ following the observation of a vaginal plug, and the symptoms of hyperglycemia appeared after the first confirmation of pregnancy. We have added the following statement in the **Materials and Methods** section of the revised Manuscript: "After a one-week acclimation period, normal female rats were mated with male rats at a 1:1 ratio in the same experimental animal cages in the evening. On the following morning, the male and female rats were separated, and the presence of a vaginal plug in the female rats was checked. The day on which a vaginal plug was observed was designated as gestational day 0 (G0). Subsequently, the female rats were randomly assigned to either the CON group or the HG group. Rats in the HG group received intraperitoneal injections of STZ (50 mg/kg body weight, Sigma, USA) dissolved in 0.01 M citric acid buffer (pH 4.5) after overnight fasting. Rats in the CON group were injected with an equivalent volume of citric acid buffer. Both CON and HG groups received daily injections for three consecutive days[1,2]."

References:

1. Kumar, S. D., Dheen, S. T. & Tay, S. S. Maternal diabetes induces congenital heart defects in mice by altering the expression of genes involved in cardiovascular development. *Cardiovasc Diabetol* **6**, 34 (2007).
2. Mdaki, K. S. *et al.* Maternal high-fat diet impairs cardiac function in offspring of diabetic pregnancy through metabolic stress and mitochondrial dysfunction. *Am J Physiol Heart Circ Physiol* **310**, H681-692 (2016).

(Q3) For the data in Fig 2J, may be helpful for the overlap 70 genes be legibly displayed for instance in a supplemental table in addition to the figure in 2J.

Response:

Thank you for your suggestion. In response, we have added a supplemental table (Supplementary Table1) listing the 70 overlapping genes corresponding to Fig. 2J.

(Q4) Line 432 – 'Given that *O*-Glycan biosynthesis pathways and abnormal Ca^{2+} homeostasis are the top of 'core pathways' – what is the basis of this statement?

Response:

Sorry for the confusion. In our RNA-seq analysis, pathways related to "*O*-glycan biosynthesis" (Other types of *O*-glycan biosynthesis pathways) and "abnormal Ca^{2+} homeostasis" (Calcium ion binding) were among the top 10 significantly enriched pathways. In addition, considering the differential expression of genes regulated by CaMKIIδ shown in Fig. 2I, we further investigated whether CaMKIIδ (Calcium/Calmodulin-Dependent Protein Kinase II) was involved in intrauterine hyperglycemia-induced morphological and functional changes in fetal cardiomyocytes. Therefore, we have deleted the "Given that *O*-Glycan biosynthesis pathways and abnormal Ca^{2+} homeostasis are the top of core pathways" sentence and revised it as follows in the **Results** section of the revised manuscript: "Based on the differences in "*O*-glycan biosynthesis pathways" (Other types of *O*-glycan biosynthesis pathways) and "abnormal Ca^{2+} homeostasis" (Calcium ion binding) observed in the RNA-seq data of CON and HG groups in Fig. 2F and Fig. 2G, as well as the differential expression of genes regulated by CaMKIIδ in Fig. 2I, we further investigated whether CaMKIIδ was involved in intrauterine hyperglycemia induced morphology and function change in fetal cardiomyocytes." (lines 237-242 of the revised manuscript).

(Q5) Several abbreviations should be defined at first mention in the manuscript. Examples include - AIP - Autocamtide-2-Related Inhibitory Peptide- GDM – gestational diabetes mellitus

Response:

This has been done. Following your suggestion, we have now clearly defined these abbreviations upon their first mention in the revised manuscript:

1) DON: "Then, coimmunoprecipitation (CoIP) experiments confirmed that elevated *O*-GlcNAcylation levels of CaMKIIδ in HG group compared to CON group, which was reduced by 6-Diazo-5-oxo-L-norleucine (DON, an inhibitor of *O*-GlcNAcylation) treatment (Fig. 2D)." (lines 201-204 of the revised manuscript)

2) AIP: "Here, we evaluated the effects of CaMKIIδ *via* applying autocamtide-2-related inhibitory peptide (AIP, a CaMKIIδ inhibitor) and *O*-GlcNAcylation inhibitor DON." (lines 242-244 of the revised manuscript)

3) GDM: "However, the effect of *O*-GlcNAcylation of CaMKIIδ during gestational diabetes mellitus (GDM) and how CaMKIIδ activates fetal inflammation have not been investigated." (lines 102-104 of the revised manuscript)

Detailed Responses to Reviewer #3:

General Comments:

The authors investigate the mechanisms through which maternal diabetes during pregnancy causes cardiac remodeling and inflammation in the offspring's hearts. This study proposes an intriguing mechanism involving CaMKIIδ *O*-GlcNAcylation, which leads to mtDNA leakage and subsequent activation of the cGAS-STING pathway, driving cardiac remodeling. Although the human relevance is unclear, the conceptual framework is solid, and the authors present insightful data to support their hypothesis. However, the manuscript should be organized more effectively to enhance its flow and overall readability, and some figures and data appear unconvincing. Below are specific comments:

Response:

We thank you for the positive comments that "the conceptual framework is solid, and the authors present insightful data to support their hypothesis." We have followed your suggestions and made every possible effort to address the concerns. Detailed responses to all further comments are provided below.

General Comments:

Unconvincing representative images raise concerns about reproducibility.

More proper alignment between representative data and quantifications is needed.

Ensuring logical flow in figure presentation and improving the quality of supporting data will greatly enhance the manuscript's clarity and impact.

Response:

1) Thanks for your constructive comments. In response, we have carefully reviewed all the figures in our manuscript, and made revisions where necessary. These revisions include updates to Fig. 2A, 2D and 2E, etc. (Fig. 2A, 2D and 2E in the revised Manuscript, which is inserted below for the Reviewer's convenience).

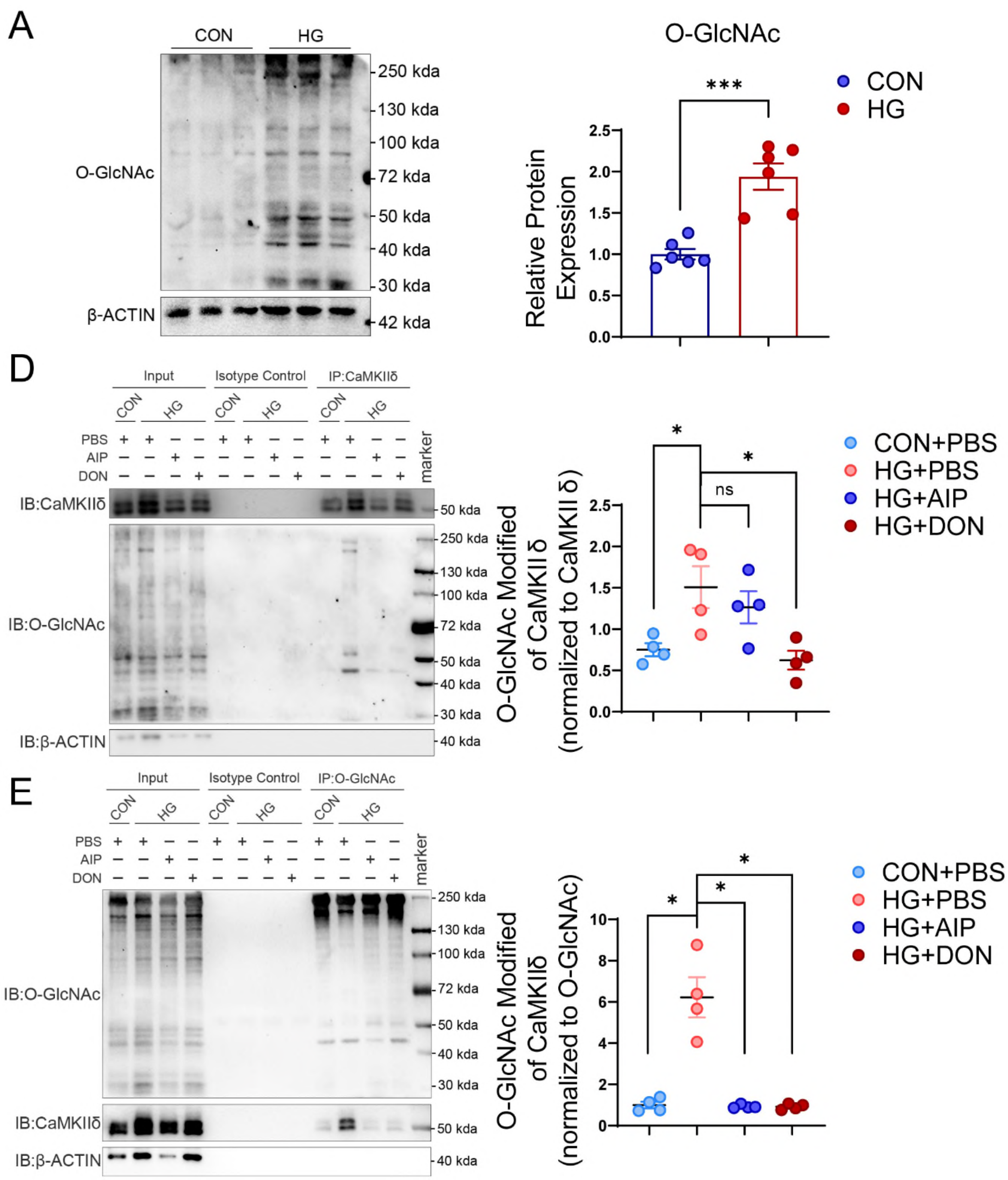

Fig. 2A and D-E: Assessment of global and CaMKIIδ-specific *O*-GlcNAcylation levels in NRCMs. *O*-GlcNAcylation of proteins was evaluated in E19.5 fetal rat hearts. CoIP experiments were performed using NRCMs isolated from E19.5 neonatal hearts, DON was applied on NRCMs at 50 μM for 48 hours, and AIP at 1 μM for 48 hours. (A) Overall protein *O*-GlcNAcylation levels in fetal hearts were detected by western blotting (left). Densitometric quantification of the blotting (right), n = 6 per group. (D) Western blotting of CoIP were performed for the CON+PBS, HG+PBS, HG+AIP, HG+DON groups (left). IP: CaMKIIδ. Statistical quantification of the blots (right), n = 4 per group. (E) Western blotting of CoIP were performed for the CON+PBS, HG+PBS, HG+AIP, HG+DON groups (left). IP: *O*-GlcNAc. Statistical quantification of the blots (right), n = 4 per group. For statistical comparisons, we initially evaluated data for normality and equality of variances. Student's *t* test and one-way analysis of variance (ANOVA) were used to determine the levels of statistical differences between two groups and among multiple groups, respectively. If equal variances were not assumed, Welch's *t* test or Welch's ANOVA were applied. Data were expressed as mean±SEM, *p<0.05, **p<0.01, ***p<0.001, ****p<0.0001. Abbreviations: IP, immunoprecipitation; IB, immunoblotting.

2) Sorry for the formatting issues related to the presentation of representative data and quantifications. To address this, we have revised the formatting throughout the manuscript.

3) Furthermore, to ensure better logical flow in figure presentation and improve the quality of supporting data, we have thoroughly reorganized the manuscript and implemented point-by-point revisions, as detailed below.

Reorganization:

(Q1) The authors start with methods, which deviates from the typical order of presentation in papers published in Nature Communications.

Response:

Thanks for your constructive comments. Following your suggestions, we have revised the manuscript to meet the formatting requirements of *Nature Communications,* including the following sections in order: a title page with author affiliations and contact information; the required sections for the content type; followed by References, Acknowledgements, Author Contributions, Competing Interests statement, and Figure Legends.

(Q2) The order of the figures (e.g., Fig. 6A-F appearing before Fig. 5K-O) makes it challenging to follow the story.

Response:

Thanks for your constructive comments. In response, we have revised the order of the figures and the corresponding **Results** sections accordingly. For example, the **Results** section in the revised manuscript describes Figure 5K to 6F as follows:

"Our data showed that CsA mitigated the decline in TMRM and mtDNA release associated with Camk2d overexpression in the HG group of NRCMs (Fig. 5K-N). Additionally, CsA reduced phosphorylation of STING, TBK1, and IRF3 proteins in Camk2d-overexpressing NRCMs under hyperglycemic conditions (Fig. 5O).

To obtain the direct evidence of CaMKIIδ inducing mtDNA release, we manipulated endonuclease G (Endog) in NRCMs under normoglycemic conditions. *Endog* is a mitochondrial intermembrane space located endonuclease which participates in apoptosis and inflammation by degrading DNA fragments[1]. *Endog*-/- mouse embryonic fibroblasts (MEFs) have higher levels of cmtDNA relative to wild-type MEFs[2]. Hence, the role of CaMKIIδ in regulating mtDNA release was further validated by co-transfecting *Endog* siRNA and *Camk2d* siRNA. Knockdown efficiencies of *Camk2d* (Supplementary Fig. 5A-C) and knockdown efficiencies of *Endog* (Supplementary Fig. 5D-F) were validated by mRNA and protein expression levels. Co-transfecting *Endog* siRNA and *Camk2d* siRNA led to a reduction in mtDNA release compared to transfection with *Endog* siRNA alone (Fig. 6A). Meanwhile, co-transfecting *Endog* siRNA and a Camk2d-overexpressing plasmid in NRCMs resulted in a marked increase in mtDNA release from mitochondrial matrix (Fig. 6A-B). qPCR results showed that cytosolic mtDNA levels were increased after *Endog* siRNA transfection (Fig. 6C), consistent with the enlarged images shown in Fig. 6A. *Endog* siRNA transfection in NRCMs led to increased phosphorylation of STING, TBK1, and IRF3 (Fig. 6D), effectively recapitulating the activation of downstream signaling pathways observed in hyperglycemia. NRCMs co-transfected with *Endog* siRNA and Camk2d overexpression plasmid showed elevated phosphorylation levels of STING, TBK1, and IRF3 (Fig. 6E). In contrast, NRCMs with *Endog* siRNA and *Camk2d* siRNA exhibited

decreased phosphorylation of STING, TBK1, and IRF3 (Fig. 6F)." (lines 308-331 of the revised manuscript)

References:

1. Wang, W., Li, J. & Zhou, Q. The biological function of cytoplasm-translocated ENDOG (endonuclease G). *Autophagy* **20**, 445-447 (2024).
2. Kim, J. *et al.* VDAC oligomers form mitochondrial pores to release mtDNA fragments and promote lupus-like disease. *Science* **366**, 1531-1536 (2019).

Data and Figures:

(Q1) Fig. 2D and E: These images do not clearly demonstrate *O*-GlcNAcylation in CaMKIIδ.

Response:

Thank you for your constructive suggestion. To further confirm the role of CaMKIIδ *O*-GlcNAcylation in intrauterine hyperglycemia, we performed a series of additional experiments as follows:

1) According to your suggestions, we revised Figure 2D and 2E with more representative images and conducted further statistical analysis of the data to confirm the occurrence of CaMKIIδ *O*-GlcNAcylation in fetal cardiomyocytes subjected to *in utero* hyperglycemia, as well as the inhibitory effects of AIP and DON on CaMKIIδ *O*-GlcNAcylation. The results demonstrated a significant increase in CaMKII *O*-GlcNAcylation in NRCMs from the HG+PBS group, which was effectively attenuated by DON treatment (Figure 2D and 2E in the revised Manuscript, which is inserted below for the Reviewer's convenience).

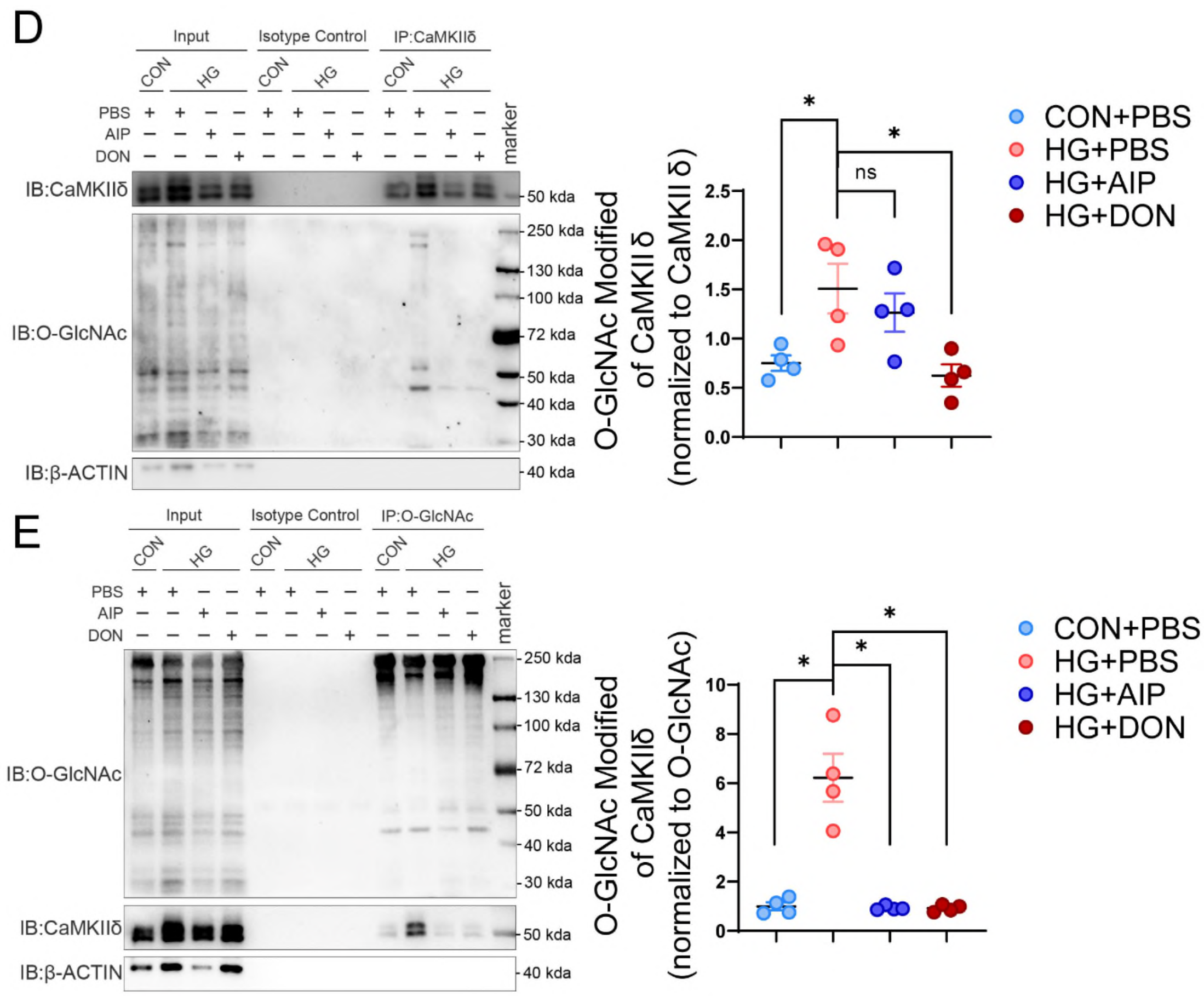

Figure 2D and 2E: CoIP western blotting and analysis of CaMKIIδ *O*-GlcNAcylation. CoIP experiments were performed using NRCMs isolated from E19.5 neonatal hearts, DON was applied on NRCMs at 50 μM for 48 hours, and AIP at 1 μM for 48 hours. (D) Western blotting of CoIP were performed for the CON+PBS, HG+PBS, HG+AIP, HG+DON groups (left). IP: CaMKIIδ. Statistical quantification of the blots (right), n = 4 per group. (E) Western blotting of CoIP were performed for the CON+PBS, HG+PBS, HG+AIP, HG+DON groups (left). IP: *O*-GlcNAc. Statistical quantification of the blots (right), n = 4 per group. For statistical comparisons, data were initially evaluated for normality and equality of variances. One-way analysis of variance (ANOVA) was used to determine statistical differences among multiple groups. If equal variances were not assumed, Welch's ANOVA was applied. Data were expressed as mean±SEM, *p<0.05, **p<0.01, ***p<0.001, ****p<0.0001. Abbreviations: IP, immunoprecipitation; IB, immunoblotting.

2) To validate the role of CaMKIIδ *O*-GlcNAcylation *in vivo*, we engineered two cardiac-specific adenoviral vectors to dissect the functional impact of CaMKIIδ *O*-GlcNAcylation:

i) pADV-cTNT-Camk2d (p.S280A)-HA (abbreviated as AdV-CaMKIIδ-S280A-OE): To preserve kinase activity while blocking hyperglycemia-induced post-translational modification, we overexpressed a CaMKIIδ mutant with abolished *O*-GlcNAcylation at Ser280 (S280A mutation).

ii) pADV-cTNT-mCherry-3xFLAG-shCamk2d (abbreviated as AdV-shCaMKIIδ): To isolate the effects of *O*-GlcNAcylation from baseline CaMKIIδ signaling, we knocked down endogenous CaMKIIδ (58% reduction in protein levels, $p = 0.0004$).

Simultaneous intramyocardial injection of these two adenoviral vectors into neonatal rat hearts resulted in a specific reduction of CaMKIIδ *O*-GlcNAcylation in *utero* hyperglycemia.

Next, to verify changes in CaMKIIδ *O*-GlcNAcylation following *in vivo* gene interventions, we isolated NRCMs from hearts injected with adenoviral vectors and performed CoIP experiments. According to your suggestions, we performed CoIP experiments using a crosslink IP kit (Thermo Scientific, USA, Cat#88848), which covalently immobilized the antibody to magnetic beads. This method ensured that the eluted samples used for subsequent western blotting were free of antibody-derived IgG contamination. As shown in Supplementary Fig. 9, overexpression of the CaMKIIδ S280A mutant effectively reduced cardiac CaMKIIδ *O*-GlcNAcylation in offspring subjected to intrauterine hyperglycemia. Moreover, the S280A mutant reduced CaMKIIδ activation in response to intrauterine hyperglycemia (inserted below for the Reviewer's convenience).

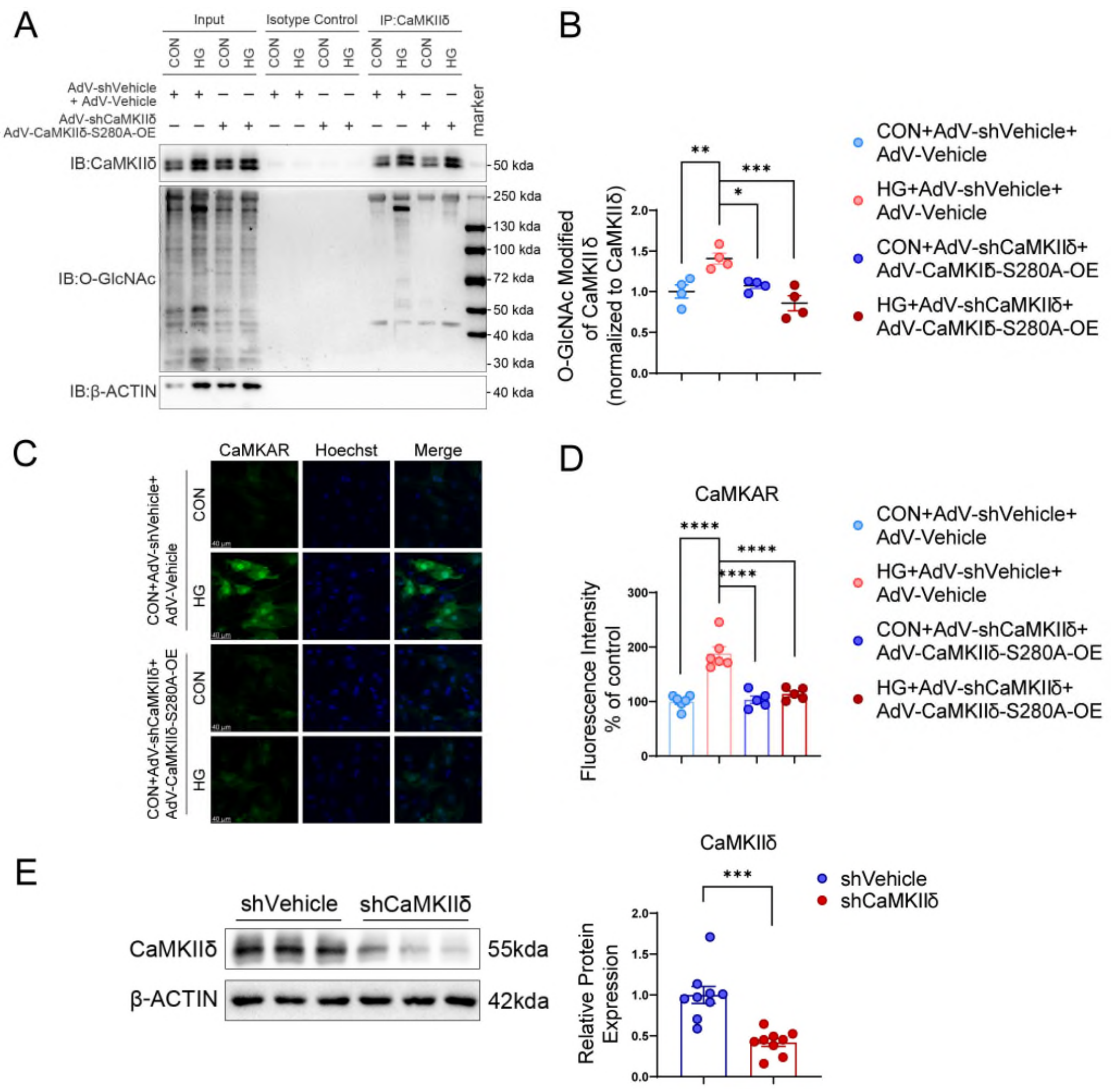

Supplementary Fig. 9: S280A mutation of CaMKIIδ abolished cardiac CaMKIIδ *O*-GlcNAcylation and reduced CaMKIIδ activation. Neonatal rats received injections on postnatal day 3 and were euthanized on postnatal day 7. CoIP experiments were performed using heart tissues from P7 neonatal rats. CaMKAR fluorescence imaging experiments were conducted using NRCMs isolated from P7 neonatal hearts. (A) Western blotting analysis of CoIP were measured for the CON+AdV-shVehicle+AdV-Vehicle, HG+AdV-shVehicle+AdV-Vehicle, CON+AdV-shCaMKIIδ+AdV-CaMKIIδ-S280A-OE, HG+AdV-shCaMKIIδ+AdV-CaMKIIδ-S280A-OE groups, IP: CaMKIIδ, n = 4 per group. (B) The statistically quantified data on the western blotting analysis of *O*-GlcNAc modified CaMKIIδ. (C) Fluorescence images of cardiomyocytes infected with CaMKAR for 24 hours and stained with Hoechst 33342 for 20 min, scale bar = 40 μm. (D) The statistically quantified data on the cellular

fluorescence intensity after infected with CaMKAR, n = 5-6 per group. (E) Statistical quantification of CaMKIIδ protein levels following injection of AdV-shCaMKIIδ, n = 9 per group. For statistical comparisons, data were initially evaluated for normality and equality of variances. Student's *t* test and one-way ANOVA were used to determine the levels of statistical differences between two groups and among multiple groups, respectively. If equal variances were not assumed, Welch's *t* test or Welch's ANOVA were applied. Data were expressed as mean±SEM, *p<0.05, **p<0.01, ***p<0.001, ****p<0.0001. Abbreviations: IP, immunoprecipitation; IB, immunoblotting.

3) To investigate *in vivo* changes in cardiac function following AdV-CaMKIIδ-S280A overexpression, we conducted a series of histological analyses and western blotting experiments. As shown in Supplementary Fig. 10, overexpression of the CaMKIIδ S280A mutant effectively attenuated myocardial fibrosis in offspring subjected to intrauterine hyperglycemia. Moreover, the S280A mutant reduced cardiomyocyte cross-sectional area in the HG group. Additionally, the expression levels of ANP and BNP were decreased in the HG+AdV-shCaMKIIδ+AdV-CaMKIIδ-S280A-OE group compared to the HG+AdV-shVehicle+AdV-Vehicle group. Notably, plasma mtDNA levels were also reduced in the HG+AdV-shCaMKIIδ+AdV-CaMKIIδ-S280A-OE group (inserted below for the Reviewer's convenience). We hope that these additional data address your concerns and help further strengthen the study.

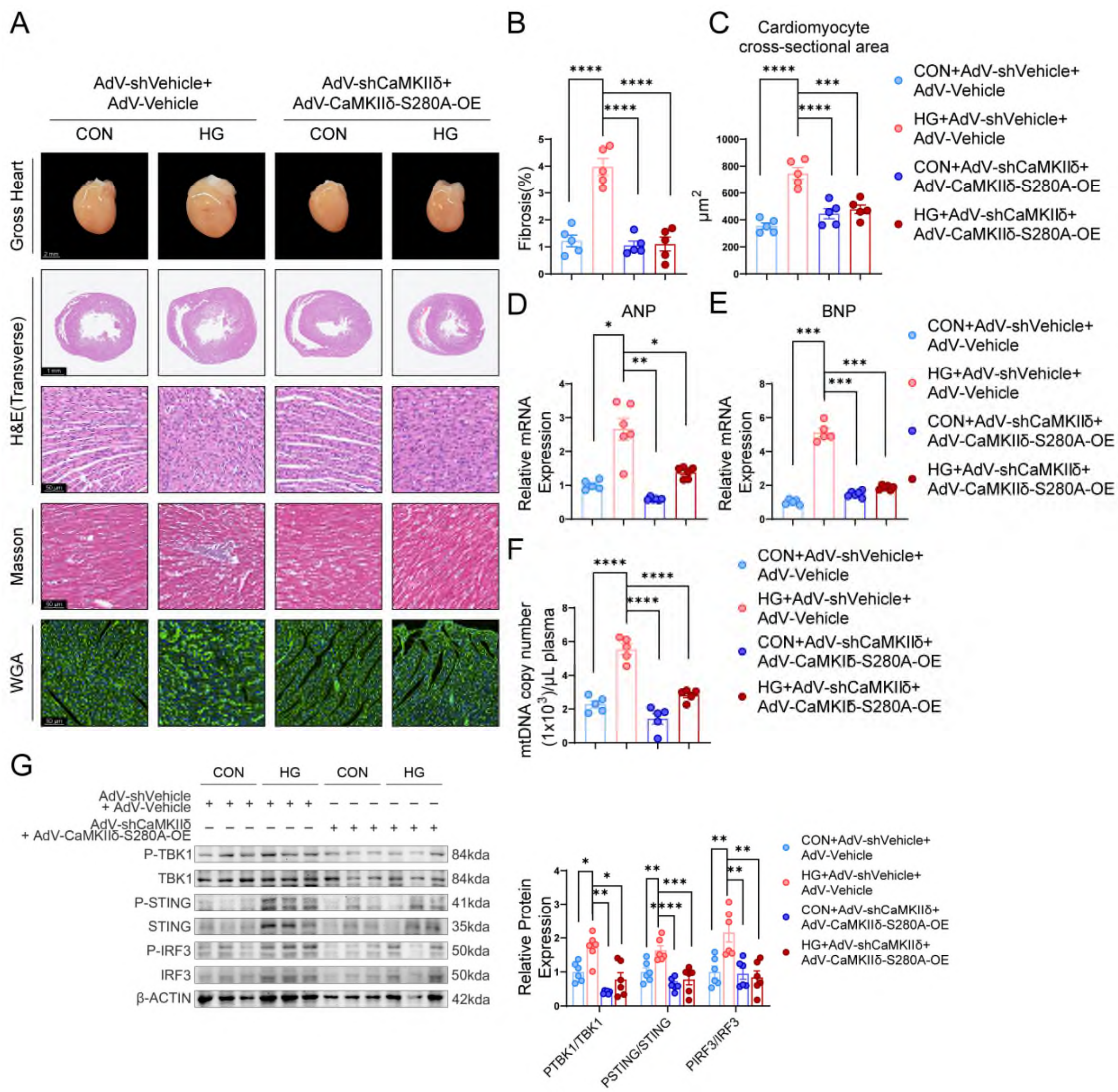

Supplementary Fig. 10: S280A mutation of CaMKIIδ abolishes cardiac CaMKIIδ *O*-

GlcNAcylation in offspring subjected to intrauterine hyperglycemia. Neonatal rats received injections on postnatal day 3 and were euthanized on postnatal day 7. All experiments in Supplementary Fig. 10 were performed using heart tissues from P7 neonatal rats, except for the plasma mtDNA copy number experiment. (A) Echocardiography, gross morphology and histological analyses were conducted for multiple groups of fetal hearts. Gross morphology, scale bar = 2.0 mm; HE, Masson and WGA staining, scale bar = 50 μm. (B) Quantitative analysis was performed on fibrotic area (Masson staining), n = 5 per group. (C) Quantitative analysis was performed on cardiomyocyte cross-sectional area (WGA staining), n = 5 per group. (D) ANP gene expression was quantified by RT-qPCR, n = 5-6 per group. (E) BNP gene expression was quantified by RT-qPCR, n = 5-6 per group. (F) Plasma mtDNA copy number was assessed, n = 5 per group. (G) Phosphorylated and total protein levels in hearts from multiple groups were detected by western blotting (left). Densitometric quantification of the blots (right), n = 6 per group. For statistical comparisons, data were initially evaluated for normality and equality of variances. One-way ANOVA was used to determine statistical differences among multiple groups. If equal variances were not assumed, Welch's ANOVA was applied. Data were expressed as mean±SEM, *p<0.05, **p<0.01, ***p<0.001, ****p<0.0001.

(Q2) Fig. 2H and J: The labels are too small, making the data difficult to interpret.

Response:

Thank you for your suggestion. In response, we have revised Fig. 2H and 2J for clarity and added Supplementary Table1 listing the 70 overlapping genes in Fig. 2J. (Figure 2H-J in the revised Manuscript, which is inserted below for the Reviewer's convenience). Furthermore, we have revised all figures in the manuscript to address similar issues.

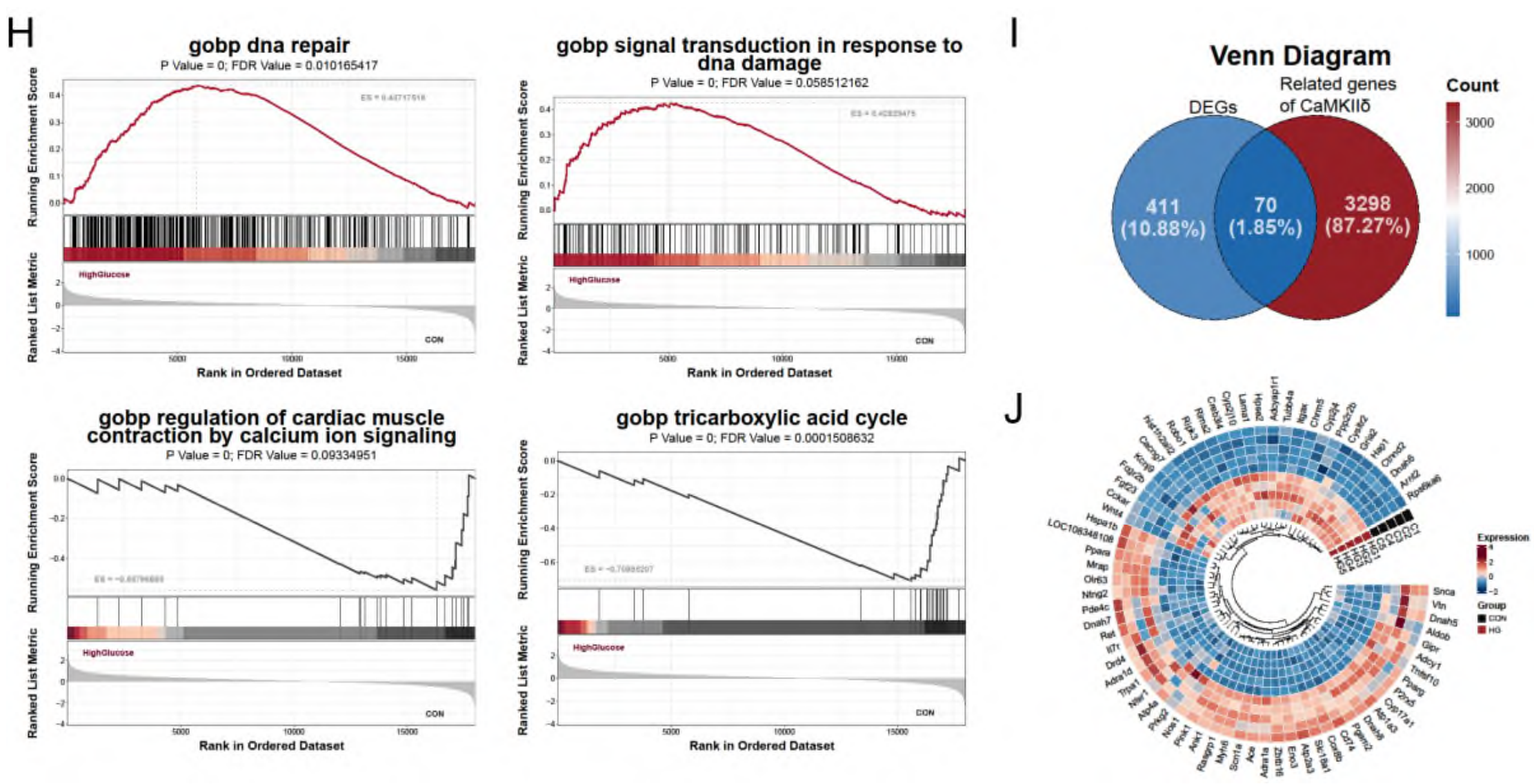

Figure 2H-J: HG enhanced *O*-GlcNAcylation of CaMKIIδ in HG fetal hearts. (H) The diagram indicated the enriched pathways of DEGs, using GSEA of biological processes. (I) Venn diagram indicated the overlap of 70 genes between genes associated with CaMKIIδ in KEGG (red) and DEGs between CON and HG groups (blue). (J) Heatmap of the overlap of 70 genes. Abbreviations: CON, control group; HG, high glucose group.

(Q3) AIP and DON explanations: The explanation for these compounds appears after their initial presentation in the data, disrupting the logical flow.

Response:

Thank you for your constructive comments. In response, we have now clearly defined both

abbreviations upon their initial introduction in the revised manuscript:

1) "Then, coimmunoprecipitation (CoIP) experiments confirmed that elevated *O*-GlcNAcylation levels of CaMKIIδ in HG group compared to CON group, which was reduced by 6-Diazo-5-oxo-L-norleucine (DON, an inhibitor of *O*-GlcNAcylation) treatment (Fig. 2D)." (lines 201-204 of the revised manuscript)

2) "Here, we evaluated the effects of CaMKIIδ via applying autocamtide-2-related inhibitory peptide (AIP, a CaMKIIδ inhibitor) and *O*-GlcNAcylation inhibitor DON." (lines 242-244 of the revised manuscript)

(Q4) Fig. 3F: No significant difference is observed in mitosox between HG and HG+AIP conditions.

Response:

Thank you for your constructive comments. In response, we replicated experiments in Fig. 3F and confirmed that the statistical conclusions were consistent with those in the original manuscript. Given the consistency of the results, we have replaced the previous images with more representative ones in the revised Manuscript. (Fig. 3F-H in the revised Manuscript, which is inserted below for the Reviewer's convenience). The raw images and quantification data used for analysis have been provided in the source data files.

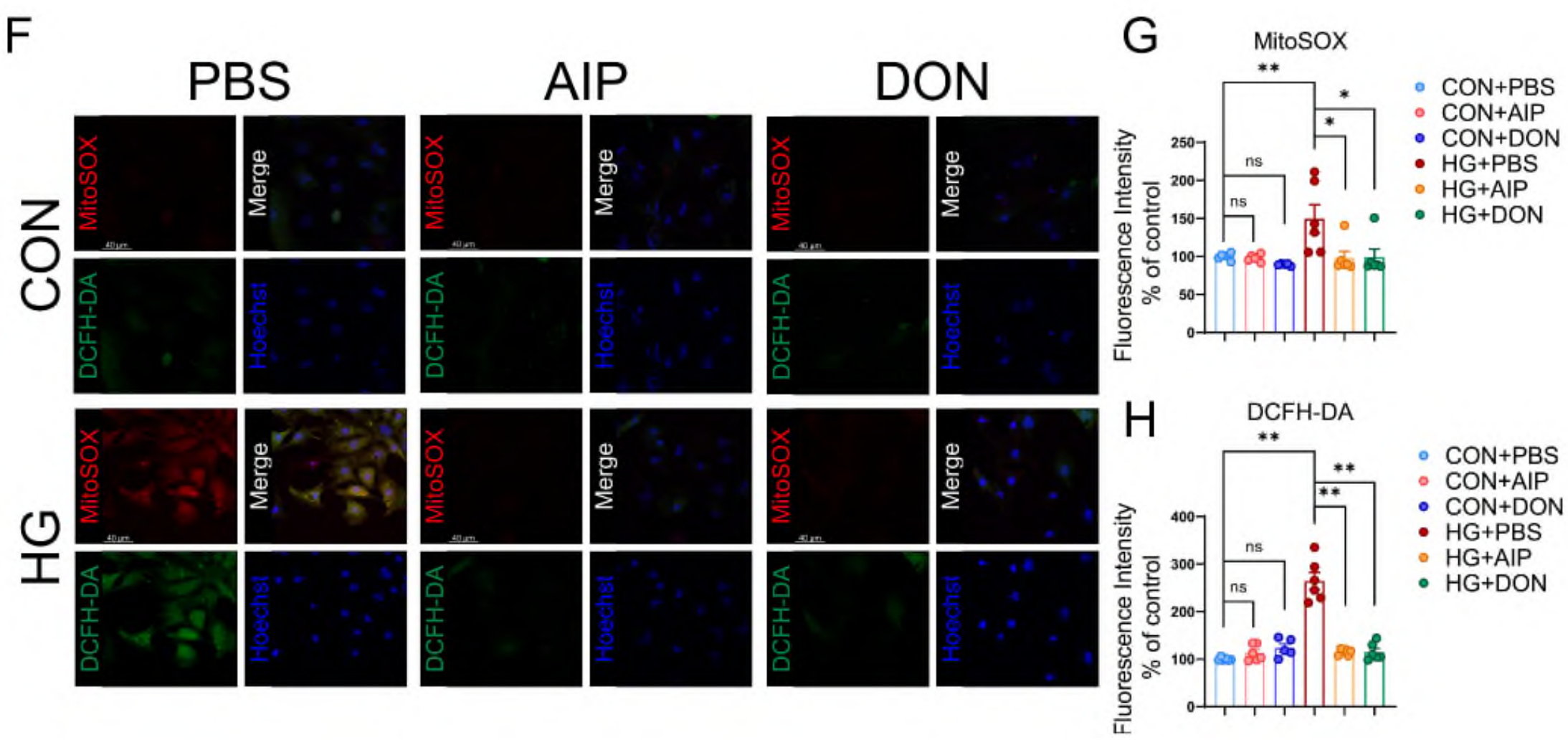

Fig. 3F-H: Assessment of intracellular and mitochondrial ROS levels in NRCMs. Experiments were conducted using NRCMs isolated from E19.5 neonatal hearts. DON was applied on NRCMs at 50 μM for 48 hours, and AIP at 1 μM for 48 hours. (F) Fluorescence images of cardiomyocytes stained with DCFH-DA, MitoSOX and Hoechst33342 for 20 mins. Before staining, cardiomyocytes were pretreated with PBS or AIP or DON for 48 h, scale bar = 40 μm. (G) The statistically quantified data on the fluorescence intensity after staining with MitoSOX, n = 5-6 per group. (H) The statistically quantified data on the cellular fluorescence intensity after staining with DCFH-DA, n = 5-6 per group. For statistical comparisons, we initially evaluated data for normality and equality of variances. One-way ANOVA was used to determine the levels of statistical differences among multiple groups. If equal variances were not assumed, Welch's ANOVA was applied. Data were expressed as mean±SEM, *p<0.05, **p<0.01, ***p<0.001, ****p<0.0001.

(Q5) Fig. 3I: Are the imaging conditions consistent? In some figures, the signal appears saturated, and the mitotracker staining is unclear in HG+.

Response:

Thank you for your comments. We confirm that all imaging conditions in Fig. 3I were strictly consistent. To address the issue of signal saturation, we conducted replicate experiments and confirmed the original findings. Accordingly, more representative images have been used in the revised Manuscript. (Fig. 3I in the revised Manuscript, which is inserted below for the Reviewer’s convenience). The raw images and quantification data used for analysis have been provided in the source data files.

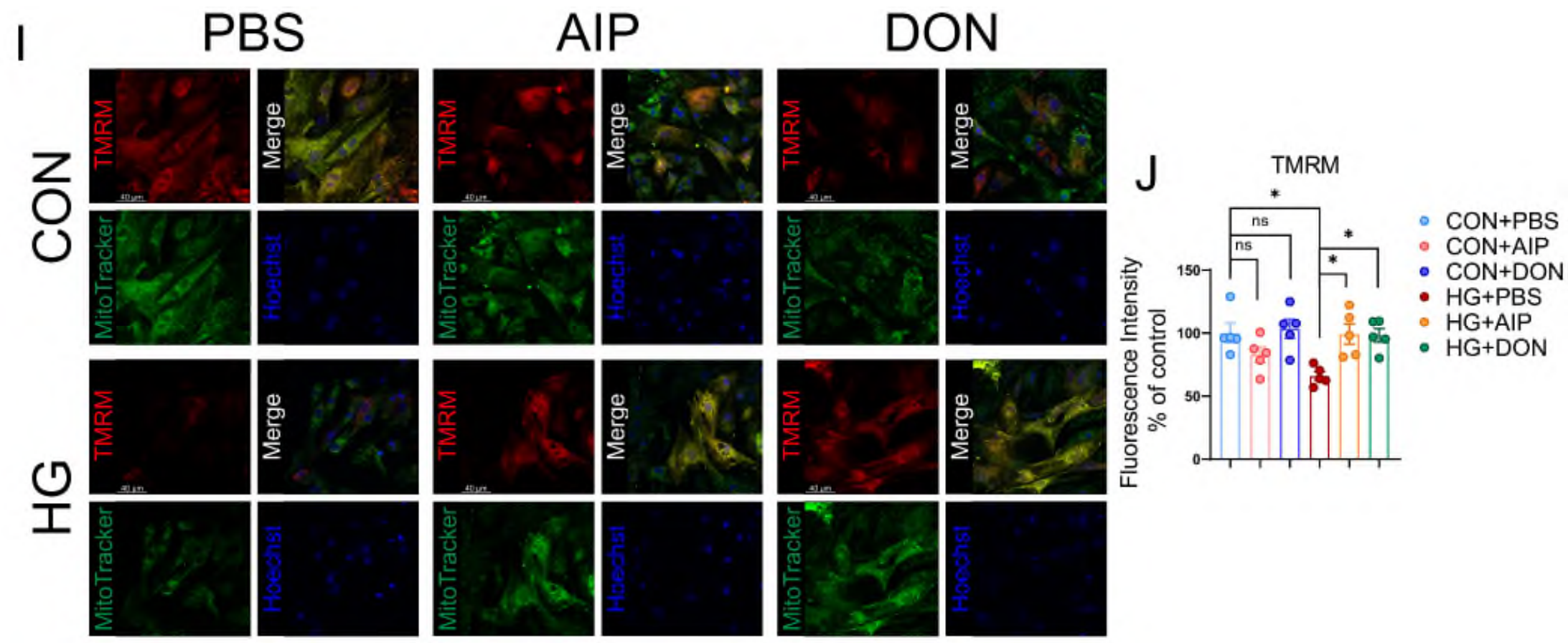

Fig. 3I-J: Assessment of mitochondrial membrane potential (ΔΨm) levels in NRCMs. Experiments were conducted using NRCMs isolated from E19.5 neonatal hearts. DON was applied on NRCMs at 50 μM for 48 hours, and AIP at 1 μM for 48 hours. (I) Fluorescence images of cardiomyocytes stained with TMRM, MitoTracker and Hoechst33342 for 20 mins, scale bar = 40 μm. (J) The statistically quantified data on the cellular fluorescence intensity after staining with TMRM, n = 5 per group. For statistical comparisons, we initially evaluated data for normality and equality of variances. One-way ANOVA was used to determine the levels of statistical differences among multiple groups. If equal variances were not assumed, Welch’s ANOVA was applied. Data were expressed as mean±SEM, *$p<0.05$, **$p<0.01$, ***$p<0.001$, ****$p<0.0001$.

(Q6) Fig. 3H: The representative images do not align with the quantification data; HG and HG+AIP appear similar.

Response:

Thank you for your constructive suggestions. In response, more representative images have been used in the revised Manuscript (Fig. 3F-H in the revised Manuscript, which is inserted below for the Reviewer’s convenience). The raw images and quantification data used for analysis have been provided in the source data files.

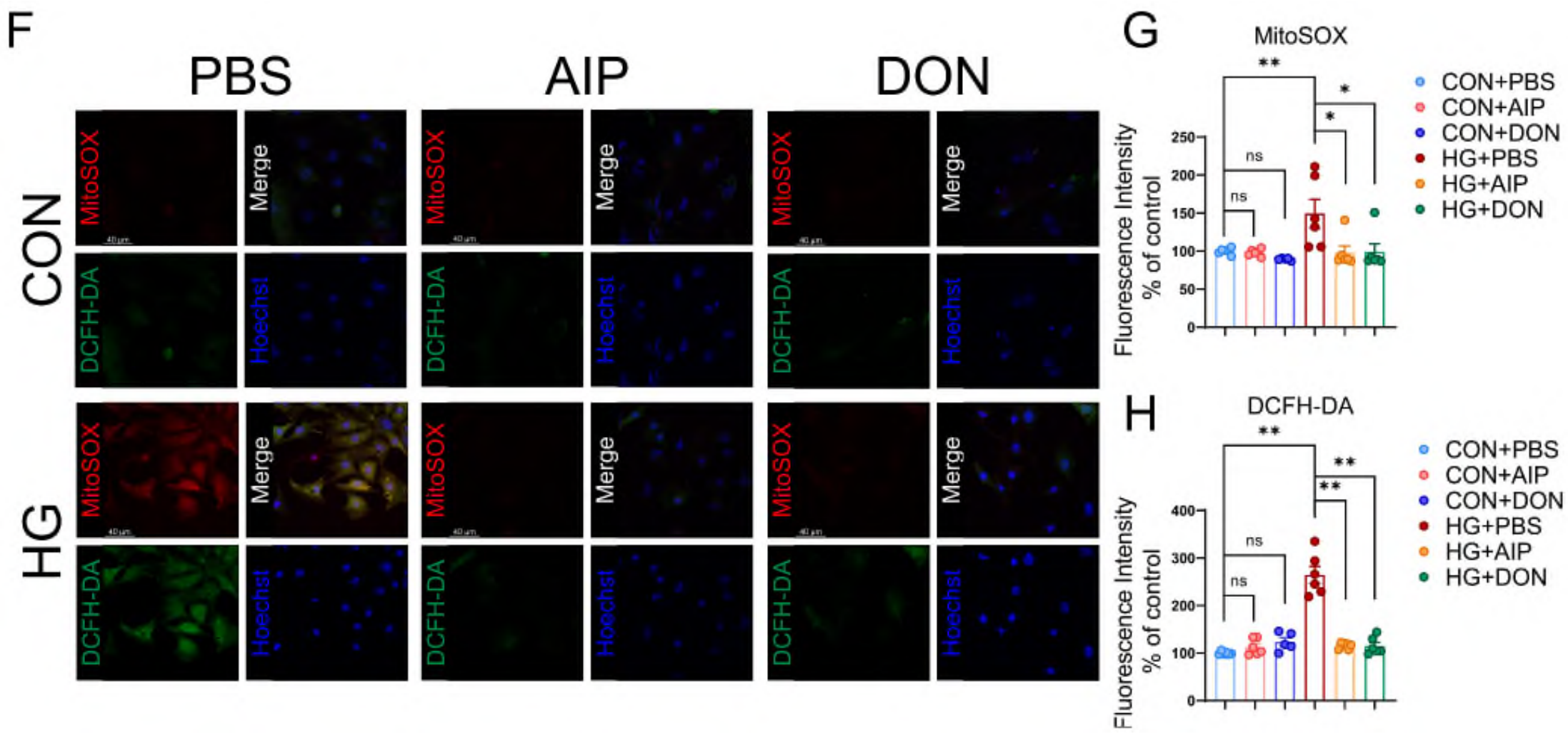

Fig. 3F-H: Assessment of intracellular and mitochondrial ROS levels in NRCMs. Experiments were conducted using NRCMs isolated from E19.5 neonatal hearts. DON was applied on NRCMs at 50 µM for 48 hours, and AIP at 1 µM for 48 hours. (F) Fluorescence images of cardiomyocytes stained with DCFH-DA, MitoSOX and Hoechst33342 for 20 mins. Before staining, cardiomyocytes were pretreated with PBS or AIP or DON for 48 h, scale bar = 40 µm. (G) The statistically quantified data on the fluorescence intensity after staining with MitoSOX, n = 5-6 per group. (H) The statistically quantified data on the cellular fluorescence intensity after staining with DCFH-DA, n = 5-6 per group. For statistical comparisons, we initially evaluated data for normality and equality of variances. One-way ANOVA was used to determine the levels of statistical differences among multiple groups. If equal variances were not assumed, Welch's ANOVA was applied. Data were expressed as mean±SEM, *p<0.05, **p<0.01, ***p<0.001, ****p<0.0001.

(Q7) Fig. 5C: One of the figure borders is missing.

__Response__:

Thank you for pointing out this error. In response, we have corrected the missing border (Fig. 5C in the revised Manuscript, which is inserted below for the Reviewer's convenience).

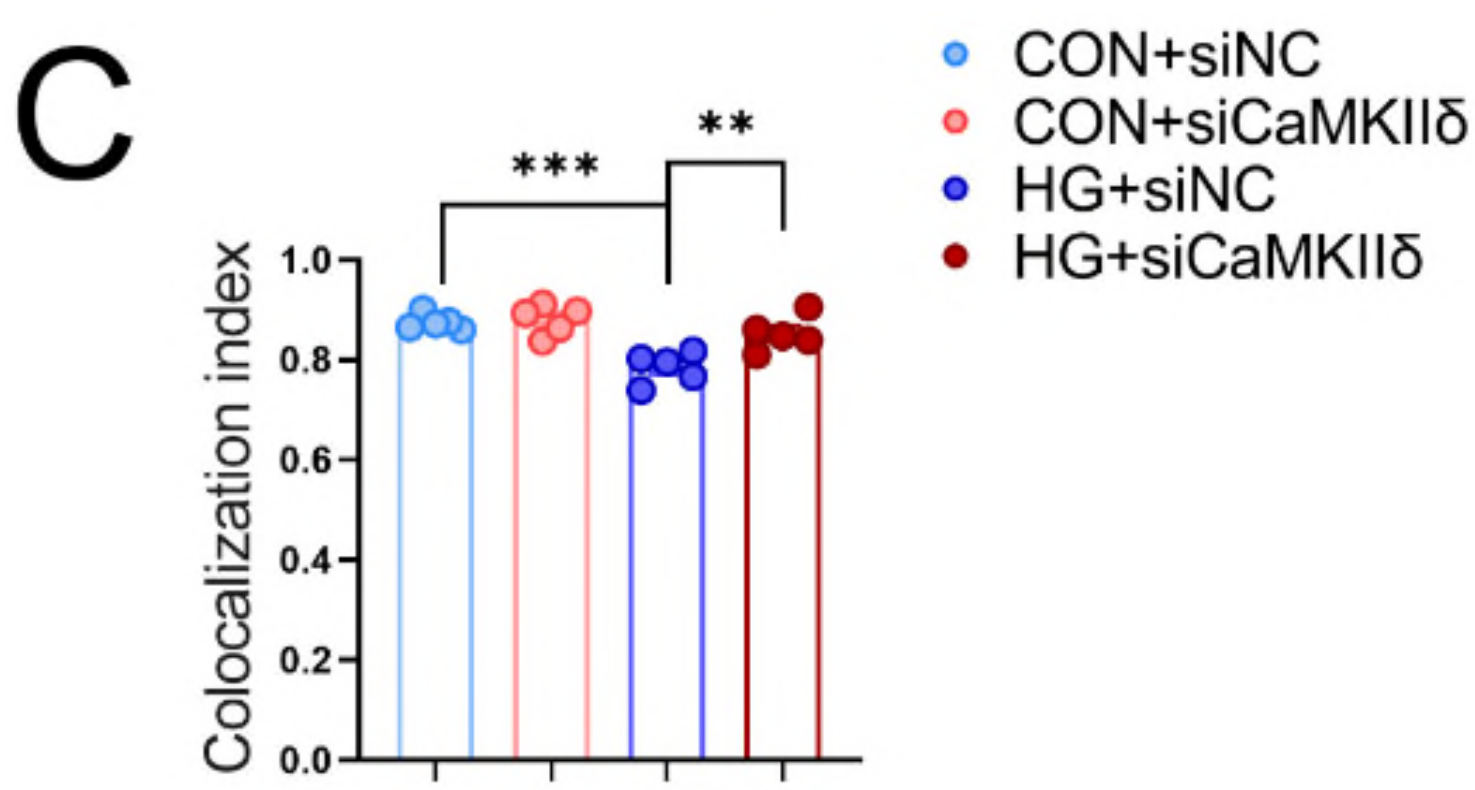

Fig. 5C: The colocalization index of dsDNA and mitochondria in panel (D) was quantified based on Manders' coefficient index, n = 5 per group. For statistical comparisons, we initially evaluated data for normality and equality of variances. One-way ANOVA was used to determine the levels of statistical differences among multiple groups. If equal variances were not assumed, Welch's ANOVA was applied. Data were expressed as mean±SEM, *p<0.05, **p<0.01, ***p<0.001, ****p<0.0001.

(Q8) Fig. 5E and J: The β-actin levels differ significantly, suggesting variations in protein loading. Baseline variations make the interpretation of the blots unclear.

<u>Response</u>:

Thank you for your constructive comments. To address this concern, we conducted replicate western blotting experiments for Fig. 5E and J, and included more representative images in the revised manuscript (Fig. 5E and J in the revised Manuscript, which is inserted below for the Reviewer's convenience). Full-membrane images and quantification data are provided in the source data files.

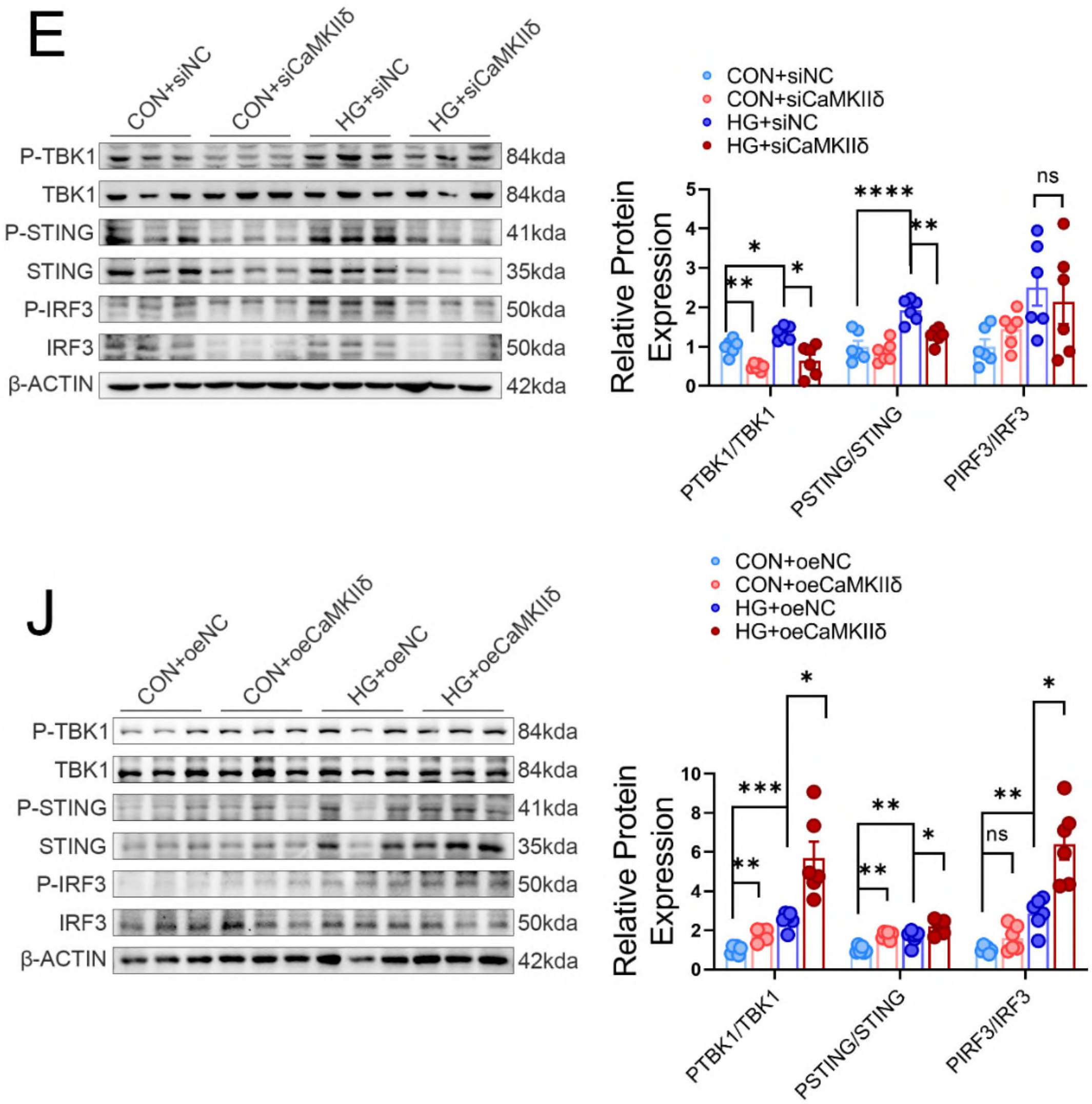

Figure 5E and J: Phosphorylated and total protein levels in NRCMs. Experiments were performed using NRCMs isolated from E19.5 rat hearts. CaMKIIδ siRNA or CaMKIIδ overexpression plasmid was transfected into NRCMs for 48 hours. DON was applied on NRCMs at 50 μM for 48 hours, and AIP at 1 μM for 48 hours. CsA was applied on NRCMs at 10 μM for the final 4 hours. (E) Phosphorylated and total protein levels in cardiomyocytes from multiple groups were detected by western blotting (left). Densitometric quantification of the blots (right), n = 6 per group. (J) Phosphorylated and total protein levels in cardiomyocytes from multiple groups were detected by western blotting (left). Densitometric quantification of the blots (right), n = 6 per group. For statistical comparisons, we initially evaluated data for normality and equality of variances. One-way ANOVA was used to determine the levels of statistical differences among multiple groups. If equal variances were not assumed, Welch's ANOVA was applied.

Data were expressed as mean±SEM, *p<0.05, **p<0.01, ***p<0.001, ****p<0.0001.

(Q9) Fig. 5E, J, and O: The changes in p-IRF3/IRF3 are not significant due to substantial IRF3 upregulation. The authors should consider an alternative method of quantification.

<u>Response</u>:

We appreciated your insightful suggestions. To exclude the influence of substantial IRF3 upregulation, we further performed ELISA to quantify the changes in P-IRF3. As shown in Supplementary Fig. R1, CaMKIIδ overexpression increased phosphorylation of IRF3 proteins. Moreover, this impact can be reversed by CsA, an established mPTP inhibitor (Supplementary Fig. R1, inserted below for the Reviewer's convenience).

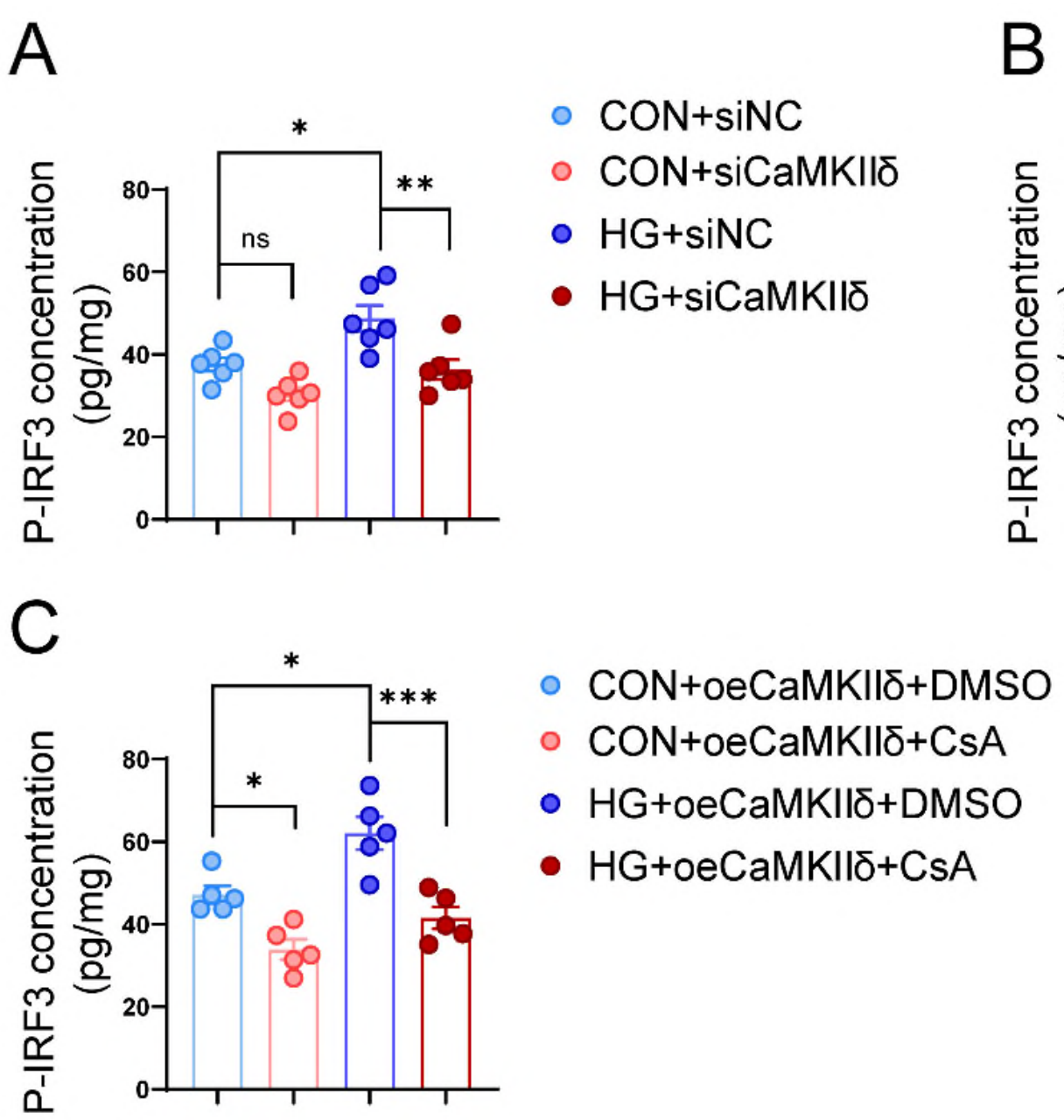

Supplementary Fig. R1. (A-C) Quantification of p-IRF3 protein expression among different groups *via* ELISA test, n = 5-6 per group. For statistical comparisons, we initially evaluated data for normality and equality of variances. One-way ANOVA was used to determine the levels of statistical differences among multiple groups. If equal variances were not assumed, Welch's t test was applied. Data were expressed as mean±SEM, *p<0.05, **p<0.01, ***p<0.001, ****p<0.0001 vs. CON.

(Q10) Fig. 6A: The representative images of siEndog do not demonstrate the quantification as depicted in Fig. 6B and C. Knockdown efficiency for siEndog is missing.

<u>Response</u>:

Sorry for the confusion. Fig. 6A presents immunofluorescence staining of NRCMs, where Tom20 (green) indicates mitochondria and dsDNA (red) indicates the distribution of mitochondrial DNA. As shown in the "zoom" images of Fig. 6A, dsDNA in the vehicle group largely colocalizes with mitochondria. In contrast, in the siEndog and siEndog+oeCaMKIIδ groups, a portion of the dsDNA appears to be released from the mitochondria, as indicated by the white arrowheads outside the Tom20 regions. In Fig. 6B, we quantified the colocalization between the red and green signals, rather than fluorescence

intensity. Therefore, the representative images of siEndog demonstrate the quantification depicted in Fig. 6B and C. The legend of Fig. 6A has been revised for clarity (Fig. 6A-C in the revised Manuscript, which is inserted below for the Reviewer's convenience).

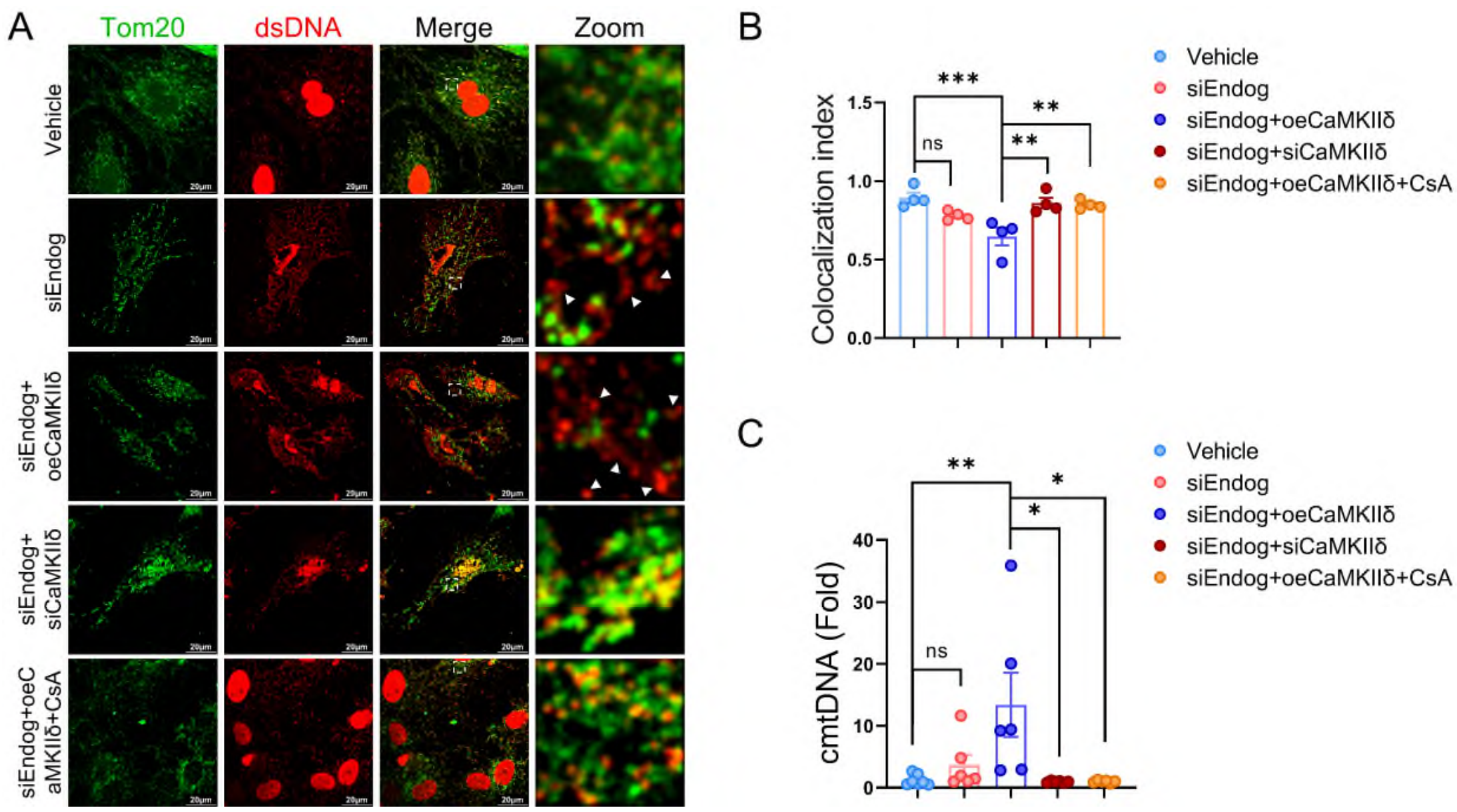

Figure 6A-C: *O*-GlcNAcylation promotes mtDNA release and subsequent inflammatory response in hyperglycemia through CaMKIIδ regulating mPTP opening. Experiments were performed using NRCMs isolated from E19.5 rat hearts. CaMKIIδ siRNA or CaMKIIδ overexpression plasmid was transfected into NRCMs for 48 hours. CsA was applied on NRCMs at 10 μM for the final 4 hours. (A) Cardiomyocytes were stained with Tom20 (green) and dsDNA (red) in all four groups, scale bar = 20 μm. White arrows denoted mtDNA released from mitochondria. (B) The colocalization index of dsDNA and mitochondria in panel (A) was quantified based on Manders' coefficient index, n = 4 per group. (C) Cytoplasmic mtDNA release was assessed by qPCR, n = 6-7 per group. For statistical comparisons, we initially evaluated data for normality and equality of variances. One-way ANOVA was used to determine the levels of statistical differences among multiple groups. If equal variances were not assumed, Welch's *t* test was applied. Data were expressed as mean±SEM, *$p<0.05$, **$p<0.01$, ***$p<0.001$, ****$p<0.0001$ vs. CON.

Additionally, a supplementary figure has been included to show the knockdown efficiency of Endog siRNA. The results demonstrate a reduction in both Endog mRNA and protein levels following transfection (Supplementary Fig. 5D-F in the revised Manuscript, which is inserted below for the Reviewer's convenience).

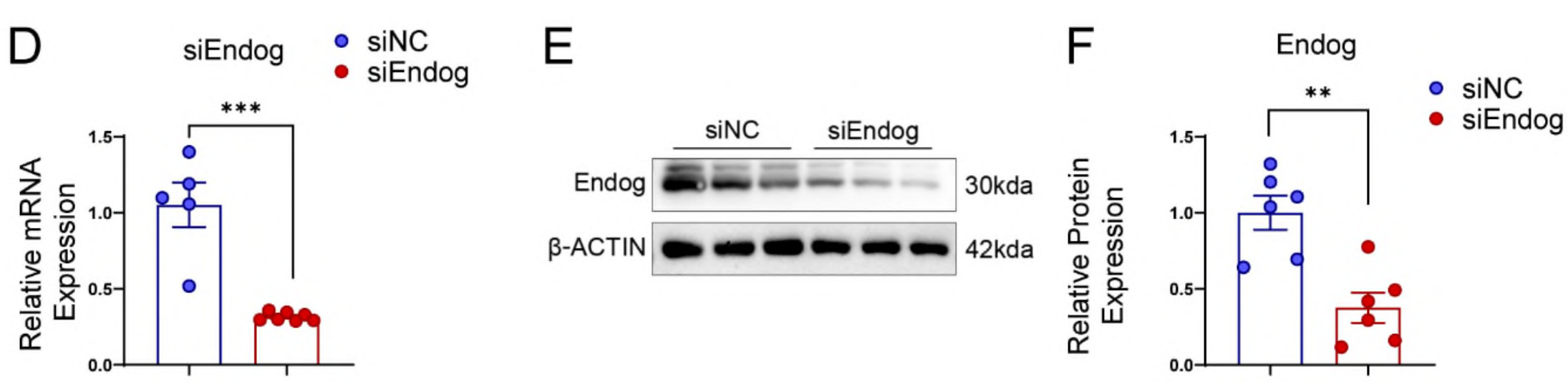

Supplementary Fig. 5D-F: Evaluation of siRNA knockdown efficiency and inhibitor efficacy in NRCMs. Experiments were performed using NRCMs isolated from E19.5 rat hearts. Endog siRNA was transfected into NRCMs for 48 hours. (D) mRNA expression of *Endog* after *Endog* siRNA transfection, n = 5-7 per group. (E) Representative immunoblots showing Endog protein levels after Endog siRNA transfection. (F) Quantification of Endog protein expression in panel E, n = 6 per group. For statistical comparisons, we initially evaluated data for normality and equality of variances. Statistical significance

was assessed using two-tailed unpaired Student's *t* test. If equal variances were not assumed, Welch's *t* test was applied. Data were expressed as mean±SEM, *p<0.05, ***p*<0.01, ****p*<0.001, *****p*<0.0001 vs. CON.

(Q11) Fig. 6E: The β-actin signal is very weak, raising concerns about protein loading amount.

Response:

Thank you for your constructive comments. The weak β-actin signal observed in the original figure was likely due to multiple uses of the stripping buffer (which removed previously bound antibodies for re-probing). To address this concern, we conducted replicate western blotting experiments for Figure 6E, and included more representative images in the revised manuscript. NRCMs co-transfected with *Endog* siRNA and CaMKIIδ overexpression plasmid showed elevated phosphorylation levels of STING, TBK1, and IRF3 (Fig. 6E in the revised Manuscript, which is inserted below for the Reviewer's convenience). Full-membrane images and quantification data are provided in the source data files.

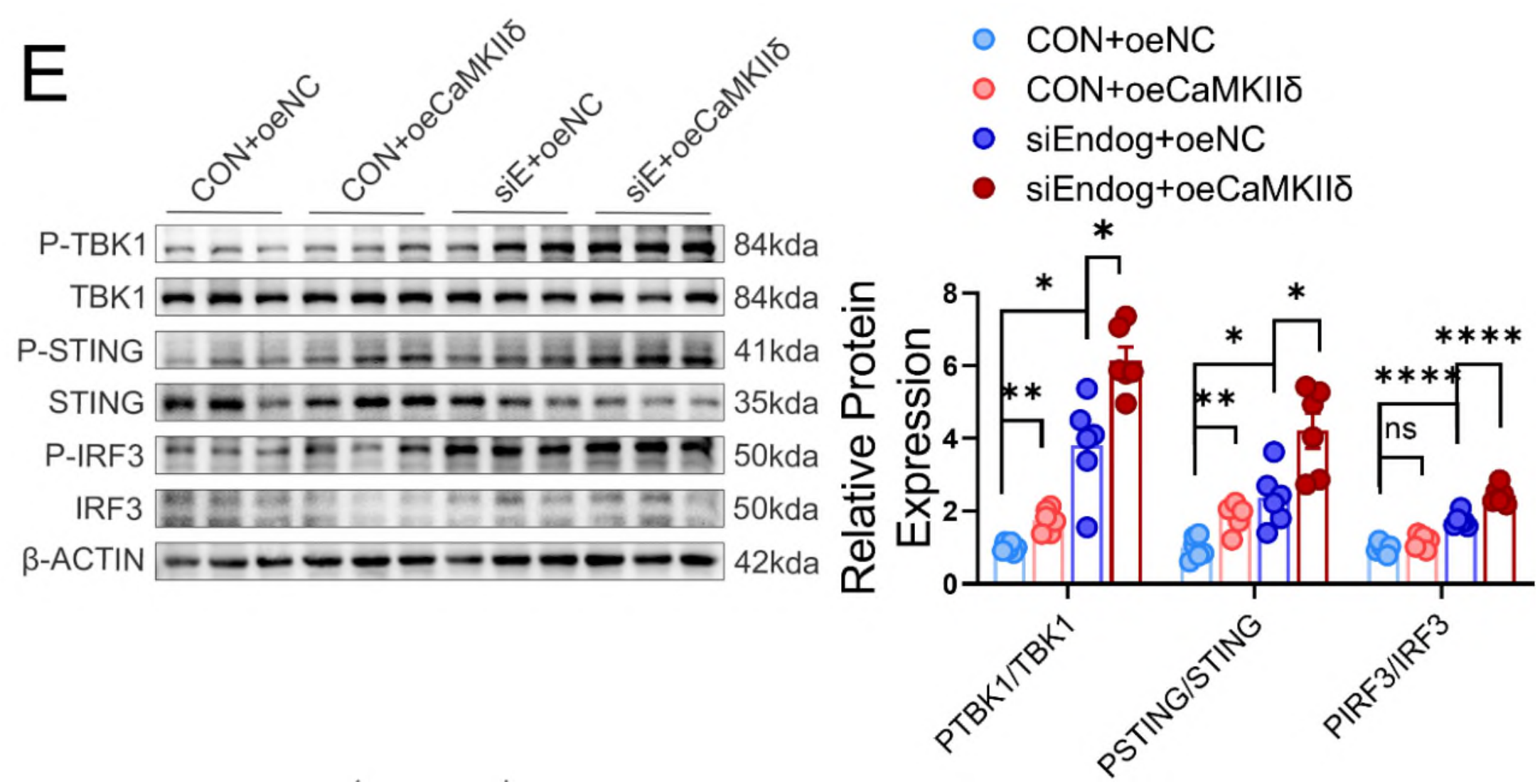

Figure 6E. *O*-GlcNAcylation promoted subsequent inflammatory response in hyperglycemia through CaMKIIδ. Experiments were performed using NRCMs isolated from E19.5 rat hearts. *Endog* siRNA or CaMKIIδ overexpression plasmid was transfected into NRCMs for 48 hours. (E) Representative immunoblots and analyses of phosphorylated and total protein levels of TBK1, STING and IRF3 in cardiomyocytes among multiple groups, n = 6 per group. For statistical comparisons, we initially evaluated data for normality and equality of variances. One-way ANOVA was used to determine the levels of statistical differences between multiple groups. Data were expressed as mean±SEM, **p*<0.05, ***p*<0.01, ****p*<0.001, *****p*<0.0001.

(Q12) Fig. 6H: The cells in the "Vehicle" condition should be centered for clarity.

Response:

This has been done. We have revised the image in the "Vehicle" condition (Fig. 6H in the revised Manuscript, which is inserted below for the Reviewer's convenience).

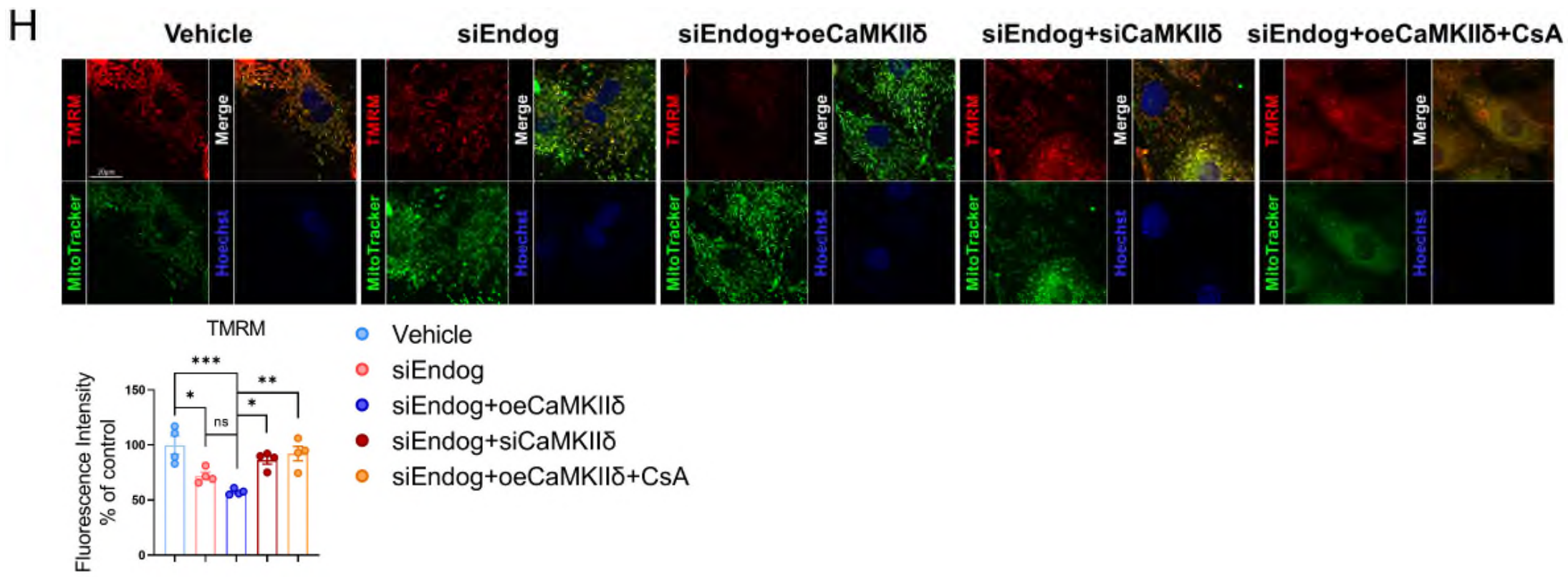

Figure 6H. *O*-GlcNAcylation drove mPTP opening through CaMKIIδ under hyperglycemia. Experiments were performed using NRCMs isolated from E19.5 rat hearts. CaMKIIδ siRNA or CaMKIIδ overexpression plasmid was transfected into NRCMs for 48 hours. CsA was applied on NRCMs at 10 μM for the final 4 hours. (H) Representative fluorescence images of cardiomyocytes stained with TMRM, MitoTracker and Hoechst33342 for 20 mins (left), scale bar = 20 μm. The statistically quantified data on the fluorescence intensity (right), n = 4 per group. For statistical comparisons, we initially evaluated data for normality and equality of variances. One-way ANOVA was used to determine the levels of statistical differences between multiple groups. Data were expressed as mean±SEM, *$p<0.05$, **$p<0.01$, ***$p<0.001$, ****$p<0.0001$.

(Q13) Fig. 7: The rationale for adding LPS is unclear. Does it enhance mitochondrial injury and mtDNA leakage without TLR4 CaMKII signaling? Providing a clearer explanation would benefit readers.

Response:

Thanks for your constructive suggestions.

The rationale for using LPS in Figure 7 and Supplementary Fig. 4 was to model the physiological postnatal exposure to microbial components and infection-related stress that all neonates typically face after birth[1,2]. Notably, studies have demonstrated that neonates born to mothers with gestational diabetes mellitus exhibited a higher susceptibility to infections[3-6]. This increased vulnerability is thought to result from impaired immune competence due to metabolic disturbances, including hyperglycemia[7-9]. Therefore, in order to simulate the clinical context in which both normal and GDM-exposed neonates encounter postnatal microbial challenges, a single intraperitoneal injection of LPS[10-12] was administered. This model enabled us to assess whether offspring subjected to intrauterine hyperglycemia exhibit an exaggerated myocardial response to inflammatory stimuli, thereby revealing the potential interaction between prenatal metabolic insults and postnatal immune stress. The timing of the intraperitoneal LPS injection was optimized to coincide with peak physiological effects: LPS-induced mitochondrial injury and cytokine release peak within 1-6 hours post-injection, as validated by temperature and immune marker kinetics in mammalian models[10,12,13]. This design captures the critical window where prenatal metabolic insults exacerbate postnatal immune stress, a phenomenon documented in both clinical cohorts and experimental systems.

Following your suggestions, we have revised the **Results** section as follows: "Notably, previous studies have demonstrated that neonates born to mothers with GDM exhibit a higher susceptibility to infections[3-6]. This increased vulnerability is thought to result from impaired immune competence due to metabolic disturbances, including hyperglycemia[7-9]. To model postnatal immune challenges[1,2], we administered a single intraperitoneal injection

of LPS (lipopolysaccharide, 2 mg/kg) to test whether intrauterine hyperglycemia exacerbated cardiac inflammatory responses to secondary stressors. This model enabled us to assess whether offspring subjected to intrauterine hyperglycemia exhibit an exaggerated myocardial response to inflammatory stimuli, thereby revealing the potential interaction between prenatal metabolic insults and postnatal immune stress." (lines 387-390 of the revised manuscript).

LPS has been reported to trigger mitochondrial injury and mtDNA leakage through the TLR4/CaMKII signaling[14]. Consistently, our results comparing the CON+LPS and CON+PBS groups (Fig. 7) align with these findings. LPS can also enhance mitochondrial injury and mtDNA leakage without TLR4 CaMKII signaling, which is evidenced by previously reported LPS-induced mitochondrial injury *via* the caspase-11/GSDMD signaling pathway[15] and the NOX4/ROS/NF-κB signaling pathway[16]. Furthermore, as demonstrated in Fig. 7, the HG+LPS group resulted in more severe cardiac damage compared to the CON+LPS group, suggesting that HG can induce CaMKII activation and subsequent mtDNA damage, thereby exacerbating the mtDNA damage induced by LPS (Fig. 7 in the revised Manuscript, inserted below for the Reviewer's convenience).

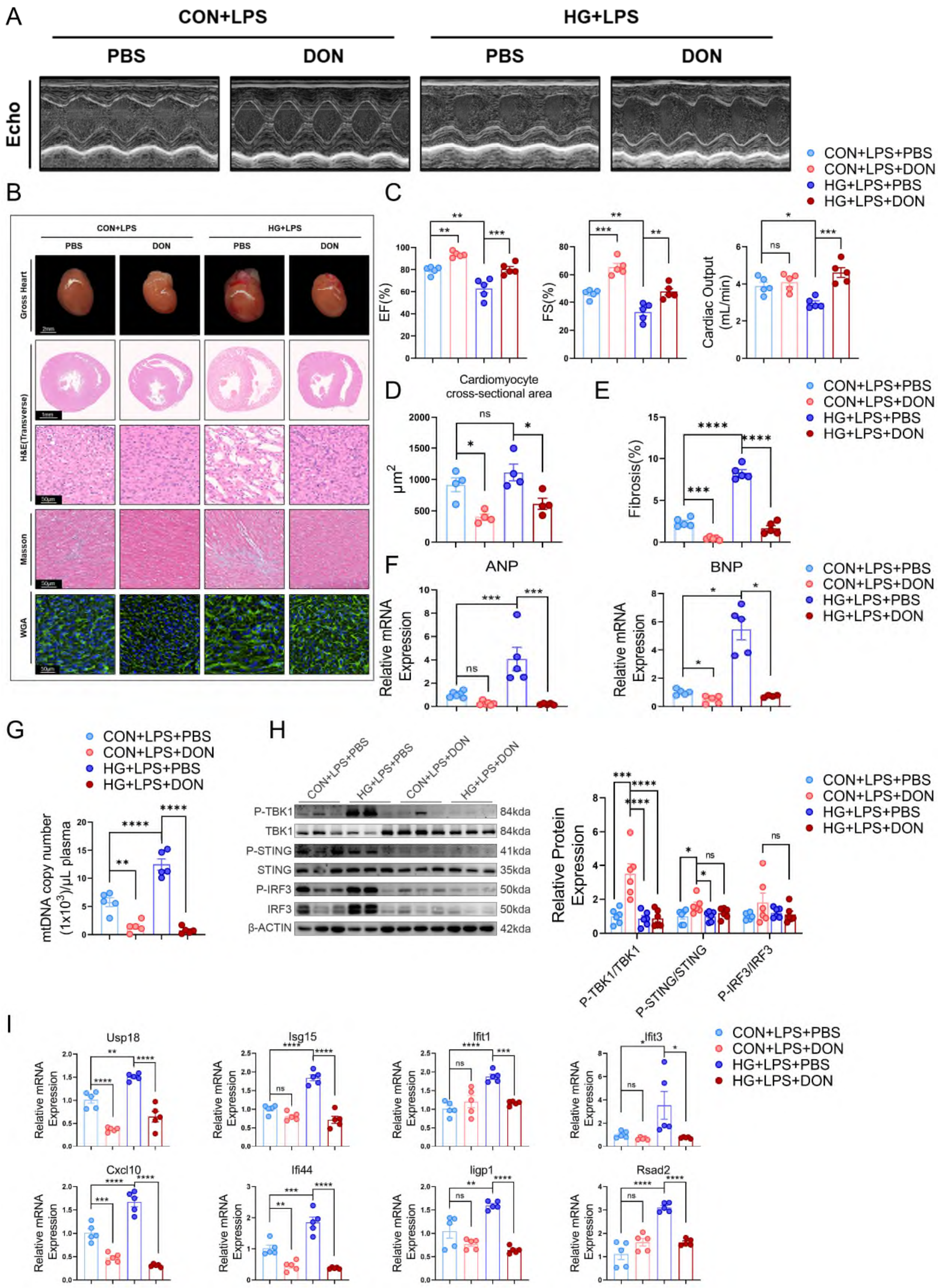

Figure 7. DON attenuated inflammatory damage induced by mtDNA release in hyperglycemic neonatal rat hearts. Neonatal rats received daily intraperitoneal injections of either PBS or DON (1 mg/kg/day) from postnatal day 3 to day 7, followed by a single LPS injection (2 mg/kg, i.p.) on day 7. All experiments in Fig. 7 were performed using heart tissues of P7 neonatal rats, except for the plasma mtDNA copy number experiment. (A-B) Echocardiography, gross morphology and histological analyses were conducted for multiple groups of fetal hearts. Gross morphology, scale bar = 2.0 mm; HE, Masson and WGA staining, scale bar = 50 μm. (C) Echocardiographic parameters were measured. (D-E)

Quantitative analysis was performed on cardiomyocyte cross-sectional area (WGA staining) and fibrotic area (Masson staining), n = 4-5 per group. (F) ANP and BNP gene expression was quantified by RT-qPCR. (G) Plasma mtDNA copy number was assessed, n = 4-5 per group. (H) Phosphorylated and total protein levels in hearts from multiple groups were detected by western blotting (left). Densitometric quantification of the blots (right), n = 6 per group. (I) RNA expression levels of *Usp18*, *Isg15*, *Ifit1*, *Ifit3*, *Cxcl10*, *Ifi44*, *Iigp1* and *Rsad2* were quantified by RT-qPCR, n = 5 per group. For statistical comparisons, data were initially evaluated for normality and equality of variances. One-way ANOVA was used to determine statistical differences among multiple groups. If equal variances were not assumed, Welch's ANOVA was applied. Data were expressed as mean±SEM, *p<0.05, **p<0.01, ***p<0.001, ****p<0.0001.

References:

1. Torow, N., Marsland, B. J., Hornef, M. W. & Gollwitzer, E. S. Neonatal mucosal immunology. *Mucosal Immunol* **10**, 5-17 (2017).
2. Tamburini, S., Shen, N., Wu, H. C. & Clemente, J. C. The microbiome in early life: implications for health outcomes. *Nat Med* **22**, 713-722 (2016).
3. Liu, J. *et al.* Gestational diabetes exacerbates intrauterine microbial exposure induced intestinal microbiota change in offspring contributing to increased immune response. *Nutr Diabetes* **14**, 87 (2024).
4. Liu, C. *et al.* Multiple perinatal characteristics affect the association between maternal diabetes status and early neonatal gut microbiota. *mSphere* **10**, e0091424 (2025).
5. Al-Shahrani, A. M. Predictors of neonatal intensive care unit admission and adverse outcomes related to gestational diabetes. *Cureus* **15**, e38579 (2023).
6. Wang, B. B. & Xue, M. Early neonatal complications in pregnant women with gestational diabetes mellitus and the effects of glycemic control on neonatal infection. *World J Diabetes* **14**, 1393-1402 (2023).
7. Shao, W. *et al.* Understanding the link between different types of maternal diabetes and the onset of autism spectrum disorders. *Diabetes Metab* **50**, 101543 (2024).
8. Jimenez-Escutia, R. *et al.* High glucose promotes inflammation and weakens placental defenses against E. coli and S. agalactiae infection: Protective role of insulin and metformin. *Int J Mol Sci* **24** (2023).
9. Xiong, Y. *et al.* Aberrant NK cell profile in gestational diabetes mellitus with fetal growth restriction. *Front Immunol* **15**, 1346231 (2024).
10. Planavila, A. *et al.* Fibroblast growth factor 21 protects the heart from oxidative stress. *Cardiovasc Res* **106**, 19-31 (2015).
11. Sun, Y. *et al.* Beclin-1-dependent autophagy protects the heart during sepsis. *Circulation* **138**, 2247-2262 (2018).
12. Wang, Y. *et al.* beta(1)-adrenoceptor stimulation promotes LPS-induced cardiomyocyte apoptosis through activating PKA and enhancing CaMKII and IkappaBalpha phosphorylation. *Crit Care* **19**, 76 (2015).
13. Cuskelly, A. *et al.* Neonatal immune challenge influences the microbiota and behaviour in a sexually dimorphic manner. *Brain Behav Immun* **103**, 232-242 (2022).
14. Han, W. M. *et al.* NMDARs antagonist MK801 suppresses LPS-induced apoptosis and mitochondrial dysfunction by regulating subunits of NMDARs via the CaM/CaMKII/ERK pathway. *Cell Death Discov* **9**, 59 (2023).

15. Zhu, F. *et al.* The orphan receptor Nur77 binds cytoplasmic LPS to activate the non-canonical NLRP3 inflammasome. *Immunity* **56**, 753-767 e758 (2023).

16. Li, J. *et al.* NOX4 is a potential therapeutic target in septic acute kidney injury by inhibiting mitochondrial dysfunction and inflammation. *Theranostics* **13**, 2863-2878 (2023).
