# Supplementary material for: Maternal hyperglycemia-induced O-GlcNAcylation of CaMKIIδ promotes mtDNA release and cardiac remodeling in offspring
Source: Nat Commun. 2026 Jul 29;17:7769. doi: 10.1038/s41467-026-75630-4 (PMC13439061; doi:10.1038/s41467-026-75630-4)

Uncropped Western Blot Images

Fig. 2a

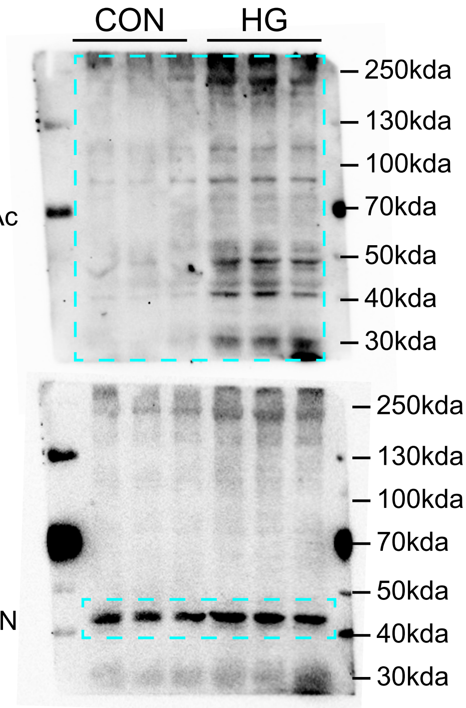

Fig. 2b

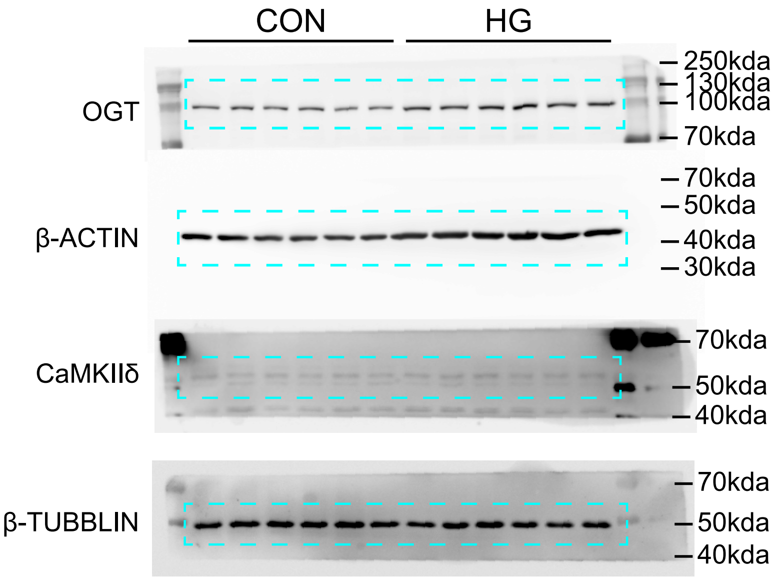

Fig. 2d

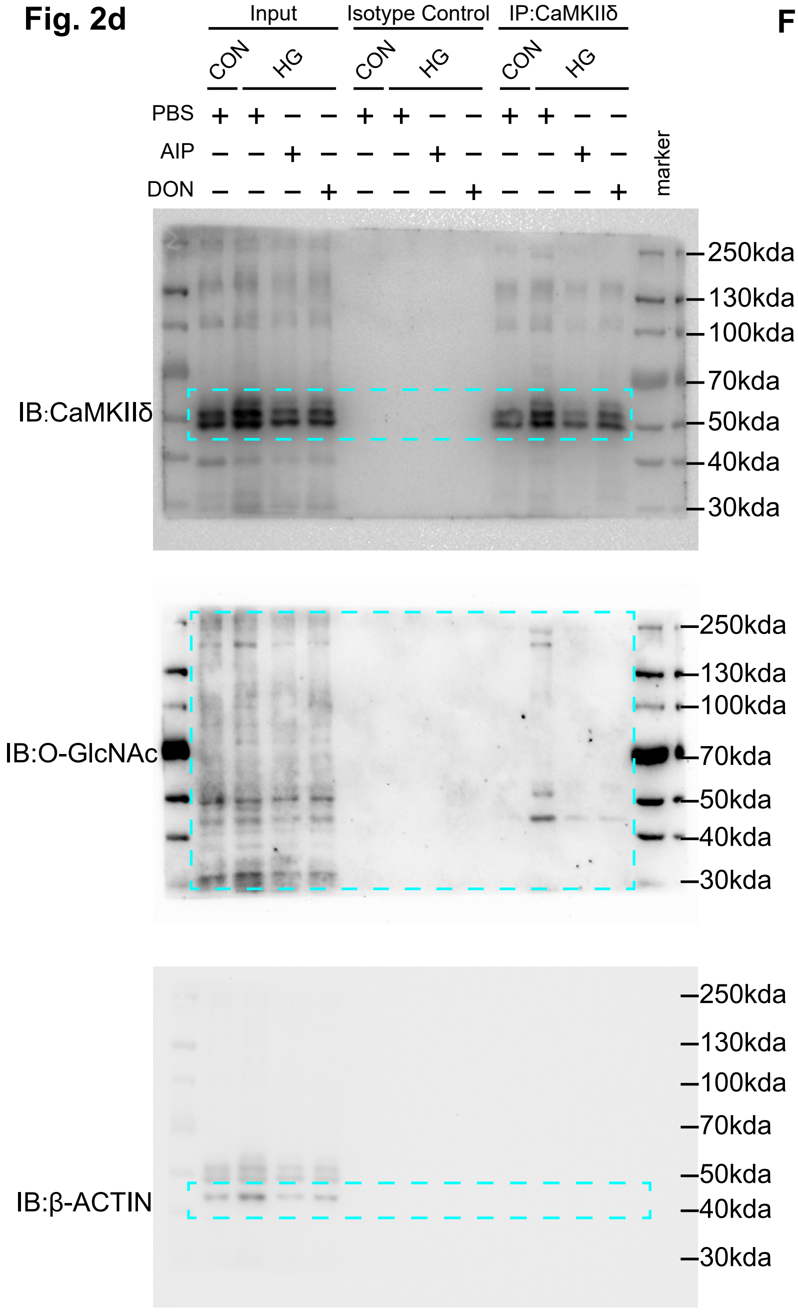

Fig. 2e

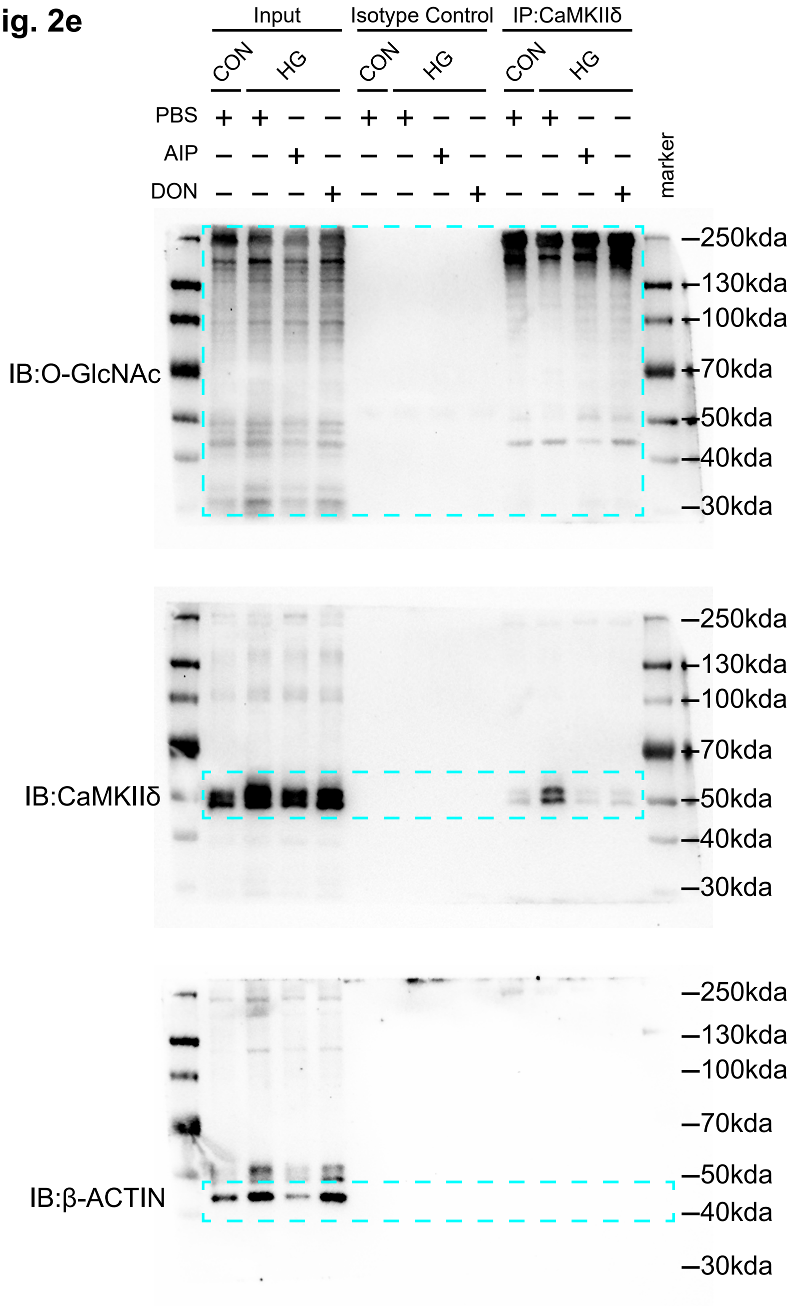

Fig. 4d

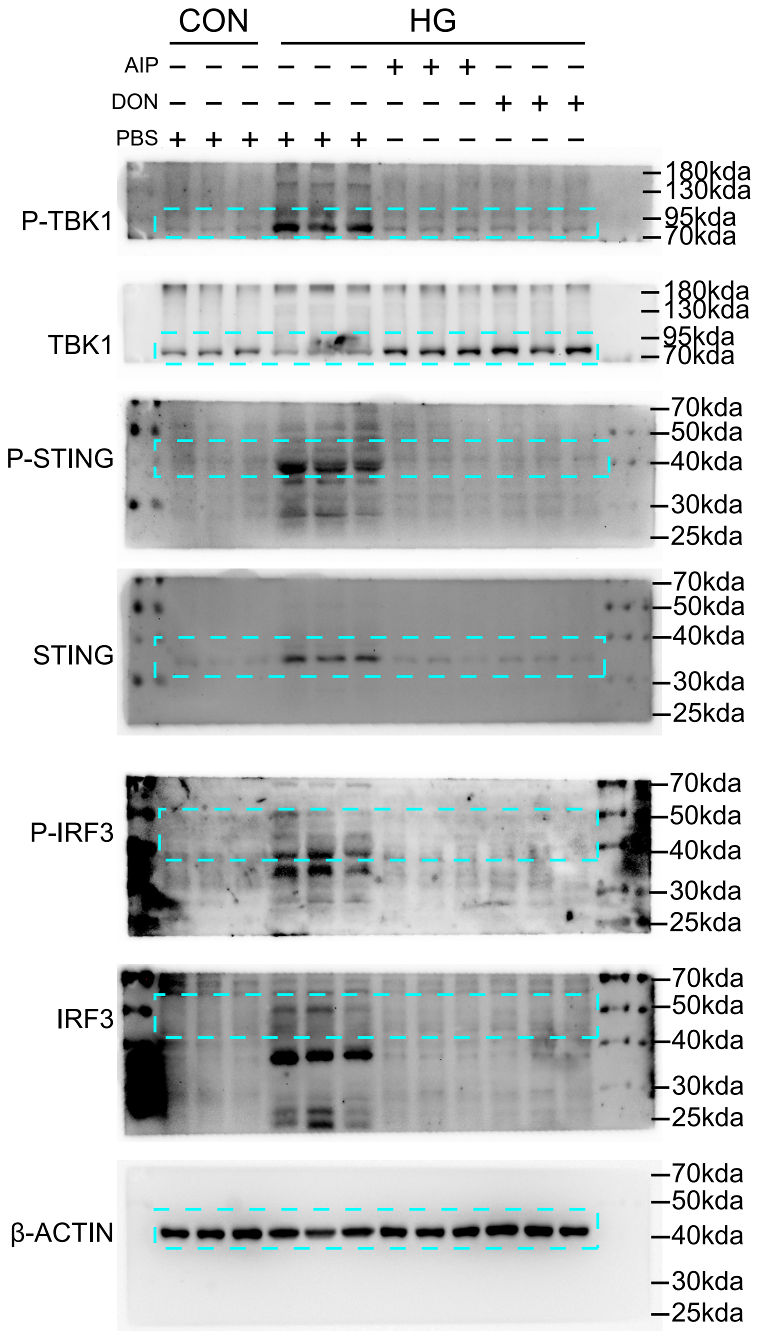
CON
HG
AIP – – – – – – + + + – – –
DON – – – – – – – – – + + +
PBS + + + + + + – – – – – –
P-TBK1
180kda
130kda
95kda
70kda
TBK1
180kda
130kda
95kda
70kda
P-STING
70kda
50kda
40kda
30kda
25kda
STING
70kda
50kda
40kda
30kda
25kda
P-IRF3
70kda
50kda
40kda
30kda
25kda
IRF3
70kda
50kda
40kda
30kda
25kda
β-ACTIN
70kda
50kda
40kda
30kda
25kda

Fig. 5e

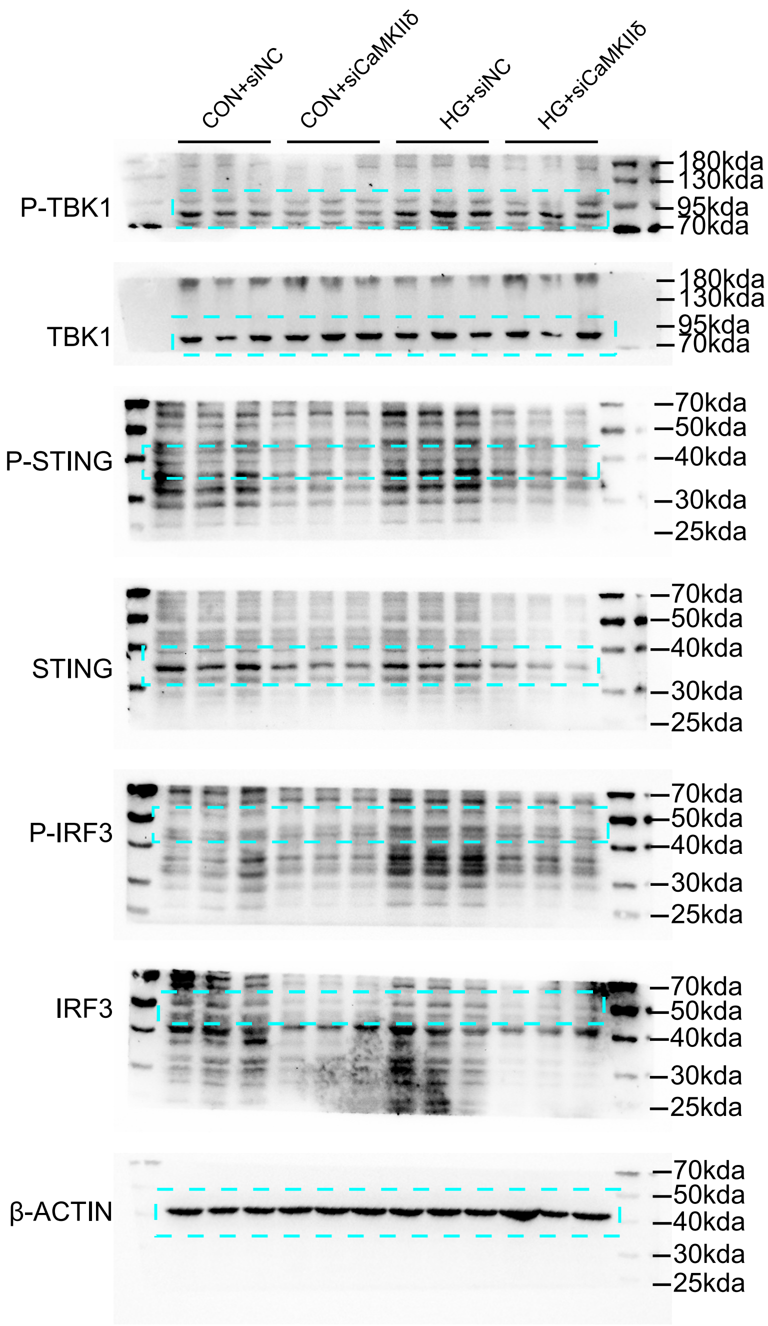
CON+siNC
CON+siCaMKIIδ
HG+siNC
HG+siCaMKIIδ
P-TBK1
TBK1
P-STING
STING
P-IRF3
IRF3
β-ACTIN
180kda
130kda
95kda
70kda
50kda
40kda
30kda
25kda

Fig. 5j

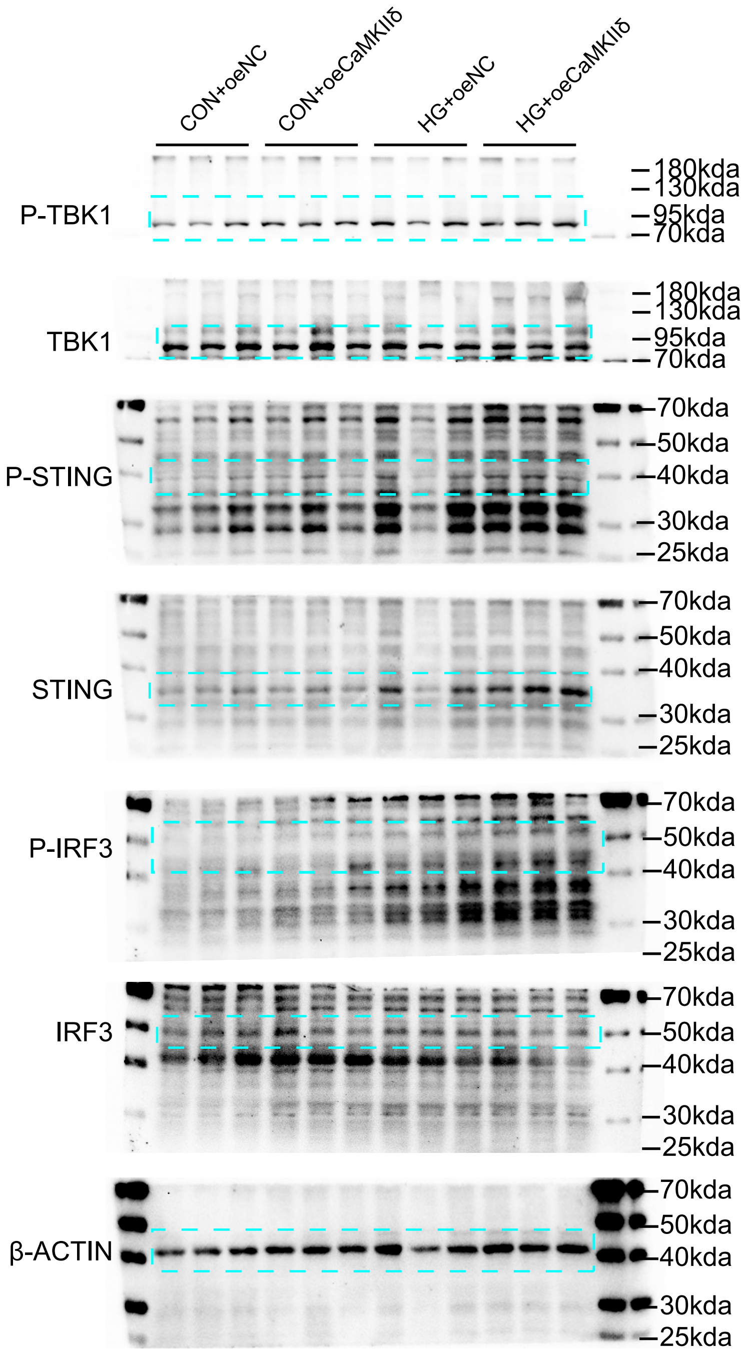
CON+oeNC
CON+oeCaMKIIδ
HG+oeNC
HG+oeCaMKIIδ
P-TBK1
TBK1
P-STING
STING
P-IRF3
IRF3
β-ACTIN
180kda
130kda
95kda
70kda
50kda
40kda
30kda
25kda

Fig. 5o

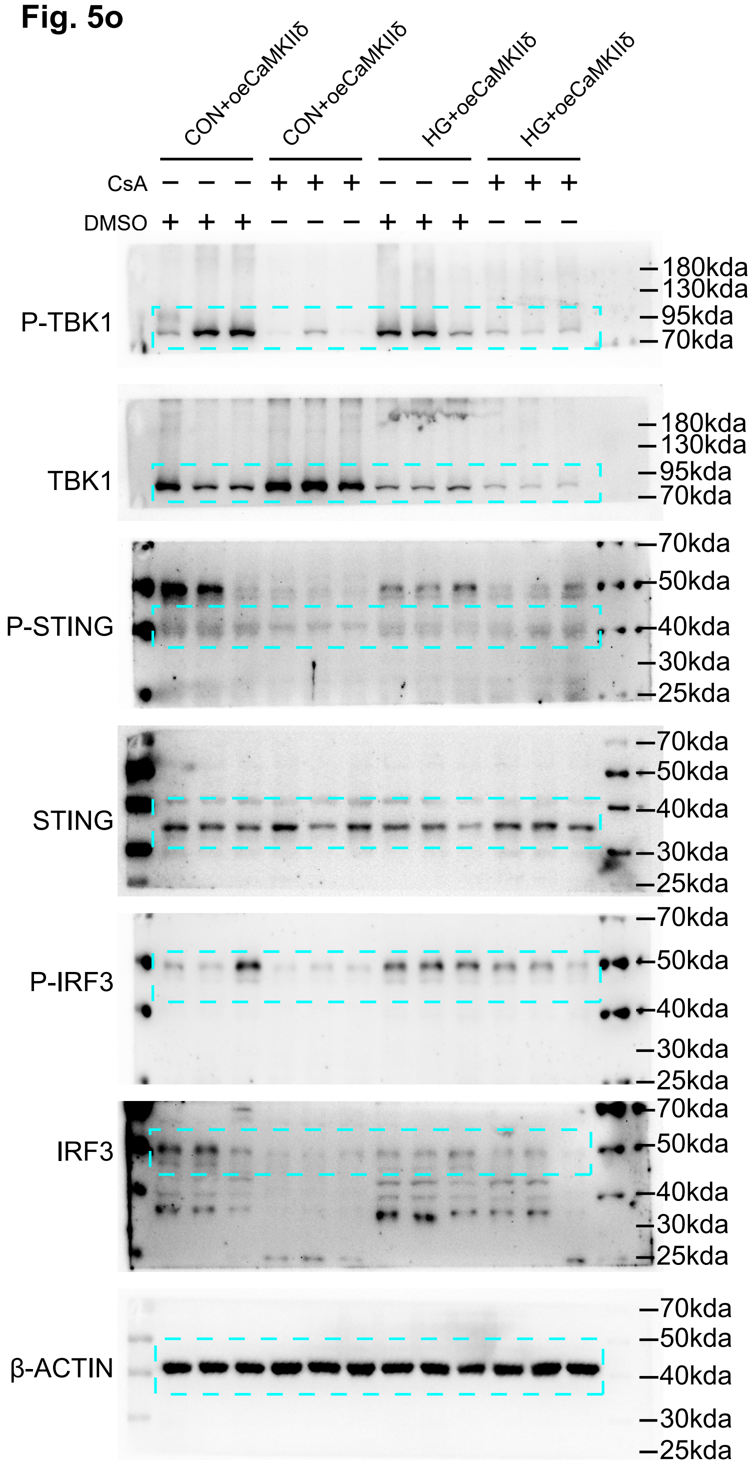
CON+oeCaMKIIδ
CON+oeCaMKIIδ
HG+oeCaMKIIδ
HG+oeCaMKIIδ
CsA − − − + + + − − − + + +
DMSO + + + − − − + + + − − −
P-TBK1
180kda
130kda
95kda
70kda
TBK1
180kda
130kda
95kda
70kda
P-STING
70kda
50kda
40kda
30kda
25kda
STING
70kda
50kda
40kda
30kda
25kda
P-IRF3
70kda
50kda
40kda
30kda
25kda
IRF3
70kda
50kda
40kda
30kda
25kda
β-ACTIN
70kda
50kda
40kda
30kda
25kda

Fig. 6d

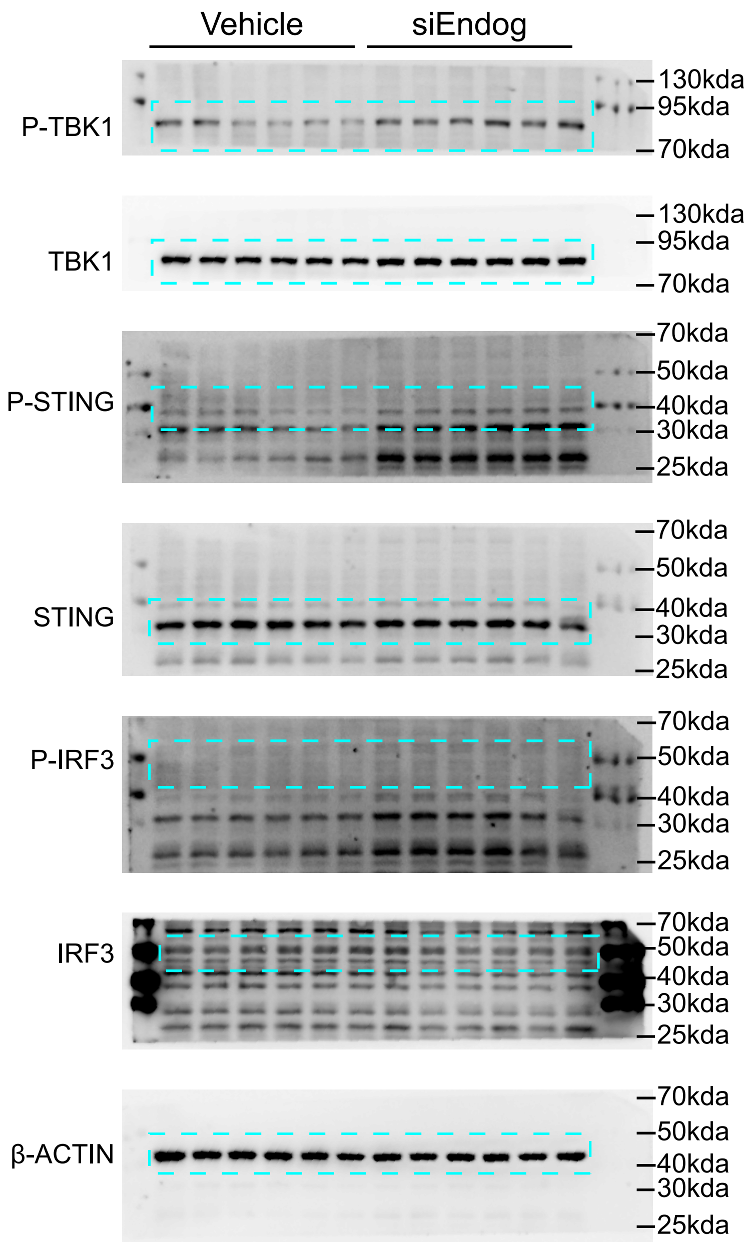
Vehicle
siEndog
P-TBK1
130kda
95kda
70kda
TBK1
130kda
95kda
70kda
P-STING
70kda
50kda
40kda
30kda
25kda
STING
70kda
50kda
40kda
30kda
25kda
P-IRF3
70kda
50kda
40kda
30kda
25kda
IRF3
70kda
50kda
40kda
30kda
25kda
β-ACTIN
70kda
50kda
40kda
30kda
25kda

Fig. 6e

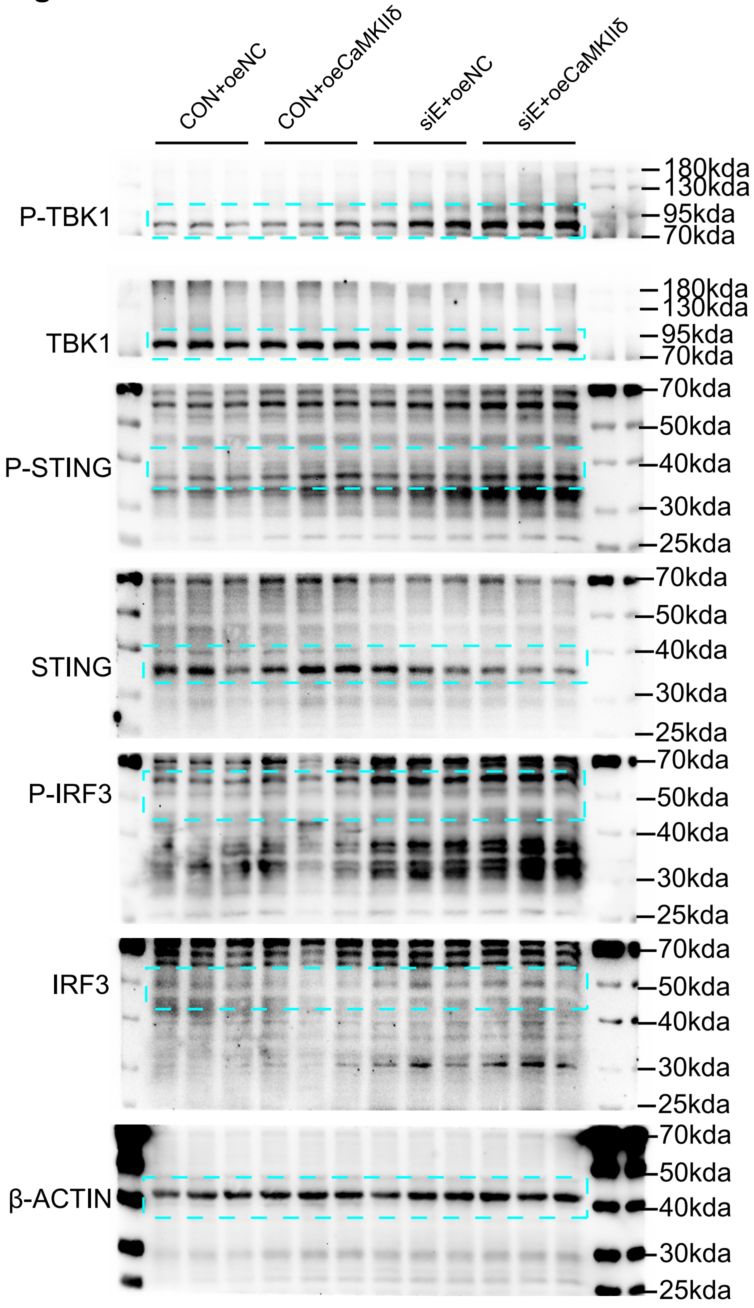
CON+oeNC
CON+oeCaMKIIδ
siE+oeNC
siE+oeCaMKIIδ
P-TBK1
180kda
130kda
95kda
70kda
TBK1
180kda
130kda
95kda
70kda
P-STING
70kda
50kda
40kda
30kda
25kda
STING
70kda
50kda
40kda
30kda
25kda
P-IRF3
70kda
50kda
40kda
30kda
25kda
IRF3
70kda
50kda
40kda
30kda
25kda
β-ACTIN
70kda
50kda
40kda
30kda
25kda

Fig. 6f

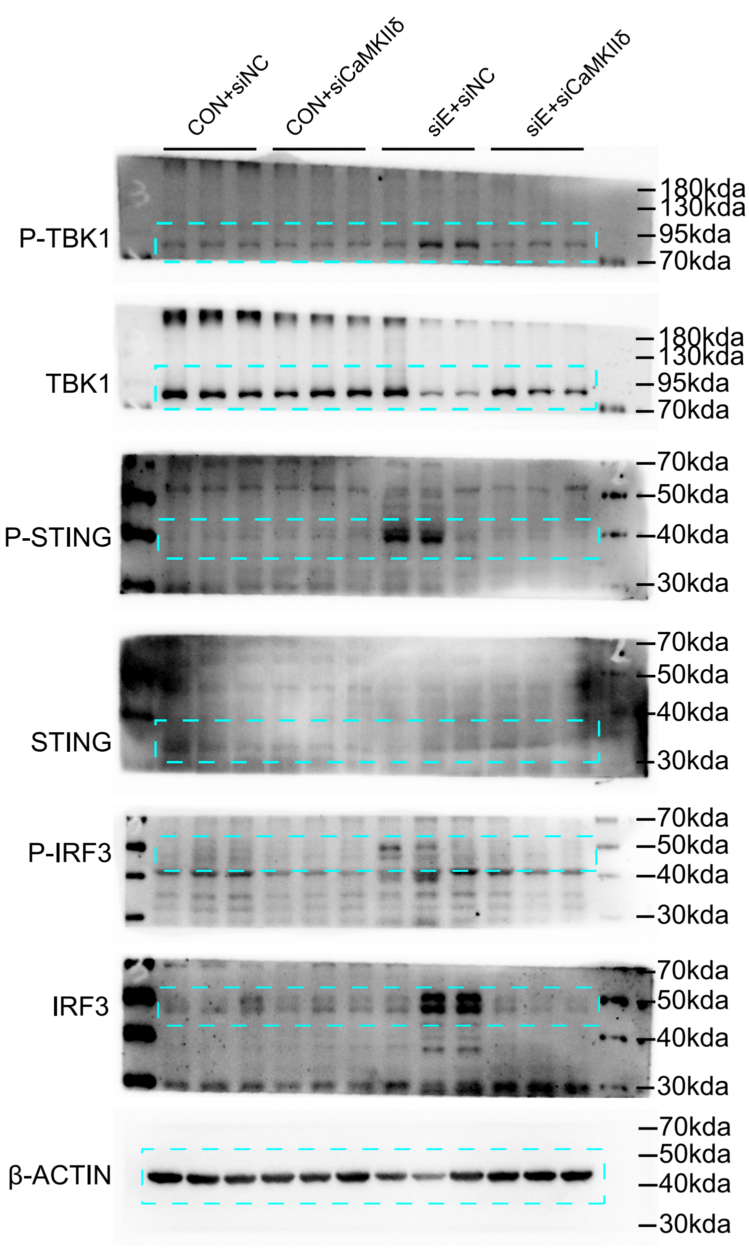
CON+siNC
CON+siCaMKIIδ
siE+siNC
siE+siCaMKIIδ
P-TBK1
TBK1
P-STING
STING
P-IRF3
IRF3
β-ACTIN
180kda
130kda
95kda
70kda
50kda
40kda
30kda

Fig. 6g

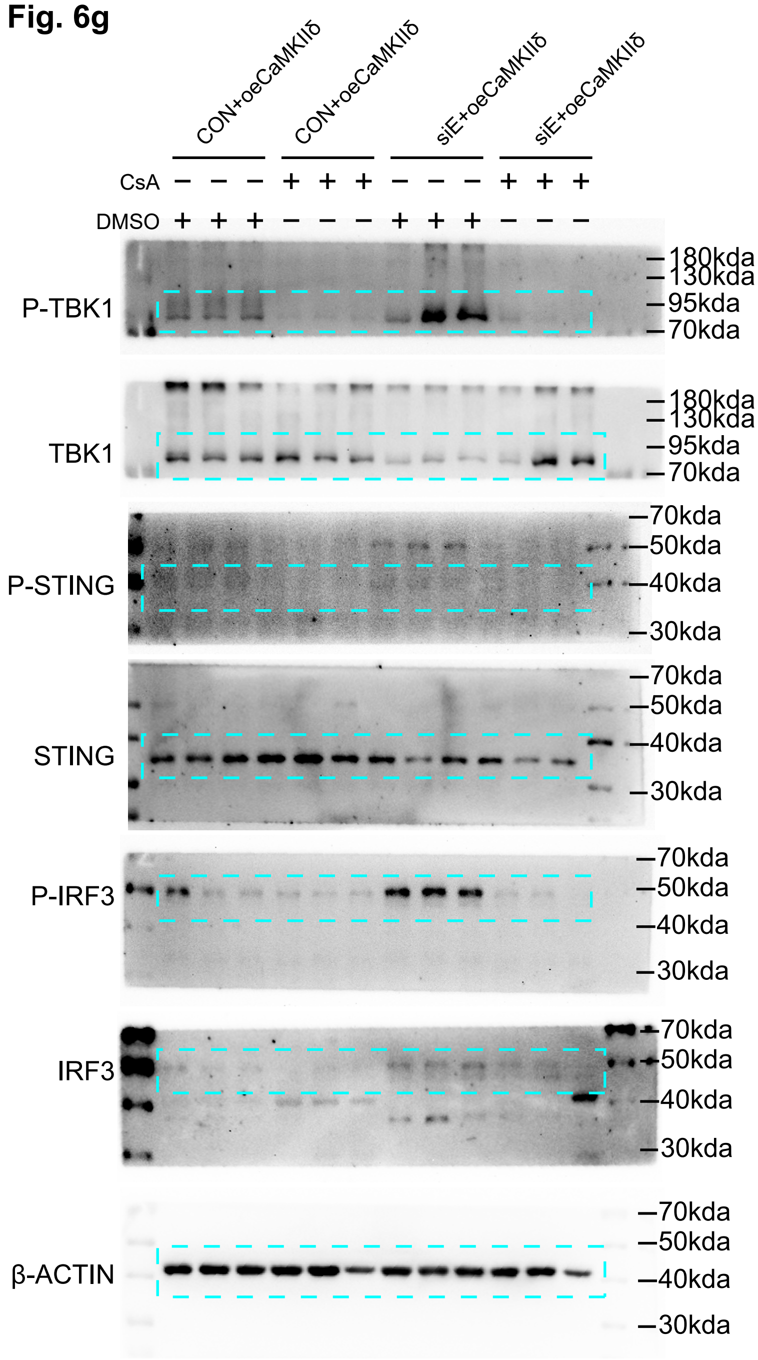
CON+oeCaMKIIδ
CON+oeCaMKIIδ
siE+oeCaMKIIδ
siE+oeCaMKIIδ
CsA – – – + + + – – – + + +
DMSO + + + – – – + + + – – –
P-TBK1
TBK1
P-STING
STING
P-IRF3
IRF3
β-ACTIN
180kda
130kda
95kda
70kda
50kda
40kda
30kda

Fig. 7h

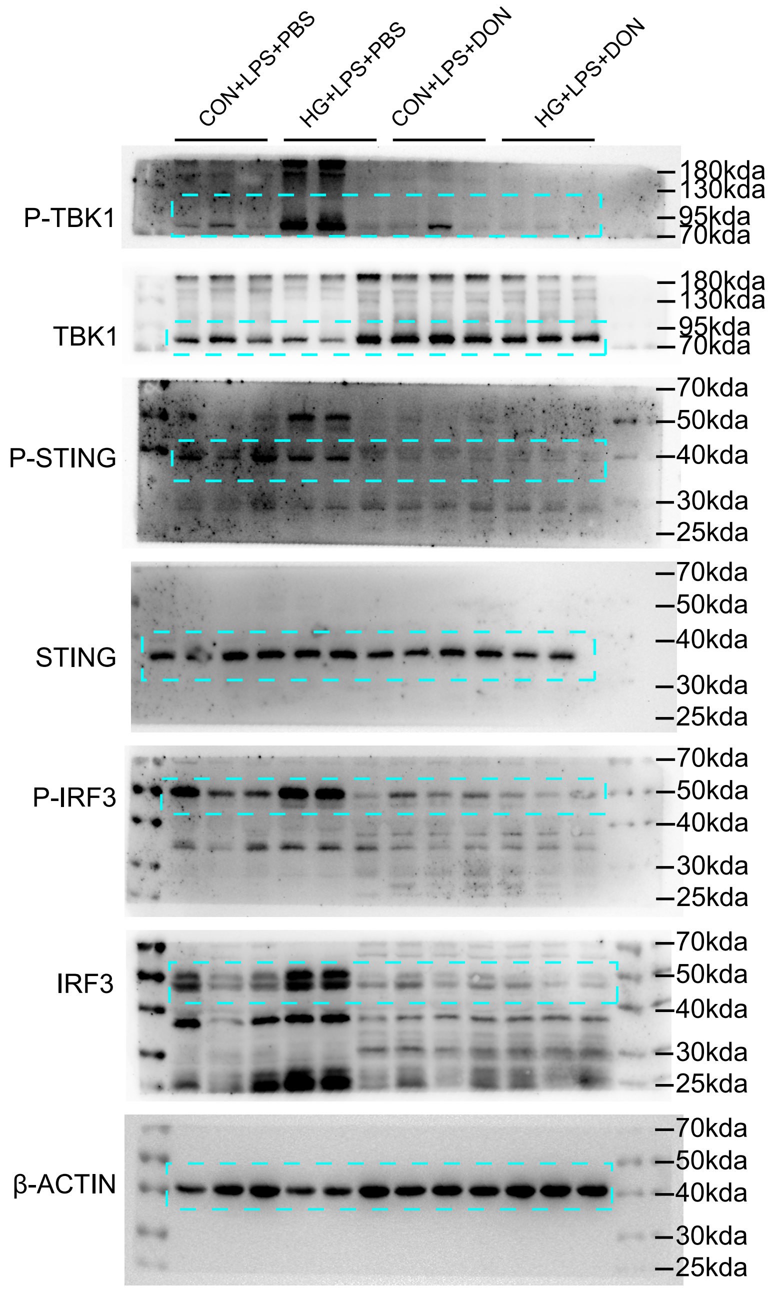

CON+LPS+PBS
HG+LPS+PBS
CON+LPS+DON
HG+LPS+DON
P-TBK1
180kda
130kda
95kda
70kda
TBK1
180kda
130kda
95kda
70kda
P-STING
70kda
50kda
40kda
30kda
25kda
STING
70kda
50kda
40kda
30kda
25kda
P-IRF3
70kda
50kda
40kda
30kda
25kda
IRF3
70kda
50kda
40kda
30kda
25kda
β-ACTIN
70kda
50kda
40kda
30kda
25kda

Supplementary Fig. 1e

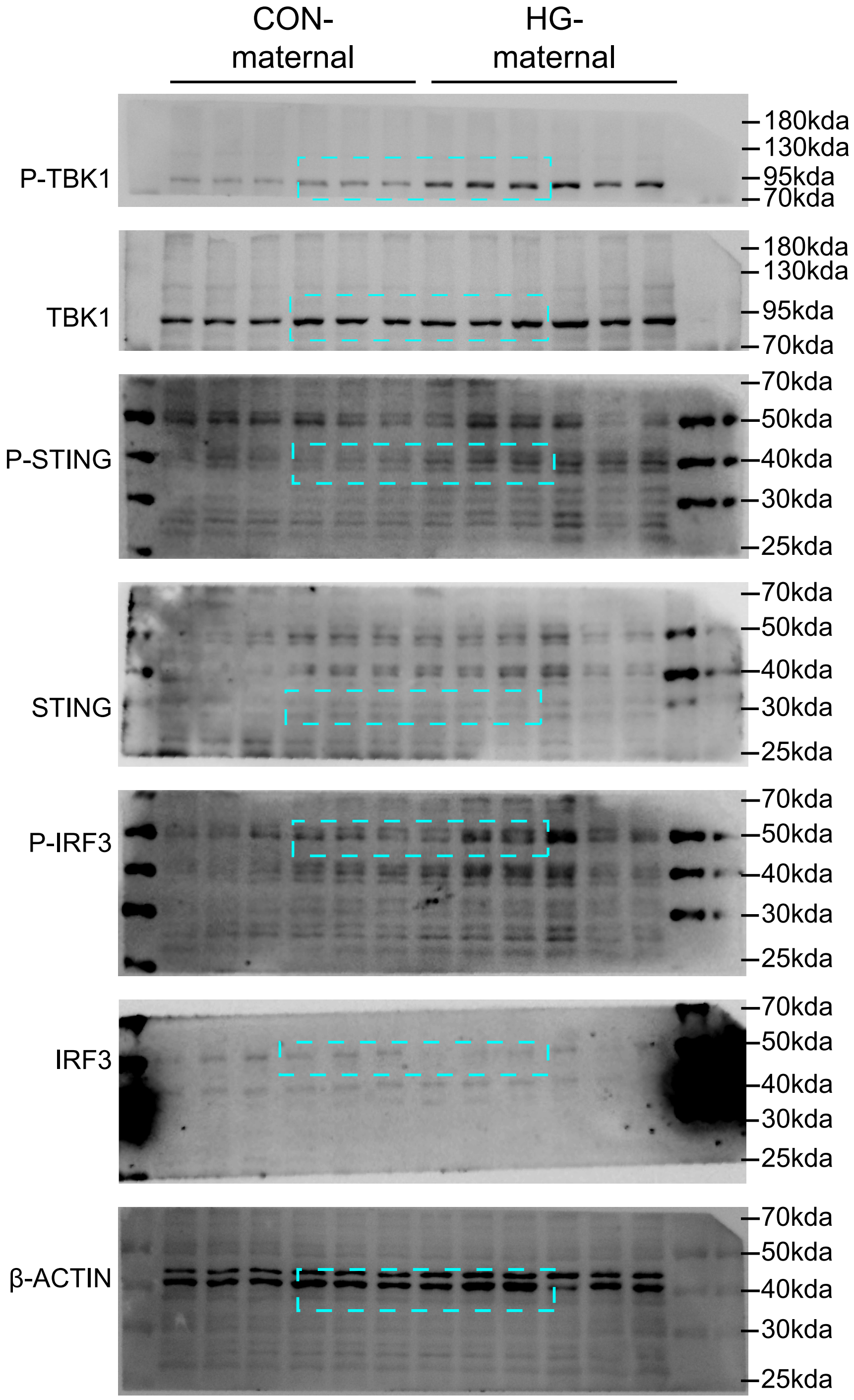

Supplementary Fig. 3b

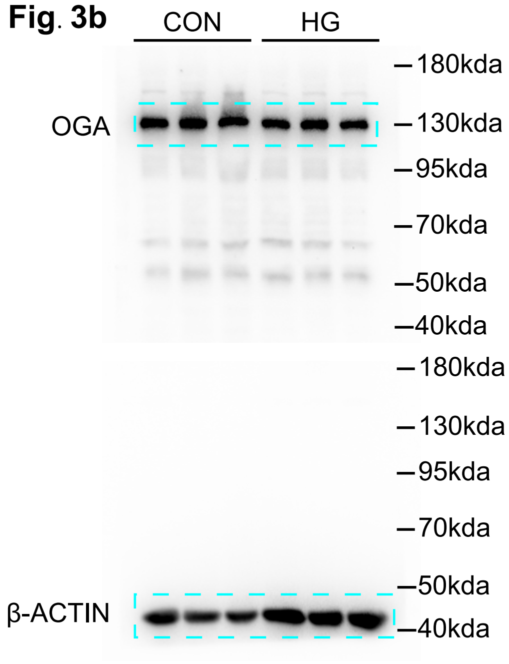

Supplementary Fig. 3d

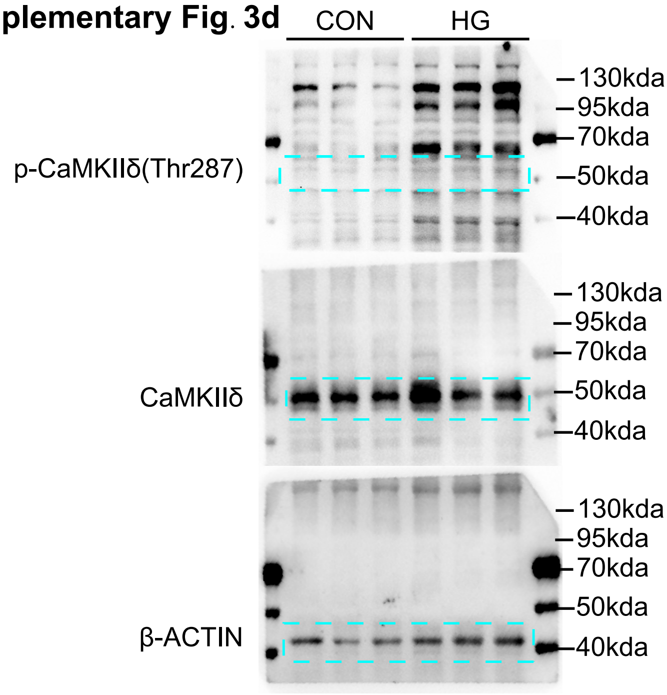

Supplementary Fig. 3e

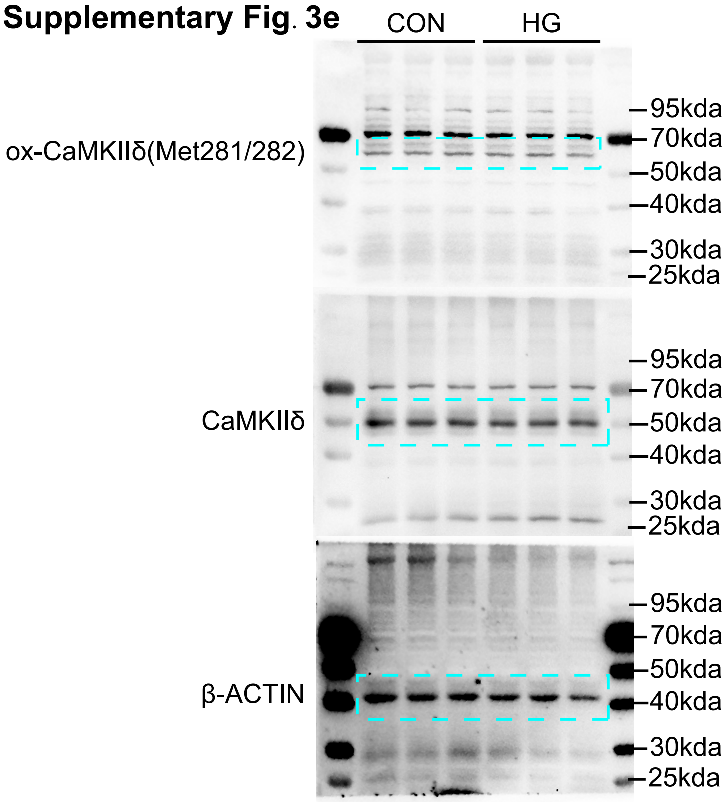

Supplementary Fig. 4a

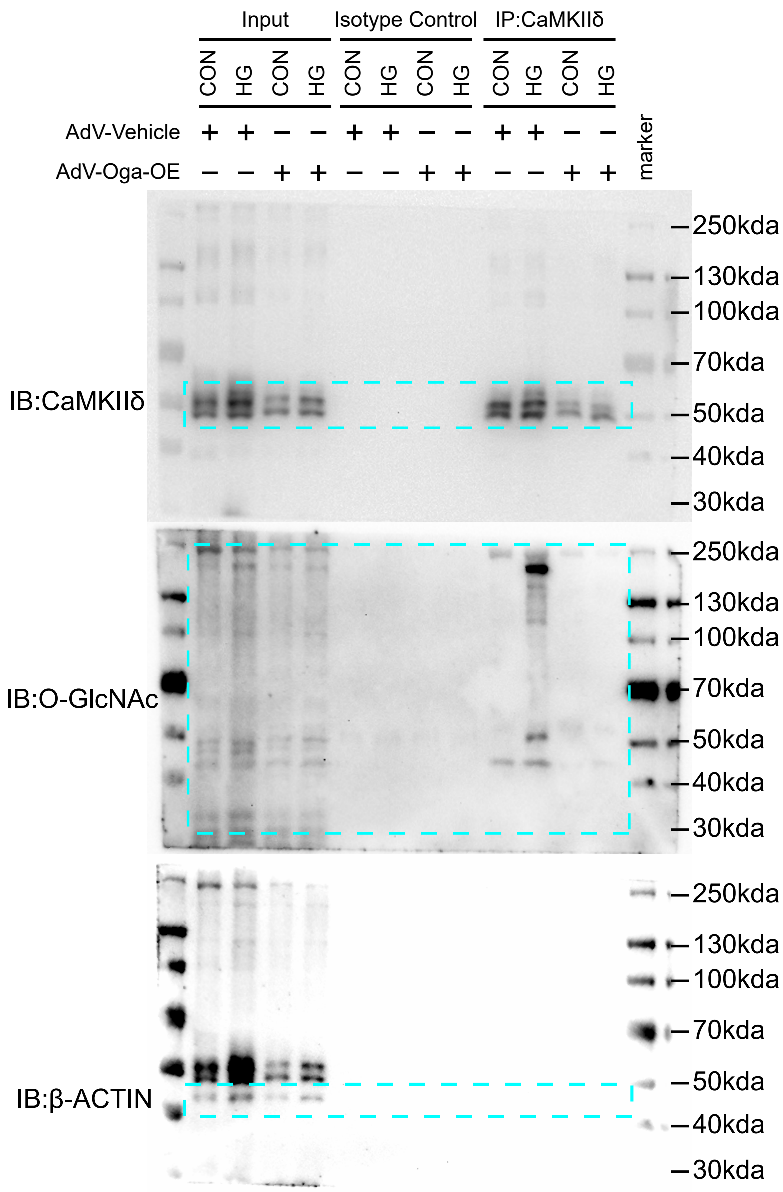

Supplementary Fig. 4c

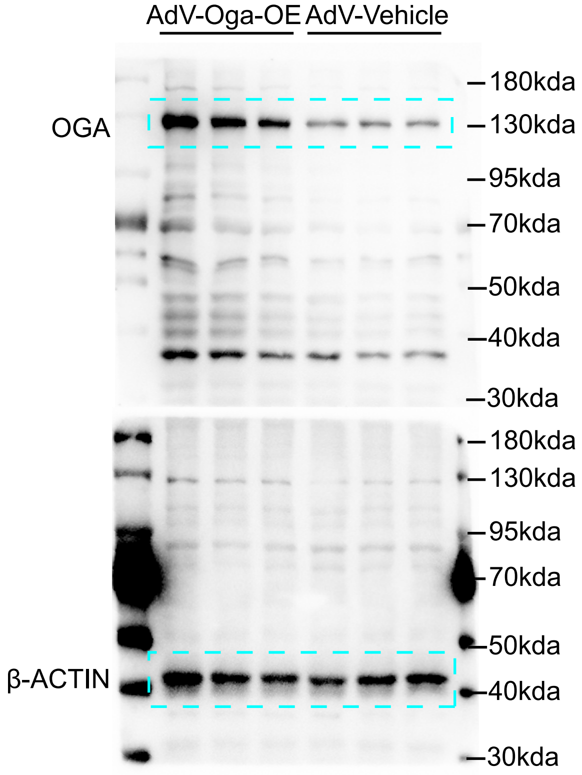

Supplementary Fig. 6b

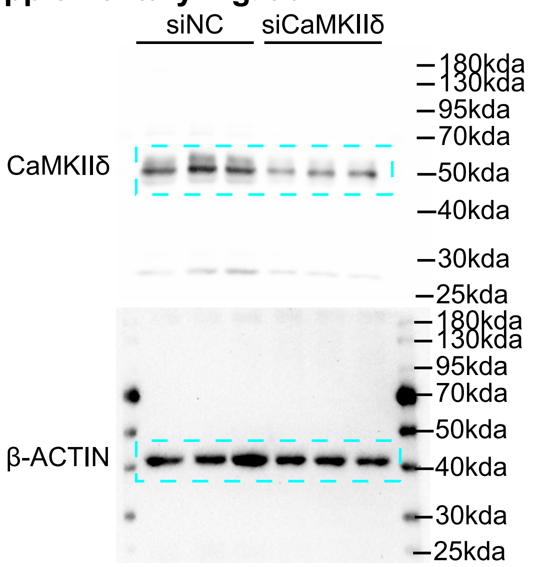

Supplementary Fig. 6e

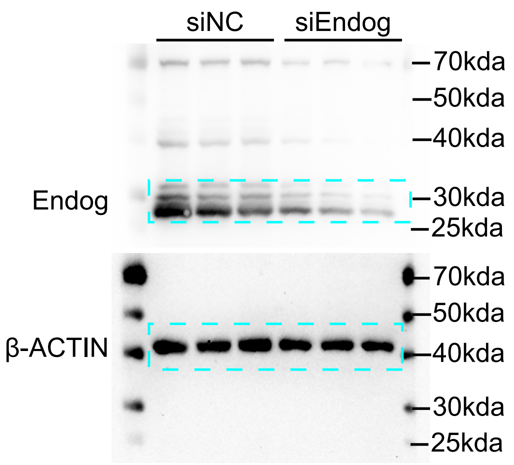

Supplementary Fig. 6g

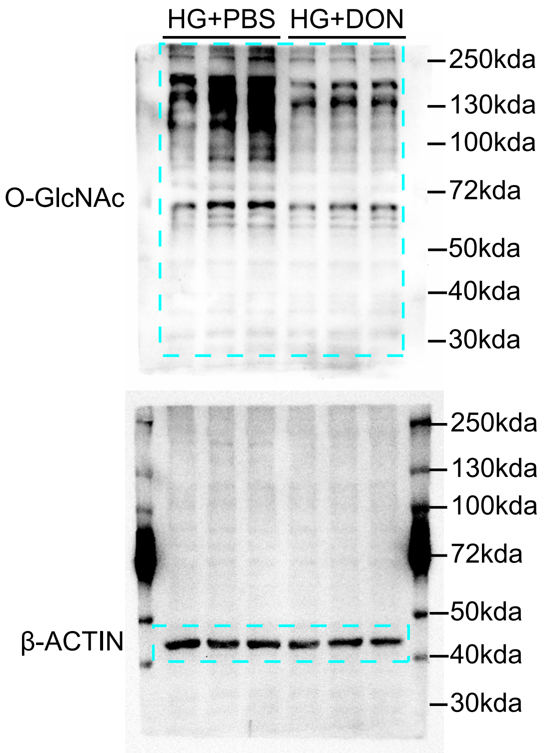

Supplementary Fig. 7c

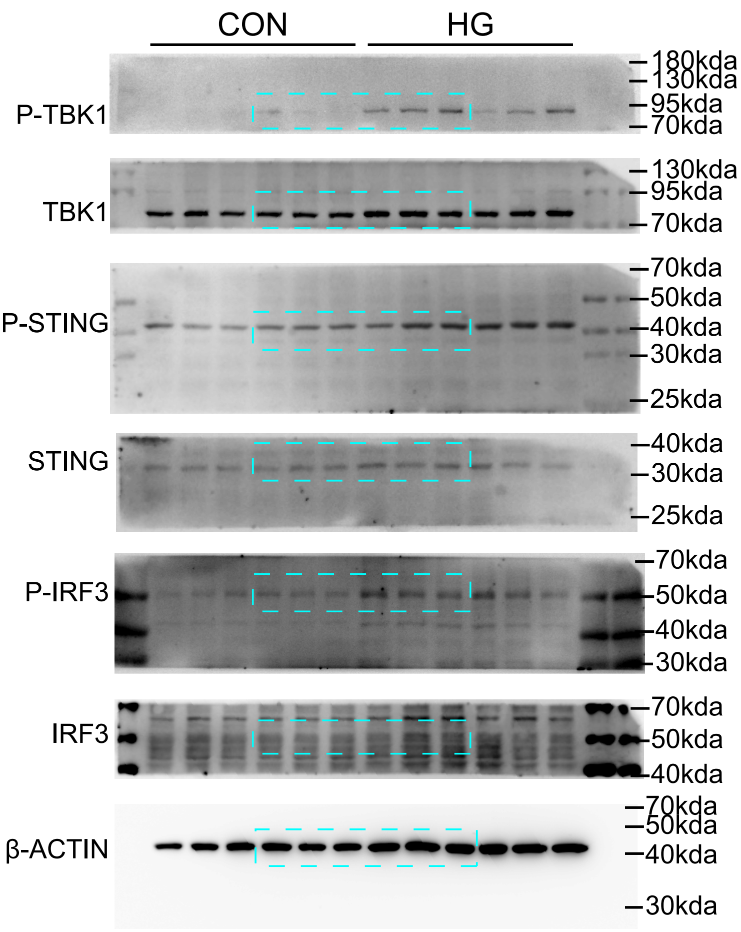

Supplementary Fig. 8g

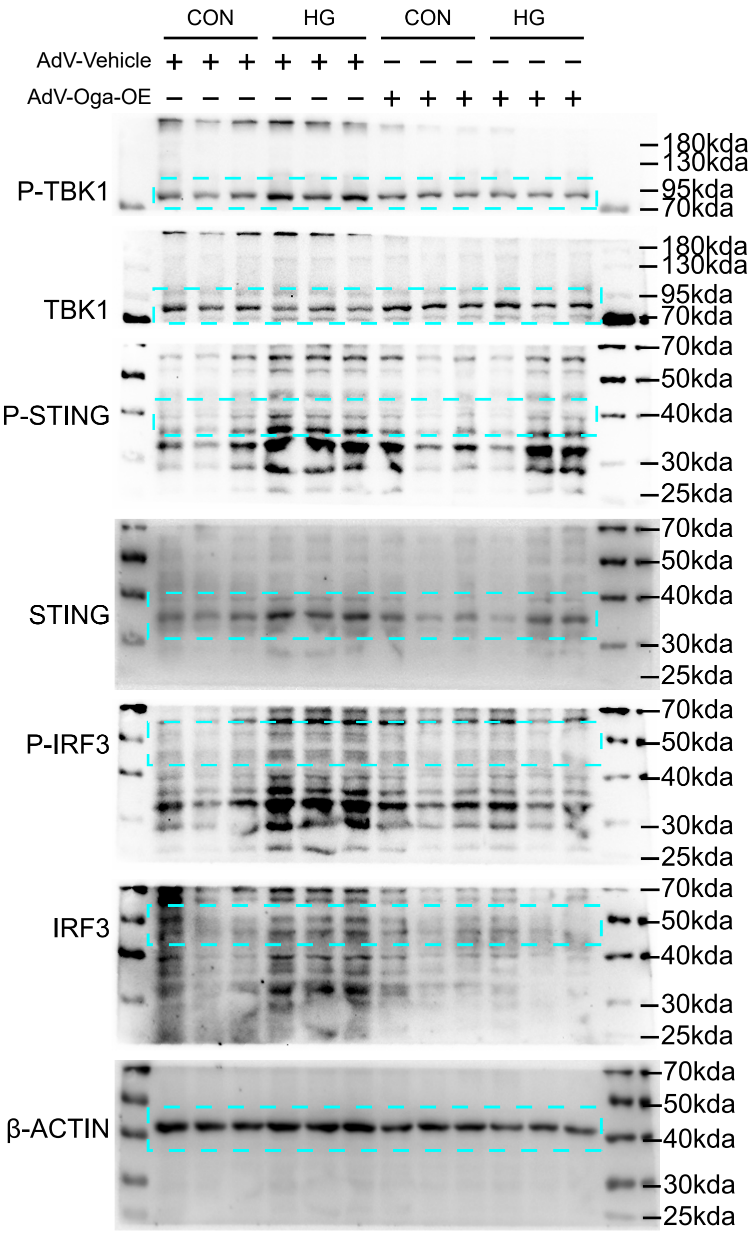

Supplementary Fig. 9a

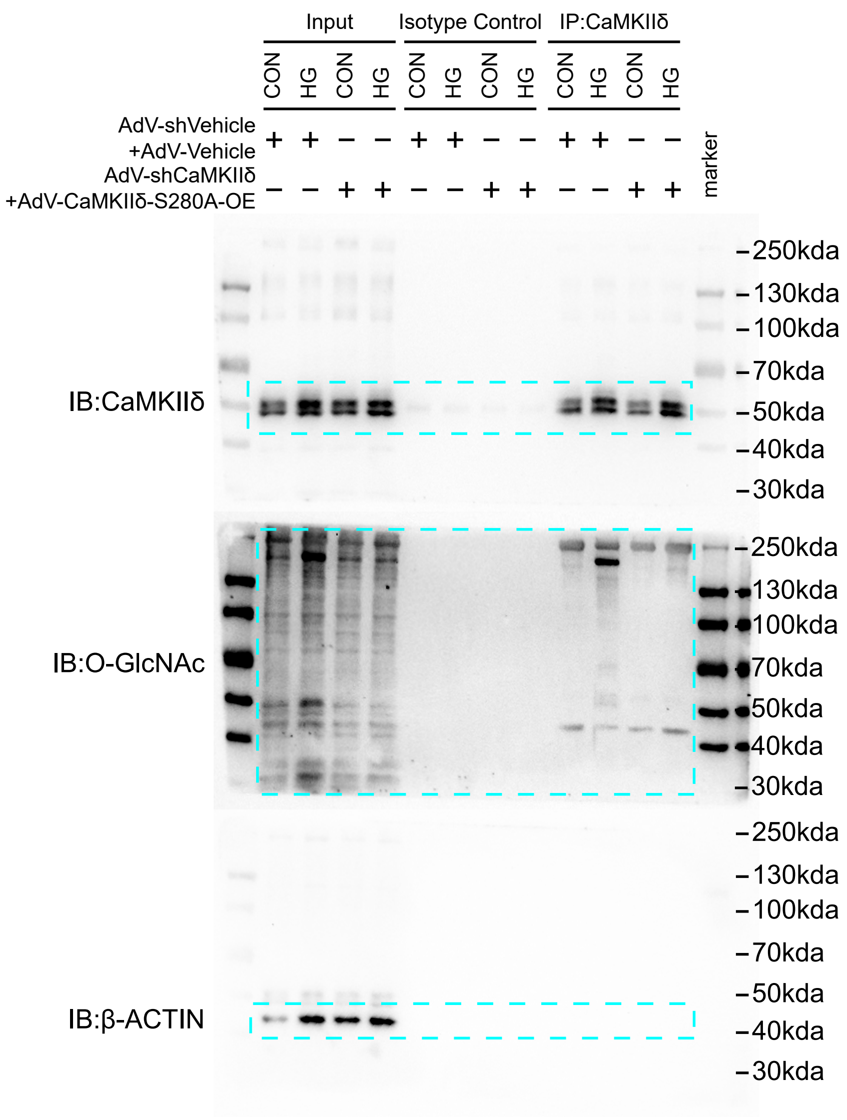

Supplementary Fig. 9e

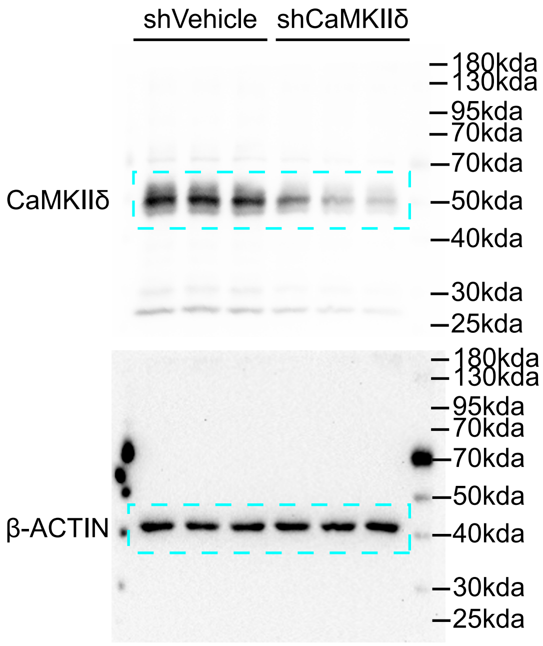

Supplementary Fig. 10g

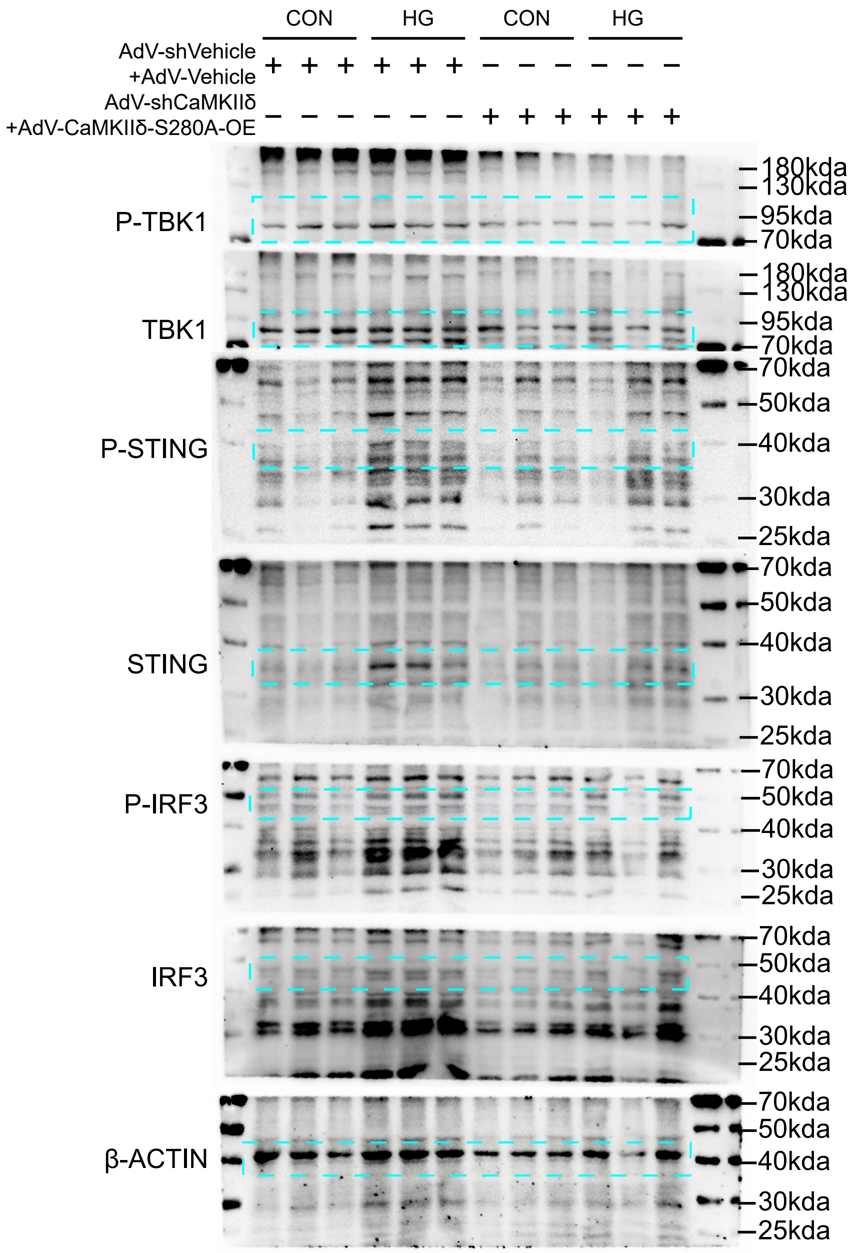

Supplementary Fig. 11h

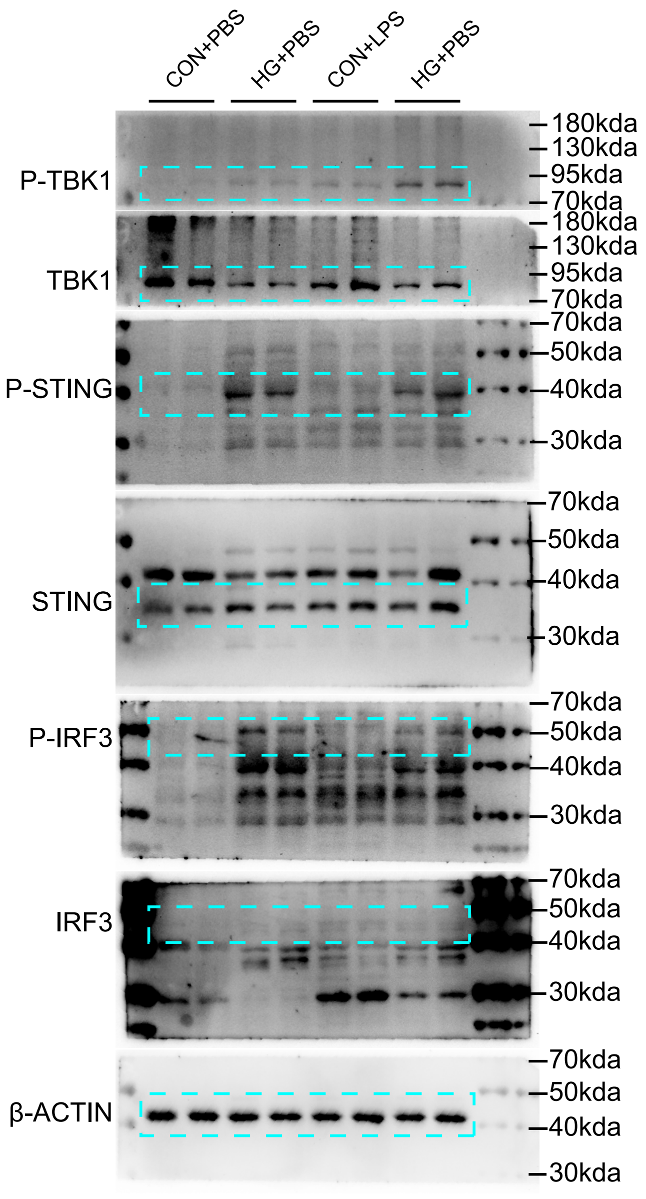

Supplement: Supplementary file 4 [file 41467_2026_75630_MOESM4_ESM.zip › Uncropped Western Blot Images.pdf]
